# Targeted Chemical Profiling and Dereplication of Australian Plants of the Family Haemodoraceae Using a Combined HPLC-MS and HRLC(ESI)-MS Approach

**DOI:** 10.3390/molecules30204044

**Published:** 2025-10-10

**Authors:** Liam Thompson, Valerie Chow, Shan Chen, Priyanka Reddy, Robert Brkljača, Colin Rix, Joseph J. Byrne, Aya C. Taki, Robin B. Gasser, Sylvia Urban

**Affiliations:** 1 Marine and Terrestrial Natural Product (MATNAP) Research Group, Department of Applied Chemistry and Environmental Science, School of Science, RMIT University, Melbourne, VIC 3001, Australia; 2 School of Chemistry, Bio21 Institute of Molecular Science and Biotechnology, University of Melbourne, Parkville, VIC 3010, Australia; 3 Monash Biomedical Imaging, Monash University, Clayton, VIC 3168, Australia; 4 Department of Veterinary Biosciences, Melbourne Veterinary School, Faculty of Science, The University of Melbourne, Parkville, VIC 3010, Australia

**Keywords:** phenylphenalenone, oxabenzochrysenone, phenylbenzoisochromenone, anti-microbial, anthelmintic, bioassay

## Abstract

Australian plants of the family Haemodoraceae have been a reliable source of new secondary metabolites, particularly those of the ‘phenylphenalenone’ class, and related chromenes and xanthones. Some of these compounds demonstrate anti-microbial properties against both Gram-negative and Gram-positive bacteria. Chemical profiling of thirty individual ethanolic extracts from six separate species of Australian plants belonging to the family Haemodoraceae was conducted using an HPLC-MS approach reinforced by HRLC(ESI)-MS. Six of the extracts were further explored by employing HRLC(ESI)-MS and the compounds present were characterised and confirmed based on a comparison to the original data. All thirty extracts were assessed for biological activity against the parasitic nematode *Haemonchus contortus* in vitro. The chemical profiling methodology adopted resulted in the identification of thirty-four previously reported compounds, identifying on average 64% of the previously reported secondary metabolites across the species *Haemodorum simulans*, *Haemodorum spicatum*, *Haemodorum brevisepalum* and *Macropidia fuliginosa*. Furthermore, compounds from the phenylbenzoisoquinolindone class were detected in the bulbs of *Haemodorum simulans* and *Haemodorum coccineum*, representing the first report of the structure class in extracts of the genus *Haemodorum*. Extracts of the *H. simulans* stems, *M. fuliginosa* bulbs and *H. distichophyllum* roots and bulbs exhibited anthelmintic activity in vitro. The chemical profiling HPLC-MS methodology adopted was successful in the rapid identification of most of the previously reported secondary metabolites across the Haemodoracae species, indicating that the analytical approach was robust. This study demonstrates the effectiveness of dereplication via HPLC-MS-based chemical profiling across six Australian Haemodoraceae species, identifying numerous known and putatively novel secondary metabolites. It also reports, for the first time, anthelmintic activity in selected species and marks the first detailed phytochemical investigation of *H. distichophyllum* since its initial pigment analysis over 50 years ago.

## 1. Introduction

### 1.1. Background

The family Haemodoraceae, consists of fifteen genera including the genus *Haemodorum*, known colloquially as the ‘bloodroots’ or ‘bloodworts’, the monospecific genus *Macropidia*, which, along with the genus *Anigozanthos* are often described by their common name as ‘kangaroo paws’ due to the distinct appearance of the flowering aerial components of the various species [1]. Predominantly found within the southern hemisphere, plants of the Haemodoraceae family have been attractive candidates for study due to documented ethnopharmacological uses as well as the distinct pigmentation often observed in the large rhizomes which are particularly characteristic of the genus *Haemodorum* [2,3,4].

**Figure 1:**
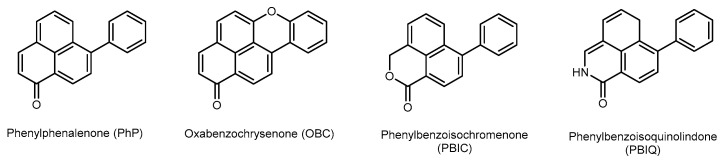
Main structure classes derived from the family Haemodoraceae.

Prior studies of the family, conducted by the Marine and Terrestrial Natural Product (MATNAP) group at RMIT University, have to date yielded the discovery of twenty-nine new compounds, primarily of the ‘phenylphenalenone’ (PhP) structure type including derivatives such as ‘oxabenzochrysenones’ (OBCs) and ‘phenylbenzoisochromenones’ (PBICs). These compound classes, along with ‘phenylbenzoisoquinolindiones’ (PBIQs) (Figure 1), are the main secondary metabolites of interest isolated from the family Haemodoraceae (collectively referred to henceforth as ‘PhP-type compounds’) and are notable for their distinct chromophores as well as their documented anti-microbial activity [1,5].

Each of these PhP-related structure classes exhibit distinctive, characteristic UV-visible chromophores. This includes the phenylphenalenones (PhP) with UV maxima between 400 and 500 nm, oxabenzochrysenones (OBC) with UV maxima above 500 nm, phenylbenzoisochromenones (PBIC) with UV maxima below 400 nm and the phenylbenzoisoquinolindones (PBIQ) with UV maxima below 445 nm [1,6,7]. These UV absorbances arise due to the high degree of conjugation present within the structures and readily distinguish these compounds from other secondary metabolites.

As of July 2025, 152 compounds across the wider PhP and related structure classes have been identified from extracts of plants of the Haemodoraceae plant family [1,8].

The bioactivity demonstrated by phenylphenalenone compounds in various anti-microbial assays has motivated further research into isolating and synthesising various structural derivatives [9,10]. This bioactivity arises as a result of phototoxicity and the generation of singlet oxygen radicals which disrupts the biochemical processes of microorganisms [1,11,12]. It is believed that the purpose of biosynthesising these compounds is to provide phytoanticipins and phytoalexins in various species across the Haemodoraceae and Musaceae families [11,13,14,15,16,17,18].

The aim of this study was to conduct a phytochemical profiling study via HPLC-MS of six distinct species of the family Haemodoraceae, four of which have been the subject of prior studies conducted by the MATNAP research group (including *Haemodorum simulans*, *Haemodorum spicatum*, *Haemodorum brevisepalum* and *Macropidia fuliginosa*) and two species (*Haemodorum coccineum* and *Haemodorum distichophyllum*) which represent additional plants of this family recently acquired by the research group [1,8,19,20,21]. Ultimately, the aim was to evaluate how effective chemical profiling using HPLC-MS would be in the rapid identification of previously reported compounds known to occur in this plant family and how effective the methodology would be in identifying the presence of potential new compounds and/or additional structure derivatives.

As part of the protocol adopted by the MATNAP research group for all specimens acquired, an ethanol voucher specimen is retained primarily as a sample repository and for any additional identification purposes. This methodology ensures that an extract is also preserved for future reference. Therefore, ethanolic extracts of specimens that had formerly been studied within the research group served as a verification tool for the efficacy of the profiling methodology by establishing the presence of previously reported compounds within each plant specimen.

A total of thirty different extracts were analysed by HPLC-MS. These voucher specimens included various plants of the Haemodoraceae family obtained from either the same plant collected on different dates and different collection sites, and it also included various plant component parts of the six species (including bulbs, stems etc.). This included the previously studied *H. simulans*, *H. spicatum*, *H. brevisepalum* and *M. fuliginosa* and the two additional species recently acquired, *H. coccineum* and *H. distichophyllum*. The crude ethanolic extracts were also evaluated for anthelmintic activity against exsheathed third-stage larvae (xL3s) of the parasitic nematode *Haemonchus contortus*.

### 1.2. Chemical Profiling Study, Rationale and Methodology

The first step in this process was to compile an internal database (full database available in S1 of the Supporting Information) of PhP, OBC, PBIC and PBIQ compounds [22,23,24,25,26,27,28,29,30,31,32,33,34,35,36,37]. This database consisted of compounds the research group had previously isolated from some of these plants or from the available literature, as established in a previous review conducted by the MATNAP research group published in 2019 [1]. The database details the structure class, the chemical structure, the molecular mass and the characteristic UV absorbances (where available), with compounds reported after the publication of the review also included from the relevant literature [8,9,10,21,38]. Phytochemical profiling was performed by comparing the properties of the individual chromatographic peaks with this database, primarily based on matching the molecular mass via ESI mass spectrometry (both positive and negative ionisation modes employed) and the characteristic UV chromophores. An additional diagnostic tool used was a comparison of observed masses with masses of common PhP and related classes based on common substituents (hydroxy and methoxy groups as well as common glycosidic moieties). This was used to establish the potential structural attributes of compounds which were unable to be positively identified. A summary of the chromatographic (retention time and UV) and mass spectrometry data was also assembled to track common secondary metabolites between the different plant specimens (see Table 1 and Table 2).

High-resolution liquid chromatography electrospray–ionisation mass spectrometry (HRLC(ESI)-MS) was also utilised to analyse six of the thirty extracts of interest to assist with the verification of the identified compounds and to provide further insight into the potential molecular formulae of some of the unknown secondary metabolites.

## 2. Results

### 2.1. Chemical Profiling

**Figure 2:**
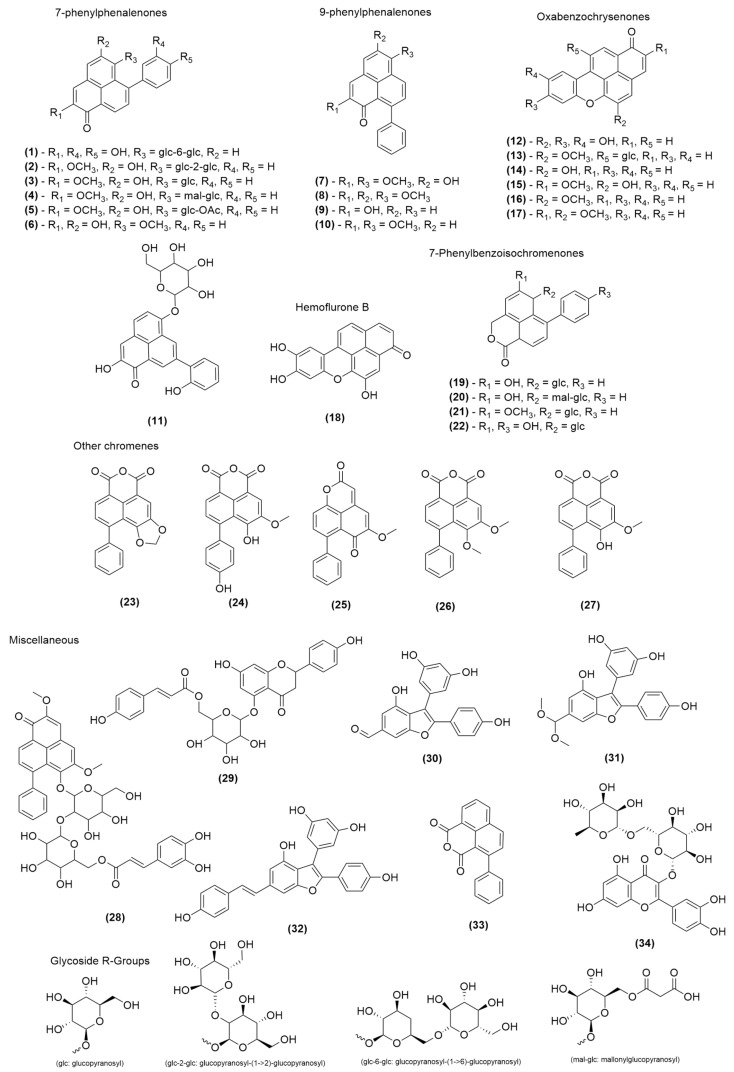
Chemical structures of the compounds identified (**1**–**34**) from the HPLC-MS and HRLC(ESI)-MS chemical profiling of Haemodoraceae plant extracts.

Chemical profiling of the ethanolic extracts of six Australian plants of the family Haemodoraceae (Table 1 and Table 2 and Figure 2) via HPLC-MS resulted in the unequivocal identification of thirty-four previously reported compounds known to occur in this plant family. These compounds were confirmed based on a comparison of UV absorbances and mass spectrometry data from published literature. The positive identification of these compounds verified that the experimental and analytical methodology was reliable and that it could provide a means to identify the presence of potential new structural derivatives. Further high-resolution mass spectrometry (HRLC(ESI)-MS) was conducted on several candidate specimens to provide additional data in support of the identification of the previously reported secondary metabolites and the ability to speculate on the potential for new compounds or isomers of previously identified compounds.

**Table 1:** Summary of the chemical classes and compounds identified across the six Haemodoraceae species chemically profiled via HPLC-MS.

Species	Chemical Class(es) Present	Compounds Confirmed
*H. simulans*	PhP, OBC, PBIC	**(3, 6, 7, 13, 15, 16, 19, 21, 23–27)**
*H. brevisepalum*	PhP, OBC, PBIC	**(1–3, 7, 11, 13–17, 28)**
*H. spicatum*	PhP, OBC, PBIC	**(7, 8, 14–16, 27)**
*M. fuliginosa*	PhP, OBC, PBIC, benzofurans, other naphthalene derivatives, flavonoid glycosides	**(6, 9, 12, 18, 29–34)**
*H. coccineum*	PhP, OBC, PBIC	**(3–5, 10, 22, 23, 25, 27)**
*H. distichophyllum*	PhP, OBC, PBIC, flavonoid glycosides	**(3, 4, 13, 15, 16, 19–21, 25, 26, 34)**

In addition to establishing the presence of previously reported compounds, the primary purpose of the HPLC-MS approach for chemical profiling was to conduct a preliminary investigation of the recently acquired *H. coccineum* and *H. distichophyllum*, as well as to ascertain the presence of any additional compounds which had not been reported in prior studies of the other four Haemodoraceae plants. Analysis of the HPLC-MS data concluded the presence of additional, potentially new compounds and structural derivatives in the bulbs and stems of *H. simulans*, as well as new compounds of interest in *H. coccineum* and *H. distichophyllum*. This was the first phytochemical analysis of *H. distichophyllum*, with only one previous compound being reported from this plant over 50 years ago in the early 1970s [39].

**Table 2:** Identification of the chemical structure classes present in the six plants of the family Haemodoraceae studied (ordered on the basis of HPLC-MS retention time, t_r_).

R*_t_ *(min)	Compound	Structure Class	Species Present (Material Type)	UV (nm)	*m*/*z*	Ref.
9.41	**(1)**	Phenylphenalenone glycoside	*H. brevisepalum* (stems)	222, 368, 434	644	[8]
9.42	**(34)**	Flavonoid glycoside	*M. fuliginosa* (all) *H. distichophyllum* (leaves)	204, 256, 354	610	[20]
9.88	**(2)**	Phenylphenalenone glycoside	*H. brevisepalum* (bulbs)	218, 278, 374, 468	642	[8]
10.66	**(28)**	Phenylphenalenone glycoside	*H. brevisepalum* (bulbs)	220, 278, 334, 470	818	[8]
10.99	**(11)**	Phenylphenalenone glycoside	*H. brevisepalum* (stems)	248, 288, 458	466	[8]
11.01	**(3)**	Phenylphenalenone glycoside	*H. simulans* (bulbs + stems) *H. brevisepalum* (bulbs) *H. coccineum* (bulbs) *H. distichophyllum* (roots)	278, 374, 474	480	[8,21,40,41]
11.05	**(12)**	Oxabenzochrysenone	*M. fuliginosa* (all)	236, 282, 394, 570	318	[20]
11.11	**(4)**	Phenylphenalenone glycoside	*H. coccineum* (bulbs) *H. distichophyllum* (roots)	224, 278, 374, 470	566	[21]
11.15	**(19)**	Phenylbenzoisochromenone glycoside	*H. simulans* (bulbs + stems) *H. distichophyllum* (leaves)	336, 366	454	[21,40,41]
11.31	**(20)**	Phenylbenzoisochromenone glycoside	*H. distichophyllum* (leaves)	260, 334, 364	540	[21]
11.41	**(18)**	Oxabenzochrysenone	*M. fuliginosa* (all)	554	318	[20]
11.65	**(13)**	Oxabenzochrysenone glycoside	*H. simulans* (stems) *H. brevisepalum* (stems) *H. distichophyllum* (flowers)	268, 288, 334, 348, 402, 542	478	[40,41]
11.67	**(5)**	Phenylphenalenone glycoside	*H. coccineum* (bulbs)	246, 270, 336, 464	522	[21]
11.82	**(21)**	Phenylbenzochromenone glycoside	*H. simulans* (bulbs) *H. distichophyllum* (leaves)	254, 346, 394	468	[40,41]
11.99	**(22)**	Phenylbenzoisochromenone glycoside	*H. coccineum* (bulbs)	228, 254, 326, 384	470	[21]
12.77–13.35	**(29)**	Flavonoid glycoside	*M. fuliginosa* (bulbs)	236, 316, 370	580	[20]
13.21	**(30)**	Benzofuran	*M. fuliginosa* (bulbs)	234, 290sh, 322, 364	362/408	[20]
13.21	**(31)**	Benzofuran	*M. fuliginosa* (bulbs)	234, 290sh, 322, 364	362/408	[20]
14.34	**(32)**	Benzofuran	*M. fuliginosa* (bulbs)	224, 292, 368	452	[20]
14.66	**(24)**	Phenylbenzoisochromenone	*H. simulans* (bulbs)	254, 342, 400	336	[40,41]
14.85	**(14)**	Oxabenzochrysenone	*H. brevisepalum* (stems) *H. spicatum* (stems)	234, 312, 520	286	[8]
16.22	**(7)**	Phenylphenalenone	*H. simulans* (bulbs) *H. brevisepalum* (stems) *H. spicatum* (bulbs)	276, 372, 460	332	[40,41]
16.47	**(6)**	Phenylphenalenone	*M. fuliginosa* (bulbs)	250, 280, 374, 476	318	[20]
16.57	**(15)**	Oxabenzochrysenone	*H. simulans* (bulbs + stems) *H. brevisepalum* (stems) *H. spicatum* (stems) *H. distichophyllum* (flowers)	236, 320, 388, 412, 540	316	[8,19,40,41]
16.75	**(23)**	Phenylbenzoisochromenone	*H. simulans* (bulbs)	254, 327, 368, 421	318	[21,40,41]
17.44	**(27)**	Phenylbenzoisochromenone	*H. simulans* (bulbs + stems) *H. spicatum* (bulbs) *H. coccineum* (bulbs)	256, 344, 396	320	[19,21,40,41]
17.55	**(16)**	Oxabenzochrysenone	*H. simulans* (bulbs + stems) *H. spicatum* (bulbs) *H. brevisepalum* (stems) *H. distichophyllum* (flowers)	244, 344, 378, 522	300	[19,40,41]
17.64	**(17)**	Oxabenzochrysenone	*H. brevisepalum* (bulbs)	238, 320, 364, 384, 536	330	[8]
17.86	**(33)**	Naphthalic anhydride	*M. fuliginosa* (bulbs)	246, 344	274	[20]
18.13	**(25)**	Phenylbenzoisochromenone	*H. simulans* (bulbs) *H. coccineum* (bulbs) *H. distichophyllum* (roots)	266, 340, 384	304	[19,40,41]
19.72	**(8)**	Phenylphenalenone	*H. spicatum* (bulbs)	238, 276, 372, 462	346	[19]
19.97	**(9)**	Phenylphenalenone	*M. fuliginosa* (bulbs)	242, 260, 350, 366, 416	272	[20]
20.16	**(10)**	Phenylphenalenone	*H. coccineum* (bulbs)	272, 370, 436	316	[19,21]
20.29	**(26)**	Phenylbenzoisochromenone	*H. simulans* (bulbs) *H. distichophyllum* (roots/bulbs)	258, 346, 394	334	[40,41]

Of the six plants subjected to phytochemical profiling, *H. simulans* as well as the recently acquired, *H. coccineum* and *H. distichophyllum*, yielded results that indicated their suitability for further in-depth studies. The following sections contain a summary of the results from each plant species.

### 2.2. Anti-Microbial and Anthelmintic Activity

Phenylphenalenone compounds and their derivatives have been of interest from a bioactivity perspective primarily due to their observed anti-microbial properties. Significant bioactivity has been observed from the compounds Hemoflurone A **(12)** and B **(18)**—see Table 1—isolated from *M. fuliginosa*, which exhibited greater activity against *Pseudomonas aeruginosa* than ampicillin [20]. Three other 7- and 9-phenylphenalenones isolated from *M. fuliginosa* also inhibited the growth of *Streptococcus pyogenes* [20]. Both *P. aeruginosa* and *S. pyogenes* have shown growing drug resistance against contemporary antibiotic treatments and, therefore the activity demonstrated by these compounds is of significant interest [1,20]. A number of compounds isolated from *H. spicatum* also demonstrated superior inhibition of *P. aeruginosa* compared with ampicillin, including the oxabenzochryenones **(14)**, **(16)** and **(17),** as well as the phenylphenalenone compound **(10)** [1,19]. PhP’s and related compounds have also been investigated for their potential anti-leishmanicidal activity, as well as structurally modified derivatives of 9-PhPs demonstrating potential anti-plasmodial properties against chloroquine resistant malarial strains [42,43].

To date, only limited anti-parasitic assessment of Haemodoraceae compounds has been performed. In this study, all thirty extracts were evaluated for biological activity against larvae (xL3s) of the parasitic nematode *Haemonchus contortus*. Prior bioactivity conducted on pure compounds isolated from *H. brevisepalum* indicated no activity for the seven compounds tested, which were all glycosidic derivatives of phenylphenalenones and phenylbenzoisochromenones—including **(1)**, **(11)**, **(19)**, **(22)** and **(28)**. As such, the antiparasitic activity of aglycone compounds from the wider PhP family remains unknown [8] and warrants exploration.

This current study represents the first anthelmintic assay assessment against this parasite using the crude Haemodoraceae extracts, the results of which are presented in Table 3.

Anthelmintic activity in this assay was defined as (i) a reduction in larval motility of ≥70% and/or (ii) an induction of abnormal phenotypes in >70% of specimens. Partial activity was defined as (i) a larval motility inhibition of >50% to ≤70% and/or (ii) an induction of abnormal phenotypes in >50% to ≤70% worms. Using these criteria, anthelmintic activity was linked to extracts from *H. simulans* stems, *M. fuliginosa* bulbs, and from the roots and bulbs of *H. distichophyllum*, with the root extract of *H. distichophyllum* exhibiting the most pronounced inhibition (≥90%) of *H. contortus* larval motility. Phytochemical analyses of the extracts of *H. simulans* and *H. distichophyllum* are provided in Section 3. Chemical profiles of the extracts from *M. fulginosa* bulbs that displayed activity, are available in the Supporting Information (S12).

## 3. Discussion

### 3.1. Haemodorum Simulans

**Figure 3:**
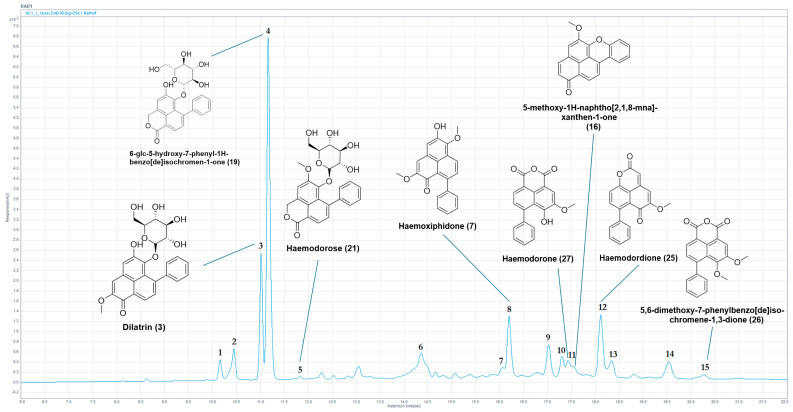
Annotated HPLC chromatogram (254 nm) of the ethanolic crude extract from the *H. simulans* bulbs (2005_01a) showing the identified compounds.

HPLC-MS chemical profiling of *H. simulans* was successful in establishing the presence of all ten compounds previously reported from the bulbs and the stem extracts [40,41]. Chemical profiling of the bulbs (2005_01a) yielded a further six compounds having molecular masses that could not be matched in the database assembled (see S1 in the Supporting Information file), thereby suggesting the presence of potential new structure derivatives. This included possible isomers of the previously reported compounds haemoxiphidone **(7)** and haemodorone **(27)**, as well as a potential compound from the phenylbenzoisoquinolindone class, which would represent the first compound of this class identified within *H. simulans* and the genus *Haemodorum*. The complete chemical profile of one of the three *H. simulans* bulb extracts illustrative of these secondary metabolites is provided in Figure 3 and Table 4.

Previously reported compounds were identified by comparing the experimental data with the literature data from the internal Haemodoraceae secondary metabolite database (see S1 in the Supporting Information file), specifically matching UV-visible chromophores as well as comparison of the mass spectra with known molecular masses. An example of the process adopted is illustrated in the identification of the known phenylphenalenone glycoside dilatrin **(3),** which was identified as a major component of the bulbs of *H. simulans* [40,41], is provided.

The UV spectrum displayed absorbances at 278, 374 and 472 nm, indicating a potential phenylphenalenone-type structure (see Supporting Information S30). The observed masses at *m*/*z* = 481 [M+H]^+^ and *m*/*z* = 319 [M+H-glc]^+^ indicated a molecular weight of 480 amu, and this data was a match for the known phenylphenalenone glucoside dilatrin **(3)**, previously isolated and reported from *H. simulans* and *H. brevisepalum* [8,40,41].

Other chromatographic peaks of note from this specimen included potential new phenylphenalenone glycosides with masses of 460 and 446 amu, with the characteristic chromophores above 400 nm suggesting phenylphenalenone moieties, and similar absorbances and a difference of 14 mass units which may indicate similar structures with the exchange of a methoxy group with a hydroxy group.

**Figure 4:**
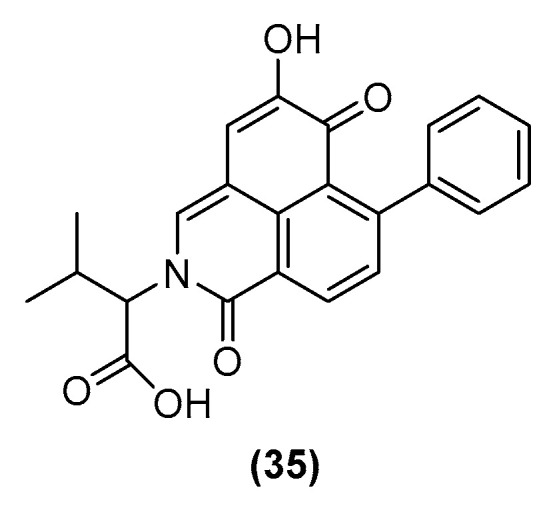
Structure of PBIQ compound **(35)**, [2-(1″-Carboxy-2″-methyl-propyl)-5-hydroxy-7-phenyl-2H-benzo[de]isoquinoline-1,6-dione] isolated from *X. caeruleum*.

The chromatographic peak 6 (tr = 14.36 min) exhibited an odd molecular ion (*m*/*z =* 388 [M−H]^−^, HRLC(ESI)-MS and corresponding *m*/*z* = 390.1330 [M+H]^+^), and chromophores indicative of a conjugated structure, supportive of the PhP-type compound class (λ_max_ = 234, 340, 432 nm). This may represent a phenylbenzoisoquinolindone (PBIQ) compound, and is of equal mass to a known PBIQ compound **(35)** previously isolated from *Xiphidium caeruleum* (Haemodoraceae) (λ_max_ = 236, 265, 323, 428 nm; HRLC(ESI)-MS *m*/*z* = 390.1334 [M+H]^+^) [44], the structure of which is given in Figure 4. The variance in UV absorbances indicates the compound observed in this sample may be a new isomer of **(35)**. The data set for this chromatographic peak has not been previously observed in the phytochemical analysis of *H. simulans*, nor within the genus *Haemodorum*.

Two additional chromatographic peaks yielding potential masses not previously observed (Chromatographic peaks 12/13, t_r_ = 18.12, 18.33 min, ESI-MS *m*/*z* = 305/351 [M+H]^+^ [co-eluting with haemodordione **(25)** (see Supporting Information S52)] and 351 [M+H]^+^) exhibited characteristic chromophores of PBIC (λ_max_ < 400 nm) and PhP (λ_max_ > 400 nm) compounds, respectively.

Potential isomers of haemoxiphidone (λ_max_ = 460 nm) **(7)** and haemodorone (λ_max_ = 393 nm) **(27)** (see Supporting Information S34 and S54, respectively), compounds previously isolated from *H. simulans* bulbs, are present in chromatographic peaks 9 and 10 (t_r_ = 17.03, 17.31 min; λ_max_ = 268, 340, 434 nm and λ_max_ = 266, 346, 410 nm; ESI-MS *m*/*z* = 333 [M+H]^+^ and 321 [M+H]^+^). An additional likely PhP-type compound is present in the chromatographic peak labelled 14 (t_r_ = 19.53 min, λ_max_ = 456 nm, ESI-MS *m*/*z* = 317 [M+H]^+^).

**Figure 5:**
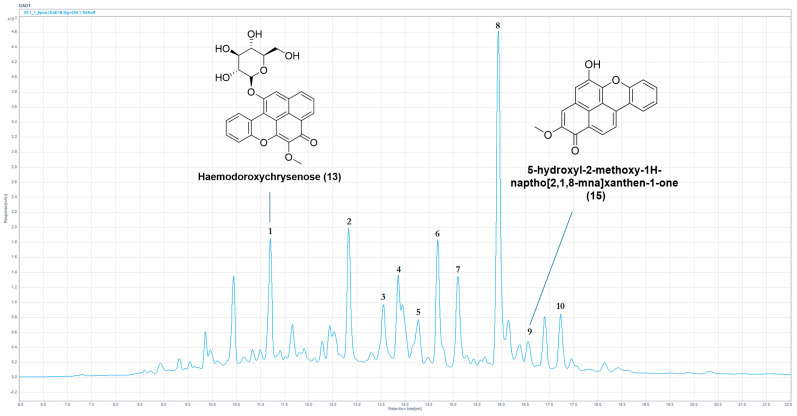
Annotated HPLC chromatogram (254 nm) of the ethanolic crude extract from the *H. simulans* stems (2010_17b) showing the identified compounds.

HPLC-MS chemical profiling of the stems of *H. simulans* (2010_17b) yielded two previously reported compounds, two chromatographic peaks with masses not corresponding to any known PhP-type compound and six potential isomers of previously reported compounds. A full chemical profile of a previously unstudied specimen of *H. simulans* (stems) is provided in Figure 5 and Table 5.

Analysis of 2010_17b permitted the identification of the previously reported compounds haemodoroxychrysenose **(13)** and 5-hydroxy-2-methoxy-1H-naphtho [2,1,8-mna]xanthen-1-one **(15)** (see Supporting Information S40 and S42, respectively). Additional unreported masses were observed for chromatographic peaks 2 and 5 (HRLC(ESI)-MS) *m*/*z* = 337.0339, 351.0495, respectively), both exhibiting chromophores indicative of PhP compounds with a high degree of conjugation (λ_max_ = 230, 306, 342, 496; λ_max_ = 246, 484, respectively). The remaining chromatographic peaks were attributable to potential isomers of known compounds, with possible isomers of haemodorol **(24)** and haemodorone **(27)** observed as chromatographic peaks 3 and 10, with a potential derivative of the latter present as chromatographic peak 6. Chromatographic peaks 4, 7 and 8 may also be isomers of known compounds, an OBC in the case of chromatographic peak 7 and PBICs in the case of chromatographic peaks 4 and 8, owing to their respective chromophores (OBC; λ_max_ > 500 nm, PBIC; λ_max_ < 400 nm).

The phytochemical profiling of *H. simulans* demonstrated the potential for future study of both the bulbs and aerial components of the plant due to the presence of possible new structural derivatives of previously isolated compounds as well as the anthelmintic activity exhibited by an extract of a previously unstudied collection of *H. simulans* stems (2010_17b) (see Section 2.2).

### 3.2. Haemodorum Coccineum

**Figure 6:**
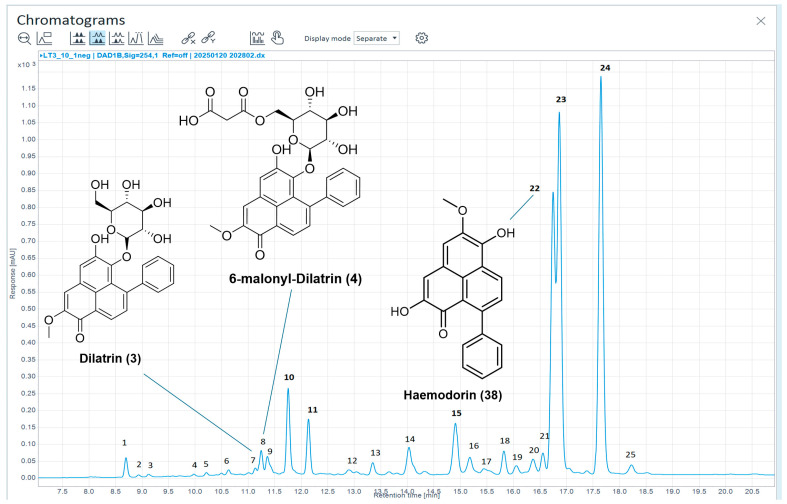
Annotated HPLC chromatogram (254 nm) of ethanolic extract from *H. coccineum* bulbs (2023_01a) showing identified compounds.

HPLC-MS chemical profiling of a recently acquired specimen of *H. coccineum* led to the identification of eight, of a possible sixteen, known compounds which were only recently reported in the literature, primarily as mixtures [21]. Chemical profiling of the bulbs of *H. coccineum* (2023_01a) yielded three previously described compounds, ten chromatographic peaks not corresponding with previously reported masses for PhP-type compounds, one chromatographic peak likely to be a new compound as well as seven potential isomers of previously reported compounds, as shown in Figure 6 and Table 6.

Based on their UV chromophores (λ_max_ = 200, 224, 328; λ_max_ = 218, 328; λ_max_ = 218, 328; λ_max_ = 218, 326; λ_max_ = 220, 330; λ_max_ = 216, 332) chromatographic peaks 1–5 and 10 are unlikely to be PhP-type compounds and may instead be potential flavonoid or other polar secondary metabolites. Both chromatographic peaks 6 and 11 may be previously reported PBIC glycosides or potential isomers of these compounds. Chromatographic peak 12 appears to be related to chromatographic peak 11 due to their similarity in UV profiles, potentially differing in the attached glycosidic groups.

The mass reported for chromatographic peak 9 has not been observed in any PhP-type compound [20], does not correspond to common arrangements of hydroxy-, methoxy- and common glycosidic substitutions and may be either a PhP or conjugated PBIC compound (λ_max_ = 218, 286, 346, 404). Peak 13 is likely to be a new compound with a mass not previously reported from PhP-type compounds, with UV chromophores (λ_max_ = 222, 268, 326, 388) indicating the compound to be a potentially new PBIC type compound.

Chromatographic peaks 14 and 15 may be structurally related, exhibiting similar UV chromophores, with chromatographic peak 15 displaying a previously unreported mass (ESI-MS *m*/*z =* 325 [M+H]^+^) and may represent a new PBIC structural derivative (λ_max_ = 222, 256, 274, 330, 394). Chromatographic peak 16 may be an isomer of a previously reported PBIC compound related to the three chromatographic peaks mentioned prior.

Chromatographic peak 18 may represent the known compound haemodordiol **(36)** previously isolated from *H. spicatum*, however the deviation of observed absorbance wavelengths from the literature values precludes a definitive identification.

**Figure 7:**
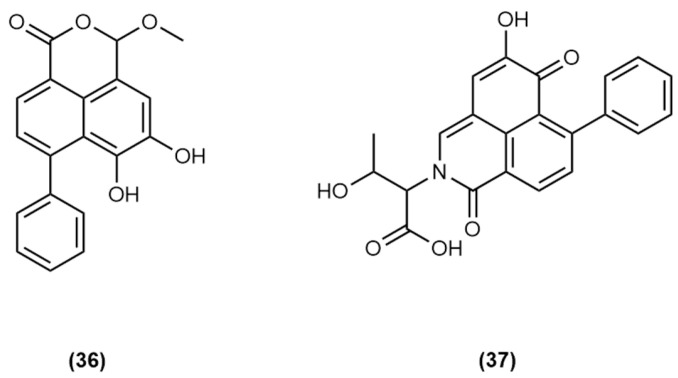
Structures of haemodordiol (5,6-dihydroxy-3-methoxy-7-phenyl-1H,3H-benzo[de]isochromen-1-one) **(36)** and (1″*S*)-2-(1″-Carboxy-2″-hydroxy-propyl)-5-hydroxy-7-phenyl-2H-benzo[de]isoquinoline-1,6-dione **(37)** [7,19].

Chromatographic peak 19 represents another PBIQ compound **(37)** and is similar to the PBIQ compound observed in the extract of the bulbs of *H. simulans* **(35)** with the only structural difference being the exchange of one of the terminal methyl groups on the lactam-affixed side chain with a hydroxyl group. Both **(35)** and **(37)** were isolated from the plant *X. caeruleum* [7,44]. Structures of **(36)** and **(37)** are given in Figure 7.

**Figure 8:**
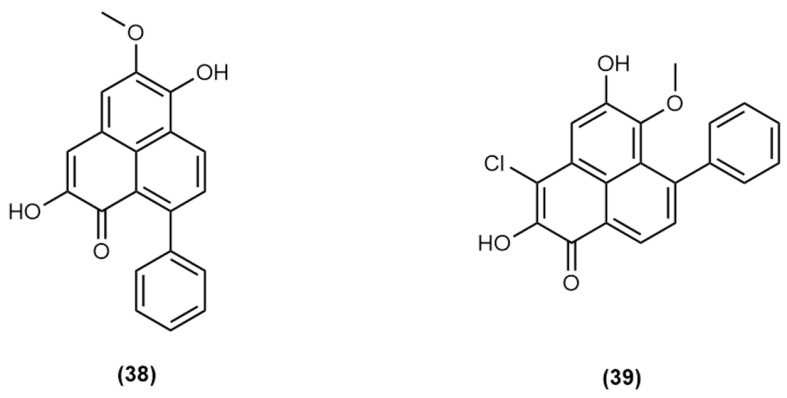
Structures of haemodorin (2,6-dihydroxy-5-methoxy-9-phenyl-1H-phenalen-1-one) **(38)** and 3-chlorofuliginol (3-chloro-2,5-dihydroxy-6-methoxy-7-phenyl-1H-phenalen-1-one) **(39)**.

Chromatographic peak 20 appears to be an isomer of the known compound haemoxiphidone **(7)**. Chromatographic peak 22 has been reported by Carpenelli de Jesus et al. [21] as haemodordioxolane **(23)**; however, the UV absorbances of this compound deviate from those reported by Urban et al. (2013) for haemodordioxolane **(23)** (original reporting of the compound and those expected of a PBIC compound (λ_max_ = 214, 278, 374, 514; literature values λ_max_ = 254, 314sh, 327, 368, 421sh, see Supporting Information S50 for data pertaining to a detection from *H. simulans*) [21,41]. Additionally, the literature HRLC(ESI)-MS data for haemodordioxolane **(23)** from Urban et al. [41] indicates a *m*/*z* of 319.0603 which is in alignment with the molecular formula for haemodordioxolane **(23)** (C_19_H_11_O_5_), whereas the observed *m*/*z* for peak 22 is 319.0956 (C_20_H_15_O_4_). This deviation in mass, in conjunction with the observed UV chromophores (λ_max_ = 218, 278, 374, 514) in fact suggests the presence of the known PhP compound haemodorin **(38),** the structure of which is given in Figure 8 [39], as opposed to haemodordioxolane **(23)** as proposed by Carpenelli de Jesus et al. [21].

Chromatographic peak 23 exhibits the same mass as chromatographic peak 13 and therefore may represent a less polar structural derivative of chromatographic peak 13. Chromatographic peak 24 shares a mass with only one reported PhP-type compound, however this compound is 3-chlorofuliginol **(39)**, which is believed to be a potential artefact arising from the extraction of *M. fuliginosa* with dichloromethane. It is noteworthy that in this current extraction regime, chlorinated solvents were not used. In addition, the ESI-MS data for peak 24 does not feature characteristic isotopic patterns indicative of a chlorine functionalised compound; therefore, it can be concluded that the detected mass corresponds to a previously unreported compound. The structures of **(38)** and **(39)** are given in Figure 8.

Chromatographic peak 25 appears to have some similarities with chromatographic peak 21 with shared UV absorption maxima (λ_max_ = 226, 338, 414; λ_max_ = 224, 268, 338, 386) and a shared ESI-MS *m*/*z* of 336; however, HRLC(ESI)-MS of both peaks shows molecular ions of two different masses ([M+H]^+^ = 337.1062 and 337.0702) indicating a difference in molecular formula between the two compounds (chromatographic peak 21 predicted as C_20_H_17_O_5_, Δ = −2.43 ppm, chromatographic peak 25 predicted as C_19_H_13_O_6_, Δ = −1.41 ppm).

Overall, the number of potential new compounds and isomers of previously reported compounds suggested by this chemical profiling study makes the bulbs of *H. coccineum* a promising candidate for future study.

### 3.3. Haemodorum Distichophyllum

This study represents the first phytochemical profiling of the different components of the rare native Australian plant *H. distichophyllum* and the first report of the chemical composition of the plant for over five decades. The only compound previously reported from this plant is the pigment haemodorin **(38)** reported by Bick and Blackman in 1973 [39]. Chemical profiling, conducted here via HPLC-MS, has established the presence of eleven other compounds for the first time within the species, including the first report of the oxabenzochrysenone and phenylbenzoisochromenone classes being present as well as the common flavonoid glycoside rutin **(34)** (see Supporting Information S61). A summary of detected secondary metabolites identified in *H. distichophyllum* is given in Table 7.

**Figure 9:**
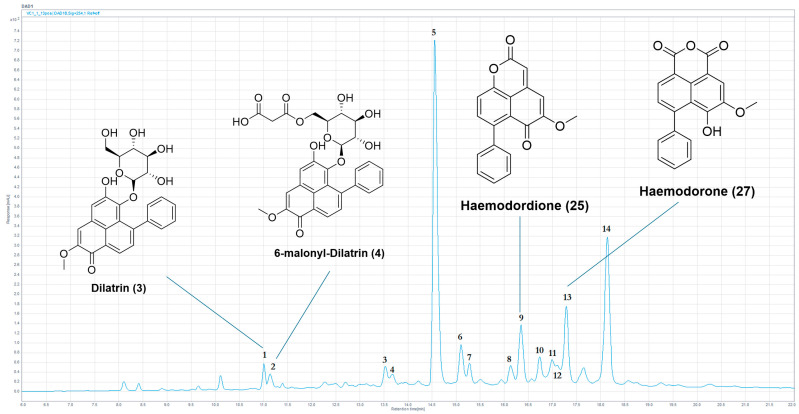
HPLC chromatogram (254 nm) of ethanolic extract from *H. distichophyllum* roots (2021_18c) showing the identified compounds.

Chemical profiling of the roots of *H. distichophyllum* (2021_18c) yielded four compounds previously reported in studies of other Haemodoraceae species, two compounds with masses not corresponding to previously reported compounds of the wider PhP class, and seven compounds which may represent new structural derivatives of known PhP, OBC and PBIC compounds as shown in Figure 9 and Table 8.

Chromatographic peaks 3 and 14 exhibited the same *m*/*z* when analysed via ESI-MS; however, the HRLC(ESI)-MS indicated a difference in the [M+H]^+^ ions of 0.0360 amu which indicates a difference in molecular formula (chromatographic peak 3 predicted as C_20_H_15_O_6_, Δ = −1.42 ppm; chromatographic peak 14 predicted as C_21_H_19_O_5_, Δ = −1.88 ppm). However, both chromatographic peaks exhibit similar UV absorbances to each other as well as the known PBIC compound haemodorone **(27)** (*m*/*z* 320), which suggests structural similarities between all three compounds.

Chromatographic peaks 5 and 12 likely represent OBC type compounds due to the observed absorbances above 500 nm, chromatographic peaks 7, 8 and 11 likely represent PhP-type compounds with absorbances in the 400–500 nm range. Chromatographic peaks 3 and 14 mentioned earlier, in addition to chromatographic peak 10, exhibit absorbances typical of PBIC-type compounds. Chromatographic peak 4, in addition to an indeterminate mass, does not exhibit absorbances indicative of the degree of conjugation present within compounds of the wider PhP family.

Phytochemical profiling of *H. distichophyllum* in conjunction with observed anthelmintic activity from two extracts of the plant (see Section 2.2) highlight the potential of *H. distichophyllum* as a candidate for future study to isolate new secondary metabolites and to establish the source of anthelmintic activity.

The chemical profiling methodology undertaken in this study was assessed based on the proportion of previously reported compounds from the four Haemodoraceae species subjected to prior study which were successfully detected. The percentage of reported compounds detected for *H. simulans*, *H. spicatum*, *M. fuliginosa* and *H. brevisepalum* are given in Table 9.

The HPLC-MS chemical profiling method employed was successful in the identification of a majority of the previously reported secondary metabolites from previously studied plants of the family Haemodoraceae. Due to the small amount of plant material extracted and the specimen extraction regime which employed EtOH as the solvent, as well as the years that the type specimen was stored in the refrigerator, it is unlikely that all secondary metabolites could be identified. Most of these compounds were previously extracted from 3:1 dichloromethane–methanol crude extracts and via other methods reported in the literature, such as the use of chloroform, methanol and acetone extraction as well as pressurised hot water extraction (PHWE), so differences in the compounds extracted is expected [8,19,20,21,40,41]. This methodology was successful in the identification of an average of 64% of the secondary metabolites originally reported from specimens studied by this research group (Table 9) and was able to identify eight of a possible 16 compounds recently reported from *H. coccineum* [21]. The methodology therefore offers a rapid and robust procedure for the chemical profiling of several species of the Haemodoraceae family and can be readily adapted for the profiling of other families or genera.

## 4. Materials and Methods

### 4.1. Plant Material

Plant material was collected and held in accordance with a Victorian Government Department of Environment, Land, Water and Planning permit. Various collections of *H. simulans*, *H. spicatum*, *H. brevisepalum*, *H. coccineum*, *H. distichophyllum* and *M. fuliginosa* were obtained from private and commercial sources as well as via a Materials Transfer Agreement (MTA) with the Royal Botanic Gardens Victoria, as detailed in Table 10.

### 4.2. Extraction

Voucher specimens were prepared by taking approximately 2 g of plant material from each specimen in the collection and extracting it with analytical grade ethanol (20 mL). In most instances, these voucher specimens had been stored in the refrigerator for many years until this study was executed; therefore, the extraction periods for the studied specimens varies from approximately 4 weeks up to 20 years. Crude extracts of these voucher specimens were then taken and decanted and dried under reduced pressure using a rotary evaporator and transferred to pre-weighed vials using HPLC grade methanol. Masses of extracts and percentage yields can be found in S122 of the Supporting Information. The methanol was evaporated on a heating block at 35 °C under a stream of nitrogen and each extract was weighed. Samples of each specimen extract were prepared at concentrations of 2.5 mg/mL in hyper-grade methanol in preparation for HPLC-MS/HRLC(ESI)-MS analyses and at 50 mg/mL in DMSO for the anthelmintic assay evaluation.

### 4.3. Chemical Profiling

Chemical profiling was carried out on all extracts employing HPLC-MS. Aliquots of each extract at a concentration of 2.5 mg/mL were filtered through a 0.45-micron PTFE membrane filter into 2 mL HPLC vials. High-performance liquid chromatography (HPLC) was performed using an Agilent 1200 series solvent delivery system equipped with a 4.6 mm × 150 mm Agilent ZORBAX Eclipse Plus C_18_ column (Agilent Technologies, Santa Clara, CA, USA), and a multi-channel diode array detector (DAD) equipped with a deuterium lamp with detection between 190 and 620 nm. Mass spectra were obtained using an Agilent 6410 Triple Quad HPLC-MS system (Agilent Technologies, Santa Clara, CA, USA) with analyses conducted in both the positive and negative electrospray ionisation modes, utilising a N_2_ flow of 10 L/min, 300 °C drying gas temperature and 4000 V capillary voltage.

The HPLC elution scheme utilised a gradient of milli-Q water and HPLC-MS-grade CH_3_CN with the method provided in Table 11.

### 4.4. High-Resolution Liquid Chromatography–Electrospray Mass Spectrometry (HRLC(ESI)-MS)

High resolution mass spectrometry was performed using the same reversed-phased column and HPLC elution gradient indicated in Table 11, as detailed in Section 4.3, utilising a Dionex UltiMate 3000 UHPLC system (Thermo Fisher Scientific™, Waltham, MA, USA) with a binary pump, autosampler and temperature-controlled column compartment, coupled with a QExactive (QE) Plus Quadropole-Orbitrap^TM^ mass spectrometer. The spectrometer was set at FT positive mode over a mass range 100–1000 amu with resolution set at 35,000; using nitrogen as the sheath, auxiliary and sweep gas with flow rates of 28, 15 and 4 L/min, respectively. Spray voltage was set at 3600 V (positive) and calibration was undertaken with Pierce^®^ LTQ Velos ESI Positive Ion Calibration Solution (Thermo Fisher Scientific™, Waltham, MA, USA). Data acquisition was performed with Thermo Xcalibur V. 2.1 (Thermo Fisher Scientific™, Waltham, MA, USA) and analysed with the Thermo Xcalibur™ Qual Browser v.2.3.26 and FreeStyle™ 1.8 SP2 QF1 (Thermo Fisher Scientific™, Waltham, MA, USA) software packages [45].

### 4.5. Nematode Assay

*Haemonchus contortus* (Haecon-5 strain) was produced in Merino sheep (3 months of age; Victoria, Australia; University of Melbourne animal ethics permit no. 23983). Third-stage larvae (L3s) of *H. contortus* were cultured from eggs in faecal matter. Then, L3s were exsheathed using 0.15% *v*/*v* of sodium hypochlorite (NaClO) at 37 °C for 20 min, followed by five washes in 50 mL sterile physiological saline by centrifugation at 500× *g* for 5 min at room temperature (22–24 °C). Extracts were provided at an approximate concentration of 50 mg/mL in dimethyl sulfoxide (DMSO) and prepared at the final concentration of 0.5 mg/mL in lysogeny broth (LB) supplemented with 100 U/mL penicillin, 100 µg/mL streptomycin and 0.25 µg/mL amphotericin B (Fungizone^®^, Thermo Fisher Scientific™, Waltham, MA, USA) for screening. This supplemented LB + 0.25% DMSO was used as a negative control, with commercial anthelmintic compounds monepantel (Zolvix™; Elanco, Sydney, NSW, Australia) and moxidectin (Cydectin^®^; Virbac, Carros, France) serving as positive controls.

Negative and positive controls and prepared extract solutions were applied to flat-bottom 96-well microtitre plates to which xL3s (~300 larvae/well) were dispensed in a final volume of 100 µL and incubated for 168 h at 38 °C and 10% CO_2_ at >90% humidity. Following incubation, worm activity was measured using a WMicroTracker ONE (Phylumtech, Sunchales, Santa Fe, Argentina). Over a period of 15 min, interference of an infrared beam in individual wells was recorded as an ‘activity count’. The activity counts measured were normalised against the positive and negative controls using the program Prism (v.10.4.2 GraphPad Software, San Diego, CA, USA), to remove plate-to-plate variation. An extract was deemed as having activity if it reduced xL3s motility by ≥70% and/or inhibited larval development after 168 h of incubation. Non-wildtype phenotypes were recorded microscopically at a 200-times magnification [46]. Worms were also examined for possible developmental alterations following staining with Lugol (Sigma-Aldrich, St. Louis, MO, USA), as described previously [46].

## 5. Conclusions

Chemical profiling of thirty extracts of six Australian plants of the family Haemodoraceae via HPLC-MS and reinforced by HRLC(ESI)-MS identified the presence, on average, of 64% of the previously reported secondary metabolites, confirming that this was a successful diagnostic approach to rapidly conduct phytochemical profiling of the plant species and is effective for the preliminary investigation of potential candidate species for a more in depth study. As a result, *H. coccineum* and *H. distichophyllum*, which have not been investigated extensively, have been identified as promising candidates for future larger-scale studies, employing traditional extraction and isolation methods. Additionally, the presence of potential new structural derivatives in extracts of *H. simulans*, warrants a re-investigation of the plant, including further analysis of the phytochemistry of the aerial components. Anthelmintic assays revealed significant activity in extracts from *H. simulans* stems and from *H. distichophyllum*, highlighting the need for renewed investigation and supporting the latter’s potential as a promising candidate for further research. Furthermore, the activity demonstrated in one of the *M. fuliginosa* bulb extracts necessitates further investigation into potential anthelmintic properties of individual compound(s) present within that extract.

## Figures and Tables

**Table 3:** Biological activity of ethanolic extracts of six Australian plants of the family Haemodoraceae on larvae of *H. contortus* in vitro.

Species	Material Type (Source)	Motility Reduction, 168 h (%)	Abnormal Phenotype (%)
*H. simulans*	Bulbs (2005_01a)	64.0 ± 13.2 = Partial	Skinny (10)
	Stems (2005_01b)	7.9 ± 18.4	Skinny (10)
	Bulbs (2007_01a)	45.8 ± 11.8	Skinny (30)
	Stems (2007_01b)	57.5 ± 5.5 = Partial	Skinny (10)
	Bulbs (2010_17a)	39.8 ± 8.9	Skinny (30)
	Stems (2010_17b)	73.7 ± 10.6 = Active *	Skinny (50)
			
*H. brevisepalum*	Bulbs (2010_19a)	−1.5 ± 7.3	Skinny (3)
	Stems (2010_19b)	34.7 ± 7.5	Skinny (17)
			
*H. spicatum*	Bulbs (2010_20a)	12.0 ± 7.5	-
	Stems (2010_20b)	9.5 ± 22.7	-
			
*M. fuliginosa*	Bulbs (2011_01a)	84.1 ± 6.6 = Active *	nca
	Stems (2011_01b)	14.1 ± 4.8	nca
	Bulbs (2011_02a)	64.2 ± 8.4 = Partial	nca
	Stems (2011_02b)	42.6 ± 6.0	Skinny (10)
	Bulbs (2012_01a)	40.6 ± 13.9	nca
	Stems (2012_01b)	18.7 ± 6.7	nca
	Flowers (2012_05a)	63.4 ± 9.1	nca
	Stems/leaves (2012_05b)	31.4 ± 5.9	Skinny (7)
	Stems (2012_05c)	31.0 ± 17.0	Skinny (30)
	Flowers (2013_02)	55.0 ± 8.0 = Partial	nca
			
*H. coccineum*	Leaves/stems (2021_17a)	18.4 ± 15.0	Skinny (10)
	Roots (2021_17b)	50 ± 12.2 = Partial	Skinny (50)
	Leaves/bulbs (2022_08)	40.9 ± 17.0 = Partial	Skinny (50)
	Bulbs (2023_01a)	44.3 ± 17.1	nca
	Stems (2023_01b)	10.0 ± 8.7	Skinny (10)
			
*H. distichophyllum*	Leaves (2021_18a)	62.1 ± 1.9 = Partial	Skinny (50)
	Flowers/seeds (2021_18b)	12.0 ± 15.0	Skinny (20)
	Roots (2021_18c)	93.3 ± 4.1 = Active *	Skinny (70)
	Leaves (2022_07a)	52.8 ± 3.4 = Partial	Skinny (40)
	Roots/bulbs (2022_07b)	45.3 ± 9.9	Skinny (80)
			
Negative control	DMSO	−15.1 ± 11.8	-
Positive controls	Moxidectin	92.8 ± 3.6 = Active *	-
	Monepantel	100.0 ± 2.5 = Active *	Coiled (100)

nca = no consistent alteration; ≥70% reduction in motility *.

**Table 4:** HPLC-MS data of the *H. simulans* bulb ethanolic extract (2005_01a).

Peak	T_r_ (min)	UV (nm)	(*m*/*z*)	HRLC(ESI)-MS ([M+H]^+^)	Compound Identification
1	10.15	240, 322, 436	460	461.1812	New mass
2	10.35	240, 324, 442	446/470	447.1656	New mass or new isomer
3	11.01	278, 374, 472	480	481.1485 *	**(3)**
4	11.15	214, 260, 336, 366	454	455.1328 *	**(19)**
5	11.83	254, 348, 380	468	469.1490 *	**(21)**
6	14.36	234, 320, 432	389	390.1330	Potential PBIQ
7	16.06	250, 350, 394	420	421.1275	New mass
8	16.21	276, 372, 460	332	333.1116 *	**(7)**
9	17.03	268, 340, 434	332	333.1115	Potential isomer of **(7)**
10	17.31	266, 346, 410	320	321.1119	PBIC
11	17.44	256, 344, 396	320	321.0756 *	**(27)**
11a	17.54	234, 322, 522	300	301.0857 *	**(16)**
12	18.12	266, 340, 384	304/350	305.0804 */351.1223	**(25)**/New mass
13	18.33	266, 346, 416	350	351.1224	New mass
14	19.53	242, 262, 298, 366, 456	316	317.1167	PhP
15	20.28	258, 344, 394	334	335.0912 *	**(26)**

* Recorded HRLC(ESI)-MS [M+H]^+^ < ±10 ppm from the literature/calculated value.

**Table 5:** HPLC-MS data of *H. simulans* stem ethanolic extract (2010_17b).

Peak	T_r_ (min)	UV (nm)	(*m*/*z*)	HRLC(ESI)-MS ([M+H]^+^)	Compound Identification
1	11.67	238, 268, 332, 404, 548	478	479.1329 *	**(13)**
2	12.84	230, 306, 342, 496	336	337.0339	New mass (HRLC(ESI)-MS)
3	13.56	252, 346, 420	322	323.0548	New isomer—potentially of **(24)**
4	13.86	268, 338, 388	308	Not detected	Potential new compound/isomer
5	14.29	246, 484	350	351.0495	New mass
6	14.69	260, 330, 348, 416	336	337.0703	Possible derivative of **(27)**
7	15.10	264, 338, 386, 550	302/322	303.0648/323.0909	OBC
8	15.94	222, 268, 346, 412	306	307.0596	PhP/PBIC
9	16.57	242, 270, 372, 412, 540	316	317.0805 *	**(15)**
10	17.23	266, 346, 410	320	321.0755	Potential isomer of **(27)**

* Recorded HRESI-MS [M+H]^+^ < ±10 ppm from the literature/calculated value.

**Table 6:** HPLC-MS data of ethanolic extract of *H. coccineum* bulbs (2023_01a).

Peak	T_r_ (min)	UV (nm)	*m*/*z*	HRLC(ESI)-MS [M+H]^+^	Compound Identification
1	8.69	200, 224, 328	594	Not detected	New mass—unlikely to be PhP-type
2	8.94	218, 328	386	387.1278	New mass—unlikely to be PhP-type
3	9.13	218, 328	386	387.1277	New mass—unlikely to be PhP-type
4	9.99	218, 326	662	Not detected	New mass—unlikely to be PhP-type
5	10.21	220, 330	754	Not detected	New mass—unlikely to be PhP-type
6	10.63	220, 328, 380sh	556	557.1281	Potential isomer of a known PBIC glycoside
7	11.15	220, 280, 374, 474	480	481.1487 *	**(3)**
8	11.25	218, 278, 374, 468	566	567.1486 *	**(4)**
9	11.37	218, 286, 346, 404	462	463.1603	New mass
10	11.75	216, 332	676	677.1848	New mass—unlikely to be PhP-type
11	12.14	222, 254, 326, 384	556	557.1280	Potential isomer of a known PBIC glycoside
12	12.90	222, 324, 384	512	513.1357	New mass—similar UV to peak 11
13	13.35	222, 268, 326, 388	338	339.0856	New mass—PBIC
14	14.04	224, 398	306	307.0596	PBIC
15	14.91	222, 256, 274, 330, 394	324	325.0701	New mass—PBIC
16	15.18	222, 282, 378	322	323.0545	No matching UVs for mass
17	-	-	-	-	Below limit of detection for UV and MS
18	15.82	222, 268, 338, 386	322	323.0908 *	PBIC (potentially **(36)**)
19	16.06	224, 286, 348, 406	391	392.1120	Potential PBIQ **(37)**
20	16.37	224, 296, 372, 422	332	333.1114	PhP
21	16.56	224, 268, 338, 386	336	337.1062	PBIC
22	16.75	214, 278, 374, 514	318	319.0956 *	**(38)**
23	16.86	220, 256, 278, 332, 400	338	339.0856	New mass—PBIC/PhP
24	17.65	218, 252, 282, 332, 386	352	353.1013	PBIC—potentially new compound
25	18.23	226, 338, 414	336	337.0702	PBIC/PhP

* Recorded HRLC(ESI)-MS [M+H]^+^ < ±10 ppm from the literature/calculated value.

**Table 7:** Chemical classes and compounds concluded from HPLC-MS profiling of *H. distichophyllum*.

Chemical Class	No. of Compounds	Compounds Confirmed
Phenylphenalenone	2	**(3)(4)**
Oxabenzochrysenone	3	**(13)(15)(16)**
Phenylbenzoisochromenone	5	**(19–21)(25)(26)**
Other (flavonoid)	1	**(34)**

**Table 8:** Chemical classes and compounds concluded from HPLC-MS profiling of *H. distichophyllum*.

Peak	T_r_ (min)	UV (nm)	(*m*/*z*)	HRLC(ESI)-MS ([M+H]^+^)	Compound Identification
1	11.01	230, 278, 374, 474	480	481.1483 *	**(3)**
2	11.12	234, 276, 374, 470	566	567.1485 *	**(4)**
3	13.53	260, 336, 384	350	351.0858	New mass—PBIC
4	13.68	268, 332	564/896	-	None
5	14.56	238, 320, 366, 384, 544	316	317.0799	OBC
6	15.11	262, 322, 386, 550	322	323.0908	PBIC/OBC
7	15.28	244, 296, 340sh, 380sh, 460sh	302	303.1011	PhP
8	16.14	244, 292, 438	316	317.0806	PhP
9	16.35	244, 388	304	305.0804 *	**(25)**
10	16.74	256, 374	336	337.1066	PBIC
11	16.99	242, 268, 312, 342, 436	288/334/446	335.0908/289.0490	PhP
12	17.06	246, 270, 446, 512, 550, 590	334/408/716	335.0909	OBC
13	17.29	266, 340, 386	320	321.0755	**(27)**
14	18.14	266, 340, 384	350	351.1218	New mass—PBIC similar UV chromophores to **(27)**

* Recorded HRLC(ESI)-MS [M+H]^+^ < ±10 ppm from the literature/calculated value.

**Table 9:** Percentages of previously reported secondary metabolites successfully identified via the HPLC-MS chemical profiling approach from Haemodoraceae species subjected to prior studies by the MATNAP research group.

Species	No. of Compounds Identified *	No. of Compounds Reported	% of Compounds Identified
*H. simulans*	10	10	100%
*H. brevisepalum*	11	30	37%
*H. spicatum*	5	11	45%
*M. fuliginosa*	10	13	77%
			64% (average % of compounds identified)

* Some secondary metabolites present in multiple species and therefore are counted multiple times.

**Table 10:** Collection details of plant material.

Species	Voucher Codes	Collection Location/s	Identified/Collected by
*Haemodorum simulans*	2005_01, 2007_01, 2010_17	Arrowsmith River region, Eneabba, Western Australia	Mr Allan Tinker, 2005–2010 (Plant licence: SW008335)
*Haemodorum brevisepalum*	2010_19	Arrowsmith River region, Eneabba, Western Australia	Mr Allan Tinker, 2010 (Plant licence: SW008335)
*Haemodorum spicatum*	2010_20	Arrowsmith River region, Eneabba, Western Australia	Mr Allan Tinker, 2010 (Plant licence: SW008335)
*Macropidia fuliginosa*	2011_01, 2011_02, 2012_01, 2012_05, 2013_02	2011_01, 2012_01, 2012_05—Kuranga Native Australian Nursery, Mt Evelyn, Victoria 2011_02, 2013_02—Arrowsmith River region, Eneabba, Western Australia	2011_01, 2012_01, 2012_05—Dr Robert Brkljača (purchased), 2011–12 2011_02, 2013_02—Mr Allan Tinker, 2011–2013 (Plant licence: SW008335)
*Haemodorum coccineum*	2021_17, 2022_08, 2023_01	2021_17, 2022_08—Cranbourne Botanical Gardens, Cranbourne, Victoria 2023_01—Territory Native Plants, Darwin, Northern Territory	2021_17, 2022_08—Ms Bronwyn Swartz, Ms Deepika Dugan, Ms Mandy Thomson, 2021–2022 (MTA—Royal Botantical Gardens Victoria) 2023_01—Ms Amber Wildi, (purchased) 2023
*Haemodorum distichophyllum*	2021_18, 2022_07	Cranbourne Botanical Gardens, Cranbourne, Victoria	2021_18, 2022_07—Mrs Bronwyn Swartz, Ms Deepika Dugan, Ms Mandy Thomson, 2021–2022 (MTA—Royal Botanical Gardens Victoria)

**Table 11:** HPLC-MS elution gradient method.

Time (min)	%H_2_O	%CH_3_CN
0–2	90	10
14–24	25	75
26–30	0	100
32–40	90	10

## Data Availability

The original contributions presented in this study are included in the article/Supplementary Materials. Further inquiries can be directed to the corresponding author.

## Supplementary Materials

The following Supporting Information can be downloaded at: https://www.mdpi.com/article/10.3390/molecules30204044/s1, S1. The database of previously reported phenylphenalenone and related compounds up to July 2025; S2–S27. UV chromatograms (254 nm) of extracts acquired via HPLC-MS; S28–S61. Profiling data (Extracted UV spectra and ESI-MS) for compounds **(1–34)**; S62–S121. +HRLC(ESI)-MS data for featured extracts; S122. Extract masses and yields from voucher samples.
