# Supplementary material for: Targeted Chemical Profiling and Dereplication of Australian Plants of the Family Haemodoraceae Using a Combined HPLC-MS and HRLC(ESI)-MS Approach
Source: Molecules. 2025 Oct 10;30(20):4044. doi: 10.3390/molecules30204044 (PMC12566560; doi:10.3390/molecules30204044)
Supplement: Supplementary file 1 [file molecules-30-04044-s001.zip › molecules-3866597-supplementary.pdf]

Targeted chemical profiling of Australian plants of the family Haemodoraceae using a combined HPLC-MS and HRLC(ESI)-MS approach

Liam Thompson, Valerie Chow, Shan Chen, Priyanka Reddy, Robert Brkljača, Colin Rix, Joseph J. Byrne, Aya C. Taki, Robin B. Gasser, and Sylvia Urban

Supporting information:

S1. Database of known phenylphenalenone and related compounds from Haemodoraceae and other species as of July 2025 6

S2. Annotated expansion of UV chromatogram (254 nm) (t_R = 6-24 min) of ethanolic extract of *Haemodorum simulans* bulbs (2005_01a) 34

S3. Annotated expansion of UV chromatogram (254 nm) (t_R = 6-24 min) of ethanolic extract of Haemodorum simulans stems (2005_01b) 34

S4. Annotated expansion of UV chromatogram (254 nm) (t_R = 6-24 min) of ethanolic extract of *Haemodorum simulans* bulbs (2007_01a) 35

S5. Annotated expansion of UV chromatogram (254 nm) (t_R = 6-24 min) of ethanolic extract of *Haemodorum simulans* stems (2007_01b) 35

S6. Annotated expansion of UV chromatogram (254 nm) (t_R = 6-24 min) of ethanolic extract of *Haemodorum simulans* bulbs (2010_17a) 36

S7. Annotated expansion of UV chromatogram (254 nm) (t_R = 6-24 min) of ethanolic extract of *Haemodorum simulans* stems (2010_17b) 36

S8. Annotated expansion of UV chromatogram (254 nm) (t_R = 6-24 min) of ethanolic extract of *Haemodorum brevisepalum* bulbs (2010_19a) 37

S9. Annotated expansion of UV chromatogram (254 nm) (t_R = 6-24 min) of ethanolic extract of *Haemodorum brevisepalum* stems (2010_19b) 37

S10. Annotated expansion of UV chromatogram (254 nm) (t_R = 6-24 min) of ethanolic extract of *Haemodorum spicatum* bulbs (2010_20a) 38

S11. Annotated expansion of UV chromatogram (254 nm) (t_R = 6-24 min) of ethanolic extract of *Haemodorum spicatum* stems (2010_20b) 38

S12. Annotated expansion of UV chromatogram (254 nm) (t_R = 6-24 min) of ethanolic extract of *Macropidia fuliginosa* bulbs (2011_01a) 39

S13. Annotated expansion of UV chromatogram (254 nm) (t_R = 6-24 min) of ethanolic extract of *Macropidia fuliginosa* bulbs (2011_02a) 39

S14. Annotated expansion of UV chromatogram (254 nm) (t_R = 6-24 min) of ethanolic extract of *Macropidia fuliginosa* bulbs (2012_01a) 40

S15. Annotated composite expansion of UV chromatograms (254 nm) (t_R = 6-24 min) of ethanolic extracts of *Macropidia fuliginosa* stems/leaves (2011_01b, 2011_02b, 2012_01b, 2012_05b) 40

S16. Annotated expansion of UV chromatograms (254 nm) (t_R = 6-24 min) of ethanolic extract of *Macropidia fuliginosa* stems (2012_05c) 41

S17. Annotated composite expansion of UV chromatograms (254 nm) (t_R = 6-24 min) of ethanolic extracts of *Macropidia fuliginosa* flowers (2012_05a, 2013_02) 41

S18. Annotated expansion of UV chromatogram (254 nm) (t_R = 6-24 min) of ethanolic extract of *Haemodorum coccineum* leaves/stems (2021_17a) 42

S19. Annotated expansion of UV chromatogram (254 nm) (t_R = 6-24 min) of ethanolic extract of *Haemodorum coccineum* roots (2021_17b) 42

S20. Annotated expansion of UV chromatogram (254 nm) (t_R = 6-24 min) of ethanolic extract of *Haemodorum coccineum* leaves/bulbs (2022_08) 43

S21. Annotated expansion of UV chromatogram (254 nm) (t_R = 6-24 min) of ethanolic extract of *Haemodorum coccineum* bulbs (2023_01a) 43

S22. Annotated expansion of UV chromatogram (254 nm) (t_R = 6-24 min) of ethanolic extract of *Haemodorum coccineum* stems (2023_01b) 44

S23. Annotated expansion of UV chromatogram (254 nm) (t_R = 6-24 min) of ethanolic extract of *Haemodorum distichophyllum* leaves (2021_18a) 44

S24. Annotated expansion of UV chromatogram (254 nm) (t_R = 6-24 min) of ethanolic extract of *Haemodorum distichophyllum* flowers/seeds (2021_18b) 45

S25. Annotated expansion of UV chromatogram (254 nm) (t_R = 6-24 min) of ethanolic extract of *Haemodorum distichophyllum* roots (2021_18c) 45

S26. Annotated expansion of UV chromatogram (254 nm) (t_R = 6-24 min) of ethanolic extract of *Haemodorum distichophyllum* leaves (2022_07a) 46

S27. Annotated expansion of UV chromatogram (254 nm) (t_R = 6-24 min) of ethanolic extract of *Haemodorum distichophyllum* roots/bulbs (2022_07b) 46

S28. Profiling data (UV chromatogram and ESI-MS) of **(1)** 7-(3,4-dihydroxyphenyl)-5-((3,4,5-trihydroxy-6-(((3,4,5-trihydroxy-6-(hydroxymethyl)tetrahydro-pyran-2-yl)oxy)methyl)-tetrahydro-pyran-2-yl)oxy)-2-hydroxy-phenalen-1-one 47

S29. Profiling data (UV chromatogram and ESI-MS) of **(2)** 6-((4,5-dihydroxy-6-(hydroxymethyl)-3-((3,4,5-trihydroxy-6-(hydroxymethyl)tetrahydro-pyran-2-yl)oxy)tetrahydro-pyran-2-yl)oxy)-5-hydroxy-2-methoxy-7-phenyl-1H-phenalen-1-one 48

S30. Profiling data (UV chromatogram and ESI-MS) of **(3)** Dilatrin 49

S31. Profiling data (UV chromatogram and ESI-MS) of **(4)** 6-O-[(6″-O-malonyl)-β-D-glucopyranosyl]-5-hydroxy-2-methoxy-7-phenyl-1H-phenalen-1-one 50

S32. Profiling data (UV chromatogram and ESI-MS) of **(5)** 6-(β-D-glucopyranosyloxy)-5-hydroxy-2-methoxy-7-phenyl-1H-phenalen-1-one 51

S33. Profiling data (UV chromatogram and ESI-MS) of **(6)** Fuliginol 52

S34. Profiling data (UV chromatogram and ESI-MS) of **(7)** Haemoxiphidone 53

S35. Profiling data (UV chromatogram and ESI-MS) of **(8)** 2,5,6-trimethoxy-9-phenyl-1H-phenalen-1-one 54
S36. Profiling data (UV chromatogram and ESI-MS) of **(9)** Anigorufone 55
S37. Profiling data (UV chromatogram and ESI-MS) of **(10)** 2,6-dimethoxy-9-phenyl-1H-phenalen-1-one 56
S38. Profiling data (UV chromatogram and ESI-MS) of **(11)** 2-hydroxy-8-(2-hydroxyphenyl)-6-((3,4,5-trihydroxy-6-(hydroxymethyl)tetrahydro-pyran-2-yl)oxy)-phenalenone 57
S39. Profiling data (UV chromatogram and ESI-MS) of **(12)** Hemoflurone A 58
S40. Profiling data (UV chromatogram and ESI-MS) of **(13)** Haemodoroxychrysenose 59
S41. Profiling data (UV chromatogram and ESI-MS) of **(14)** 5-hydroxy-1H-naphtho[2,1,8-mna]xanthen-1-one 60
S42. Profiling data (UV chromatogram and ESI-MS) of **(15)** 5-hydroxy-2-methoxy-1H-naphtho[2,1,8-mna]xanthen-1-one 61
S43. Profiling data (UV chromatogram and ESI-MS) of **(16)** 5-methoxy-1H-naphtho[2,1,8-mna]xanthen-1-one 62
S44. Profiling data (UV chromatogram and ESI-MS) of **(17)** 2,5-dimethoxy-1H-naphtho[2,1,8-mna]xanthen-1-one 63
S45. Profiling data (UV chromatogram and ESI-MS) of **(18)** Hemoflurone B 64
S46. Profiling data (UV chromatogram and ESI-MS) of **(19)** 6-(β-D-glycopyranosyl)-5-hydroxy-7-phenyl-1H-benzo[de]isochromen-1-one 65
S47. Profiling data (UV chromatogram and ESI-MS) of **(20)** 6-O-[(6″-O-malonyl)-β-D-glucopyranosyl]-5-hydroxy-7-phenyl-3H-benzo[de]isochromen-1-one 66
S48. Profiling data (UV chromatogram and ESI-MS) of **(21)** Haemodorose 67
S49. Profiling data (UV chromatogram and ESI-MS) of **(22)** 6-(β-D-glycopyranosyl)-5-hydroxy-7(4′-hydroxyphenyl)-1H,3H-benzo[de]isochromen-1-one 68
S50. Profiling data (UV chromatogram and ESI-MS) of **(23)** Haemodordioxolane 69
S51. Profiling data (UV chromatogram and ESI-MS) of **(24)** Haemodorol 70
S52. Profiling data (UV chromatogram and ESI-MS) of **(25)** Haemodordione 71
S53. Profiling data (UV chromatogram and ESI-MS) of **(26)** 5,6-dimethoxy-7-phenylbenzo[de]isochromene-1,3-dione 72
S54. Profiling data (UV chromatogram and ESI-MS) of **(27)** Haemodorone 73
S55. Profiling data (UV chromatogram and ESI-MS) of **(28)** (6-((2-((2,5-dimethoxy-1-oxo-7-phenyl-phenalen-6-yl)oxy)-4,5-dihydroxy-6-(hydroxymethyl)tetrahydro-pyran-3-yl)oxy)-3,4,5-trihydroxytetrahydro-pyran-2-yl)methyl (E)-3-(3,4-dihydroxyphenyl)acrylate 74
S56. Profiling data (UV chromatogram and ESI-MS) of **(29)** P-hydroxycinnamate of salipurposide 75

S57. Profiling data (UV chromatogram and ESI-MS) of **(30)** Fulginosin A 76
S58. Profiling data (UV chromatogram and ESI-MS) of **(31)** Fulginosin B 77
S59. Profiling data (UV chromatogram and ESI-MS) of **(32)** Angiopressin A 78
S60. Profiling data (UV chromatogram and ESI-MS) of **(33)** 2-phenylnaphthalic anhydride 79
S61. Profiling data (UV chromatogram and ESI-MS) of **(34)** Rutin 80
S62. +HRLC(ESI)MS spectrum of 2005_01a Peak 1 81
S63. +HRLC(ESI)MS spectrum of 2005_01a Peak 2 81
S64. +HRLC(ESI)MS spectrum of 2005_01a Peak 3 82
S65. +HRLC(ESI)MS spectrum of 2005_01a Peak 4 82
S66. +HRLC(ESI)MS spectrum of 2005_01a Peak 5 83
S67. +HRLC(ESI)MS spectrum of 2005_01a Peak 6 83
S68. +HRLC(ESI)MS spectrum of 2005_01a Peak 7 84
S69. +HRLC(ESI)MS spectrum of 2005_01a Peak 8 84
S70. +HRLC(ESI)MS spectrum of 2005_01a Peak 9 85
S71. +HRLC(ESI)MS spectrum of 2005_01a Peak 10 85
S72. +HRLC(ESI)MS spectrum of 2005_01a Peak 11 86
S73. +HRLC(ESI)MS spectrum of 2005_01a Peak 11a 86
S74. +HRLC(ESI)MS spectrum of 2005_01a Peak 12 87
S75. +HRLC(ESI)MS spectrum of 2005_01a Peak 13 87
S76. +HRLC(ESI)MS spectrum of 2005_01a Peak 14 88
S77. +HRLC(ESI)MS spectrum of 2005_01a Peak 15 88
S78. +HRLC(ESI)MS spectrum of 2010_17b Peak 1 89
S79. +HRLC(ESI)MS spectrum of 2010_17b Peak 2 89
S80. +HRLC(ESI)MS spectrum of 2010_17b Peak 3 90
S81. +HRLC(ESI)MS spectrum of 2010_17b Peak 4 90
S82. +HRLC(ESI)MS spectrum of 2010_17b Peak 5 91
S83. +HRLC(ESI)MS spectrum of 2010_17b Peak 6 91
S84. +HRLC(ESI)MS spectrum of 2010_17b Peak 7 92
S85. +HRLC(ESI)MS spectrum of 2010_17b Peak 8 92
S86. +HRLC(ESI)MS spectrum of 2010_17b Peak 9 93
S87. +HRLC(ESI)MS spectrum of 2010_17b Peak 10 93
S88. +HRLC(ESI)MS spectrum of 2023_01a Peak 2 94

S89. +HRLC(ESI)MS spectrum of 2023_01a Peak 3 94
S90. +HRLC(ESI)MS spectrum of 2023_01a Peak 6 95
S91. +HRLC(ESI)MS spectrum of 2023_01a Peak 7 95
S92. +HRLC(ESI)MS spectrum of 2023_01a Peak 8 96
S93. +HRLC(ESI)MS spectrum of 2023_01a Peak 9 96
S94. +HRLC(ESI)MS spectrum of 2023_01a Peak 10 97
S95. +HRLC(ESI)MS spectrum of 2023_01a Peak 11 97
S96. +HRLC(ESI)MS spectrum of 2023_01a Peak 12 98
S97. +HRLC(ESI)MS spectrum of 2023_01a Peak 13 98
S98. +HRLC(ESI)MS spectrum of 2023_01a Peak 14 99
S99. +HRLC(ESI)MS spectrum of 2023_01a Peak 15 99
S100. +HRLC(ESI)MS spectrum of 2023_01a Peak 16 100
S101. +HRLC(ESI)MS spectrum of 2023_01a Peak 18 100
S102. +HRLC(ESI)MS spectrum of 2023_01a Peak 19 101
S103. +HRLC(ESI)MS spectrum of 2023_01a Peak 20 101
S104. +HRLC(ESI)MS spectrum of 2023_01a Peak 21 102
S105. +HRLC(ESI)MS spectrum of 2023_01a Peak 22 102
S106. +HRLC(ESI)MS spectrum of 2023_01a Peak 23 103
S107. +HRLC(ESI)MS spectrum of 2023_01a Peak 24 103
S108. +HRLC(ESI)MS spectrum of 2023_01a Peak 25 104
S109. +HRLC(ESI)MS spectrum of 2021_18c Peak 1 104
S110. +HRLC(ESI)MS spectrum of 2021_18c Peak 2........ 105
S111. +HRLC(ESI)MS spectrum of 2021_18c Peak 3 105
S112. +HRLC(ESI)MS spectrum of 2021_18c Peak 5 106
S113. +HRLC(ESI)MS spectrum of 2021_18c Peak 6 106
S114. +HRLC(ESI)MS spectrum of 2021_18c Peak 7 107
S115. +HRLC(ESI)MS spectrum of 2021_18c Peak 8 107
S116. +HRLC(ESI)MS spectrum of 2021_18c Peak 9 108
S117. +HRLC(ESI)MS spectrum of 2021_18c Peak 10 108
S118. +HRLC(ESI)MS spectrum of 2021_18c Peak 11 109
S119. +HRLC(ESI)MS spectrum of 2021_18c Peak 12 109
S120. +HRLC(ESI)MS spectrum of 2021_18c Peak 13........ 109
S121. +HRLC(ESI)MS spectrum of 2021_18c Peak 14 110

S122. Extract masses and yields from voucher samples 111

S1. Database of known phenylphenalenone and related compounds from Haemodoraceae and other species as of July 2025

#	Name	Class	Mass	UV	Structure	References
1	2-phenyl-1H-phenalen-1-one (Fuliginone)	2-PhP	256.3	333, 368, 431	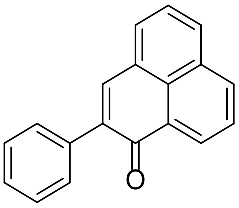	(Brkljača, White et al. 2015)
2	Fuliginosone (3-phenylacenaphthylene-1,2-dione)	Misc	260	240, 345	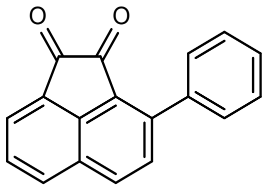	(Brkljača, White et al. 2015)
3	2-hydroxy-9-phenyl-1H-phenalen-1-one (Anigorufone)	9-PhP	272.3	410	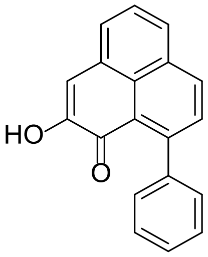	(Cooke and Thomas 1975)
4	4-hydroxy-9-phenyl-1H-phenalen-1-one	9-PhP	272.3	224, 272, 330, 448 (MeOH)	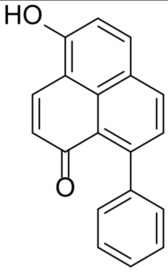	(Holscher and Schneider 1998)
5	6-hydroxy-9-phenyl-1H-phenalen-1-one	9-PhP	272.3	266, 295, 345, 360, 453	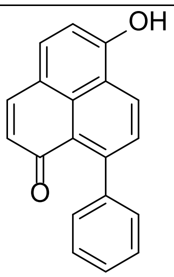	(Cooke 1955)
6	4-Phenyl-1*H*,3*H*-naphtho[1,8-*cd*]pyran-1,3-dione (2-phenylnaphthalic anhydride)	Misc	274.3	240, 323	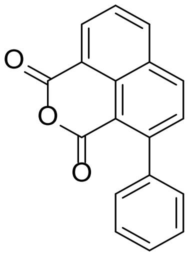	(Brkljača, White et al. 2015)

7	5-hydroxy-1H-naphtho[2,1,8-mna]xanthen-1-one	OBC	286.3	230, 260, 317, 329, 362, 379, 523	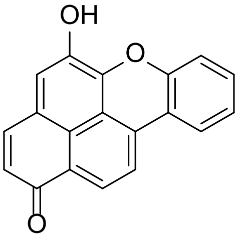	(Norman, Hombsch et al. 2021)
8	2-methoxy-9-phenyl-1H-phenalen-1-one	9-PhP	286.3	-	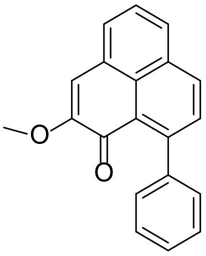	(Norman, Lever et al. 2019)
9	3-(4-methoxyphenyl)-1H-phenalen-1-one	3-PhP	286.3	-	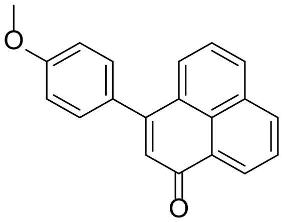	(Norman, Lever et al. 2019)
10	5-methoxy-7-phenyl-1H-phenalen-1-one	7-PhP	286.3	-	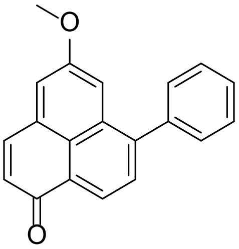	(Norman, Lever et al. 2019)
11	11-hydroxy-1H-naphtho[2,1,8-mna]xanthen-1-one	OBC	286.3	-	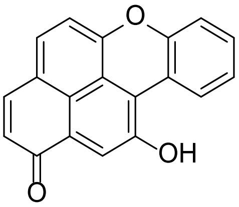	(Norman, Lever et al. 2019)
12	2-hydroxy-9-(4-hydroxyphenyl)-1H-phenalen-1-one (Hydroxyanigorufone)	9-PhP	288.3	225, 240sh, 272sh, 318, 357sh, 374, 430 (EtOH)	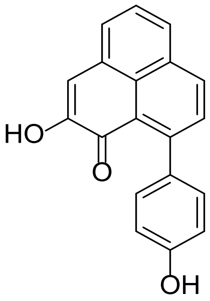	(Cooke and Thomas 1975)
13	2,6-dihydroxy-9-phenyl-1H-phenalen-1-one Lachnanthocarpone?	9-PhP	288.3	243, 270, 302, 368, 492	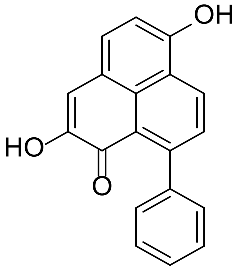	(Morrison, Laundon et al. 1971, Edwards and Weiss 1974)

14	5,6-dihydroxy-7-phenyl-1H-phenalen-1-one	7-PhP	288.3	243, 270, 302, 368, 492	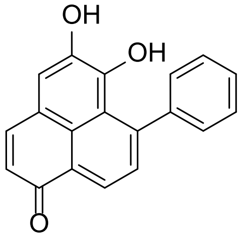	(Morrison, Laundon et al. 1971)
15	2,4-dihydroxy-9-phenyl-1H-phenalen-1-one	9-PhP	288.3	-	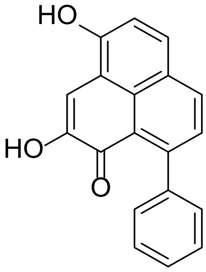	(Norman, Lever et al. 2019)
16	2-hydroxy-8-(4-hydroxyphenyl)-phenalen-1-one	8-PhP	288.3	197, 276, 418 (MeOH)	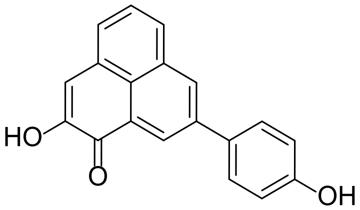	(Hoelscher and Schneider 2005)
17	5-hydroxy-7-phenyl-2H-benzo[de]isoquinoline-1,6-dione	PBIQ	289.3	(similar compound) 236, 322, 432	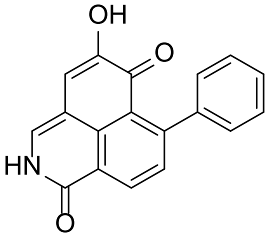	(Fang, Hoelscher et al. 2012)
18	5-amino-7-phenyl-benzo[de]isochromene-1,6-dione	PBIC	289.3	204, 244, 269, 327, 446	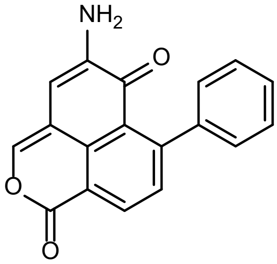	(Norman, Hombsch et al. 2021)
19	5-hydroxy-7-phenylbenzoisochromene-1,6-dione (lachnanthopyrone)	PBIC	290.3	243, 395	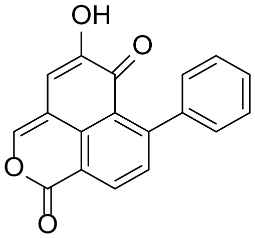	(Opitz and Schneider 2002)
20	5,6-dihydroxy-7-phenylbenzoisochromen-1-one	PBIC	292.3	215, 271, 382	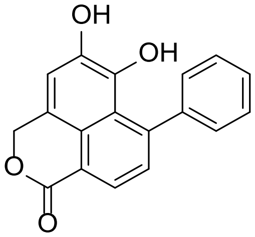	(Chen, Paetz et al. 2017)

21	5-methoxy-1H-naphtho[2,1,8-mna]xanthen-1-one	OBC	300.3	234, 280, 328, 523	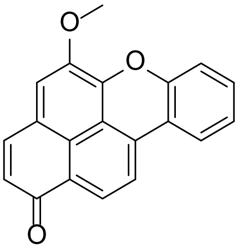	(Opitz, Otalvaro et al. 2002)
22	5-methoxy-1H-naphtho[2,1,8-mna]xanthen-3-one	OBC	300.3	228, 258, 312, 487, 513	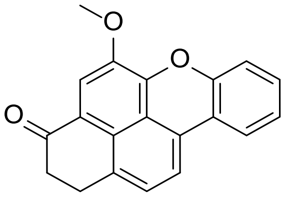	(Opitz, Otalvaro et al. 2002)
23	2-hydroxy-9-(4-methoxyphenyl)-1H-phenalen-1-one	9-PhP	302.3	-	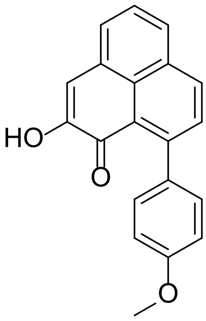	(Norman, Lever et al. 2019)
24	2-hydroxy-6-methoxy-9-phenyl-1H-phenalen-1-one	9-PhP	302.3	-	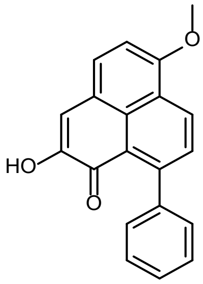	(Norman, Lever et al. 2019, Carpinelli de Jesus, Church et al. 2023)
25	6-hydroxy-5-methoxy-9-phenyl-1H-phenalen-1-one	9-PhP	302.3	-	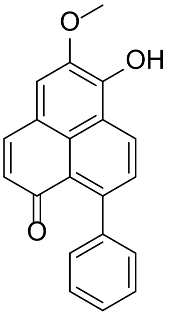	(Norman, Lever et al. 2019)
26	4-hydroxy-2-methoxy-9-phenyl-1H-phenalen-1-one	9-PhP	302.3	208, 288, 514 (MeOH)	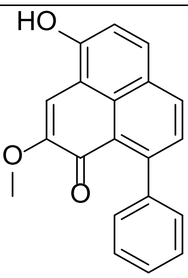	(Liu, Zhang et al. 2014)

27	2,5-dihydroxy-1H-naphtho[2,1,8-mna]xanthen-1-one	OBC	302.3	238, 320, 396, 553	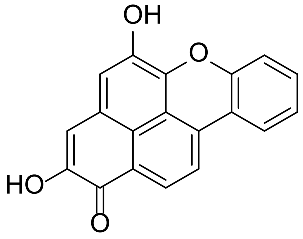	(Opitz, Otalvaro et al. 2002)
28	5-hydroxy-6-methoxy-7-phenyl-1H-phenalen-1-one	7-PhP	302.33	274, 369, 463 (MeOH)	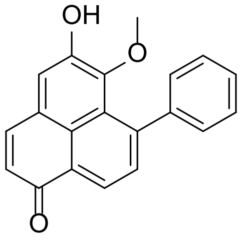	(Holscher and Schneider 1997)
29	8-hydroxy-7-methoxy-6-phenyl-1H-phenalen-1-one	6-PhP	302.3	215, 267, 428	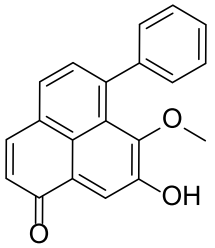	(Holscher and Schneider 1998)
30	2,5,6-trihydroxy-9-phenyl-1H-phenalen-1-one (lachnanthoside aglycone)	9-PhP	304.3	208, 278, 374	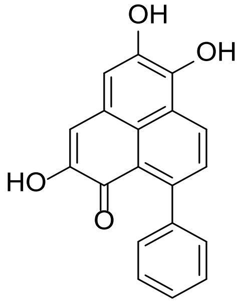	(Munde, Brand et al. 2013)
31	2,5,6-trihydroxy-7-phenyl-1H-phenalen-1-one	7-PhP	304.3	-	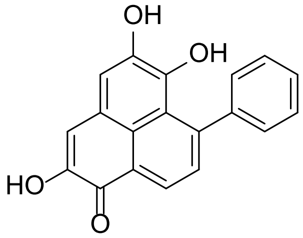	(Munde, Brand et al. 2013)
32	2-hydroxy-8-(3,4-dihydroxyphenyl)-phenalen-1-one	8-PhP	304.3	196, 275, 427	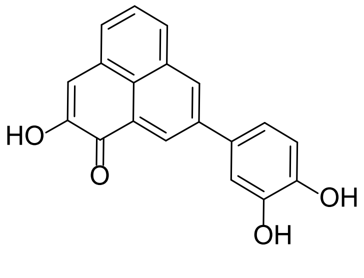	(Hoelscher and Schneider 2005)
33	5-methoxy-7-phenylbenzo[de]chromene-2,6-dione (Haemodordione)	Misc	304.3	255, 313, 373 (EtOH)	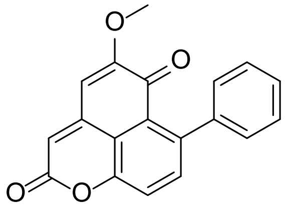	(Brkljača and Urban 2015)

34	2-hydroxy-9-(3,4-dihydroxyphenyl)-1H-phenalen-1-one (dihydroxyanigorufone)	9-PhP	304.3	219, 244, 261sh, 270sh, 292sh, 340sh, 355, 370, 425, 540sh (EtOH)	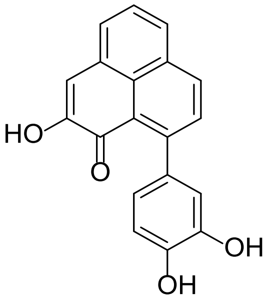	(Cooke and Thomas 1975)
35	2,4,6-trihydroxy-9-phenyl-1H-phenalen-1-one	9-PhP	304.3	-	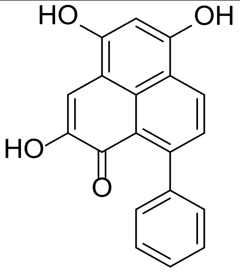	(Norman, Lever et al. 2019)
36	2,4-dihydroxy-9-(4'-hydroxyphenyl)-1H-phenalen-1-one	9-PhP	304.3	-	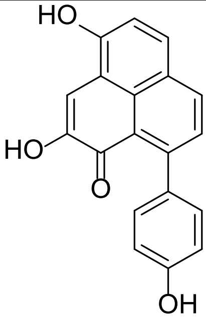	(Norman, Lever et al. 2019)
37	5-methoxy-7-phenylbenzo[de]chromene-1,6-dione	PBIC	304.3	243, 390	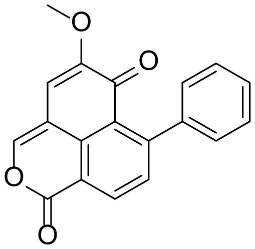	(Schneider, Paetz et al. 2005)
38	5,6-dihydroxy-7-phenylbenzo[de]chromene-1,3-dione	PBIC	306.3	-	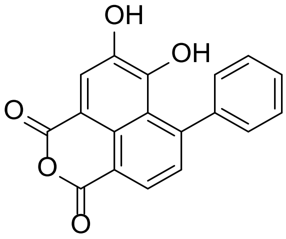	(Norman, Lever et al. 2019)
39	3,5-dihydroxy-1H,3H-isochromeno[6,5,4-mna]xanthen-1-one	Misc	306.3	230, 269, 435	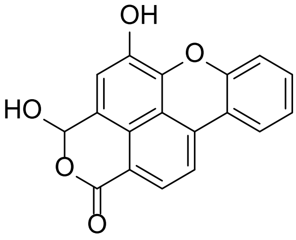	(Opitz, Otalvaro et al. 2002)

40	6-hydroxy-5-methoxy-7-phenylbenzo[de]chromen-1-one	PBIC	306.3	214, 268, 388	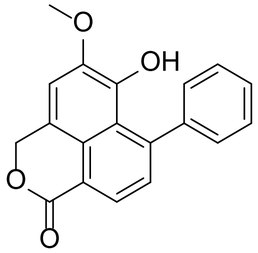	(Schneider, Paetz et al. 2005)
41	3,5,6-trihydroxy-7-phenylbenzo[de]chromen-1-one	PBIC	308.3	269, 341, 387	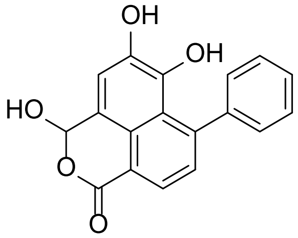	(Opitz, Hoelscher et al. 2002)
42	5-hydroxy-2-methoxy-1H-naphtho[2,1,8-mna]xanthen-1-one	OBC	316.3	237, 320, 386, 547	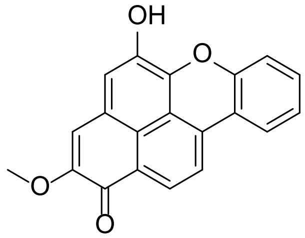	(Opitz, Otalvaro et al. 2002, Carpinelli de Jesus, Church et al. 2023)
43	2,6-dimethoxy-7-phenyl-1H-phenalen-1-one	7-PhP	316.3	244, 263, 295, 306, 347, 365, 458	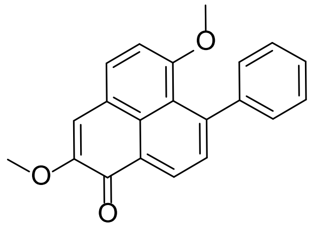	(Morrison, Laundon et al. 1971)
44	5,6-dimethoxy-7-phenyl-1H-phenalen-1-one	7-PhP	316.3	218, 271, 370, 454	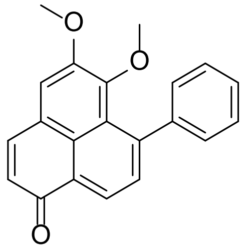	(Morrison, Laundon et al. 1971)
45	2,6-dimethoxy-9-phenyl-1H-phenalen-1-one	9-PhP	316.4	-	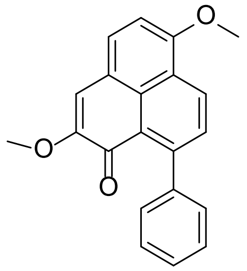	(Brkljača and Urban 2015)
46	2,4-dimethoxy-9-phenyl-1H-phenalen-1-one	9-PhP	316.4	231, 273, 430	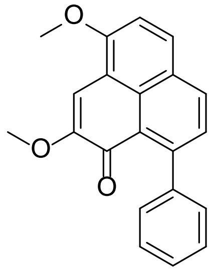	(Norman, Lever et al. 2019)

47	2,3-dimethoxy-4-phenyl-1H-phenalen-1-one	4-PhP	316.4	243, 342, 362	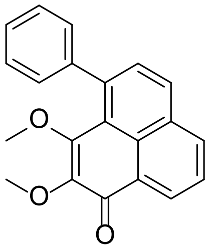	(Holscher and Schneider 1997)
48	7,8-dimethoxy-6-phenyl-1H-phenalen-1-one	6-PhP	316.4	212, 268, 429	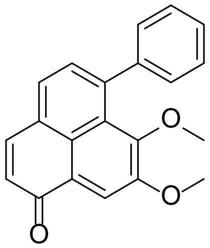	(Holscher and Schneider 1997)
49	2-methoxy-5-methyl-7-phenyl-1H-benzo[de]isoquinoline-1,6(2H)-dione	PBIQ	317.3	-	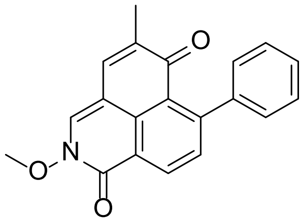	(Norman, Lever et al. 2019)
50	5,8,9-trihydroxy-1H-naphtho[2,1,8-mna]xanthen-1-one (Hemoflurone A)	OBC	318.3	572	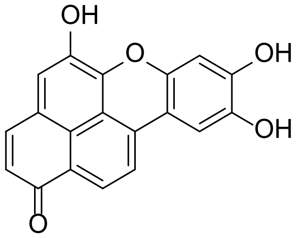	(Brkljača, White et al. 2015)
51	5,8,9-trihydroxy-3H-naphtho[2,1,8-mna]xanthen-3-one (Hemoflurone B)	Misc	318.3	551	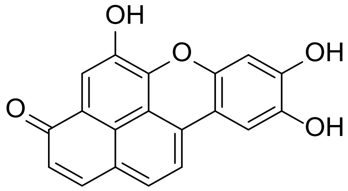	(Brkljača, White et al. 2015)
52	2,5-dihydroxy-6-methoxy-7-phenyl-1H-phenalen-1-one (Fuliginol)	7-PhP	318.3	275, 375, 463	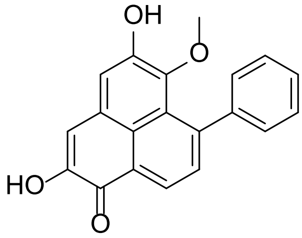	(Brkljača, White et al. 2015)
53	2,5-dihydroxy-6-methoxy-9-phenyl-1H-phenalen-1-one (anigozanthin)	9-PhP	318.3	-	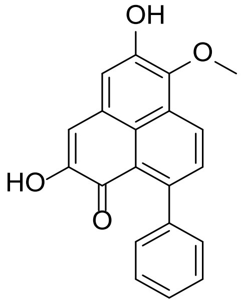	(Holscher and Schneider 1997)

54	2-Methoxy-9-(3',4'-dihydroxyphenyl)-1H-phenalen-1-one	9-PhP	318	205, 263, 268, 366, 412	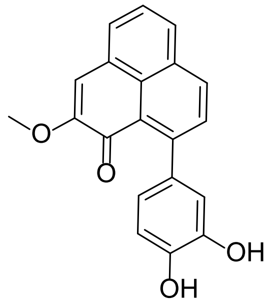	(Dong, He et al. 2011)
55	2,6-dihydroxy-5-methoxy-9-phenyl-1H-phenalen-1-one (Haemocorin aglycone)	9-PhP	318.3	250, 277, 300sh, 355, 372, 505 (dioxane)	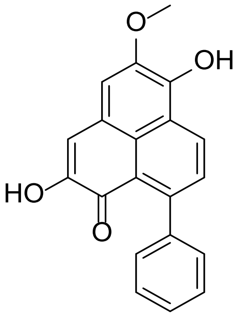	(Cooke 1955)
56	2,6-dihydroxy-5-methoxy-7-phenyl-1H-phenalen-1-one	7-PhP	318.3	-	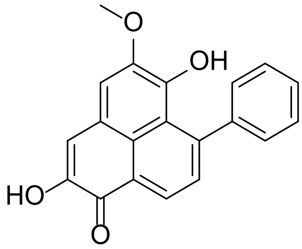	(Norman, Lever et al. 2019)
57	Haemodordioxolane (1-phenyl-5,8,10-trioxa-cyclopenta[a]phenalene-4,6-dione)	Misc	318.3	254, 314s, 327, 368, 421	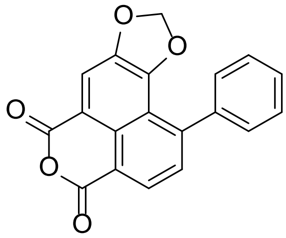	(Urban, Brkljaca et al. 2013)
58	2,4-dihydroxy-9-(3,4-dihydroxyphenyl)-1H-phenalen-1-one	9-PhP	320.3	277, 329, 446	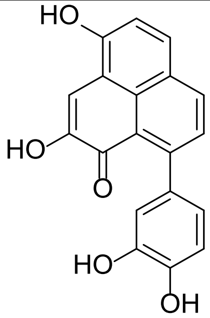	(Fang, Paetz et al. 2011)
59	5-hydroxy-6-methoxy-7-phenylbenzo[de]chromen-1,3-dione (Haemodorone)	PBIC	320.3	218, 259, 327, 344, 393	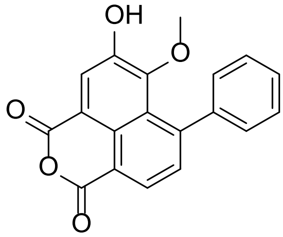	(Dias, Goble et al. 2009)

60	5-hydroxy-3-methoxy-7-phenylbenzo[de]chromen-1,6-dione	PBIC	320.3	267, 321	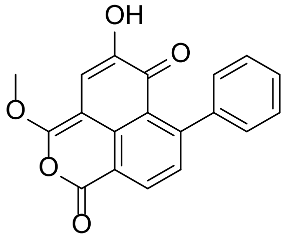	(Ocampos, Paetz et al. 2017)
61	6-hydroxy-5-methoxy-7-phenylbenzo[de]chromen-1,3-dione	PBIC	320.3	209, 268, 347, 413	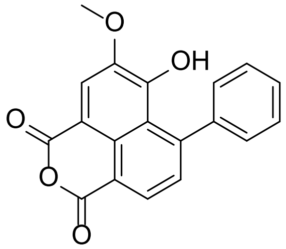	(Opitz, Hoelscher et al. 2002)
62	5-hydroxy-3-methoxy-isochromeno[6,5,4-mna]xanthenone	OBC	320	231, 269, 316, 439	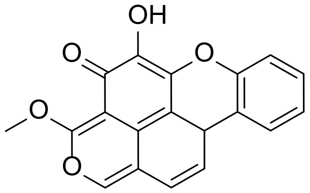	(Norman, Hombsch et al. 2021)
63	Haemodordiol (5,6-dihydroxy-3-methoxy-7-phenyl-1H-3H-benzo[de]isochromen-1-one)	PBIC	322.3	261, 326 (EtOH)	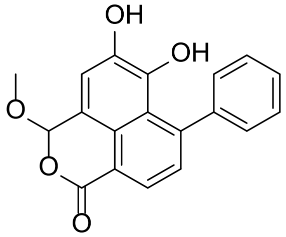	(Brkljača and Urban 2015) (Carpinelli de Jesus, Church et al. 2023)
64	3,6-dihydroxy-5-methoxy-7-phenyl-1H-3H-benzo[de]isochromen-1-one	PBIC	322.3	266, 340, 386	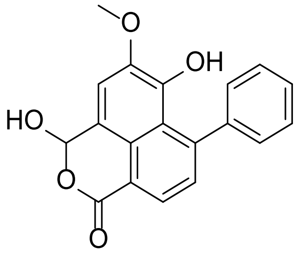	(Opitz, Hoelscher et al. 2002)
65	4-(4-methoxyphenyl)-2,3-dihydro-1H-phenalene-1,2,3-triol	Misc	322.4	-	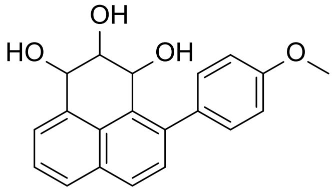	(Norman, Lever et al. 2019)
66	methyl 3-methoxy-5-phenyl-1,4-dihydronaphthoquinone-8-carboxylate	Misc	322	256, 337, 404	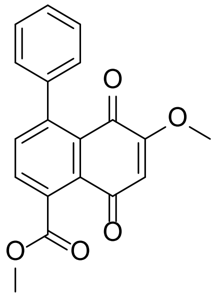	(Norman, Hombsch et al. 2021)

67	2,5-dimethoxy-1H-naphtho[2,1,8-mna]xanthen-1-one	OBC	330.3	238, 322, 264, 385, 544 (MeOH) 535 (CH3CN/D2O)	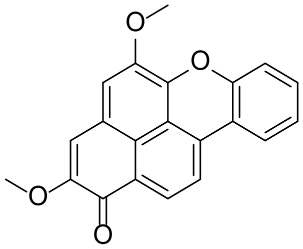	(Brkljača and Urban 2015) (Carpinelli de Jesus, Church et al. 2023)
68	5-hydroxy-2,6-dimethoxy-9-phenyl-1H-phenalen-1-one (Haemoxiphidone)	9-PhP	332.3	276, 375, 468	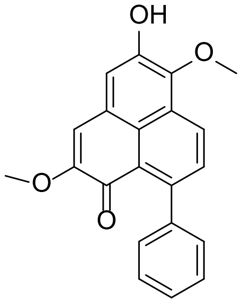	(Urban, Brkljaca et al. 2013, Brkljača and Urban 2015)
69	2-hydroxy-4,6-dimethoxy-9-phenyl-1H-phenalen-1-one (Haemodoronol)	9-PhP	332.4	275, 295, 400, 519	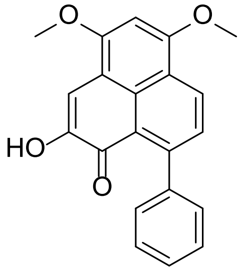	(Brkljača and Urban 2015)
70	6-hydroxy-2,5-dimethoxy-7-phenyl-1H-phenalen-1-one	7-PhP	332.4	218, 282, 356, 373, 514 and 245, 275, 352, 369, 478 and 278, 356, 372, 504	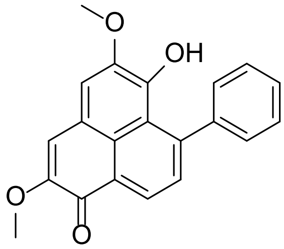	(Norman, Lever et al. 2019)
71	5-hydroxy-2,6-dimethoxy-7-phenyl-1H-phenalen-1-one (xiphidone)	7-PhP	332.4	274, 369, 463	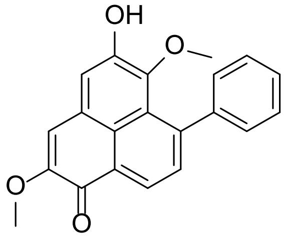	(Dias, Goble et al. 2009)
72	2-Methoxy-9-(3'-methoxy-4'-hydroxyphenyl)-1H-phenalen-1-one	9-PhP	332	217, 262, 365, 412	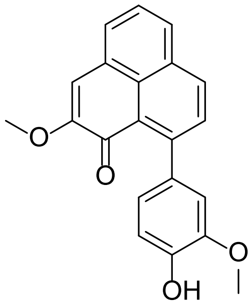	(Dong, He et al. 2011)

73	5-hydroxy-2-(2-hydroxyethyl)-7-phenyl-1H-benzo[de]isoquinoline-1,6(2H)-dione	PBIQ	333.3	-	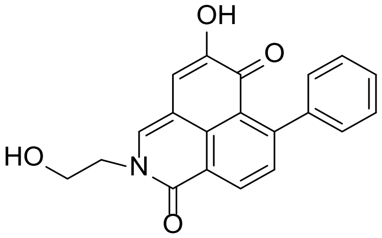	(Norman, Lever et al. 2019)
74	2,5-dimethoxy-7-phenyl-1H-benzo[de]isoquinoline-1,6(2H)-dione	PBIQ	333.3	-	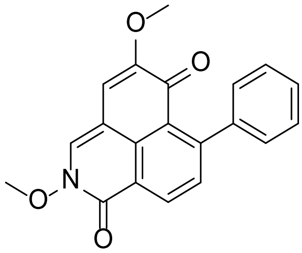	(Norman, Lever et al. 2019)
75	5,6-dimethoxy-7-phenylbenzo[de]isochromene-1,3-dione	PBIC	334.3	216, 259, 330, 346, 395	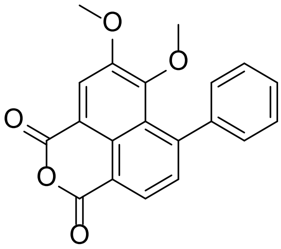	(Dias, Goble et al. 2009)
76	5,6-dimethoxy-9-phenyl benzo[de]isochromene-1,3-dione	PBIC	334	255, 307, 339	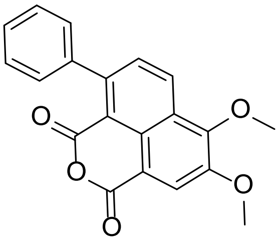	(Norman, Lever et al. 2019, Norman, Hombsch et al. 2021)
77	7,8-dihydroxy-3-oxo-6-phenyl-1H,3H-benzo[de]isochromene-1-carboxylic acid	PBIC	336.3		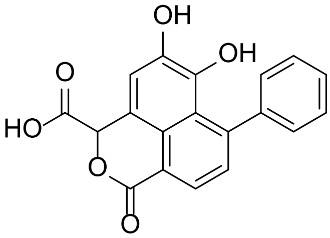	(Norman, Lever et al. 2019)
78	6-hydroxy-3,5-dimethoxy-7-phenylbenzo[de]chromen-1-one	PBIC	336.3	265, 323, 338, 384	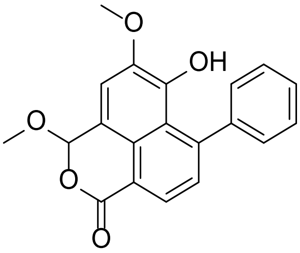	(Fang, Kai et al. 2012)
79	5-hydroxy-3,6-dimethoxy-7-phenylbenzo[de]chromen-1-one	PBIC	336.3	266, 340, 386	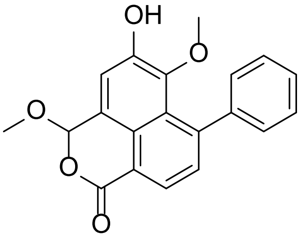	(Norman, Hombsch et al. 2021)

80	6-hydroxy-7-(4-hydroxyphenyl)-5-methoxybenzo[de]isochromene-1,3-dione (Haemodorol)	PBIC	336.3	215, 259, 348, 404	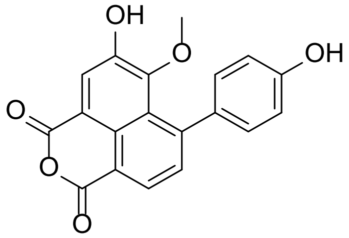	(Dias, Goble et al. 2009)
81	2-butyl-5-hydroxy-7-phenyl-1H-benzo[de]isoquinoline-1,6(2H)-dione	PBIQ	345.4	237, 266, 323, 442	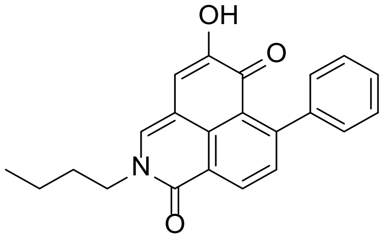	(Fang, Hoelscher et al. 2012)
82	5-methoxy-7-phenyl-2-propyl-1H-benzo[de]isoquinoline-1,6(2H)-dione	PBIQ	345.4	-	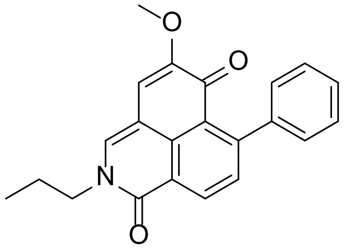	(Norman, Lever et al. 2019)
83	7-(4-hydroxyphenyl)-5-methyl-2-propyl-1H-benzo[de]isoquinoline-1,6(2H)-dione	PBIQ	345.4	-	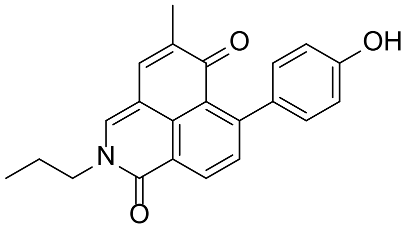	(Norman, Lever et al. 2019)
84	2,5,6-trimethoxy-9-phenyl-1H-phenalen-1-one	9-PhP	346.4	464	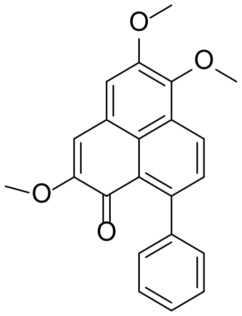	(Brkljača and Urban 2015)
85	2-(5-hydroxy-1,6-dioxo-7-phenyl-1H-benzo[de]isoquinolin-2(6H)-yl)acetic acid	PBIQ	347.3	236, 322, 439	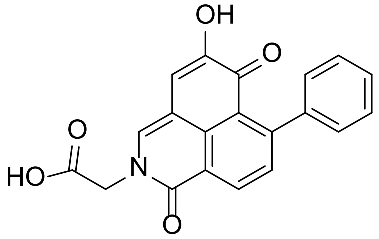	(Fang, Hoelscher et al. 2012)
86	6-acetoxy-5-methoxy-7-phenyl-3H-benzo[de]isochromen-1-one	PBIC	348	216, 255, 328, 360	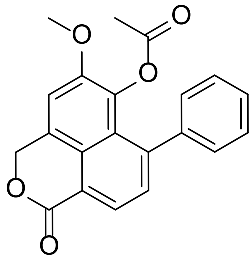	(Opitz, Hoelscher et al. 2002)

87	3-chloro-2,5-dihydroxy-6-methoxy-7-phenyl-1H-phenalen-1-one (3-chlorofuliginol)	7-PhP	352.8	275, 375, 463	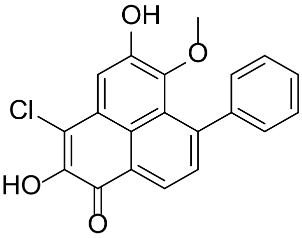	(Brkljača, White et al. 2015)
88	3-(5-hydroxy-1,6-dioxo-7-phenyl-1H-benzo[de]isoquinolin-2(6H)-yl)propanoic acid	PBIQ	361.3	-	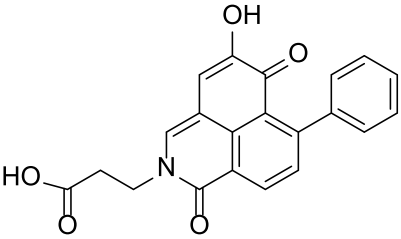	(Norman, Lever et al. 2019)
89	2-(n-Butyl)-5-hydroxy-7-(4-hydroxyphenyl)-2H-benzo[de]isoquinoline-1,6-dione	PBIQ	361	236, 266, 327, 425	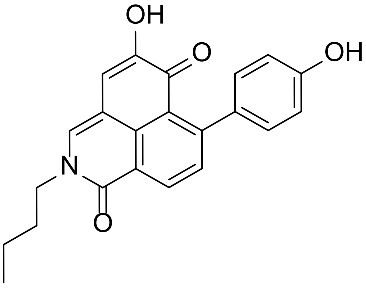	(Fang, Hoelscher et al. 2012)
90	2-(5-hydroxy-1,6-dioxo-7-phenyl-1H-benzo[de]isoquinolin-2(6H)-yl)propanoic acid	PBIQ	361.3	236, 322, 433	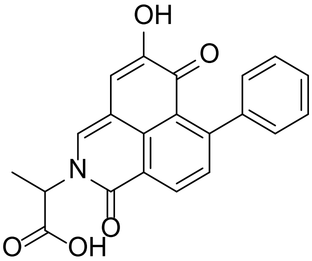	(Fang, Hoelscher et al. 2012)
91	2,3,4-trihydroxy-2-(2-oxopropyl)-9-phenyl-2,3-dihydro-1H-phenalen-1-one	PhP-Misc	362.4	-	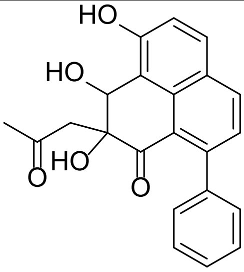	(Ocampos, Paetz et al. 2017)
92	Fulginosin A [3-(3,5-dihydroxyphenyl)-4-hydroxy-2-(4-hydroxyphenyl)benzofuran-6-carbaldehyde]	Misc	362	238, 268, 322, 362	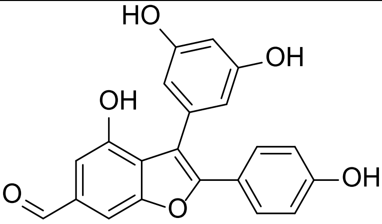	(Brkljača, White et al. 2015)
93	3,5-dihydroxy-5-(2-oxopropyl)-7-phenyl-3,5-dihydro-1H,6H-benzo[de]isochromene-1,6-dione	Misc	364.3	-	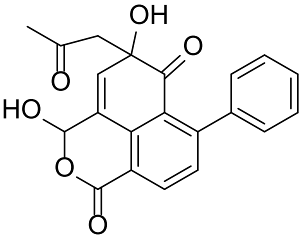	(Ocampos, Paetz et al. 2017)

94	2-(3-Carboxy-n-propyl)-5-hydroxy-7-phenyl-2H-benzo[de]isoquinoline-1,6-dione	PBIQ	375	236, 322, 439	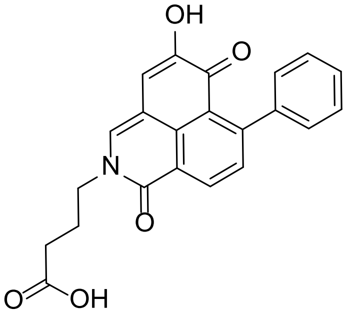	(Fang, Kai et al. 2012)
95	2,7,8-trihydroxy-2-(2-oxopropyl)-4-phenyl-1H-phenalene-1,3(2H)-dione	PhP-Misc	376.4	-	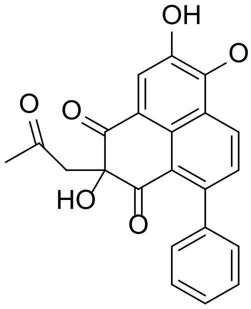	(Norman, Lever et al. 2019)
96	2,5,6-trihydroxy-2-(2-oxopropyl)-7-phenyl-1H-phenalene-1,3(2H)-dione	PhP-Misc	376.4	-	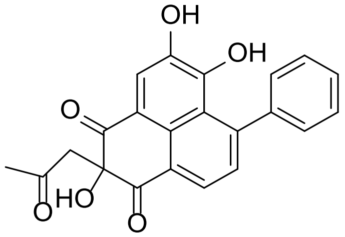	(Norman, Lever et al. 2019)
97	2-(2-hydroxyethyl)-5-((2-hydroxyethyl)amino)-7-phenyl-1H-benzo[de]isoquinoline-1,6(2H)-dione	PBIQ	376.4	-	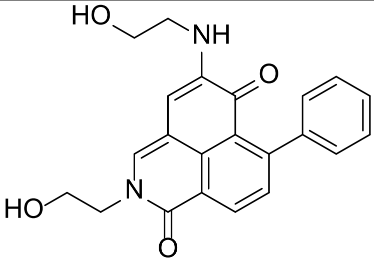	(Norman, Lever et al. 2019)
98	3-hydroxy-2-(5-hydroxy-1,6-dioxo-7-phenyl-1H-benzo[de]isoquinolin-2(6H)-yl)propanoic acid	PBIQ	377.4	214, 238, 322, 435	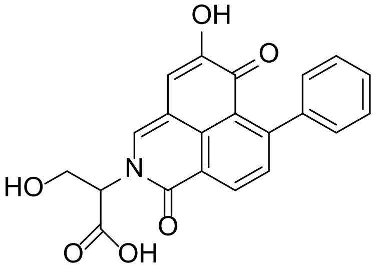	(Chen, Paetz et al. 2016)
99	3,5-dihydroxy-7-(4-hydroxyphenyl)-5-(2-oxopropyl)-3,5-dihydro-1H,6H-benzo[de]isochromene-1,6-dione	Misc	380.3	222, 243, 292	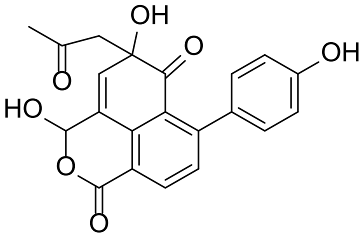	(Ocampos, Paetz et al. 2017)

100	2-(5-hydroxy-1,6-dioxo-7-phenyl-1H-benzo[de]isoquinolin-2(6H)-yl)-3-methylbutanoic acid	PBIQ	389.4	236, 265, 322, 427	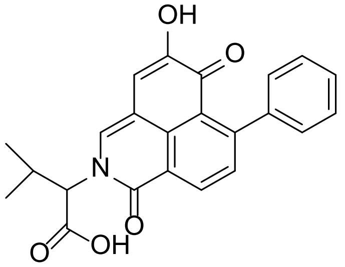	(Fang, Kai et al. 2012)
101	6-hydroxy-2-methoxy-6-(2-oxopropyl)-9-phenyl-1H-phenalene-1,3,5(2H,6H)-trione	PhP-Misc	390.4	-	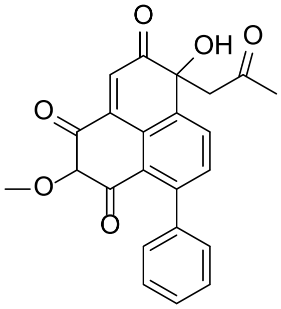	(Norman, Lever et al. 2019)
102	2,7-dihydroxy-8-methoxy-2-(2-oxopropyl)-4-phenyl-1H-phenalene-1,3(2H)-dione	PhP-Misc	390.4	-	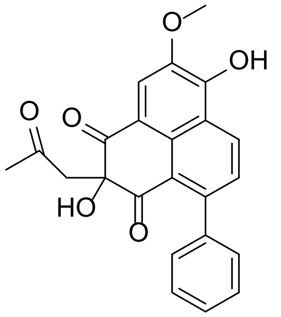	(Norman, Lever et al. 2019)
103	3-amino-2-(5-hydroxy-1,6-dioxo-7-phenyl-1H-benzo[de]isoquinolin-2(6H)-yl)-3-oxopropanoic acid	PBIQ	390.4	-	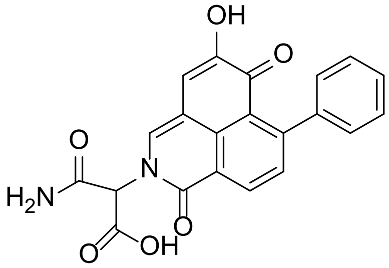	(Norman, Lever et al. 2019)
104	3-hydroxy-2-(5-hydroxy-1,6-dioxo-7-phenyl-1H-benzo[de]isoquinolin-2(6H)-yl)butanoic acid	PBIQ	391.4	205, 237, 323, 435	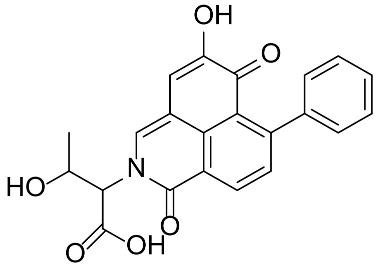	(Chen, Paetz et al. 2016)
105	2,3,6-trihydroxy-5-methoxy-2-(2-oxopropyl)-7-phenyl-2,3-dihydro-1H-phenalen-1-one	PhP-Misc	392.4	216, 270, 394	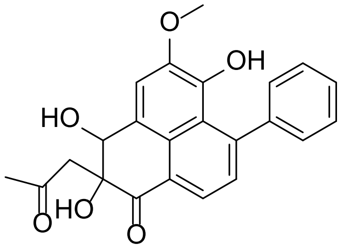	(Ocampos, Paetz et al. 2017)

106	2-hydroxy-3,7-dioxo-4-phenyl-7,9,10,11,11a,11b-hexahydro-3H-11l4-benzo[de]thiazolo[2,3-a]isoquinoline-9-carboxylic acid	PBIQ	395.4	-	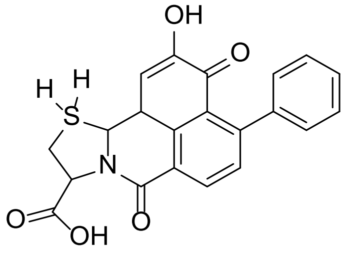	(Norman, Lever et al. 2019)
107	2-(5-hydroxy-1,6-dioxo-7-phenyl-1H-benzo[de]isoquinolin-2(6H)-yl)-3-methylpentanoic acid	PBIQ	403.4	-	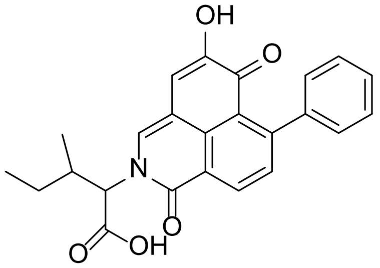	(Norman, Lever et al. 2019)
108	2-(5-hydroxy-1,6-dioxo-7-phenyl-1H-benzo[de]isoquinolin-2(6H)-yl)-4-methylpentanoic acid	PBIQ	403.4	-	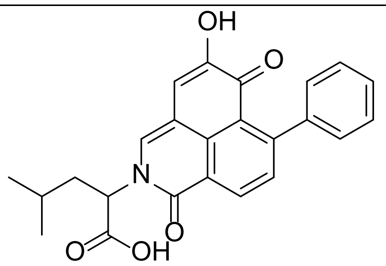	(Norman, Lever et al. 2019)
109	3-(3-acetyl-5-hydroxy-1-oxo-7-phenyl-1H,3H-benzo[de]isochromen-6-yl)-3-oxopropanoic acid	PBIC	404.4	-	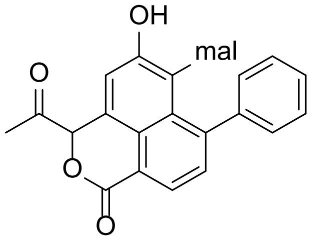	(Norman, Lever et al. 2019)
110	(1”)-2-(1”-Carboxy-2”-carboxamide-ethyl)-5-hydroxy-7-phenyl-2H-benzo[de]isoquinoline-1,6-dione	PBIQ	404.4	204, 237, 326, 437	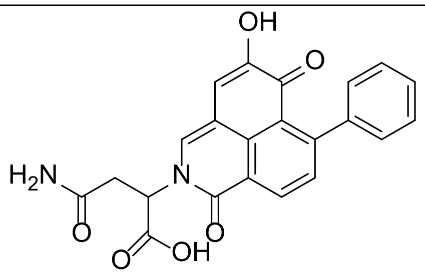	(Chen, Paetz et al. 2016)
111	2-(5-hydroxy-1,6-dioxo-7-phenyl-1H-benzo[de]isoquinolin-2(6H)-yl)succinic acid	PBIQ	405.4	237, 321, 434	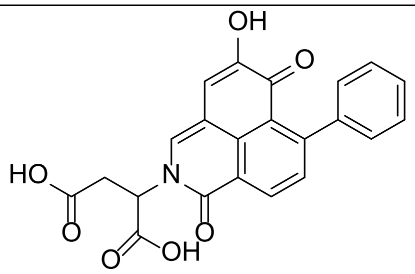	(Fang, Hoelscher et al. 2012)

112	Fulginosin B [5-(6-(dimethoxymethyl)-4-hydroxy-2-(4-hydroxyphenyl)benzofuran-3-yl)benzene-1,3-diol]	Misc	408	238, 268, 322, 362	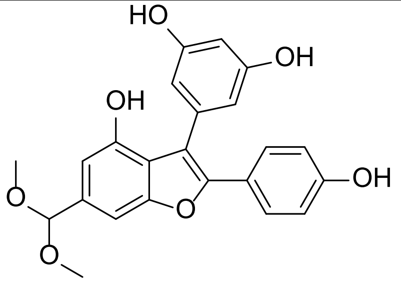	(Brkljača, White et al. 2015)
113	(1”S)-2-(1”-Carboxy-3”-carboxamide-propyl)-5-hydroxy-7-phenyl-2H-benzo[de]isoquinoline-1,6-dione	PBIQ	418	205, 239, 323, 436	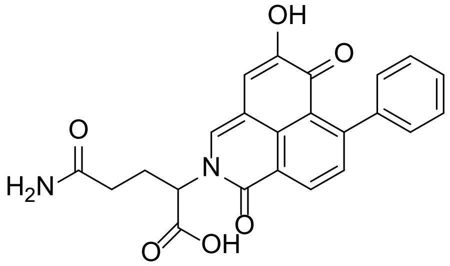	(Chen, Paetz et al. 2016)
114	(1”S)-2-(1”,3”-Dicarboxyethyl)-5-hydroxy-7-phenyl-2H-benzo [de]isoquinoline-1,6-dione	PBIQ	419	204, 239, 323, 436	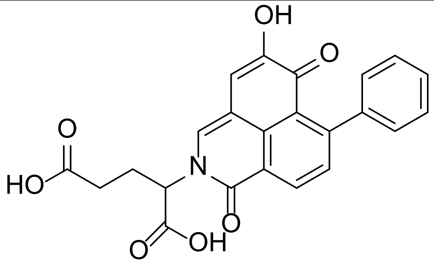	(Chen, Paetz et al. 2016)
115	3-Carboxy-5-hydroxy-6-O-malonyl-7-phenyl-3H-benzo[de]isochromen-1-one	PBIC	422	220, 253, 325, 357	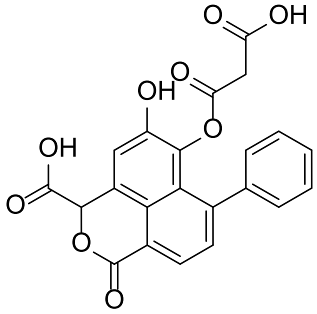	(Fang, Kai et al. 2012)
116	2-[1”-Carboxy-2’’’-(1’’’H-imidazol-5’’’-yl)-ethyl)]-5-hydroxy-7-phenyl-2H-benzo[de]isoquinoline-1,6-dione	PBIQ	427.4	208, 240, 323, 441	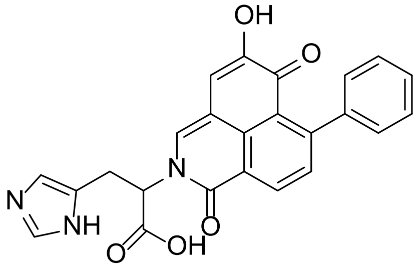	(Chen, Paetz et al. 2016)
117	4-guanidino-2-(5-hydroxy-1,6-dioxo-7-phenyl-1H-benzo[de]isoquinolin-2(6H)-yl)butanoic acid	PBIQ	432.4	-	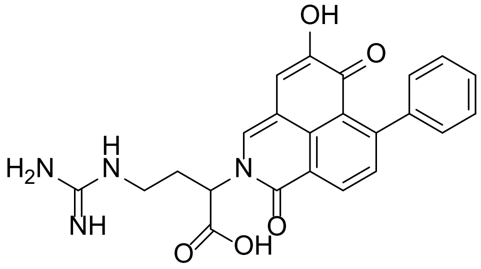	(Norman, Lever et al. 2019)

118	2-(5-hydroxy-1,6-dioxo-7-phenyl-1H-benzo[de]isoquinolin-2(6H)-yl)-3-phenylpropanoic acid	PBIQ	437.5	237, 322, 433	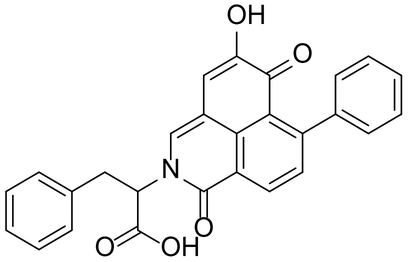	(Fang, Kai et al. 2012)
119	(1”S)-2-(1”-Carboxy-4”-diaminomethylideneamino-n-butyl)-5-hydroxy-7-phenyl-2H-benzo[de]isoquinoline-1,6-dione	PBIQ	446	205, 238, 323, 436	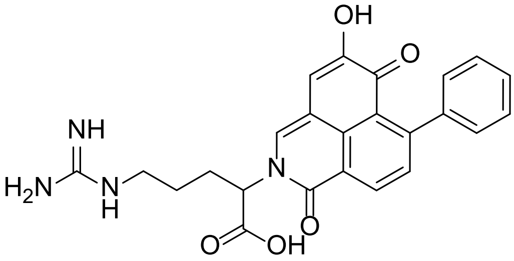	(Chen, Paetz et al. 2016)
120	Angiopressin A	Misc	452	263, 303, 355	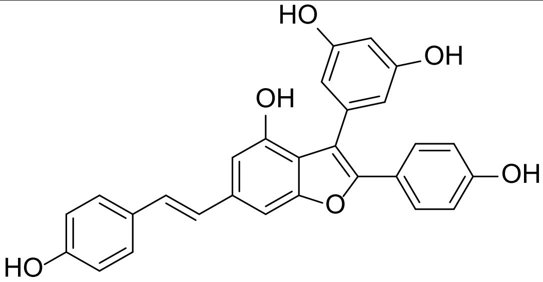	(Brkljača, White et al. 2015)
121	2-(5-hydroxy-7-(4-hydroxyphenyl)-1,6-dioxo-1H-benzo[de]isoquinolin-2(6H)-yl)-3-phenylpropanoic acid	PBIQ	453.5	206, 237, 325, 443	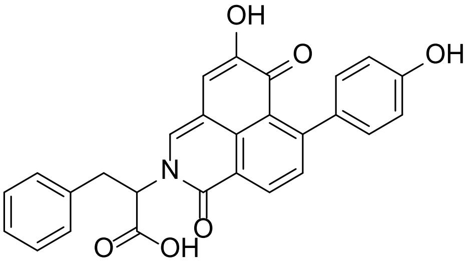	(Norman, Lever et al. 2019)
122	5-hydroxy-7-phenyl-6-((3,4,5-trihydroxy-6-(hydroxymethyl)tetrahydro-2H-pyran-2-yl)oxy)-1H,3H-benzo[de]isochromen-1-one	PBIC	454.4	220, 260, 334, 371	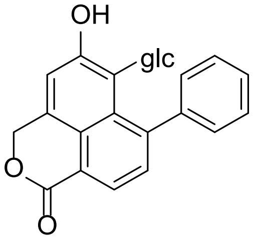	(Fang, Paetz et al. 2011) (Carpinelli de Jesus, Church et al. 2023)
123	2-methoxy-9-phenyl-6-((3,4,5-trihydroxy-6-(hydroxymethyl)tetrahydro-2H-pyran-2-yl)oxy)-1H-phenalen-1-one	9-PhP	464.5	-	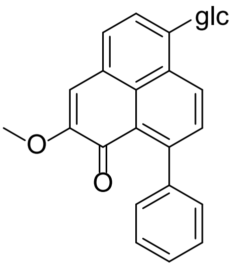	(Norman, Lever et al. 2019)

124	2-(3,4-dihydroxyphenyl)-5,7-dihydroxy-3-((3,4,5-trihydroxy-6-(hydroxymethyl)tetrahydro-pyran-2-yl)oxy)-chromen-4-one	PBIC	464	259, 359 (MeOH)	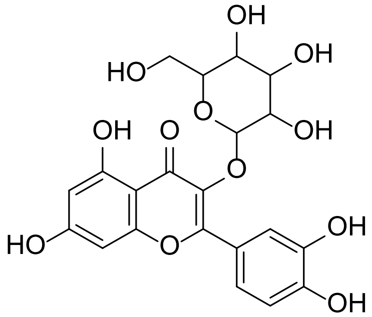	(Norman, Hombsch et al. 2021)
125	2,5-dihydroxy-7-phenyl-6-((3,4,5-trihydroxy-6-(hydroxymethyl)tetrahydro-2H-pyran-2-yl)oxy)-1H-phenalen-1-one	7-PhP	466.4	-	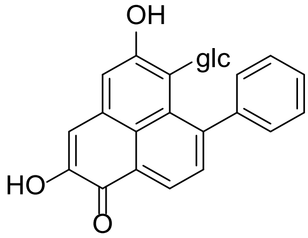	(Norman, Hombsch et al. 2021)
126	7-(4-hydroxyphenyl)-5-((3,4,5-trihydroxy-6-(hydroxymethyl)-tetrahydro-pyran-2-yl)oxy)-phenalen-1-one	7-PhP	466	271, 372, 437	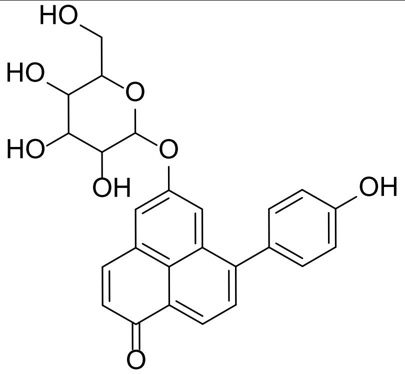	(Norman, Hombsch et al. 2021)
127	2-hydroxy-8-(2-hydroxyphenyl)-6-((3,4,5-trihydroxy-6-(hydroxymethyl)tetrahydro-pyran-2-yl)oxy)-phenalenone	8-PhP	466	248, 289, 457	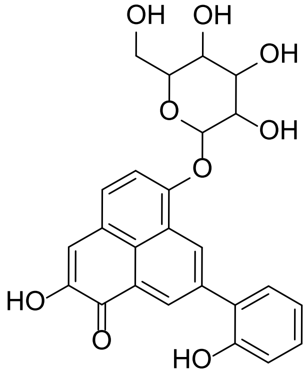	(Norman, Hombsch et al. 2021)
128	5-hydroxy-8-(2-hydroxyphenyl)-6-((3,4,5-trihydroxy-6-(hydroxymethyl)tetrahydro-pyran-2-yl)-oxy)-phenalenone	8-PhP	466	242, 257, 393	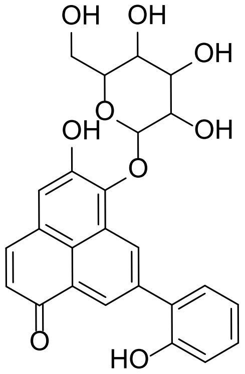	(Norman, Hombsch et al. 2021)

129	5-methoxy-7-phenyl-6-((3,4,5-trihydroxy-6-(hydroxymethyl)tetrahydro-2H-pyran-2-yl)oxy)-1H,3H-benzo[de]isochromen-1-one	PBIC	468.5	316, 323, 336, 361, 369	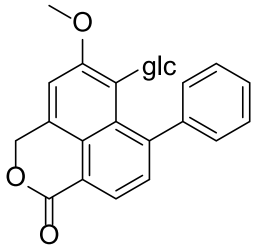	(Norman, Lever et al. 2019)
130	5-hydroxy-7-(4-hydroxyphenyl)-6-((3,4,5-trihydroxy-6-(hydroxymethyl)tetrahydro-2H-pyran-2-yl)oxy)-1H,3H-benzo[de]isochromen-1-one	PBIC	470.4	223, 255, 326, 369	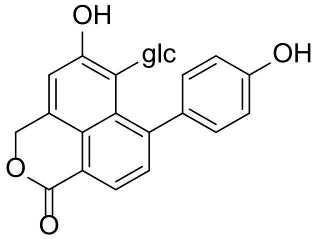	(Fang, Paetz et al. 2011) (Carpinelli de Jesus, Church et al. 2023)
131	(3R/S)-3,5-dihydroxy-7-phenyl-6-((3,4,5-trihydroxy-6-(hydroxymethyl)tetrahydro-pyran-2-yl)oxy)-benzo[de]isochromenone	PBIC	470.4	218, 252, 336, 364	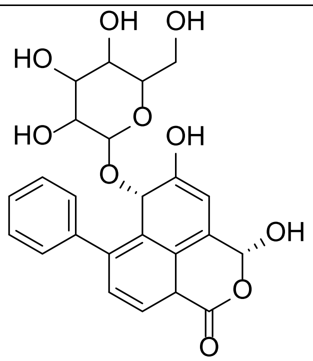	(Chen, Paetz et al. 2019)
132	2-(5-hydroxy-1,6-dioxo-7-phenyl-1H-benzo[de]isoquinolin-2(6H)-yl)-3-(1H-indol-3-yl)propanoic acid	PBIQ	476.5	206, 221, 243, 273, 327, 443	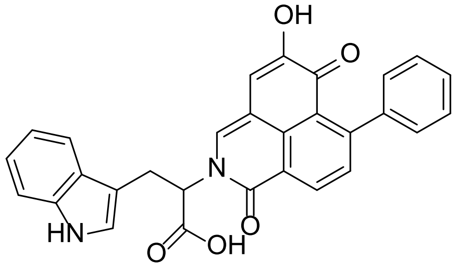	(Chen, Paetz et al. 2016)
133	5-methoxy-8-((3,4,5-trihydroxy-6-(hydroxymethyl)tetrahydro-2H-pyran-2-yl)oxy)-1H-naphtho[2,1,8-mna]xanthen-1-one (Haemodoroxychrysenose)	OBC	478.4	236s, 266s, 288s, 371s, 402, 537	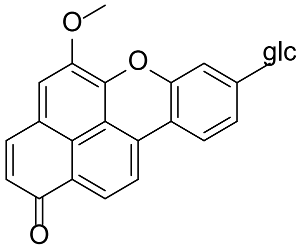	(Dias, Goble et al. 2009, Norman, Hombsch et al. 2021)
134	5-hydroxy-2-methoxy-7-phenyl-6-(3,4,5-trihydroxy-6-(hydroxy-methyl)tetrahydro-2H-pyran-2-yloxy)-1H-phenalen-1-one (Dilatrin)	7-PhP	480.5	214, 279, 360, 375, 474	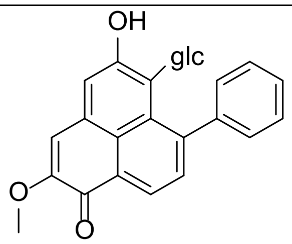	(Dias, Goble et al. 2009, Norman, Hombsch et al. 2021)

135	2,5-dimethoxy-7-phenyl-6-(3,4,5-trihydroxy-6-(hydroxy-methyl)tetrahydro-2H-pyran-2-yloxy)-1H-phenalen-1-one	7-PhP	494.5	-	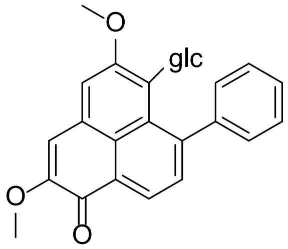	(Norman, Lever et al. 2019)
136	3-acetyl-5-hydroxy-7-phenyl-6-((3,4,5-trihydroxy-6-(hydroxymethyl)tetrahydro-2H-pyran-2-yl)oxy)-1H,3H-benzo[de]isochromen-1-one	PBIC	496.5	-	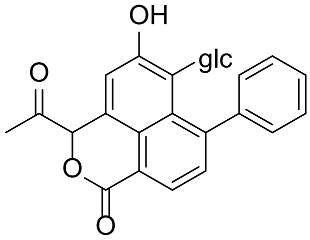	(Norman, Lever et al. 2019)
137	5-hydroxy-(3R/S)-3-carboxy-7-phenyl-6-((3,4,5-trihydroxy-6-(hydroxymethyl)tetrahydro-pyran-2-yl)oxy)-benzo[de]-isochromenone	PBIC	498.4	221, 260, 334, 367	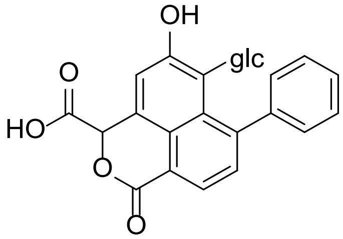	(Opitz, Schnitzler et al. 2003, Norman, Hombsch et al. 2021)
138	(3,4,5-trihydroxy-6-((5-hydroxy-1-oxo-7-phenyl-1H,3H-benzo[de]isochromen-6-yl)oxy)tetrahydro-2H-pyran-2-yl)methyl hydrogen carbonate	PBIC	498.4	-	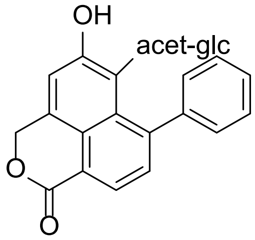	(Norman, Lever et al. 2019)
139	3-oxo-3-((3,4,5-trihydroxy-6-((2-hydroxy-1-oxo-9-phenyl-1H-phenalen-4-yl)oxy)tetrahydro-2H-pyran-2-yl)methoxy)propanoic acid	9-PhP	536.5	-	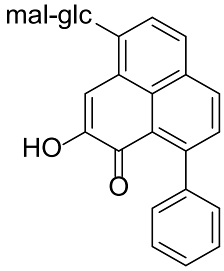	(Norman, Lever et al. 2019)
140	6-O-[6”-O-malonyl-b-D-glucopyranosyl]-5-hydroxy-7-phenyl-3H-benzo[de]isochromen-1-one	PBIC	540.5	258, 337, 368	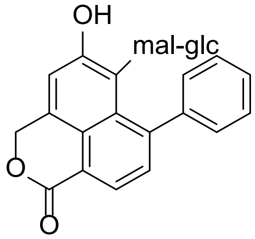	(Fang, Hoelscher et al. 2012) (Carpinelli de Jesus, Church et al. 2023)
141	5-hydroxy-6-allophanylglucopyranosyl-7-phenylisochromen-1-one	PBIC	540.5	214, 260, 334, 366	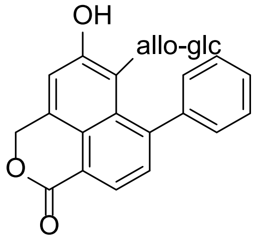	(Norman, Lever et al. 2019)

142	5-methoxy-6-allophanylglucopyranosyl-9-phenyl-1H-phenalen-1-one	9-PhP	550.5	-	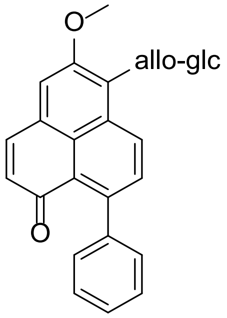	(Norman, Lever et al. 2019)
143	3-oxo-3-((3,4,5-trihydroxy-6-((2-methoxy-1-oxo-9-phenyl-1H-phenalen-4-yl)oxy)tetrahydro-2H-pyran-2-yl)methoxy)propanoic acid	9-PhP	550.5	-	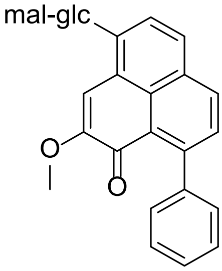	(Norman, Lever et al. 2019)
144	2,5-dihydroxy-6-allophanylglucopyranosyl-7-phenyl-1H-phenalen-1-one	7-PhP	552.5	211, 279, 378, 477	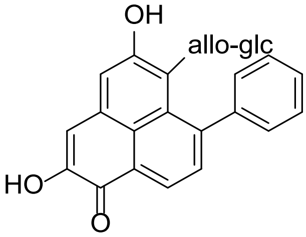	(Schneider, Paetz et al. 2005)
145	2,5-dihydroxy-6-malonylglucopyranosyl-7-phenyl-1H-phenalen-1-one	7-PhP	552.5	277, 374, 474	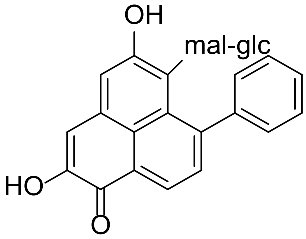	(Fang, Hoelscher et al. 2012) (Carpinelli de Jesus, Church et al. 2023)
146	5-methoxy-6-allophanylglucopyranosyl-7-phenylisochromen-1-one	PBIC	554.5	199, 260, 336, 370	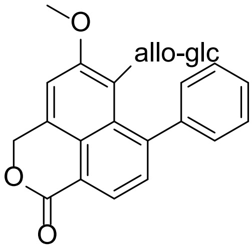	(Opitz, Hoelscher et al. 2002)
147	5-methoxy-6-malonylglucopyranosyl-7-phenylisochromen-1-one	PBIC	554.5	259, 333, 364	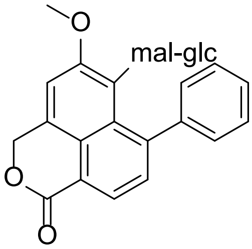	(Fang, Kai et al. 2012)
148	5-hydroxy-6-allophanylglucopyranosyl-7(4-hydroxyphenyl)-isochromen-1-one	PBIC	556.5	-	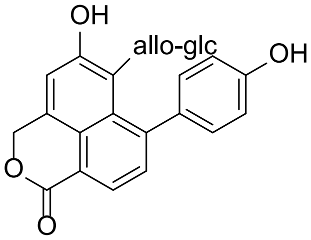	(Norman, Lever et al. 2019)

149	6-O-[(6"-O-malonyl)-β-D-glucopyranosyl]-5-hydroxy-7-(4'-hydroxyphenyl)-3H-benzo[de]isochromen-1-one	PBIC	556	225, 243, 255, 337, 366	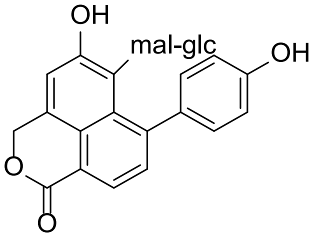	(Norman, Lever et al. 2019) (Carpinelli de Jesus, Church et al. 2023)
150	5-hydroxy-2-methoxy-6-malonylglucopyranosyl-7-phenyl-1H-phenalen-1-one	7-PhP	566.5	277, 374, 474	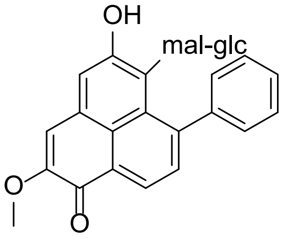	(Fang, Kai et al. 2012) *
151	5-hydroxy-2-methoxy-6-allophanylglucopyranosyl-7-phenyl-1H-phenalen-1-one	7-PhP	566.5	209, 279, 375, 470	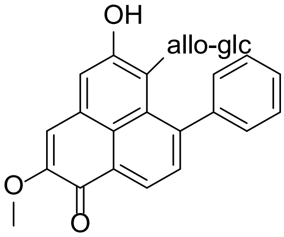	(Opitz, Hoelscher et al. 2002)
152	2-hydroxy-5-methoxy-6-malonylglucopyranosyl-7-phenyl-1H-phenalen-1-one	7-PhP	566.5	278, 374, 478	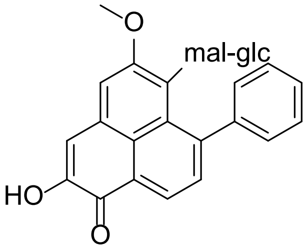	(Fang, Hoelscher et al. 2012)
153	p-hydroxycinnamate of salipurposide	Misc	580	317, 368	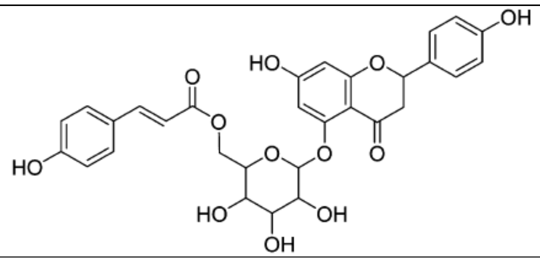	(Brkljača, White et al. 2015)
154	Rutin	Misc	610	353	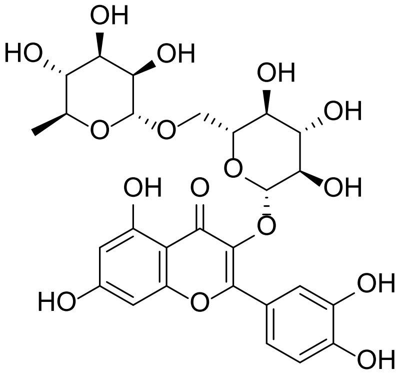	(Brkljača, White et al. 2015)

155	2-(3,4-dihydroxyphenyl)-5,7-dihydroxy-3-((3,4,5-trihydroxy-6-(((3,4,5-trihydroxy-6-(hydroxymethyl)tetrahydro-pyran-2-yl)-oxy)methyl)tetrahydro-pyran-2-yl)oxy)-chromen-4-one	PBIC	626	256, 265, 364	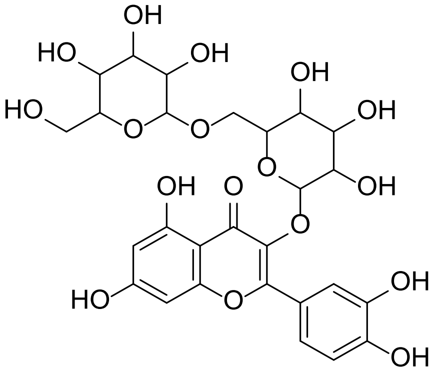	(Norman, Hombsch et al. 2021)
156	8-(2-hydroxyphenyl)-5,6-bis((3,4,5-trihydroxy-6-(hydroxy-methyl)tetrahydro-pyran-2-yl)oxy)-phenalenone	8-PhP	628	257, 283s, 416	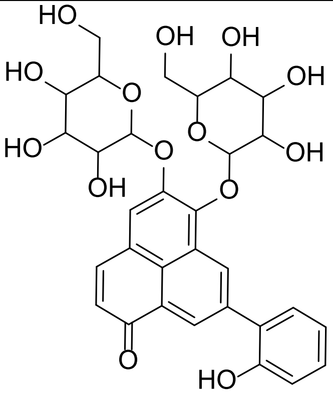	(Norman, Hombsch et al. 2021)
157	6-hydroxy-5-methoxy-8-glucopyranosyl-1->4-glucopyranosyl-9-phenyl-1H-phenalen-1-one	9-PhP	642.6	-	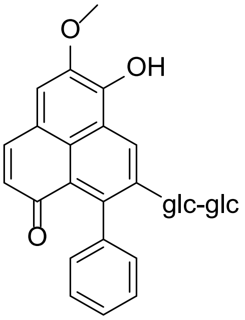	(Norman, Hombsch et al. 2021)
158	6-((4,5-dihydroxy-6-(hydroxymethyl)-3-((3,4,5-trihydroxy-6-(hydroxymethyl)tetrahydro-pyran-2-yl)oxy)tetrahydro-pyran-2-yl)oxy)-5-hydroxy-2-methoxy-7-phenyl-phenalen-1-one	7-PhP	642	278, 373, 465	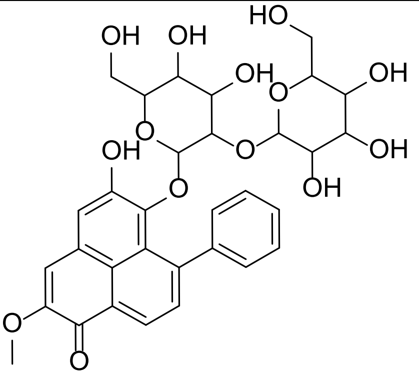	(Norman, Hombsch et al. 2021)

159	7-(3,4-dihydroxyphenyl)-5-((3,4,5-trihydroxy-6-(((3,4,5-trihydroxy-6-(hydroxymethyl)tetrahydro-pyran-2-yl)oxy)methyl)-tetrahydro-pyran-2-yl)oxy)-2-hydroxy-phenalenone	7-PhP	644	269, 371, 435	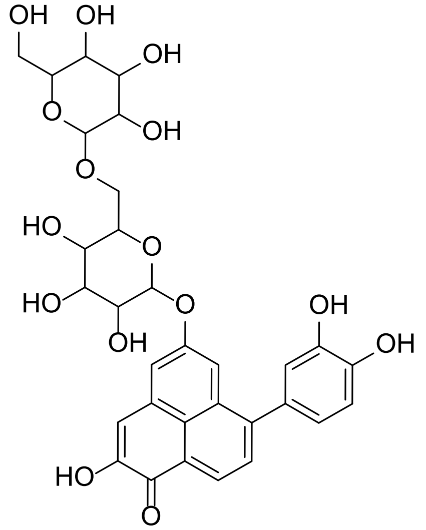	(Norman, Hombsch et al. 2021)
160	2-((5-acetoxy-1-oxo-7-phenyl-benzo[de]isochromen-6-yl)oxy)-6-(acetoxymethyl)tetrahydro-pyran-3,4,5-triyl triacetate	7-PBIC	664	259, 333, 365	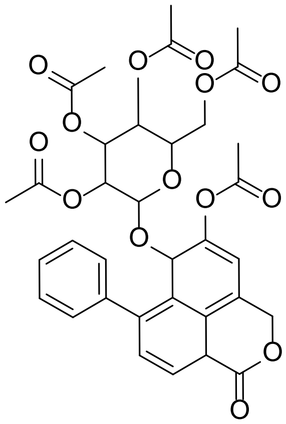	(Norman, Hombsch et al. 2021)
161	(6-((2-((2,5-dimethoxy-1-oxo-7-phenyl-phenalen-6-yl)oxy)-4,5-dihydroxy-6-(hydroxymethyl)tetrahydro-pyran-3-yl)oxy)-3,4,5-trihydroxytetrahydro-pyran-2-yl)methyl (E)-3-(3,4-dihydroxyphenyl)acrylate	7-PhP	818	279, 336, 465	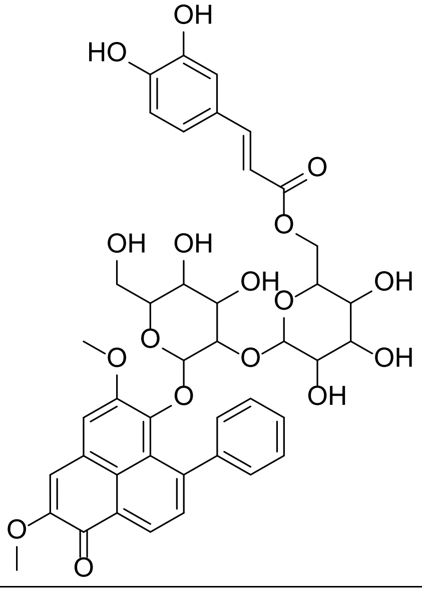	(Norman, Hombsch et al. 2021)

Database References

Brkljača, R. and S. Urban (2015). "HPLC-NMR and HPLC-MS Profiling and Bioassay-Guided Identification of Secondary Metabolites from the Australian Plant *Haemodorum spicatum*." Journal of Natural Products **78**(7): 1486-1494.

Brkljača, R., et al. (2015). "Phytochemical Investigation of the Constituents Derived from the Australian Plant *Macropidia fuliginosa*." Journal of Natural Products **78**(7): 1600-1608.

Carpinelli de Jesus, M., et al. (2023). "Differentiating Dyes: A Spectroscopic Investigation into the Composition of Scarlet Bloodroot (*Haemodorum coccineum* R.Br.) Rhizome." Molecules **28**(21): 7422.

Chen, Y., et al. (2016). "Phenylbenzoisoquinolindione alkaloids accumulate in stamens of *Xiphidium caeruleum* Aubl. flowers." Phytochemistry (Elsevier) **128**: 95-101.

Chen, Y., et al. (2017). "Cultured roots of *Xiphidium caeruleum*: Phenylphenalenones and their biosynthetic and extractant-dependent conversion." Phytochemistry (Elsevier) **133**: 15-25.

Chen, Y., et al. (2019). "Organ-specific distribution and non-enzymatic conversions indicate a metabolic network of phenylphenalenones in *Xiphidium caeruleum*." Phytochemistry (Elsevier) **159**: 30-38.

Cooke, R. G. and R. L. Thomas (1975). "Coloring matters of Australian plants. XVIII. Constituents of *Anigozanthos rufus*." Aust. J. Chem. **28**(5): 1053.

Dias, D. A., et al. (2009). "Phenylphenalenones from the Australian Plant *Haemodorum simplex*." J. Nat. Prod. **72**(6): 1075-1080.

Dong, L.-B., et al. (2011). "Chemical constituents from the aerial parts of *Musella lasiocarpa*." Nat. Prod. Bioprospect. **1**(1): 41-47.

Edwards, J. M. and U. Weiss (1974). "Pigments of *Lachnanthes tinctoria*. V. Phenalenone pigments of the root system of *Lachnanthes tinctoria*." Phytochemistry **13**(8): 1597.

Fang, J.-J., et al. (2011). "Phenylphenalenones and related natural products from *Wachendorfia thyrsiflora* L." Phytochem. Lett. **4**(2): 203-208.

Fang, J., et al. (2012). "Co-occurrence of phenylphenalenones and flavonoids in *Xiphidium caeruleum* Aubl. flowers." Phytochemistry (Elsevier) **82**: 143-148.

Fang, J., et al. (2012). "Phytochemical profile of aerial parts and roots of *Wachendorfia thyrsiflora* L. studied by LC-DAD-SPE-NMR." Phytochemistry (Elsevier) **81**: 144-152.

Hoelscher, D. and B. Schneider (2005). "The biosynthesis of 8-phenylphenalenones from *Eichhornia crassipes* involves a putative aryl migration step." Phytochemistry (Elsevier) **66**(1): 59-64.

Holscher, D. and B. Schneider (1997). "Phenylphenalenones from root cultures of Anigozanthos preissii." Phytochemistry **45**(1): 87-91.

Holscher, D. and B. Schneider (1998). "HPLC-NMR analysis of phenylphenalenones and a stilbene from *Anigozanthos flavidus*." Phytochemistry **50**(1): 155-161.

Holscher, D. and B. Schneider (1998). "Phenylphenalenones from *Ensete ventricosum*." Phytochemistry **49**(7): 2155-2157.

Liu, F., et al. (2014). "Diarylheptanoids and phenylphenalenones from Musa itinerans fruits." Phytochemistry (Elsevier) **103**: 171-177.

Morrison, G. A., et al. (1971). "Naturally occurring compounds related to phenalenone. I. Synthesis of lachnanthocarpone." J. Chem. Soc., C(1): 36-40.

Munde, T., et al. (2013). "Biosynthesis of tetraoxygenated phenylphenalenones in *Wachendorfia thyrsiflora*." Phytochemistry (Elsevier) **91**: 165-176.

Norman, E. O., et al. (2021). "Phytochemical Profiling and Biological Testing of the Constituents of the Australian Plant *Haemodorum brevisepalum*." Journal of Natural Products **84**(11): 2832-2844.

Norman, E. O., et al. (2019). "Distribution, biosynthesis, and biological activity of phenylphenalenone-type compounds derived from the family of plants, Haemodoraceae." Natural Product Reports **36**(5): 753-768.

Ocampos, F. M. M., et al. (2017). "Phytochemical profile of *Schiekia orinocensis* (Haemodoraceae)." Phytochem. Lett. **21**: 139-145.

Opitz, S., et al. (2002). "Phenylphenalenone-related compounds: Chemotaxonomic markers of the Haemodoraceae from *Xiphidium caeruleum*." J. Nat. Prod. **65**(8): 1122-1130.

Opitz, S., et al. (2002). "Isomeric oxabenzochrysenones from *Musa acuminata* and *Wachendorfia thyrsiflora*." Nat. Prod. Lett. **16**(5): 335-338.

Opitz, S. and B. Schneider (2002). "Organ-specific analysis of phenylphenalenone-related compounds in *Xiphidium caeruleum*." Phytochemistry (Elsevier) **61**(7): 819-825.

Opitz, S., et al. (2003). "Histochemical analysis of phenylphenalenone-related compounds in *Xiphidium caeruleum* (haemodoraceae)." Planta **216**(5): 881-889.

Schneider, B., et al. (2005). "HPLC-NMR for tissue-specific analysis of phenylphenalenone-related compounds in *Xiphidium caeruleum* (Haemodoraceae)." Magn. Reson. Chem. **43**(9): 724-728.

Urban, S., et al. (2013). "Phenylphenalenones and oxabenzochrysenones from the Australian plant *Haemodorum simulans*." Phytochemistry (Elsevier) **95**: 351-359.

S2. Annotated expansion of UV chromatogram (254 nm) (t_R = 6-24 min) of ethanolic extract of *Haemodorum simulans* bulbs (2005_01a)

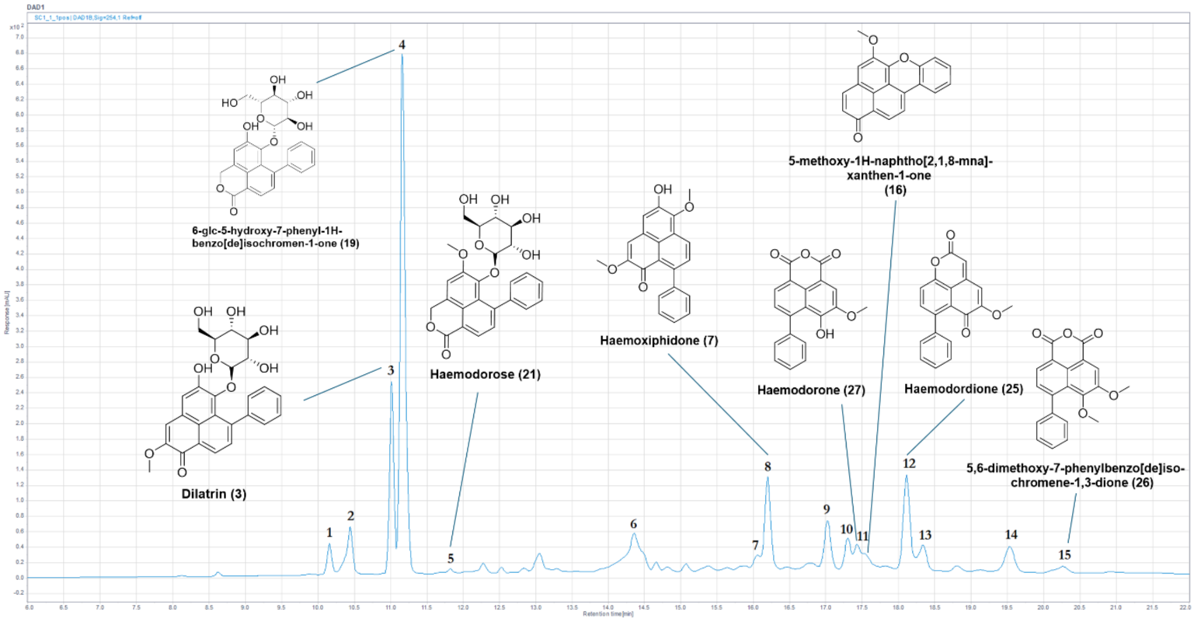

S3. Annotated expansion of UV chromatogram (254 nm) (t_R = 6-24 min) of ethanolic extract of Haemodorum simulans stems (2005_01b)

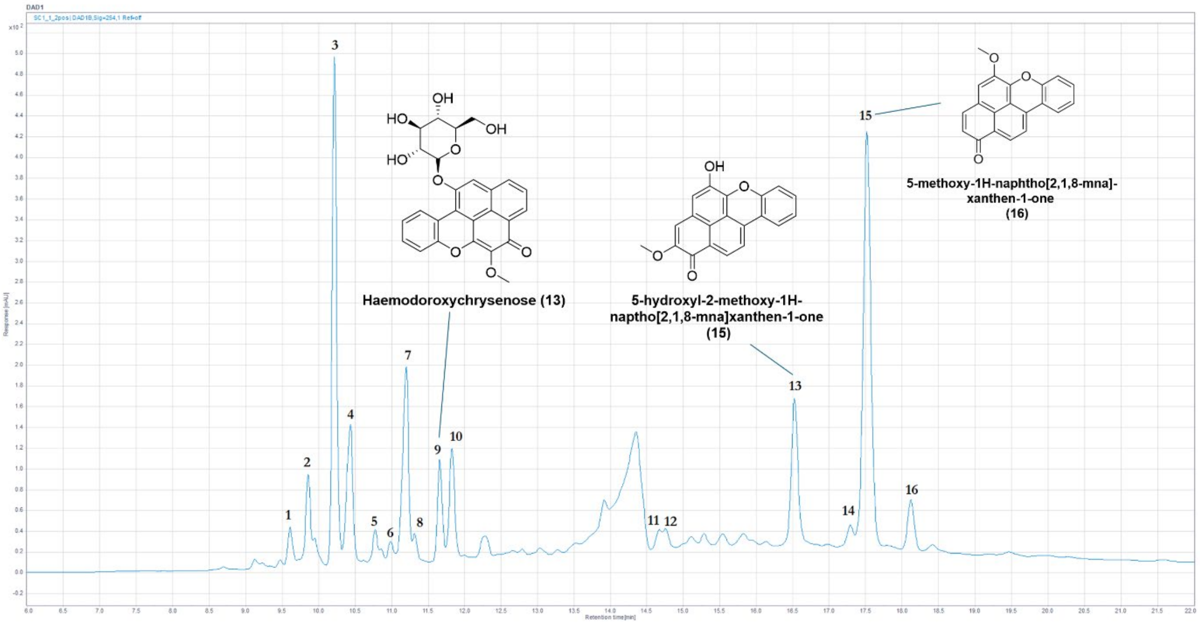

S4. Annotated expansion of UV chromatogram (254 nm) (t_R = 6-24 min) of ethanolic extract of *Haemodorum simulans* bulbs (2007_01a)

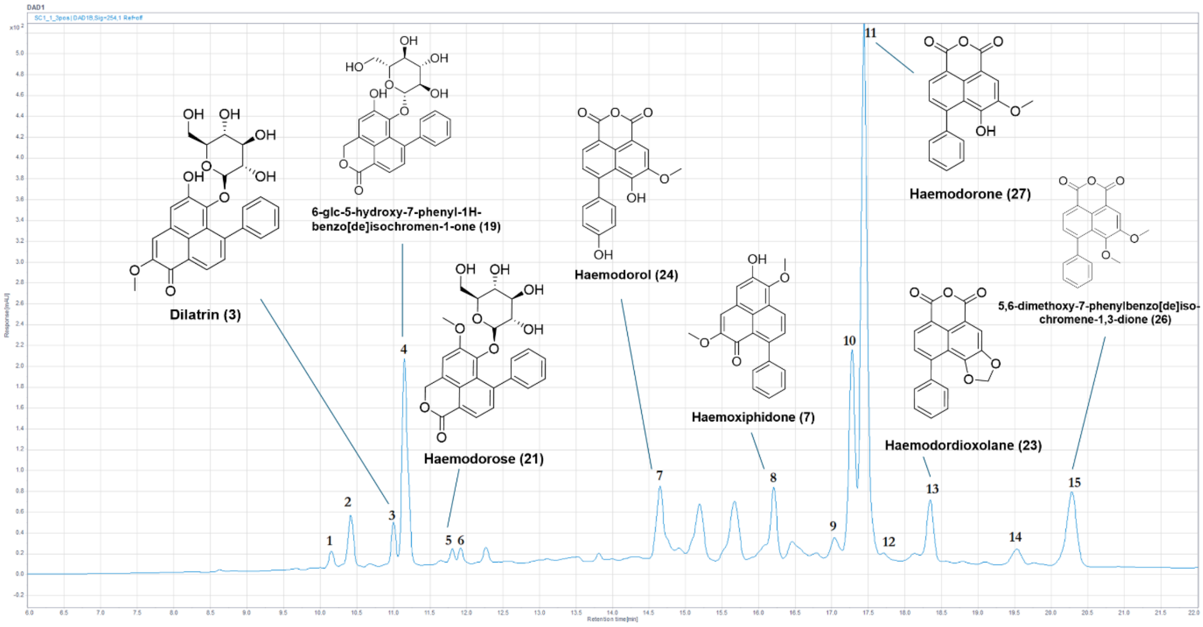

S5. Annotated expansion of UV chromatogram (254 nm) (t_R = 6-24 min) of ethanolic extract of *Haemodorum simulans* stems (2007_01b)

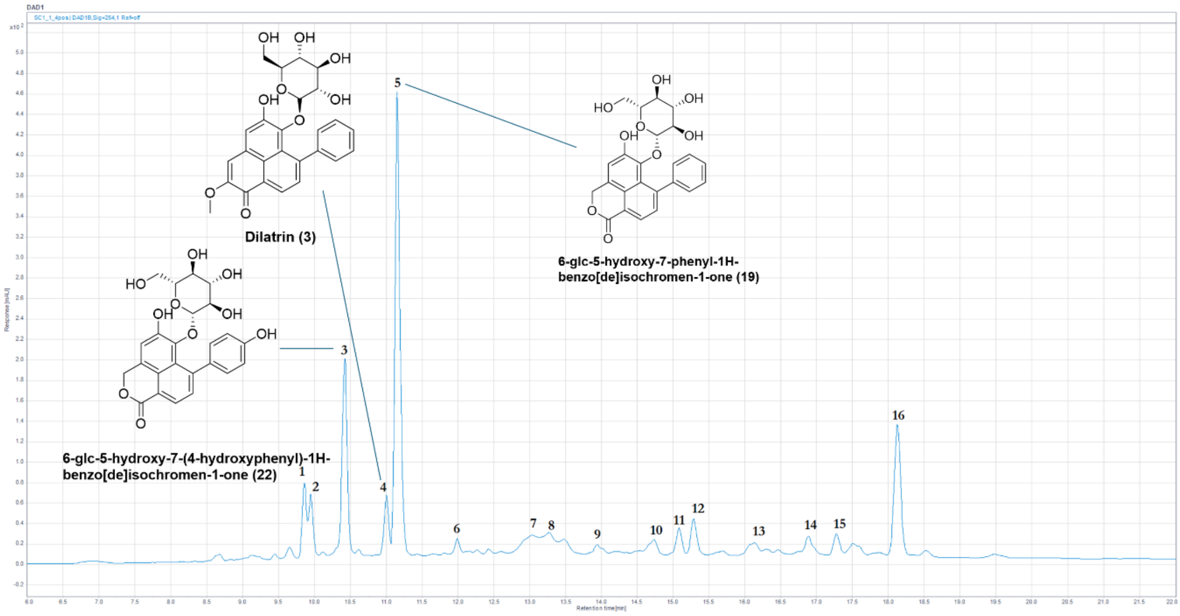

S6. Annotated expansion of UV chromatogram (254 nm) (t_R = 6-24 min) of ethanolic extract of *Haemodorum simulans* bulbs (2010_17a)

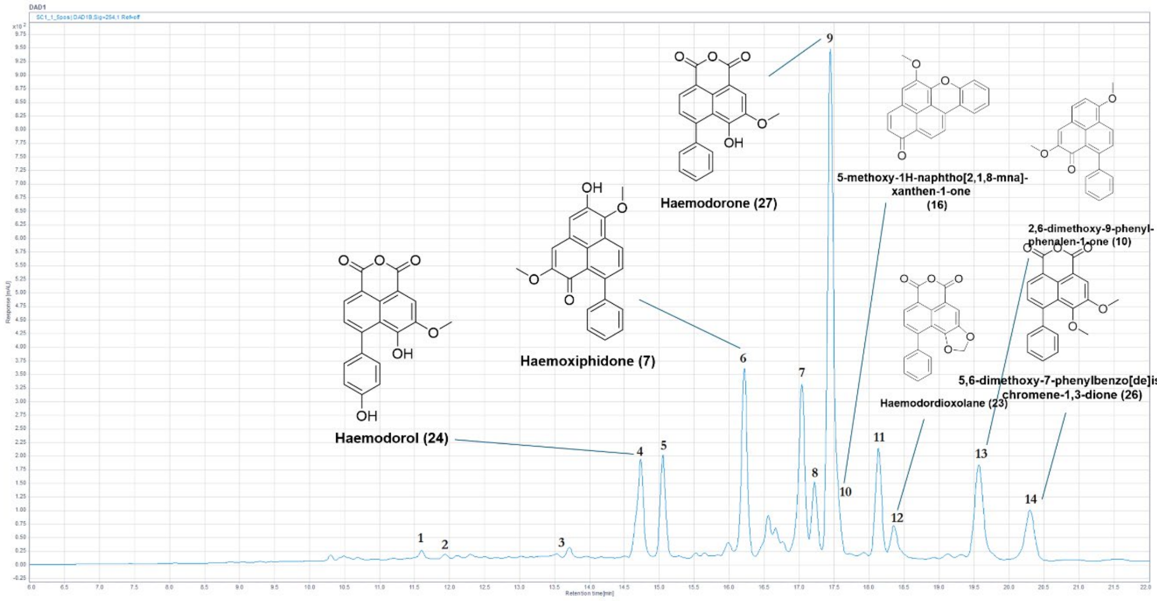

S7. Annotated expansion of UV chromatogram (254 nm) (t_R = 6-24 min) of ethanolic extract of *Haemodorum simulans* stems (2010_17b)

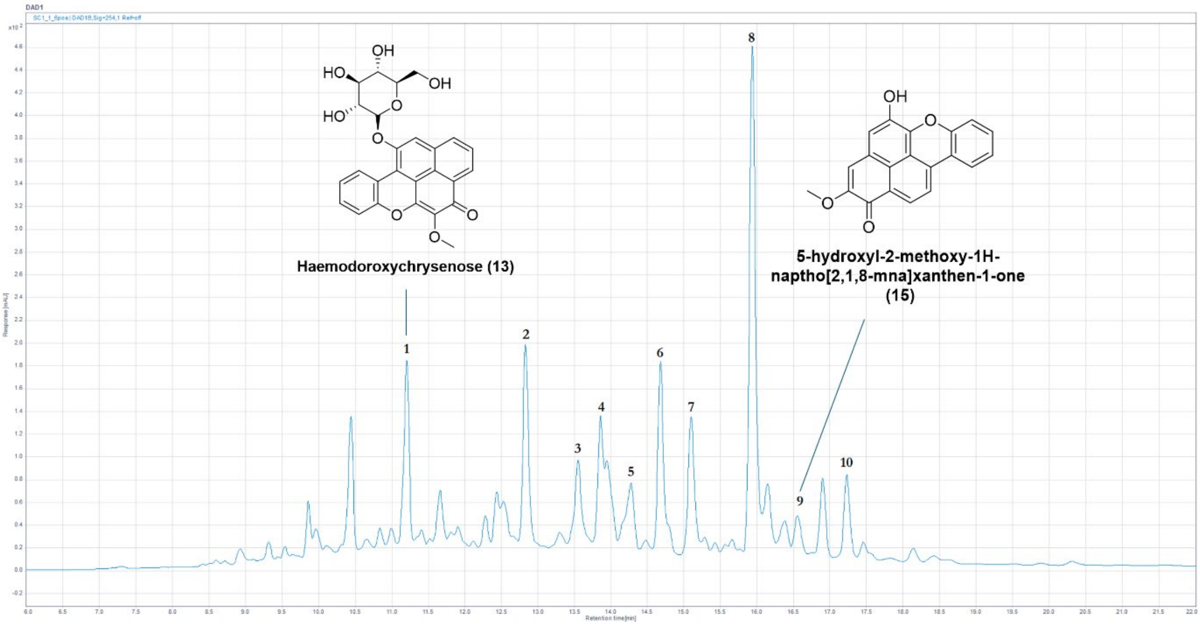

S8. Annotated expansion of UV chromatogram (254 nm) (t_R = 6-24 min) of ethanolic extract of *Haemodorum brevisepalum* bulbs (2010_19a)

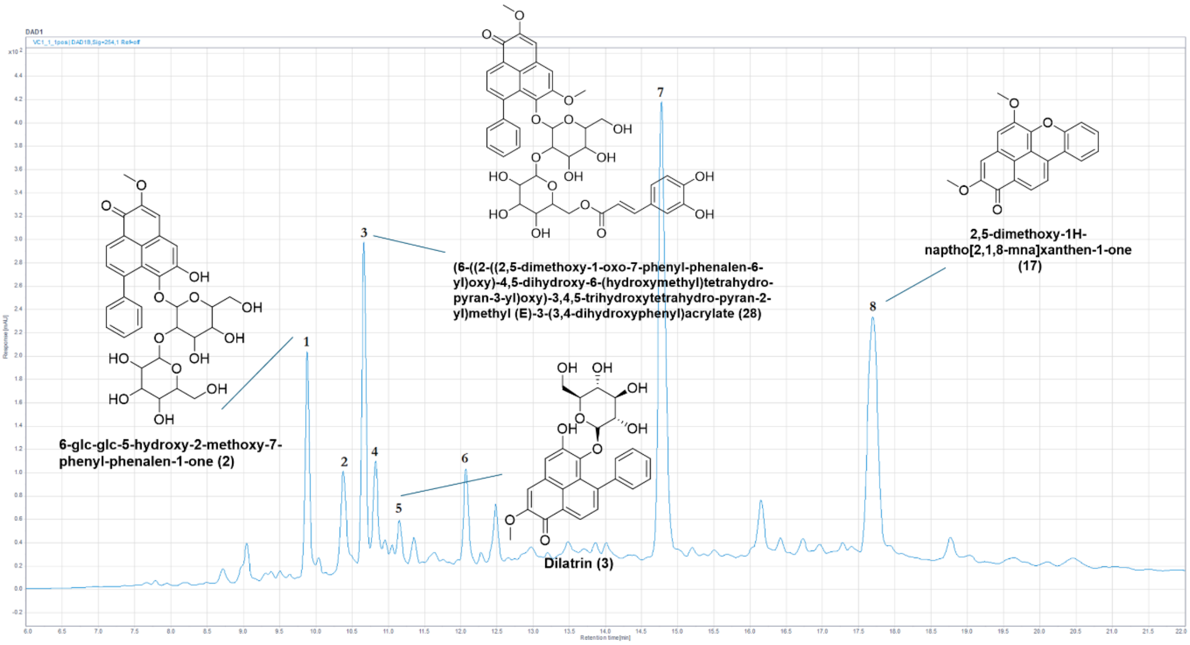

S9. Annotated expansion of UV chromatogram (254 nm) (t_R = 6-24 min) of ethanolic extract of *Haemodorum brevisepalum* stems (2010_19b)

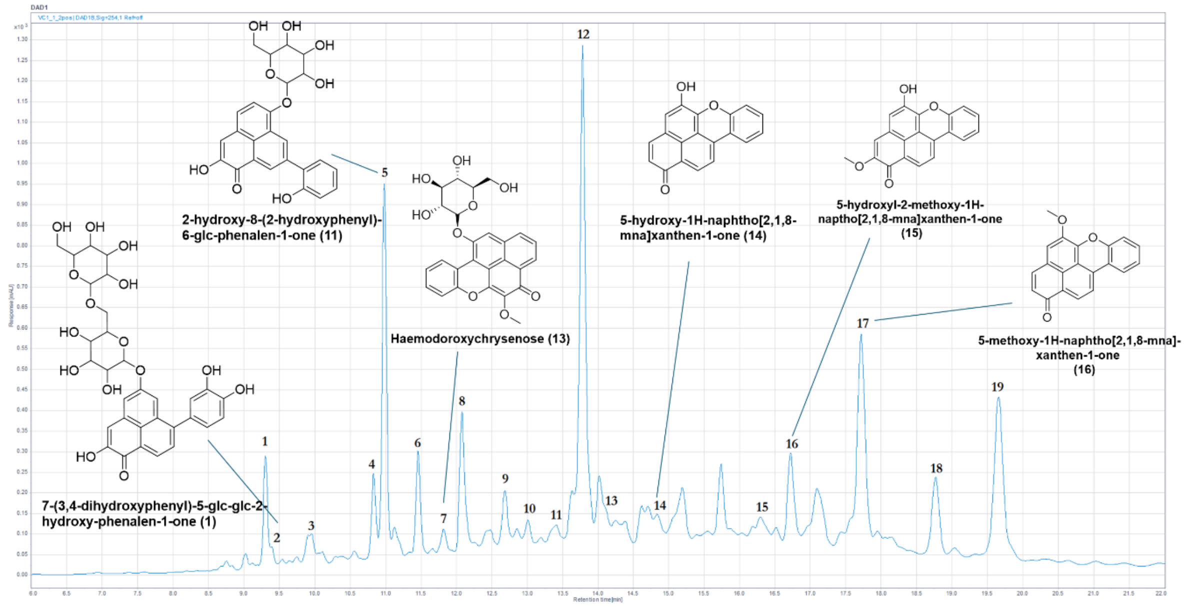

S10. Annotated expansion of UV chromatogram (254 nm) (t_R = 6-24 min) of ethanolic extract of *Haemodorum spicatum* bulbs (2010_20a)

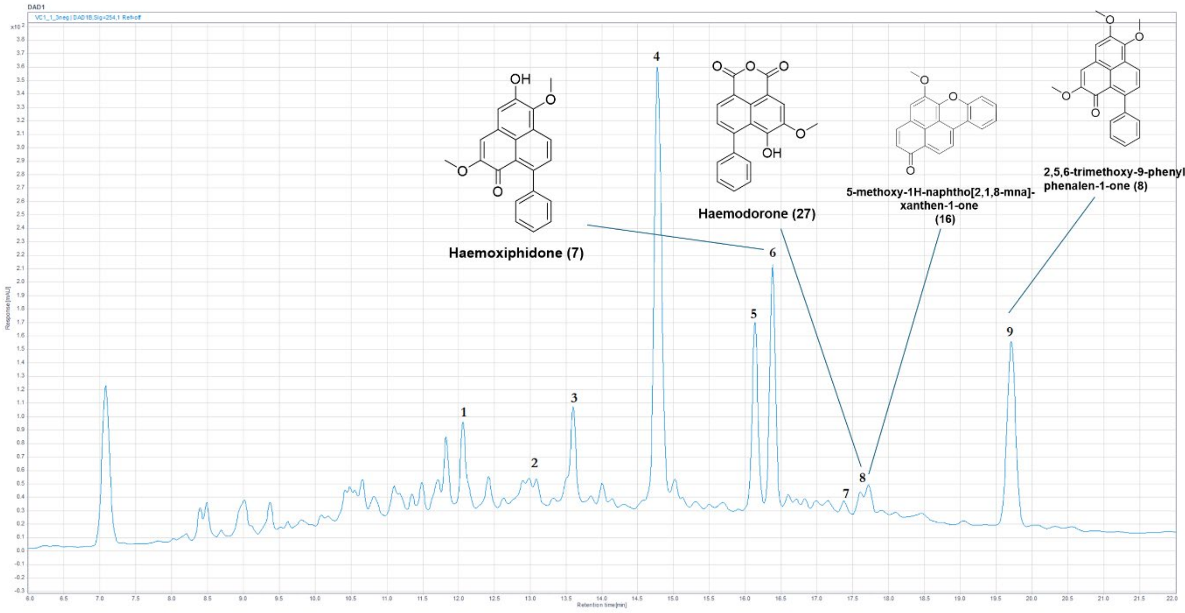

S11. Annotated expansion of UV chromatogram (254 nm) (t_R = 6-24 min) of ethanolic extract of *Haemodorum spicatum* stems (2010_20b)

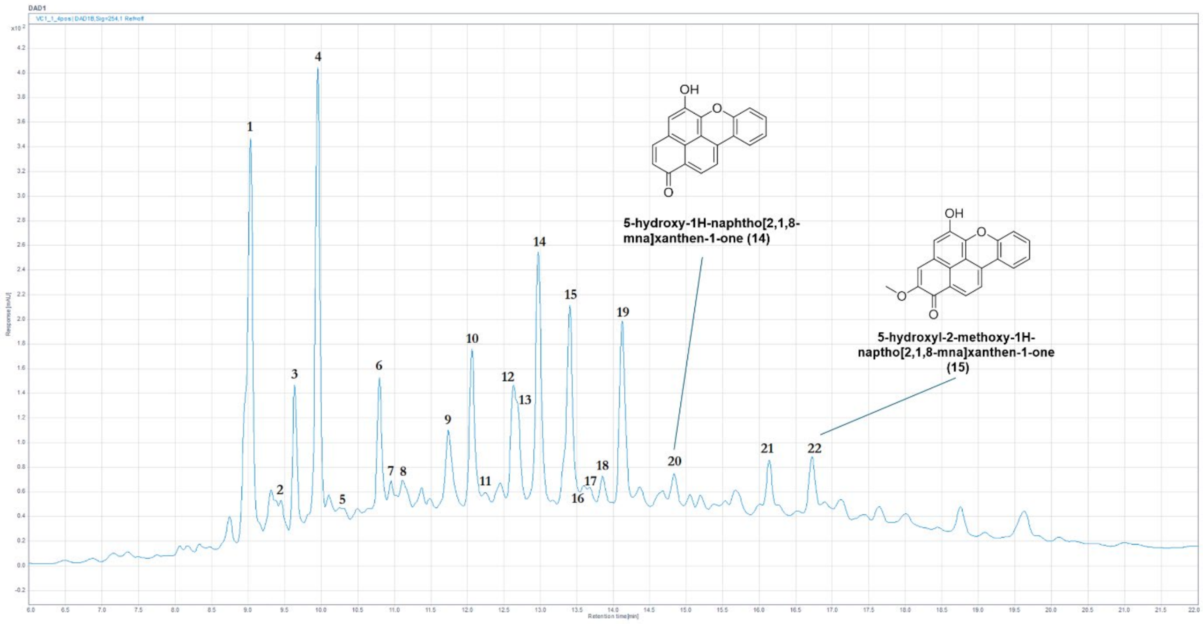

S12. Annotated expansion of UV chromatogram (254 nm) (t_R = 6-24 min) of ethanolic extract of *Macropidia fuliginosa* bulbs (2011_01a)

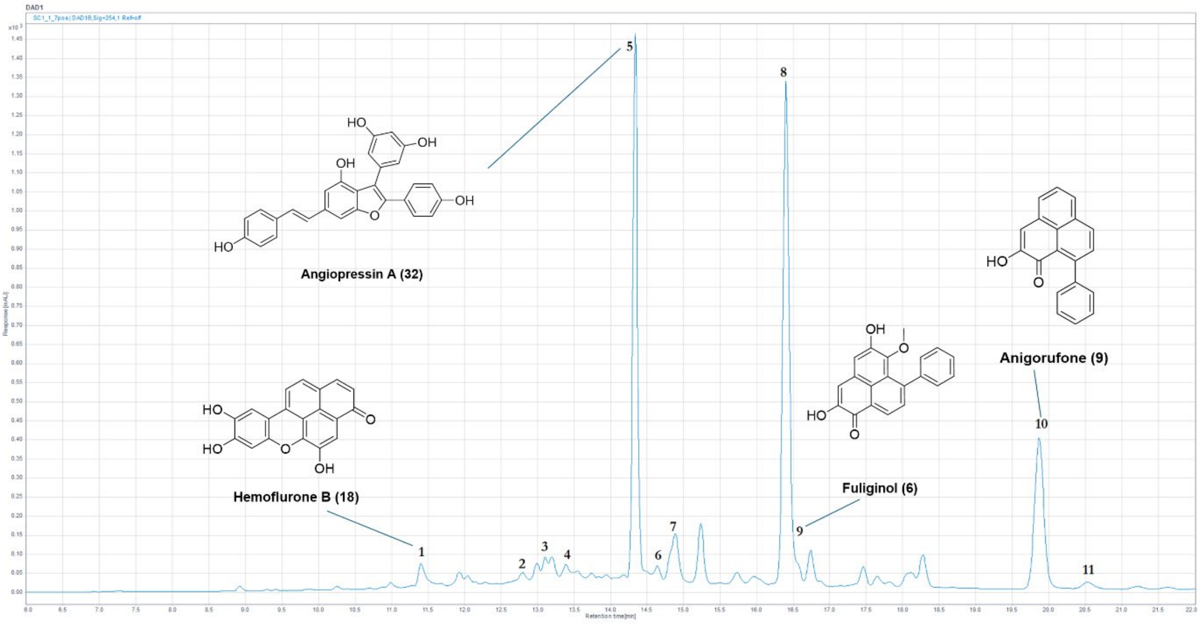

S13. Annotated expansion of UV chromatogram (254 nm) (t_R = 6-24 min) of ethanolic extract of *Macropidia fuliginosa* bulbs (2011_02a)

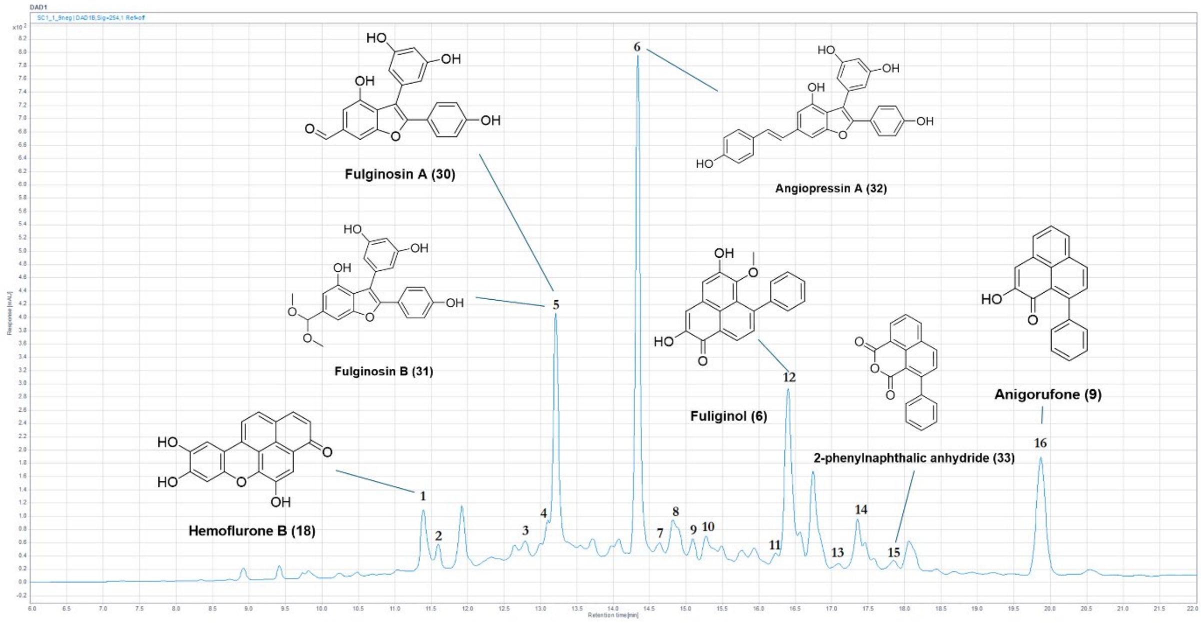

S14. Annotated expansion of UV chromatogram (254 nm) (t_R = 6-24 min) of ethanolic extract of *Macropidia fuliginosa* bulbs (2012_01a)

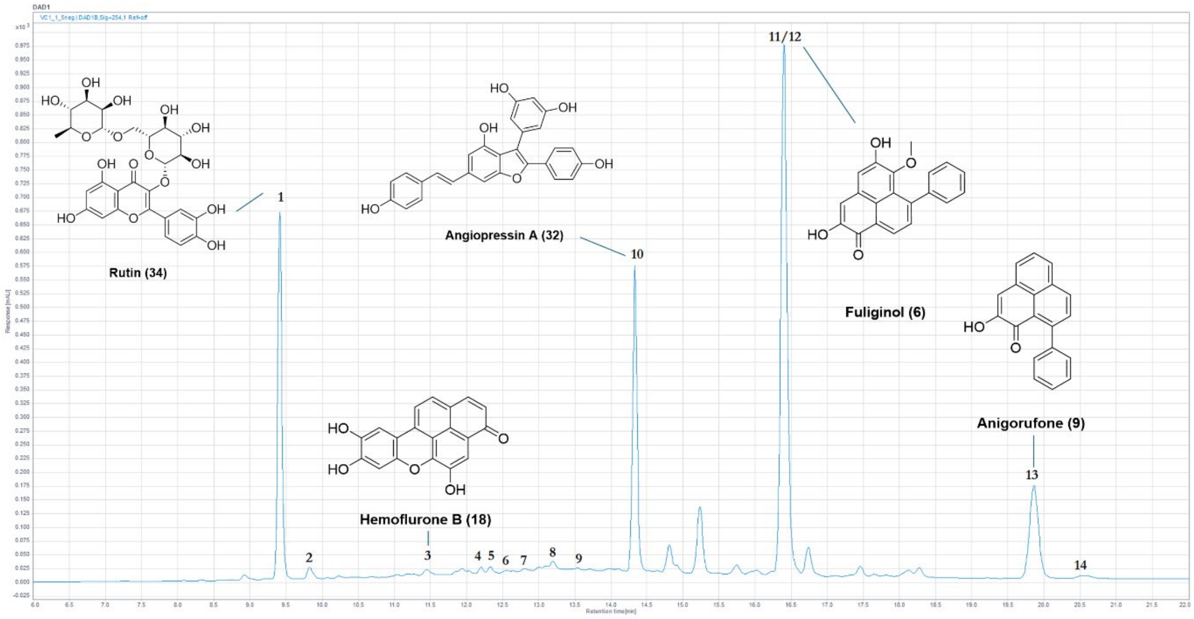

S15. Annotated composite expansion of UV chromatograms (254 nm) (t_R = 6-24 min) of ethanolic extracts of *Macropidia fuliginosa* stems/leaves (2011_01b, 2011_02b, 2012_01b, 2012_05b)

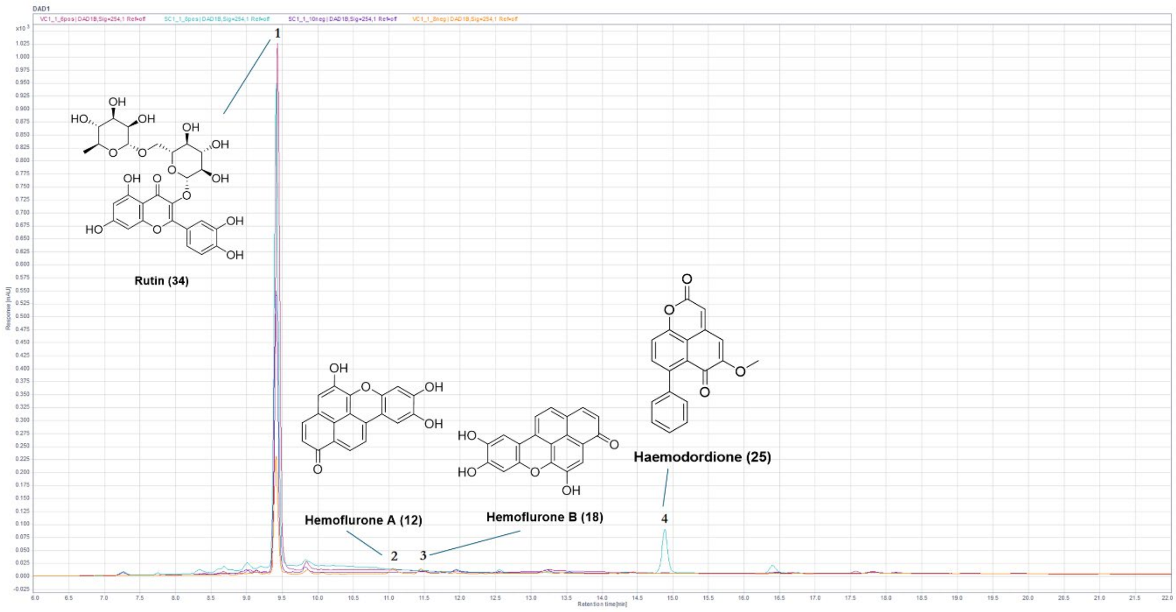

S16. Annotated expansion of UV chromatograms (254 nm) (t_R = 6-24 min) of ethanolic extract of *Macropidia fuliginosa* stems (2012_05c)

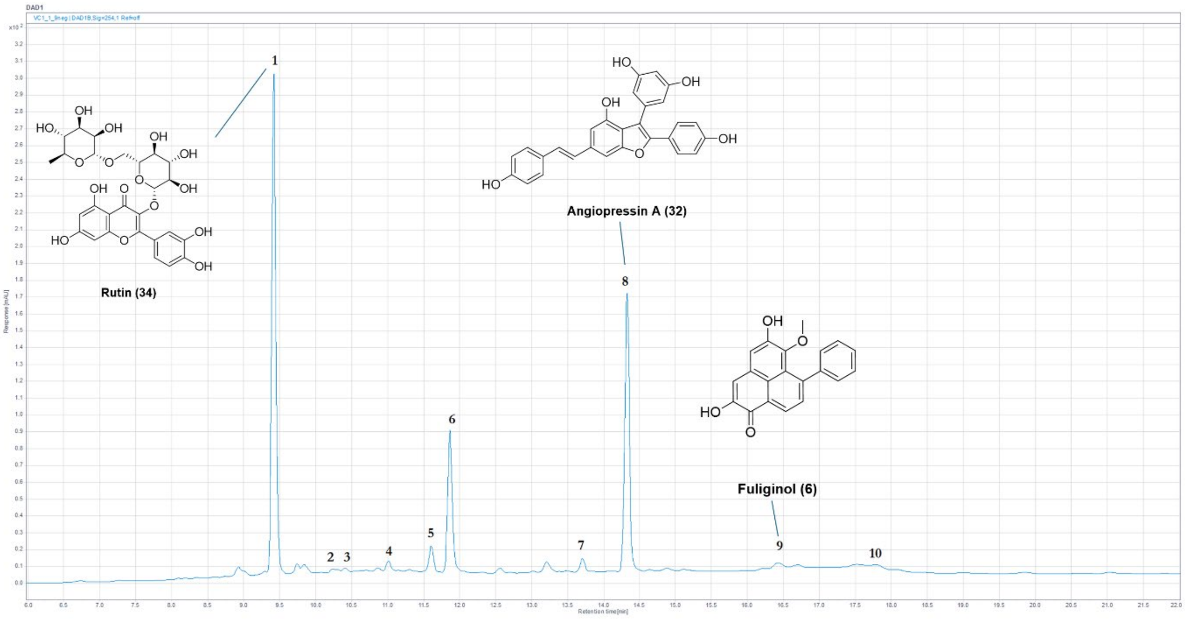

S17. Annotated composite expansion of UV chromatograms (254 nm) (t_R = 6-24 min) of ethanolic extracts of *Macropidia fuliginosa* flowers (2012_05a, 2013_02)

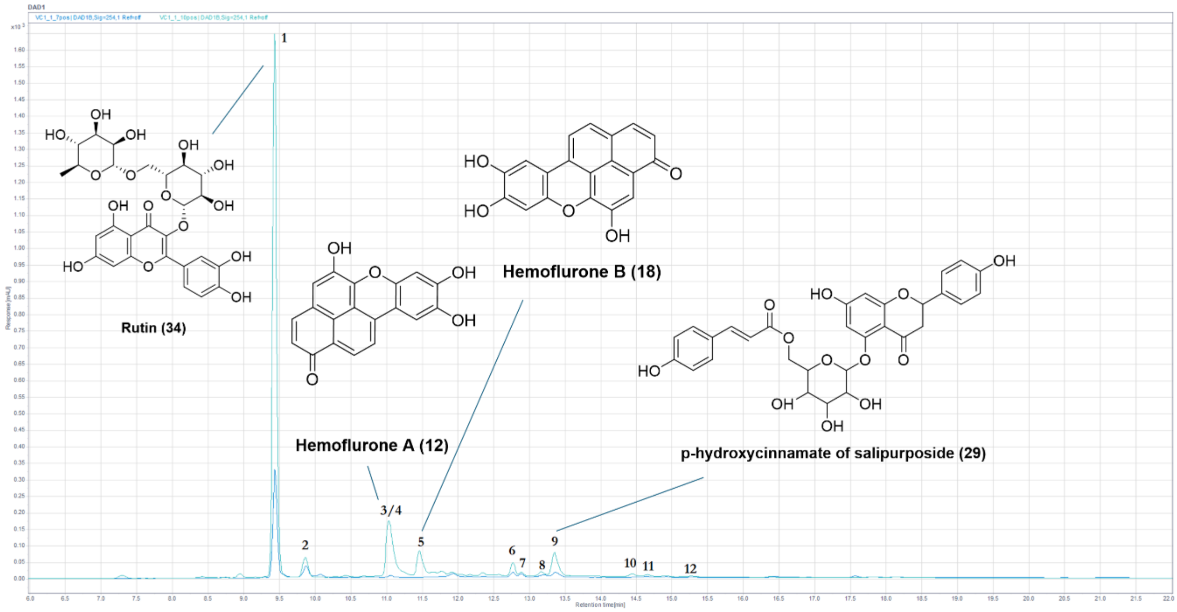

S18. Annotated expansion of UV chromatogram (254 nm) (t_R = 6-24 min) of ethanolic extract of *Haemodorum coccineum* leaves/stems (2021_17a)

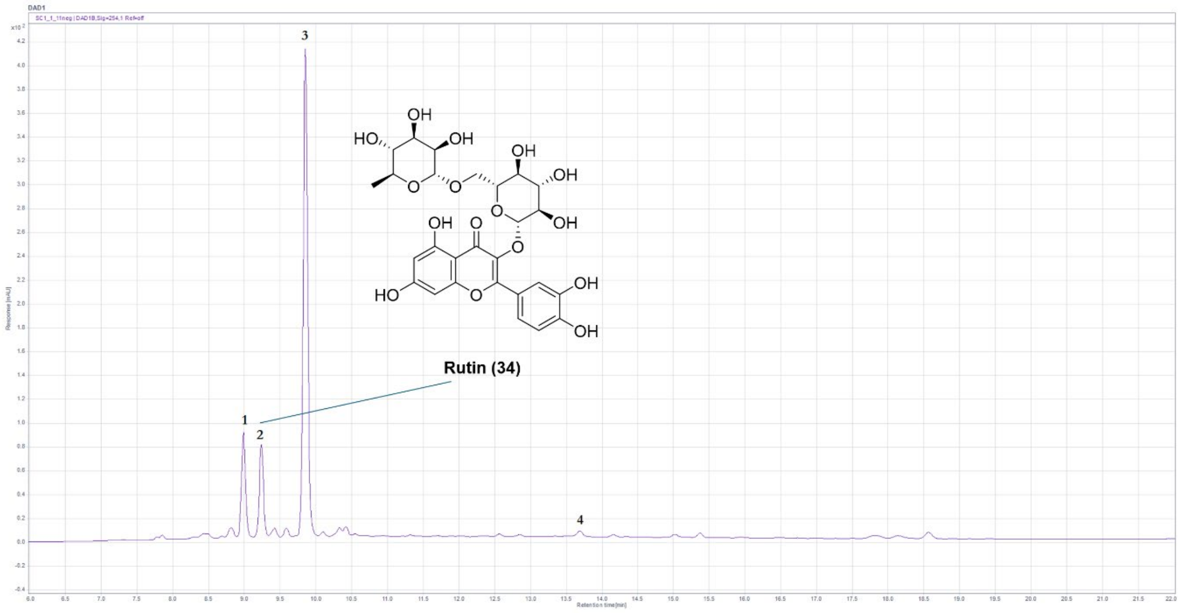

S19. Annotated expansion of UV chromatogram (254 nm) (t_R = 6-24 min) of ethanolic extract of *Haemodorum coccineum* roots (2021_17b)

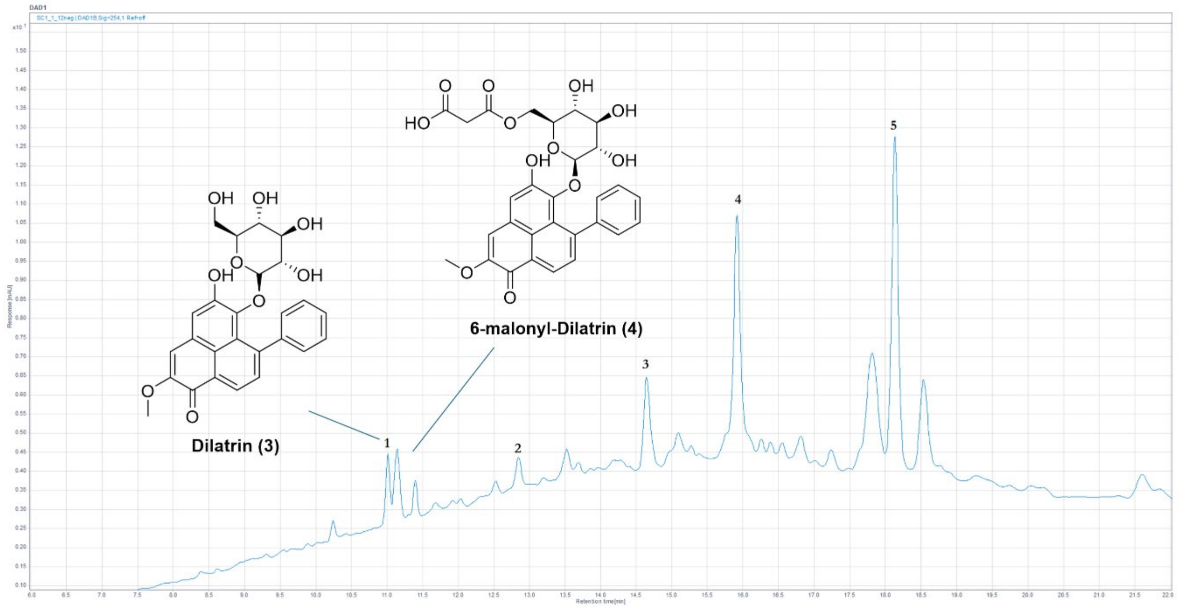

S20. Annotated expansion of UV chromatogram (254 nm) (t_R = 6-24 min) of ethanolic extract of *Haemodorum coccineum* leaves/bulbs (2022_08)

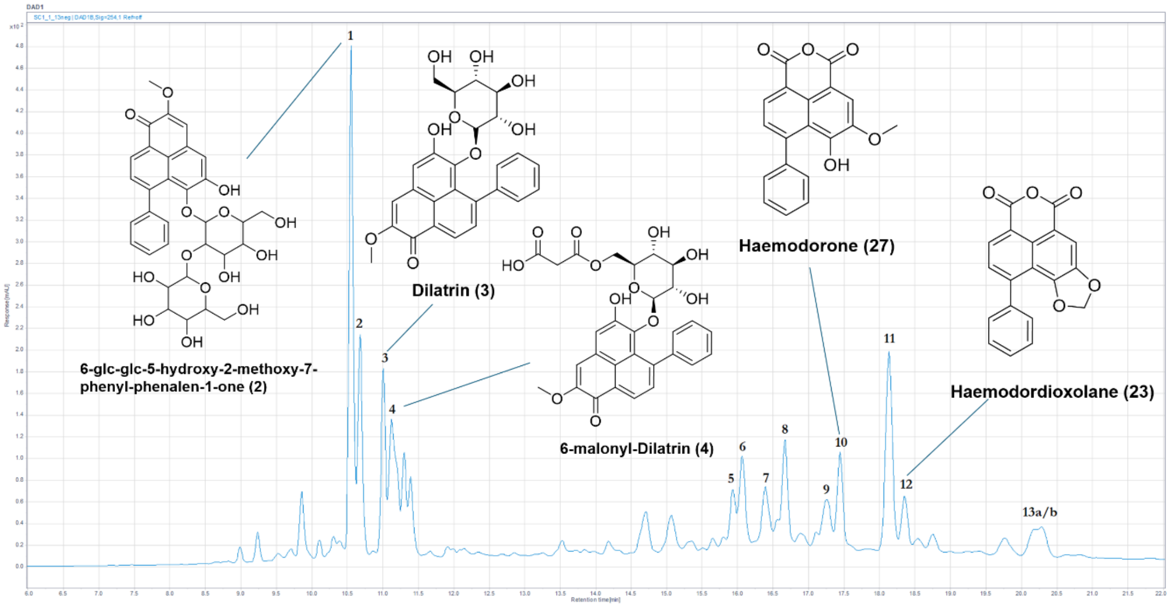

S21. Annotated expansion of UV chromatogram (254 nm) (t_R = 6-24 min) of ethanolic extract of *Haemodorum coccineum* bulbs (2023_01a)

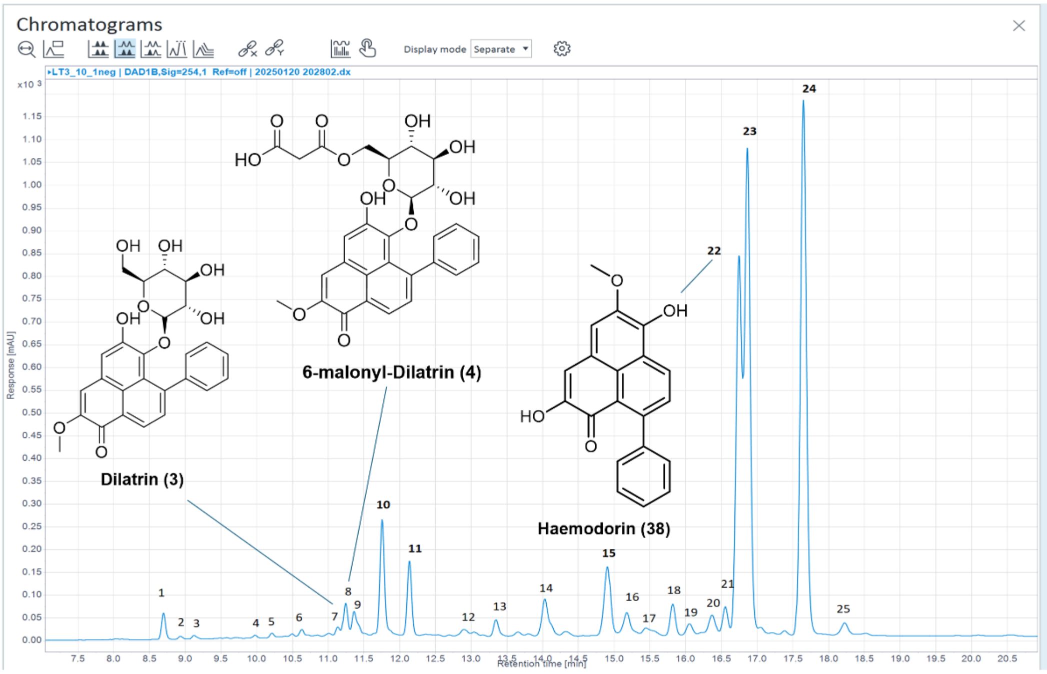

S22. Annotated expansion of UV chromatogram (254 nm) (t_R = 6-24 min) of ethanolic extract of *Haemodorum coccineum* stems (2023_01b)

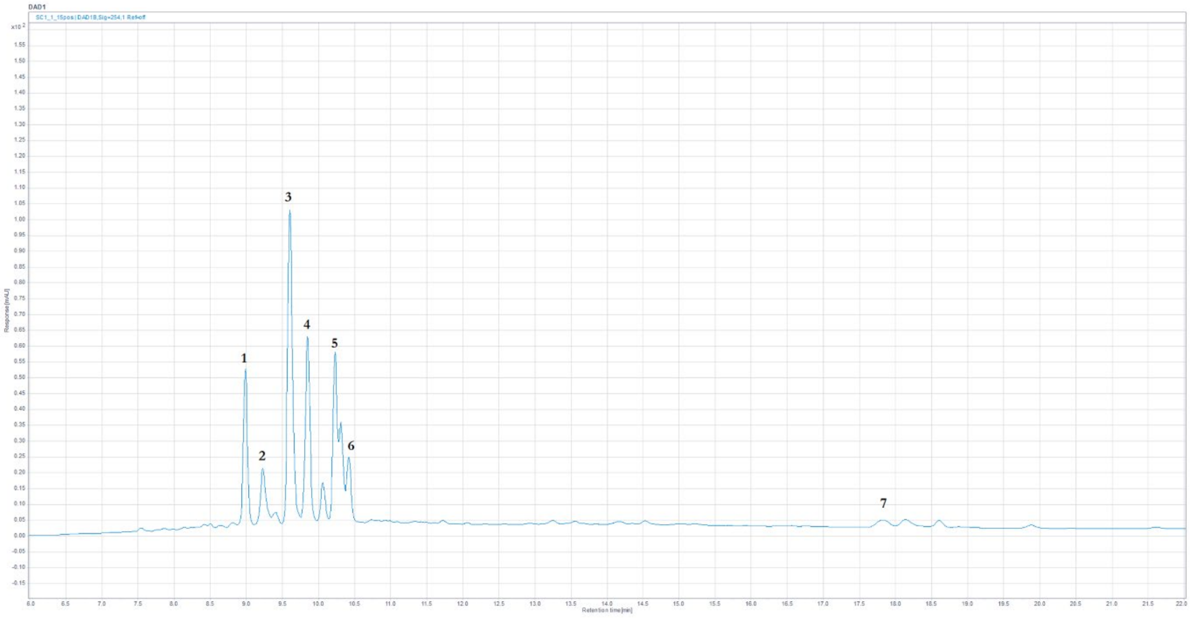

S23. Annotated expansion of UV chromatogram (254 nm) (t_R = 6-24 min) of ethanolic extract of *Haemodorum distichophyllum* leaves (2021_18a)

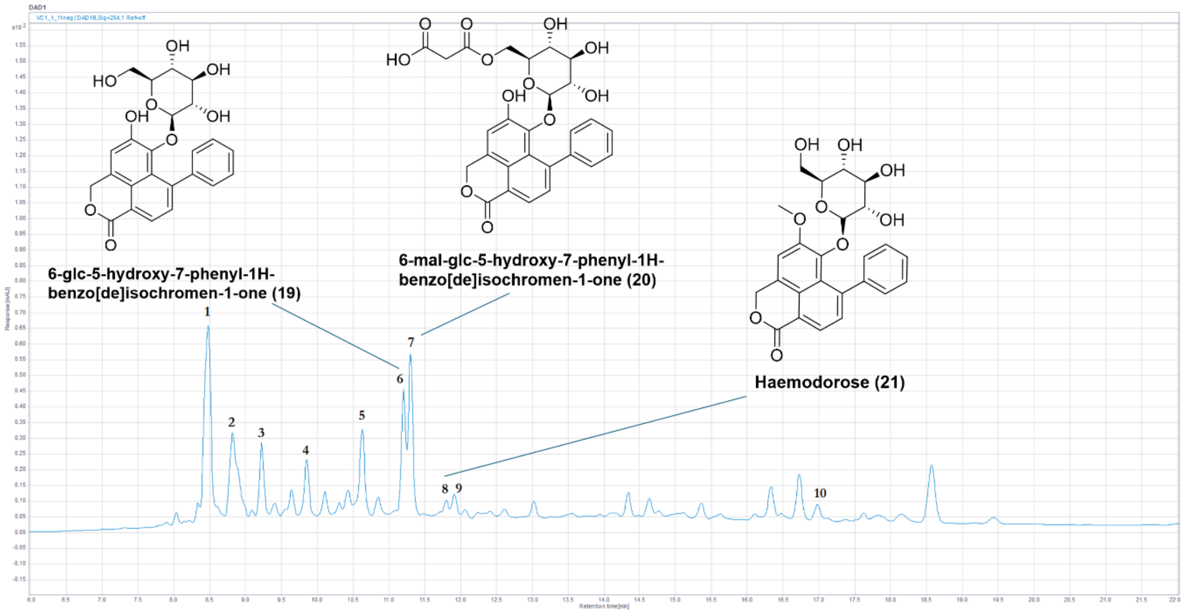

S24. Annotated expansion of UV chromatogram (254 nm) (t_R = 6-24 min) of ethanolic extract of *Haemodorum distichophyllum* flowers/seeds (2021_18b)

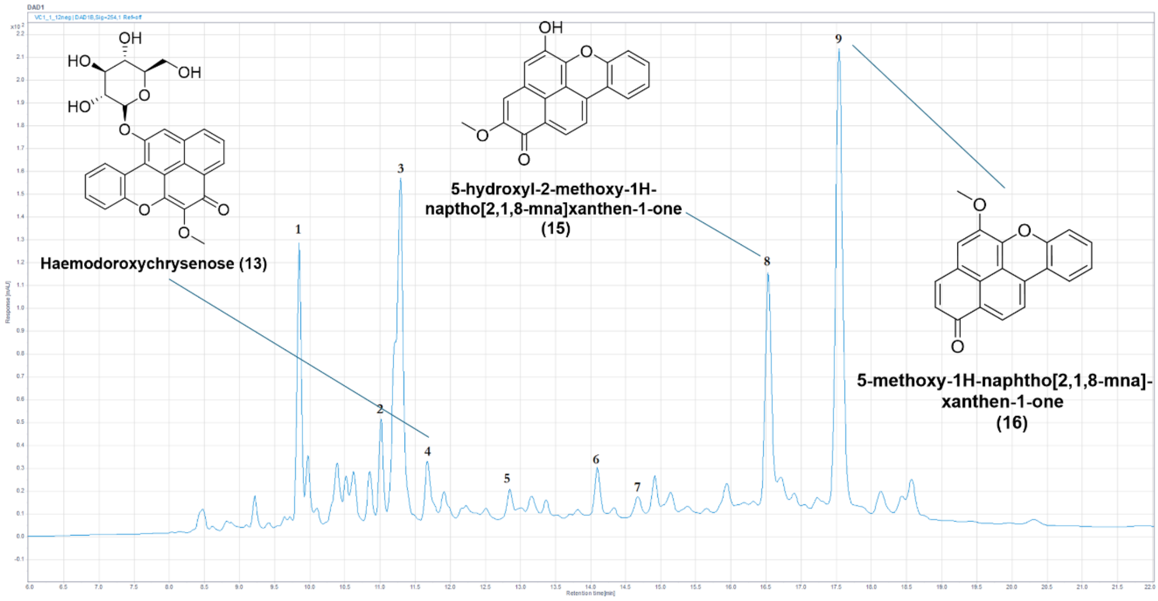

S25. Annotated expansion of UV chromatogram (254 nm) (t_R = 6-24 min) of ethanolic extract of *Haemodorum distichophyllum* roots (2021_18c)

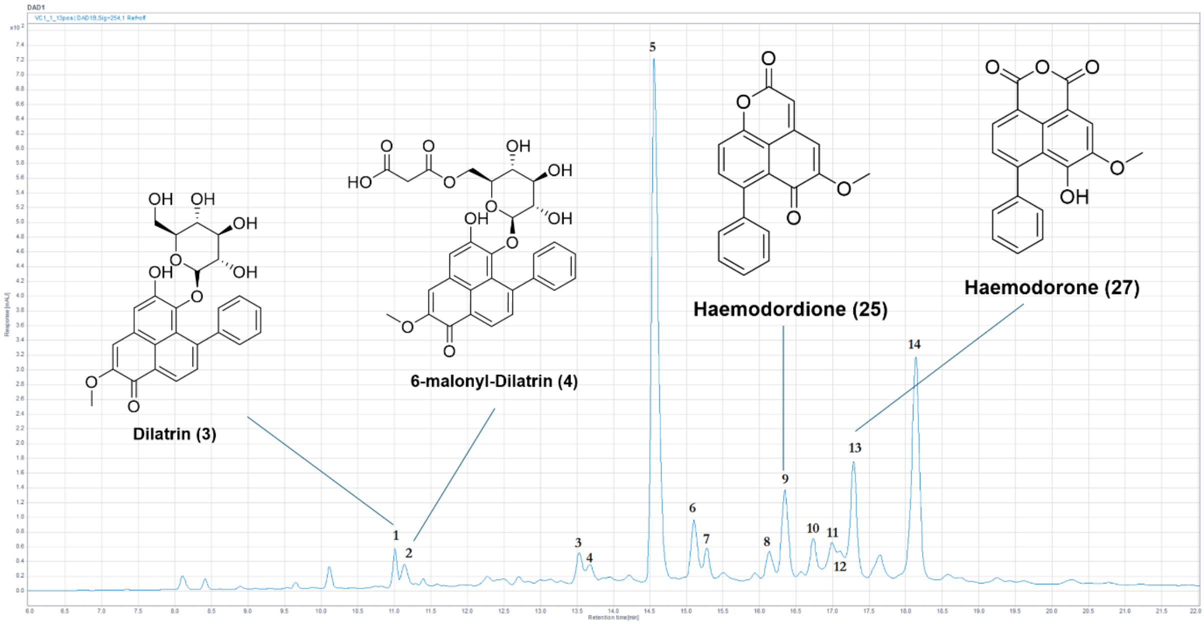

S26. Annotated expansion of UV chromatogram (254 nm) (t_R = 6-24 min) of ethanolic extract of *Haemodorum distichophyllum* leaves (2022_07a)

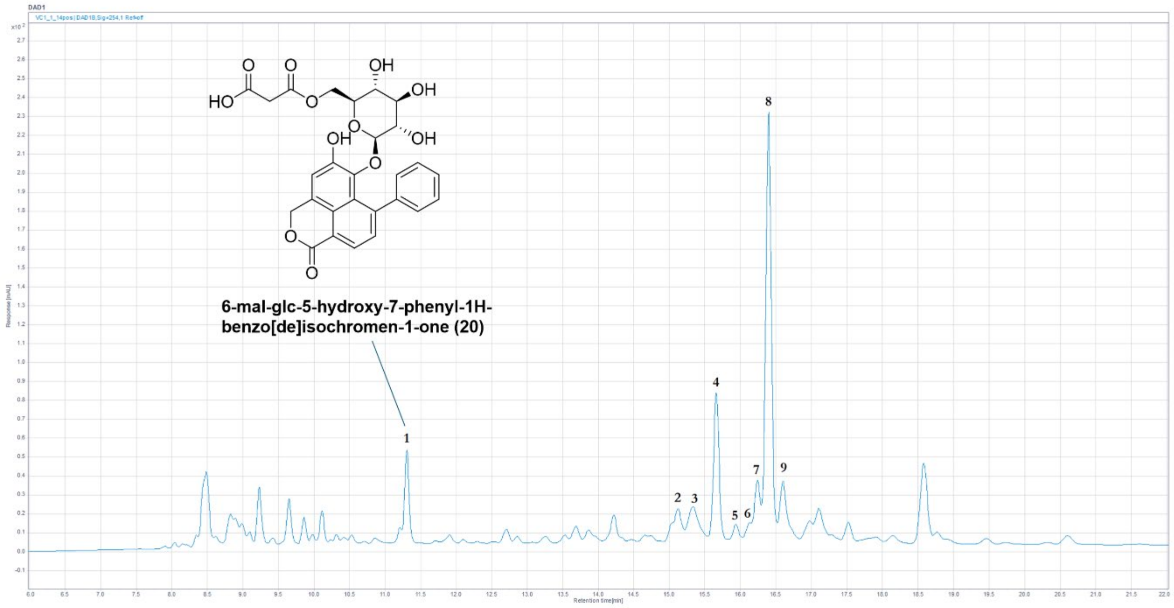

S27. Annotated expansion of UV chromatogram (254 nm) (t_R = 6-24 min) of ethanolic extract of *Haemodorum distichophyllum* roots/bulbs (2022_07b)

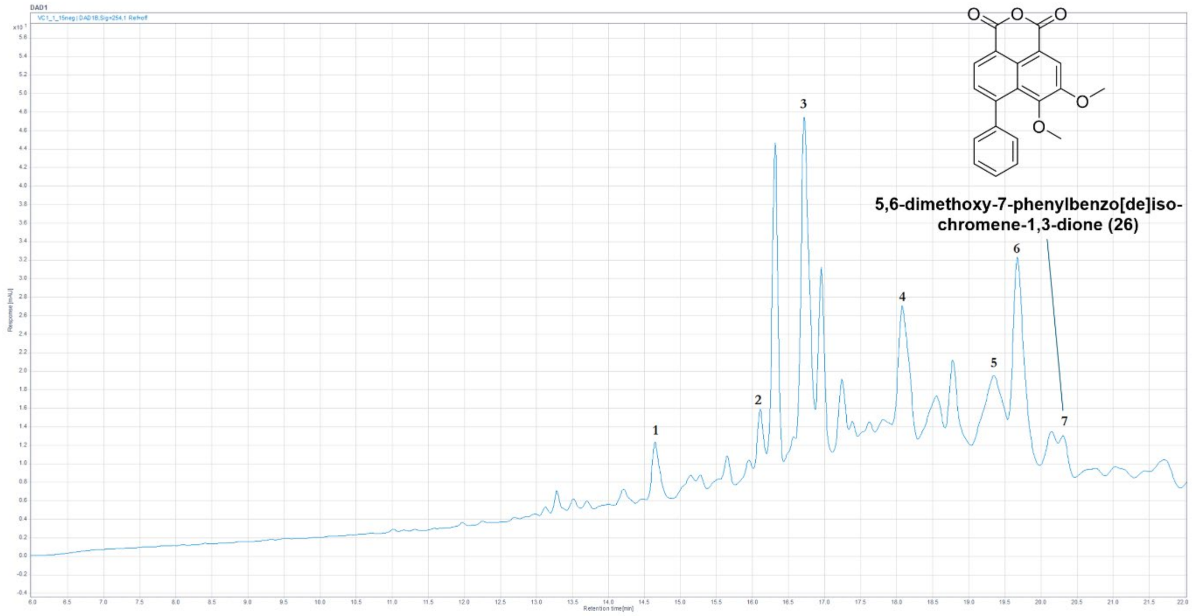

S28. Profiling data (UV chromatogram and ESI-MS) of **(1)** 7-(3,4-dihydroxyphenyl)-5-((3,4,5-trihydroxy-6-(((3,4,5-trihydroxy-6-(hydroxymethyl)tetrahydro-pyran-2-yl)oxy)methyl)-tetrahydro-pyran-2-yl)oxy)-2-hydroxy-phenalen-1-one

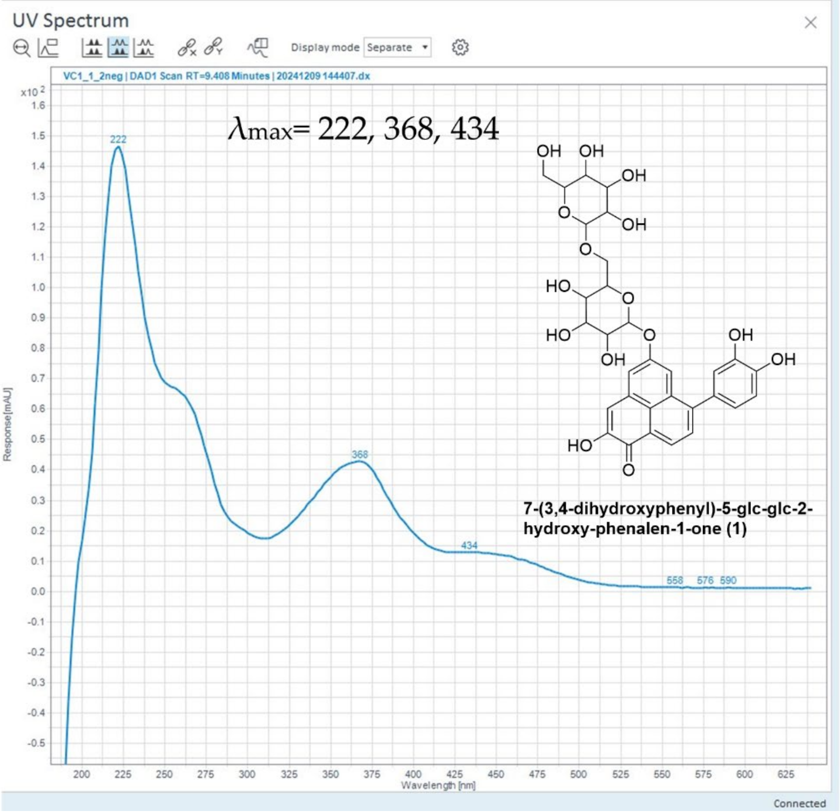

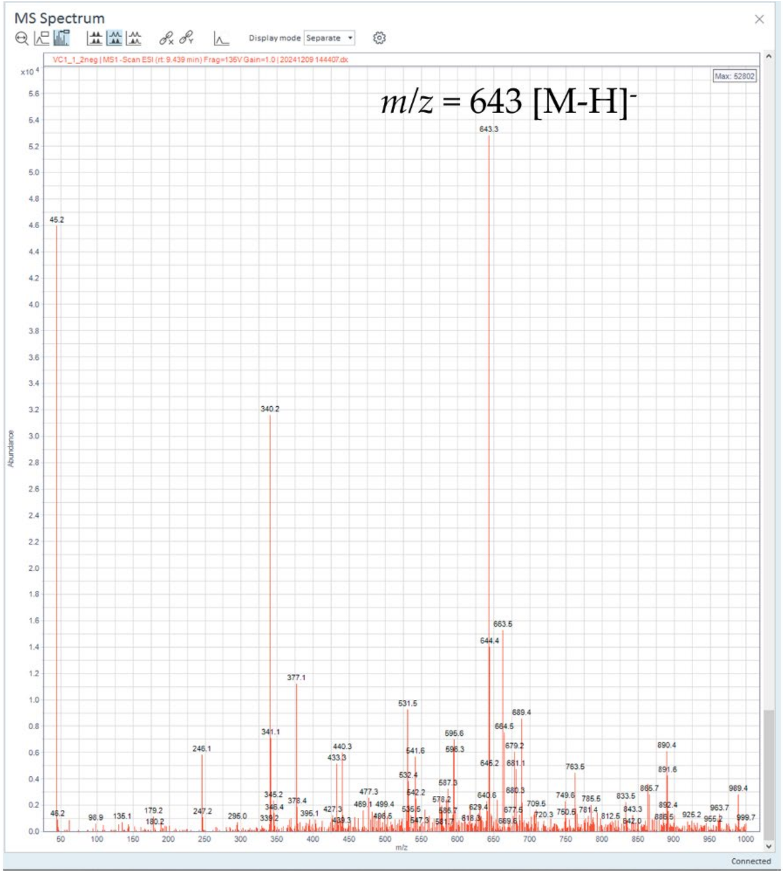

S29. Profiling data (UV chromatogram and ESI-MS) of **(2)** 6-((4,5-dihydroxy-6-(hydroxymethyl)-3-((3,4,5-trihydroxy-6-(hydroxymethyl)tetrahydro-pyran-2-yl)oxy)tetrahydro-pyran-2-yl)oxy)-5-hydroxy-2-methoxy-7-phenyl-1H-phenalen-1-one

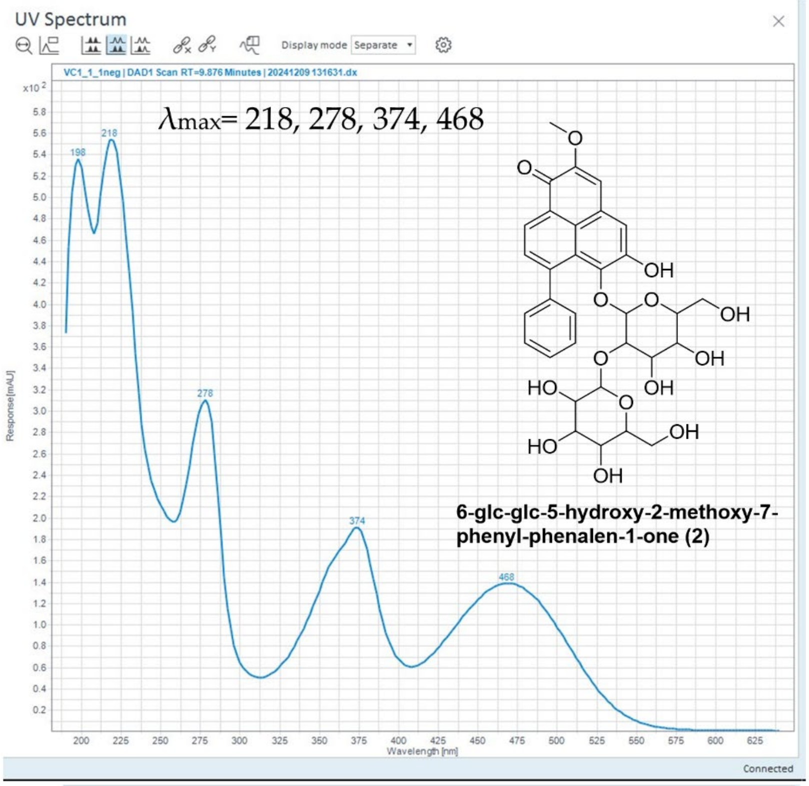

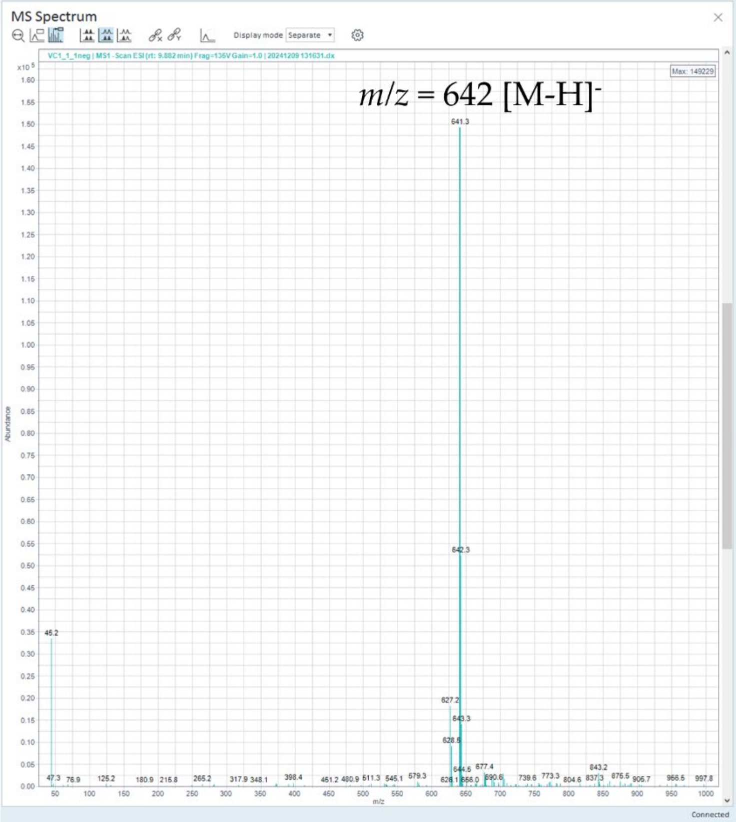

S30. Profiling data (UV chromatogram and ESI-MS) of **(3)** Dilatrin

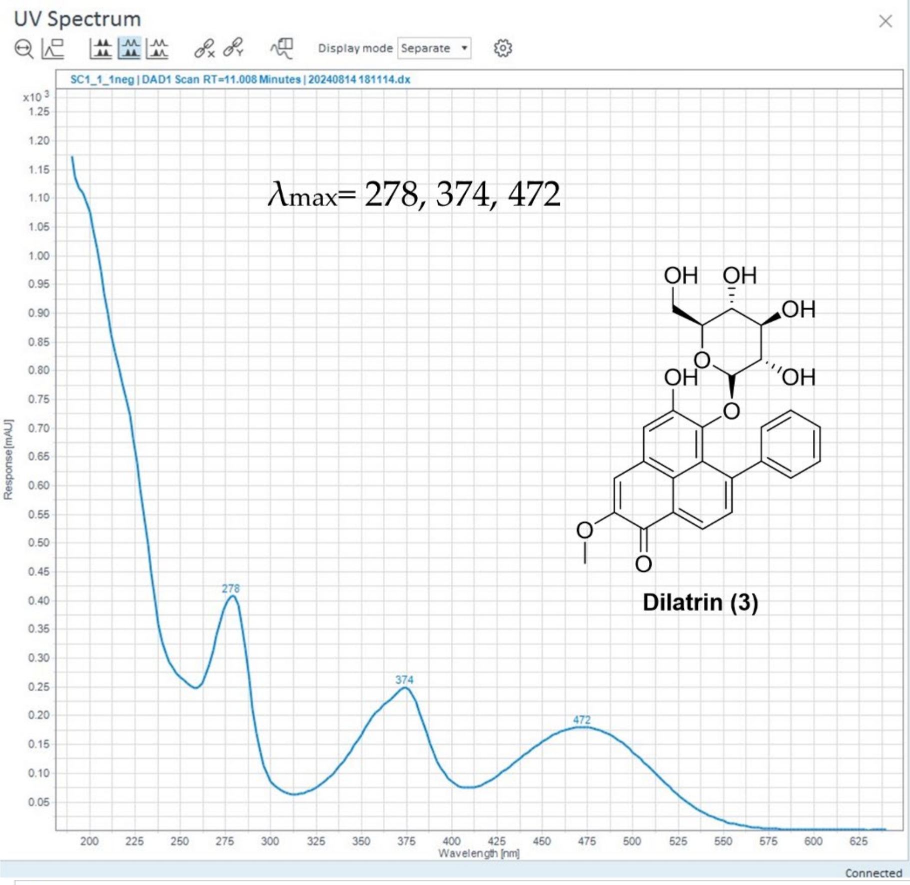

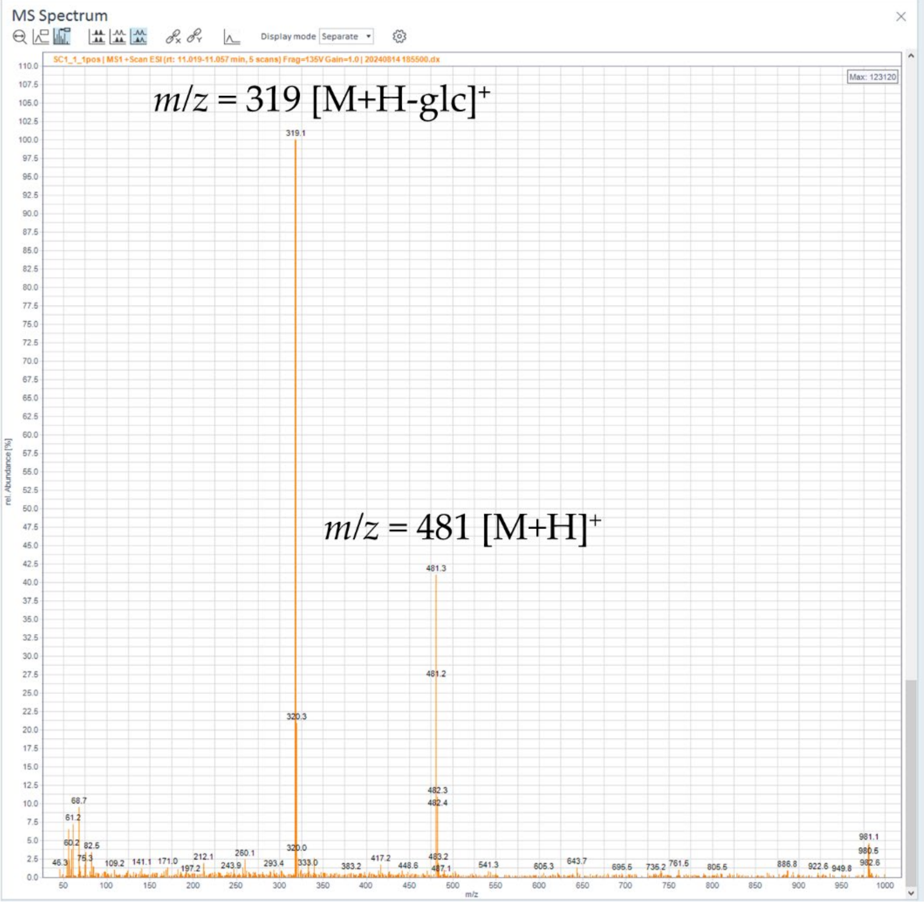

S31. Profiling data (UV chromatogram and ESI-MS) of **(4)** 6-O-[(6″-O-malonyl)-β-D-glucopyranosyl]-5-hydroxy-2-methoxy-7-phenyl-1H-phenalen-1-one

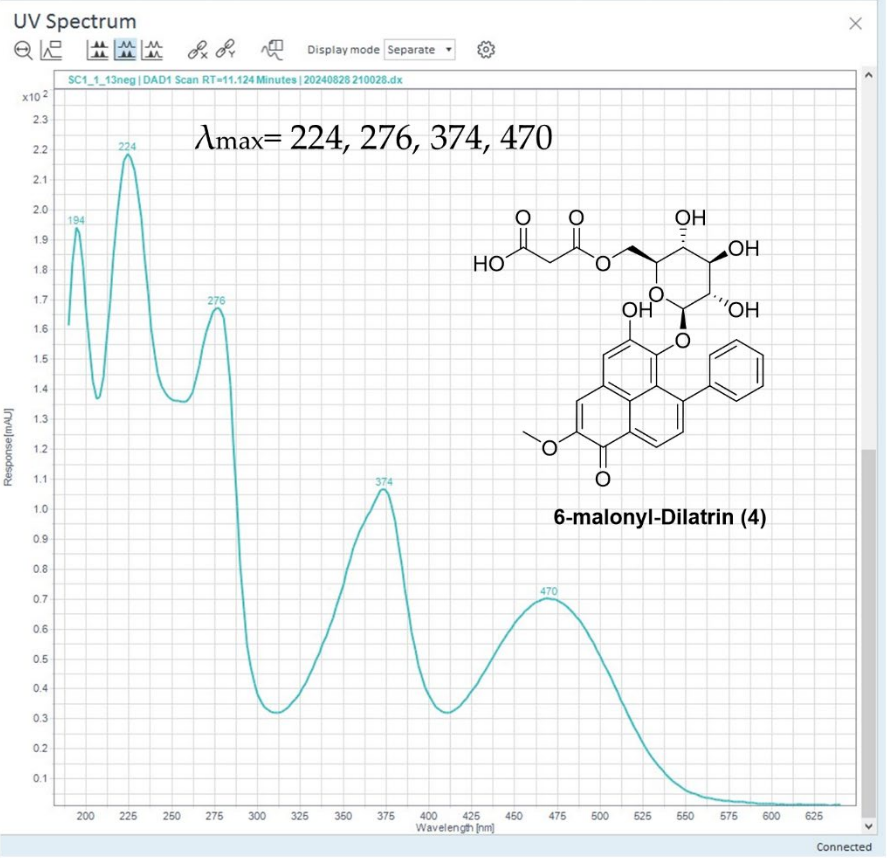

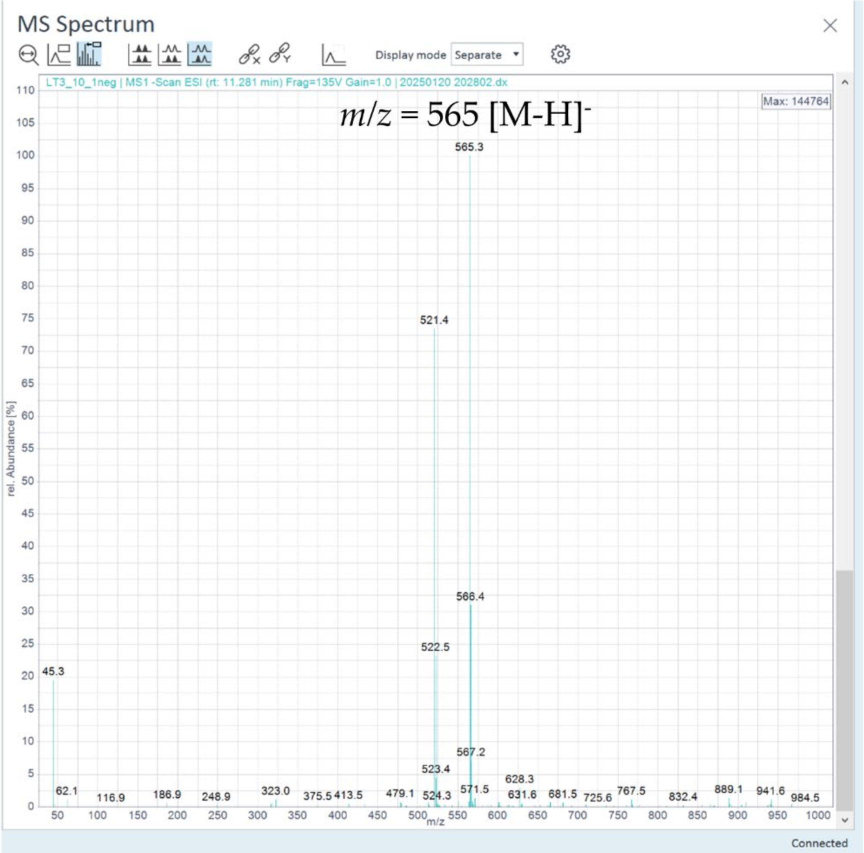

S32. Profiling data (UV chromatogram and ESI-MS) of **(5)** 6-(β-D-glucopyranosyloxy)-5-hydroxy-2-methoxy-7-phenyl-1H-phenalen-1-one

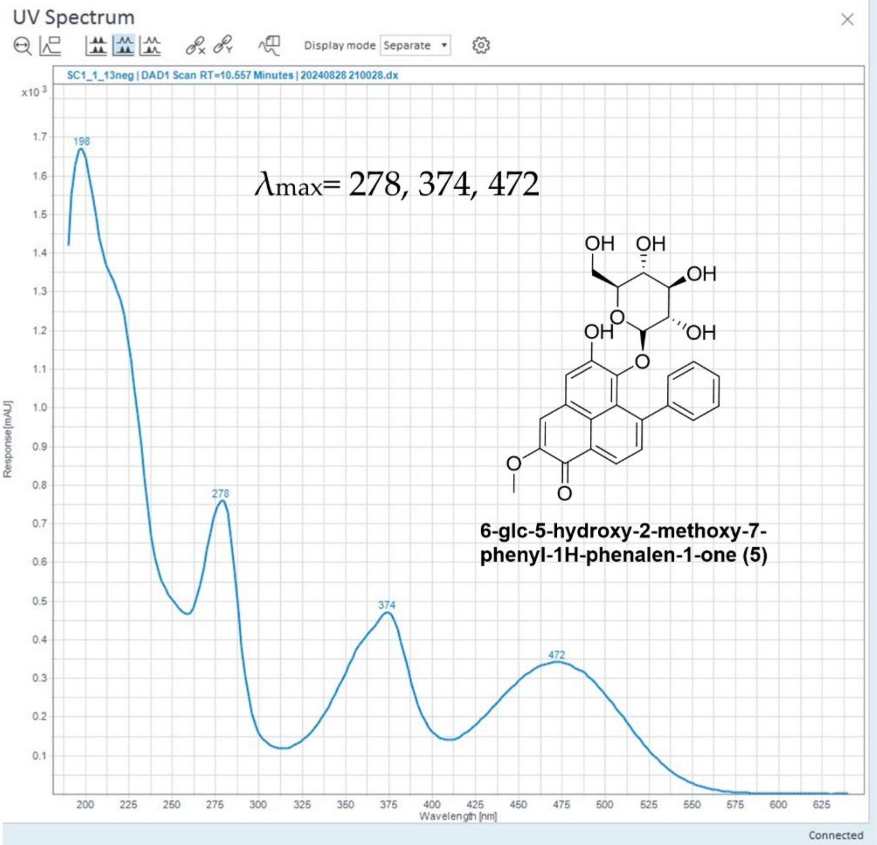

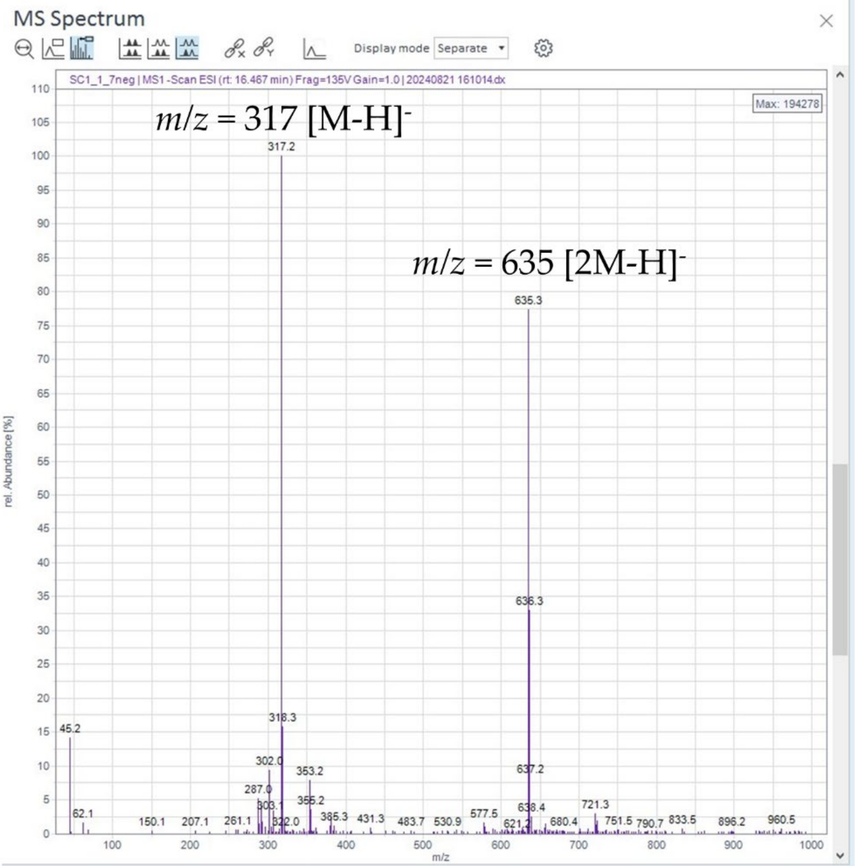

S33. Profiling data (UV chromatogram and ESI-MS) of **(6)** Fuliginol

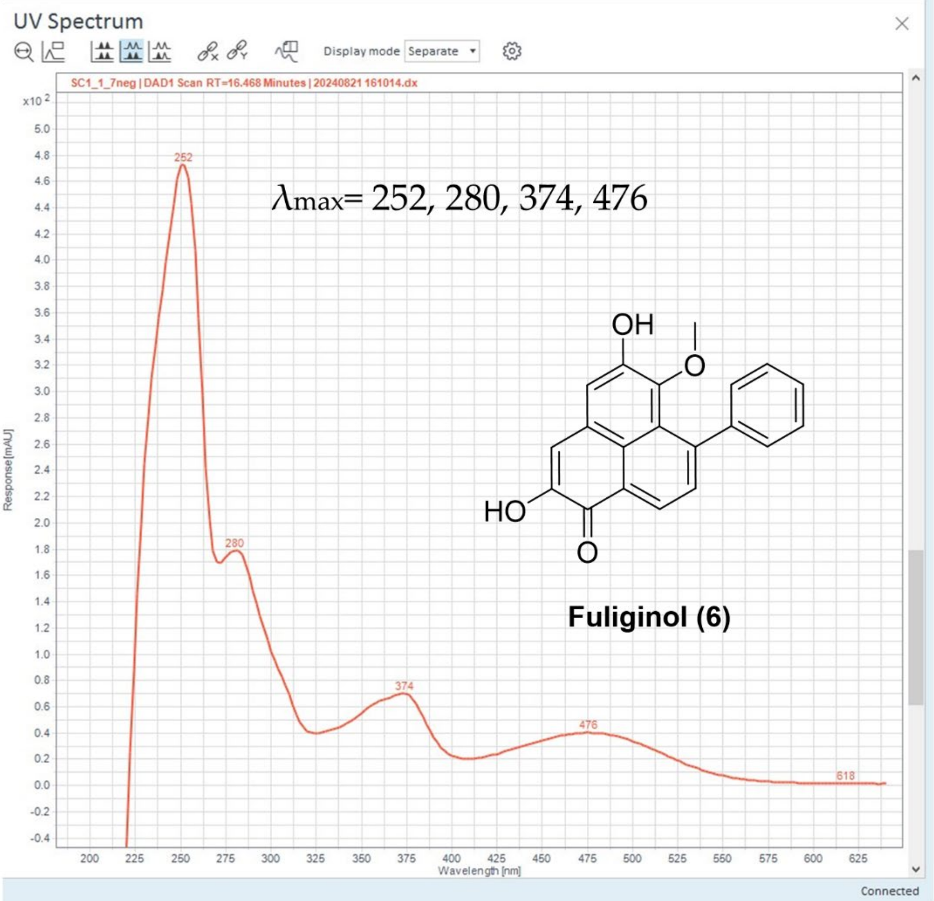

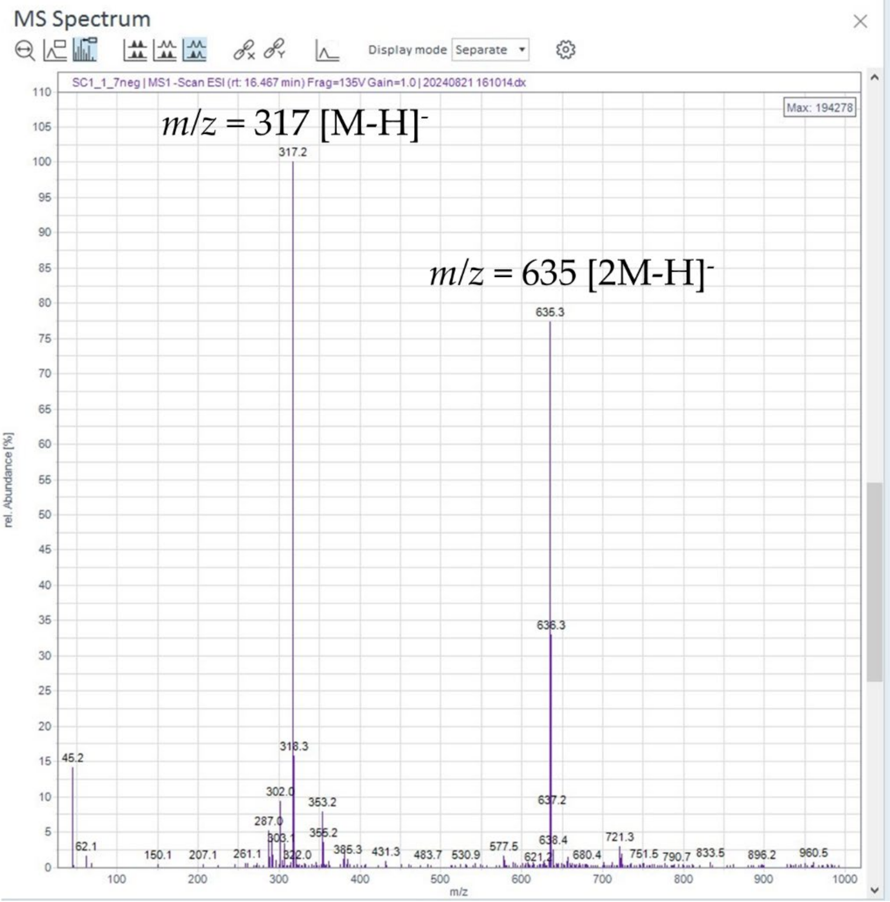

S34. Profiling data (UV chromatogram and ESI-MS) of **(7)** Haemoxiphidone

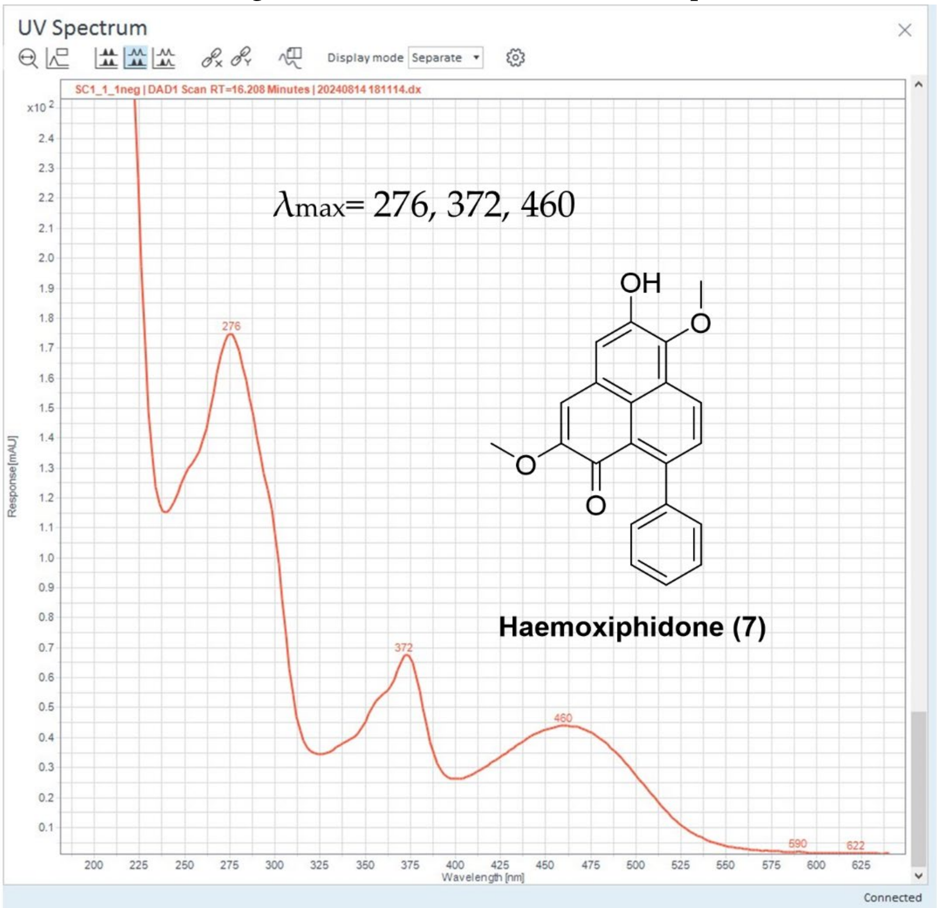

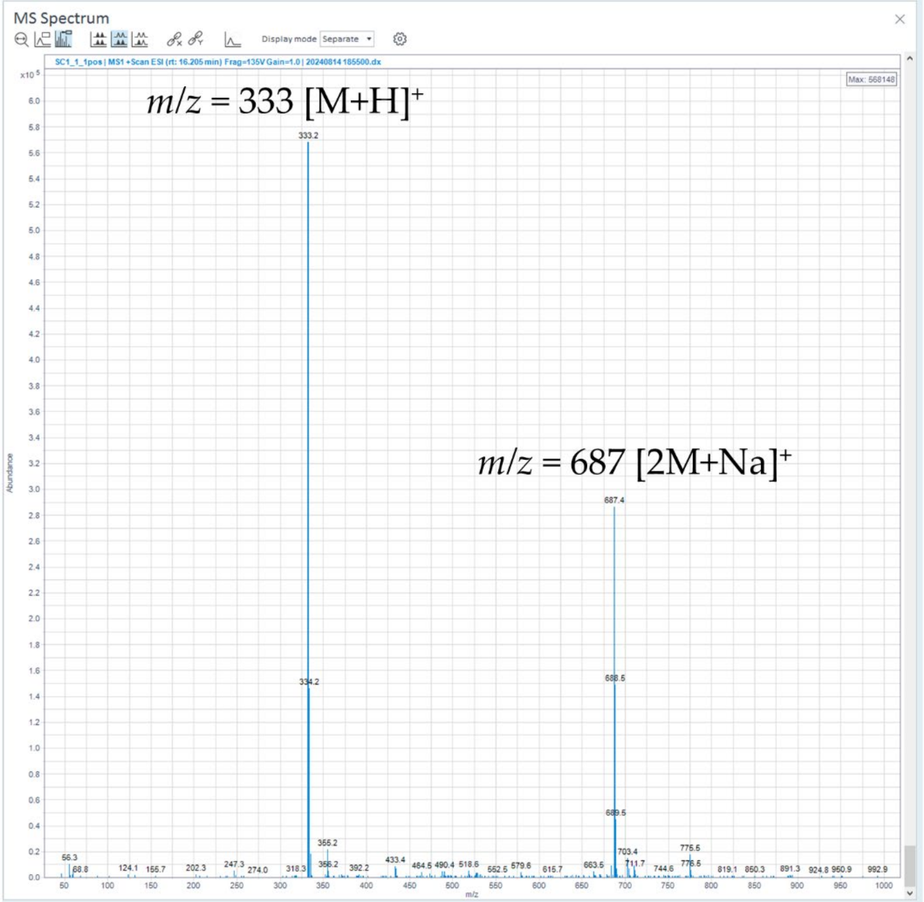

S35. Profiling data (UV chromatogram and ESI-MS) of **(8)** 2,5,6-trimethoxy-9-phenyl-1H-phenalen-1-one

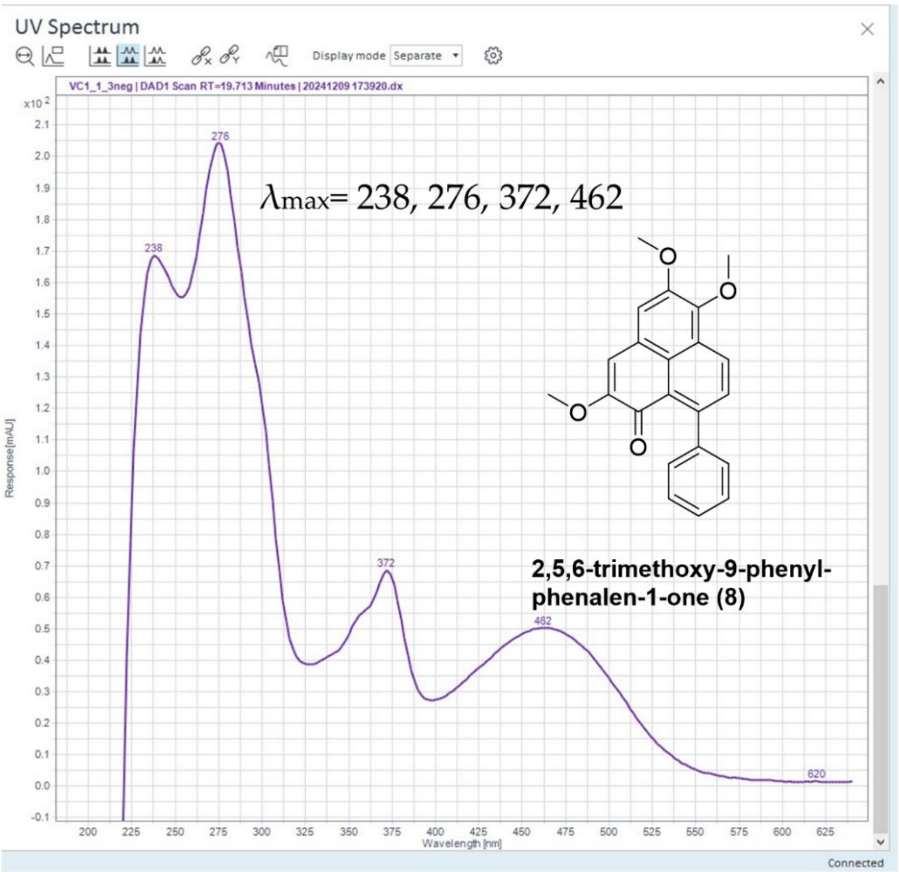

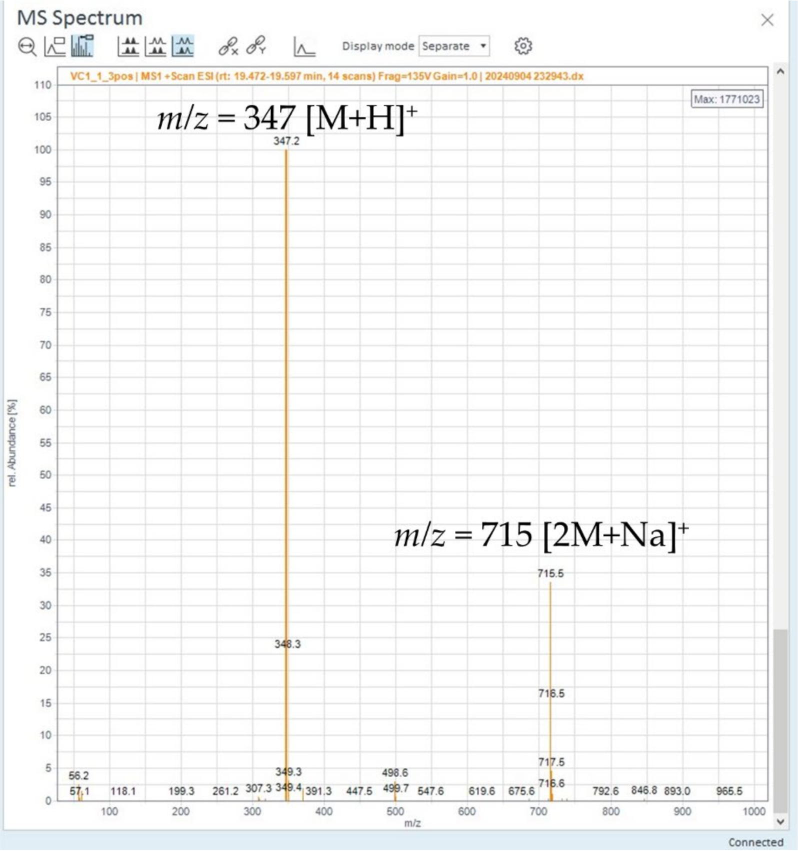

S36. Profiling data (UV chromatogram and ESI-MS) of **(9)** Anigorufone

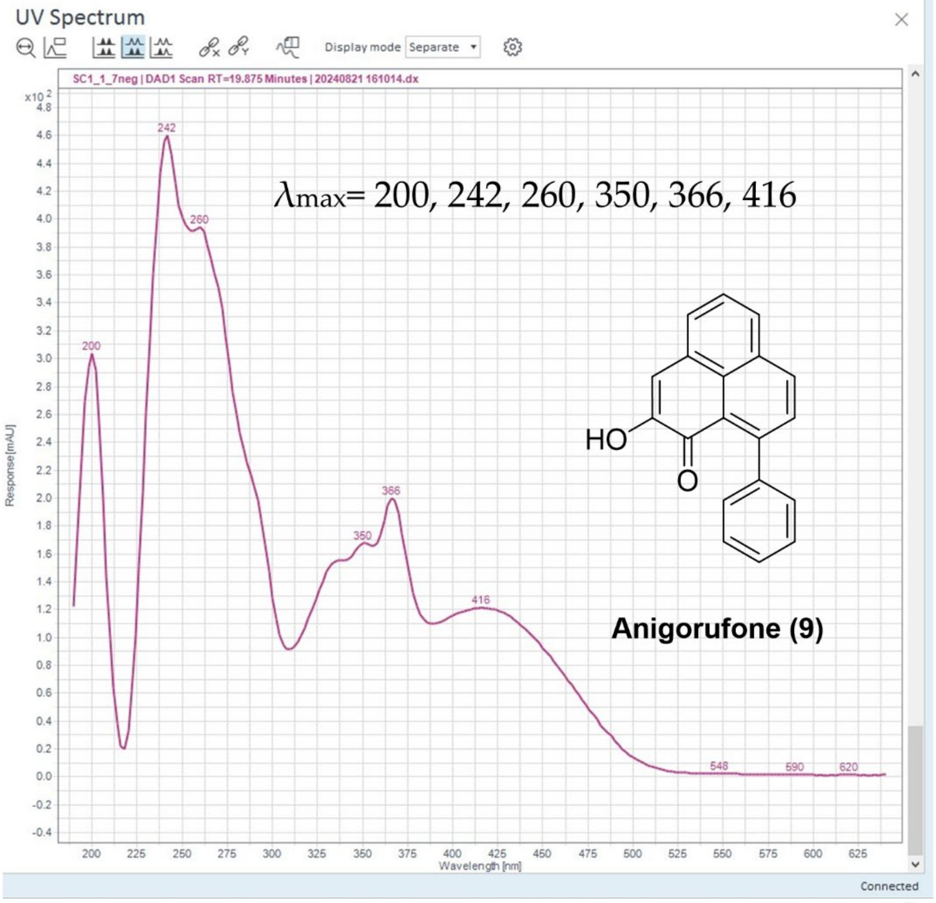

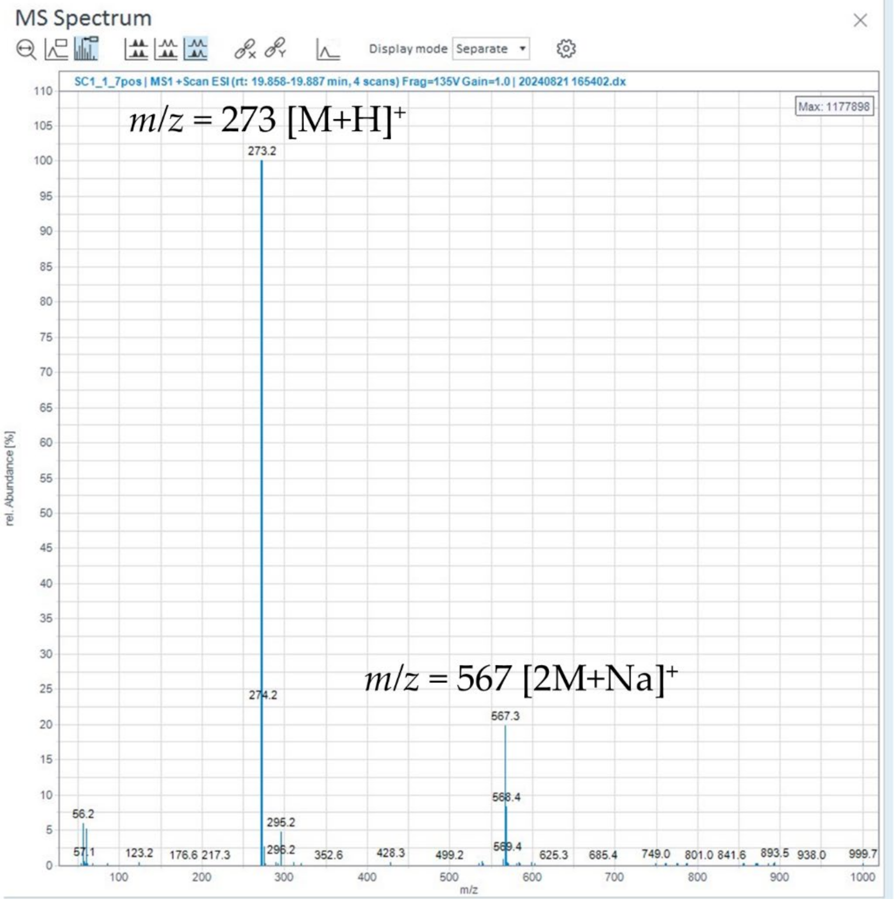

S37. Profiling data (UV chromatogram and ESI-MS) of **(10)** 2,6-dimethoxy-9-phenyl-1H-phenalen-1-one

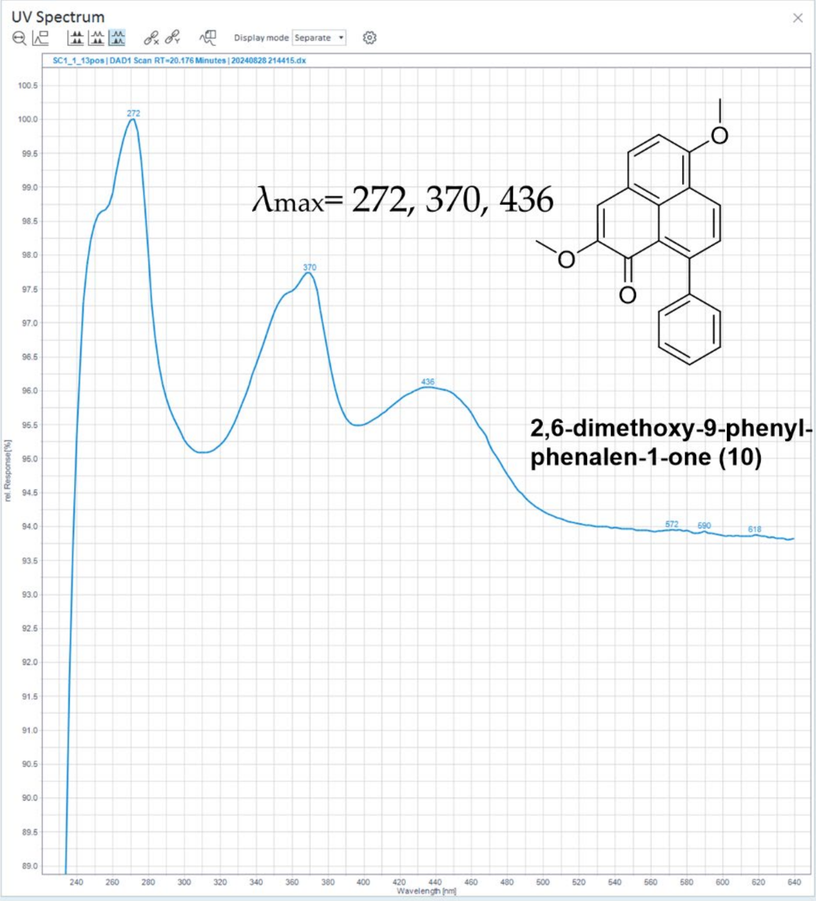

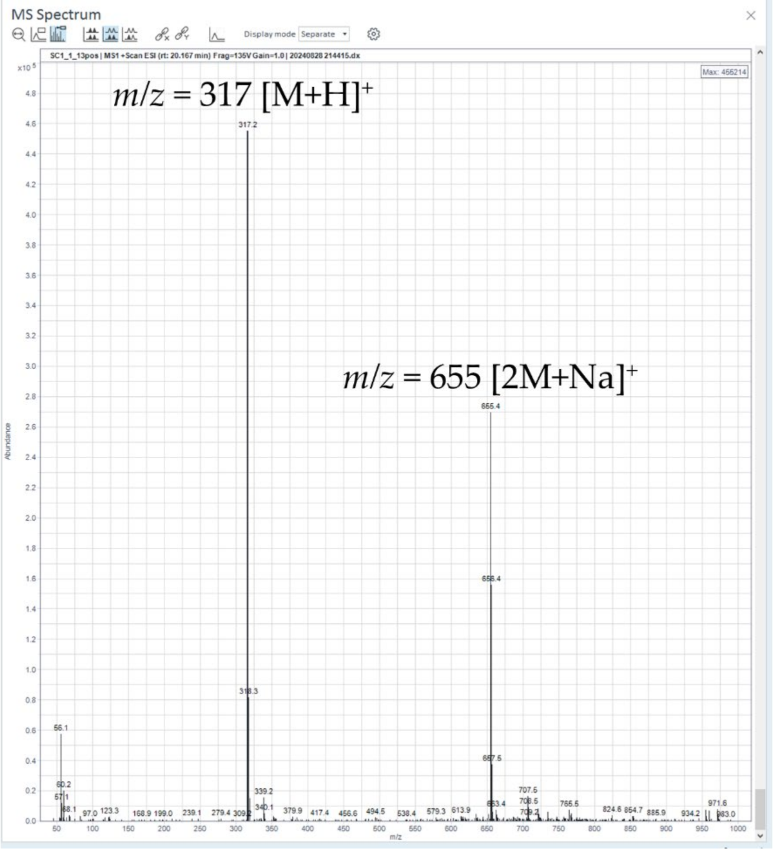

S38. Profiling data (UV chromatogram and ESI-MS) of **(11)** 2-hydroxy-8-(2-hydroxyphenyl)-6-((3,4,5-trihydroxy-6-(hydroxymethyl)tetrahydro-pyran-2-yl)oxy)-phenalenone

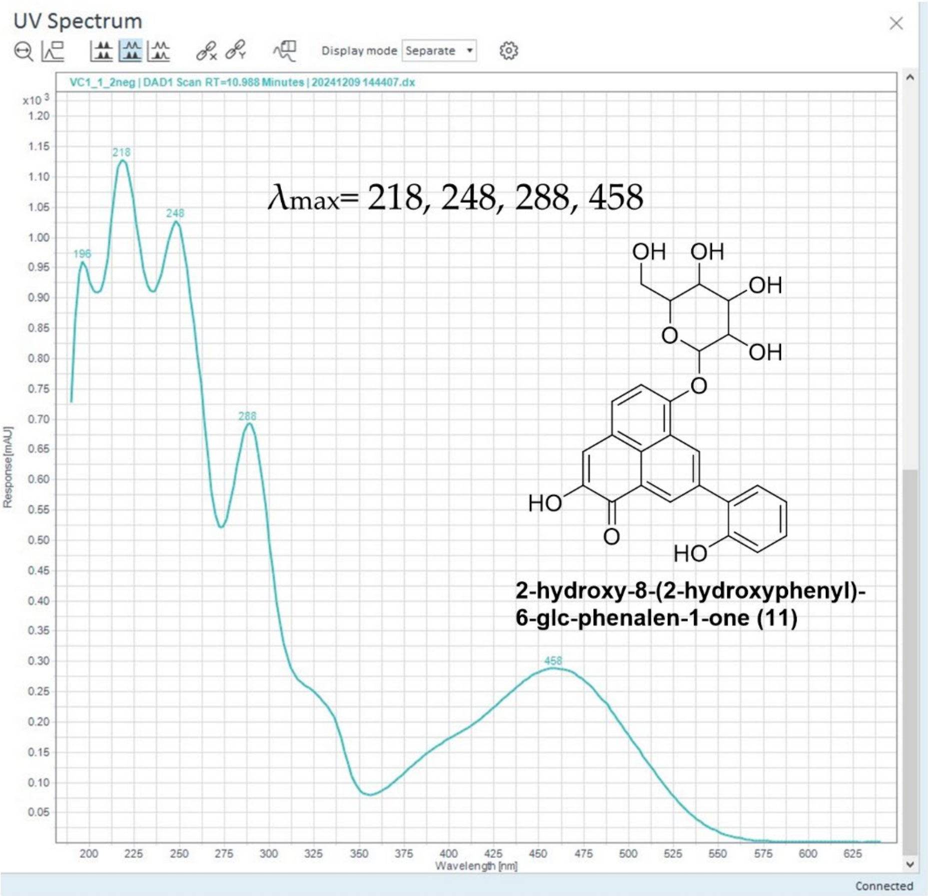

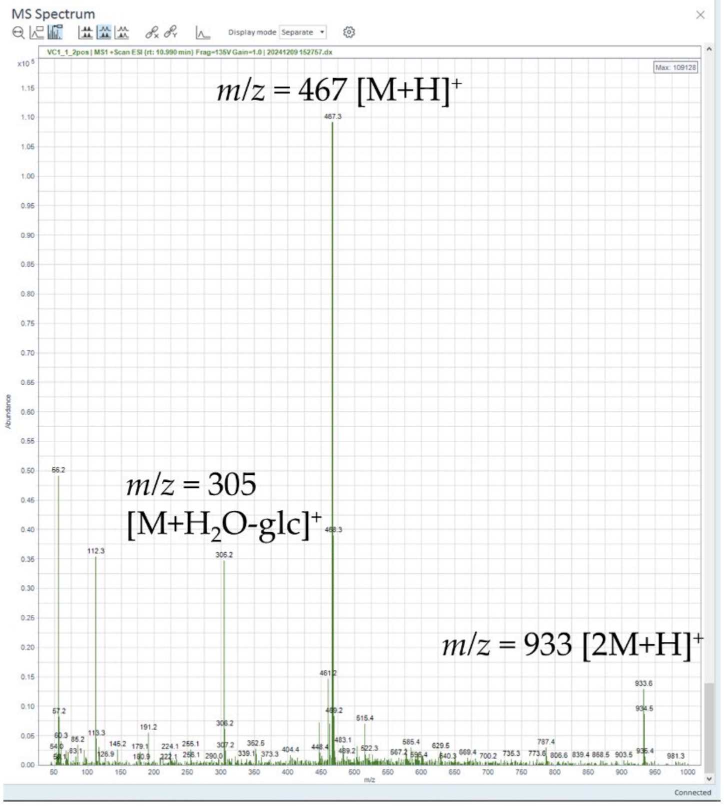

S39. Profiling data (UV chromatogram and ESI-MS) of **(12)** Hemoflurone A

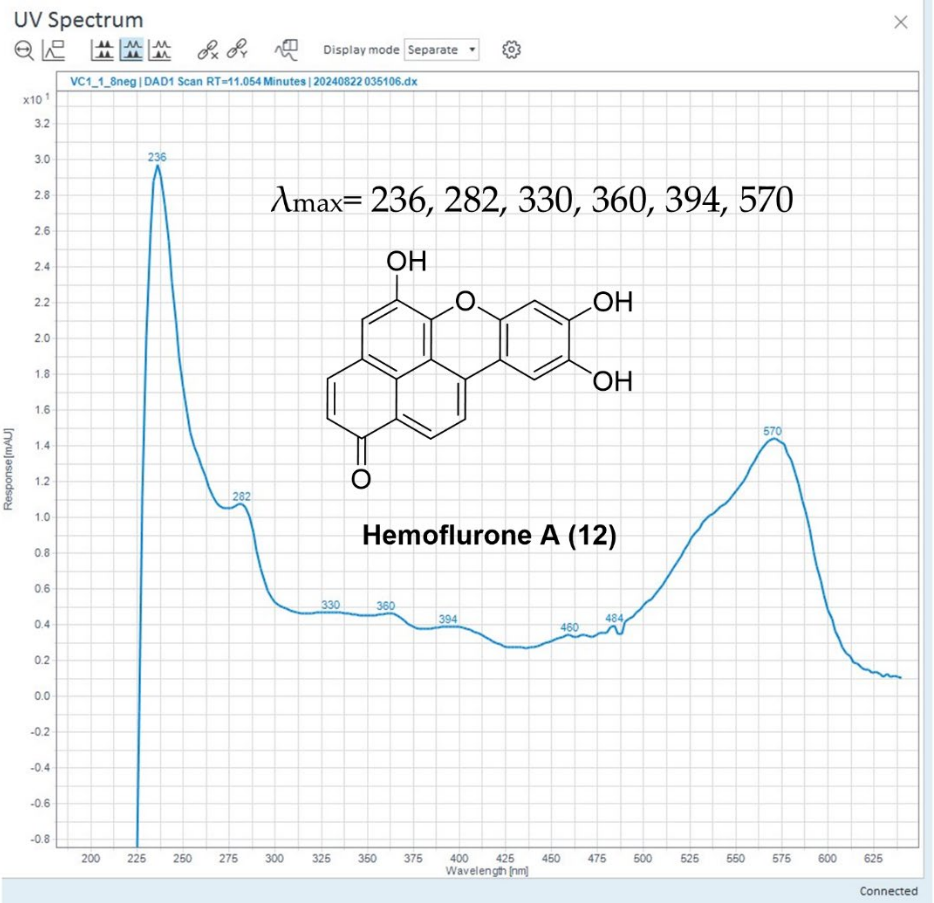

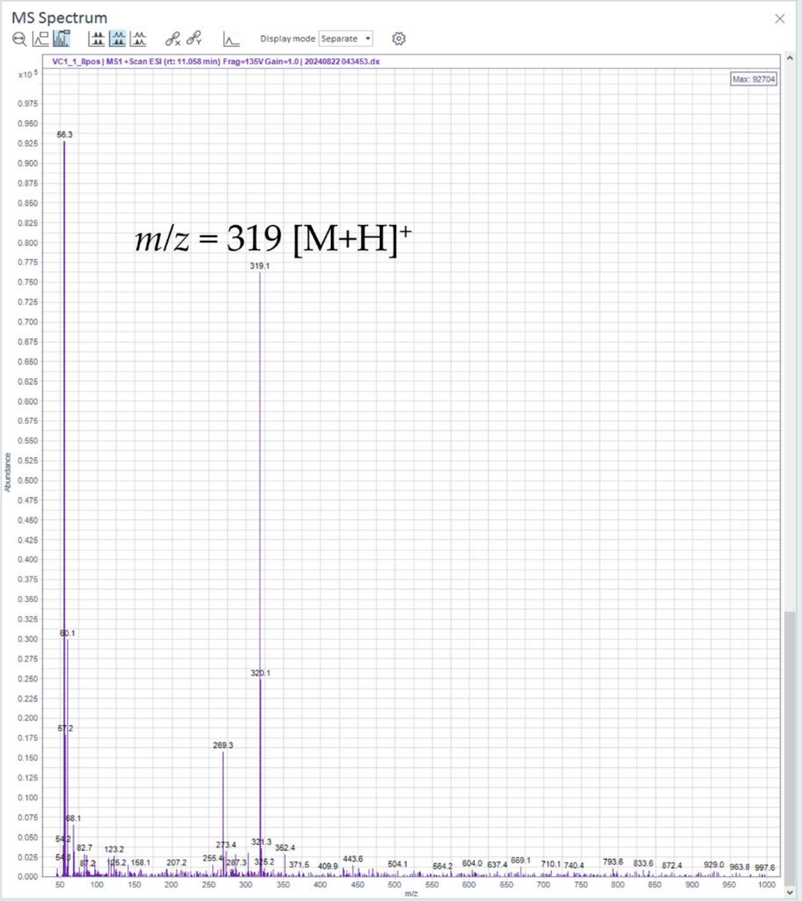

S40. Profiling data (UV chromatogram and ESI-MS) of **(13)** Haemodoroxychrysenose

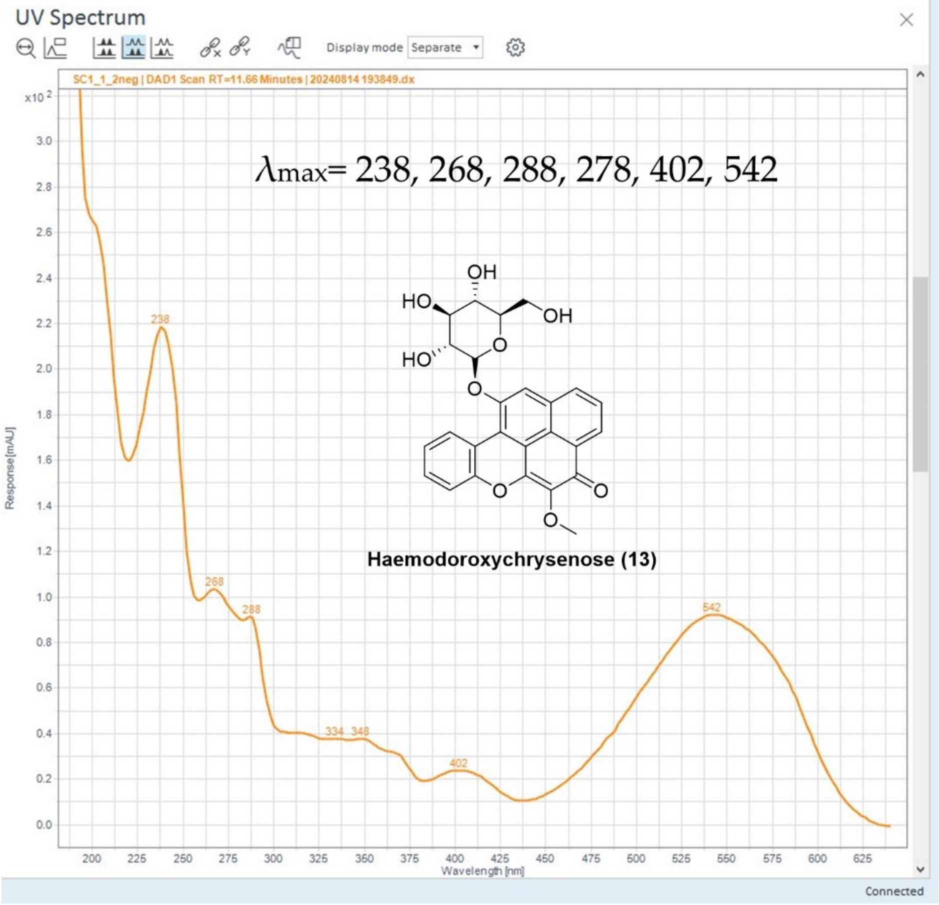

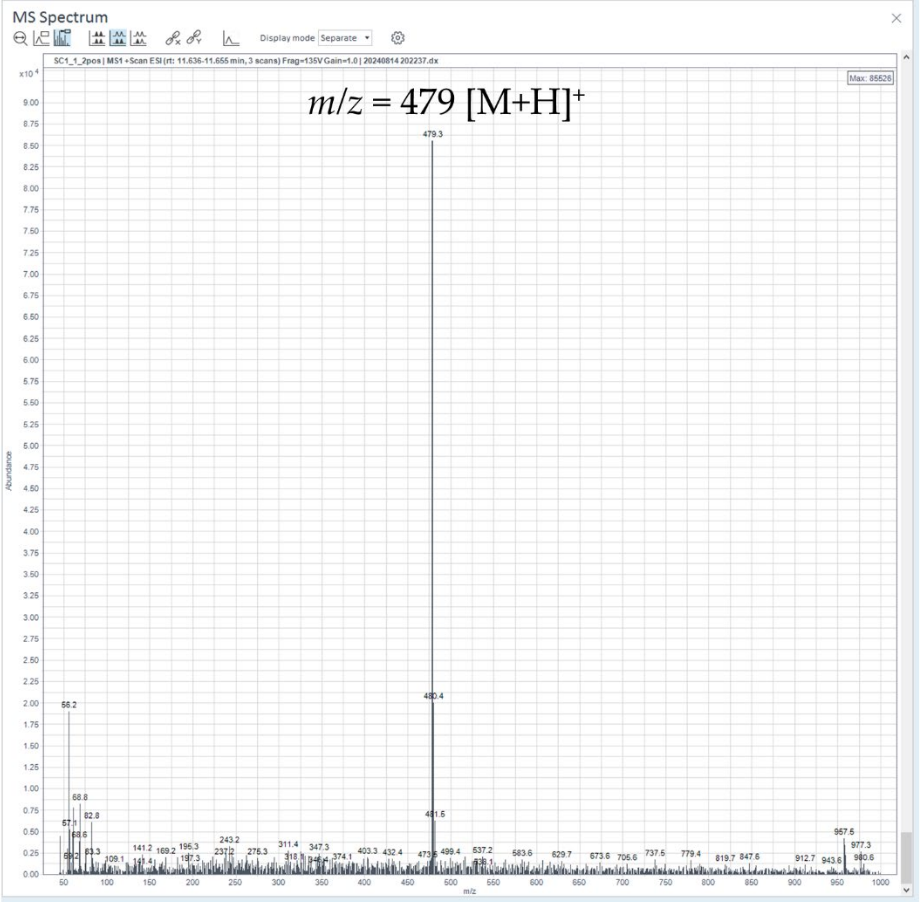

S41. Profiling data (UV chromatogram and ESI-MS) of **(14)** 5-hydroxy-1H-naphtho[2,1,8-mna]xanthen-1-one

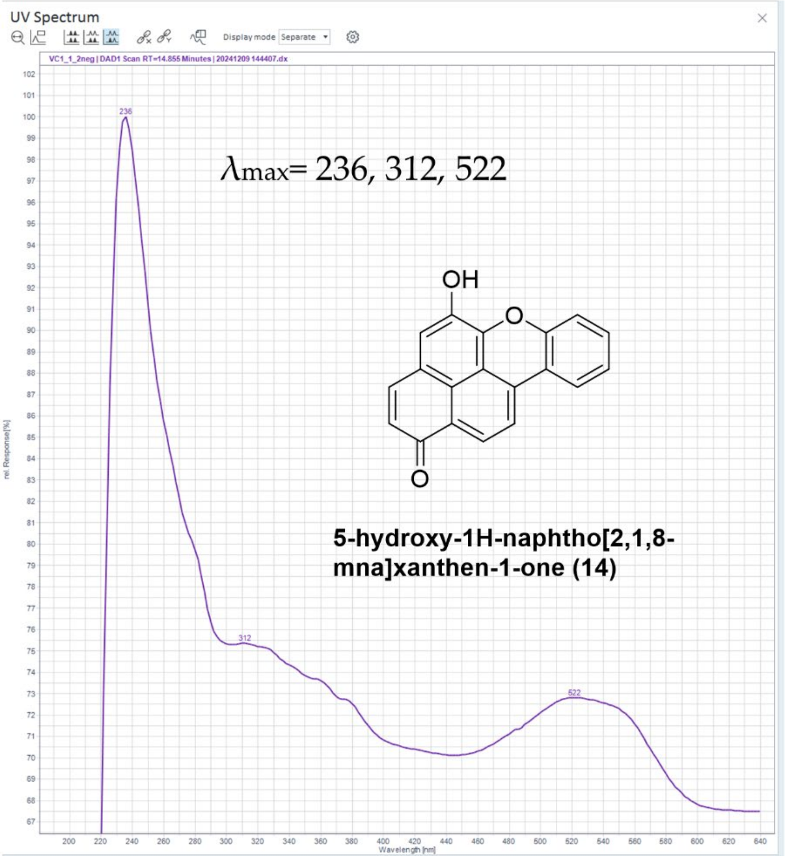

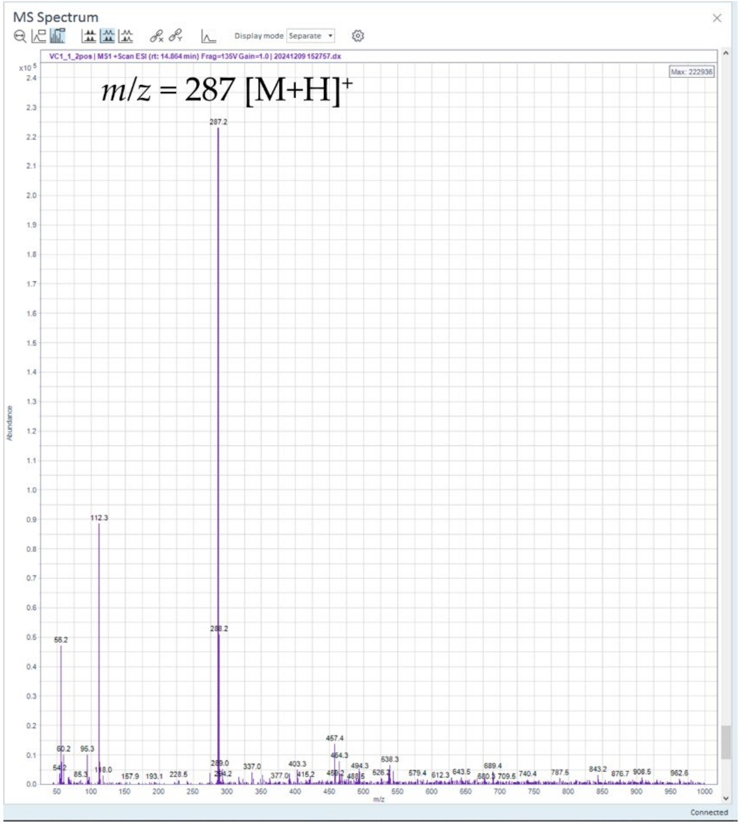

S42. Profiling data (UV chromatogram and ESI-MS) of **(15)** 5-hydroxy-2-methoxy-1H-naphtho[2,1,8-mna]xanthen-1-one

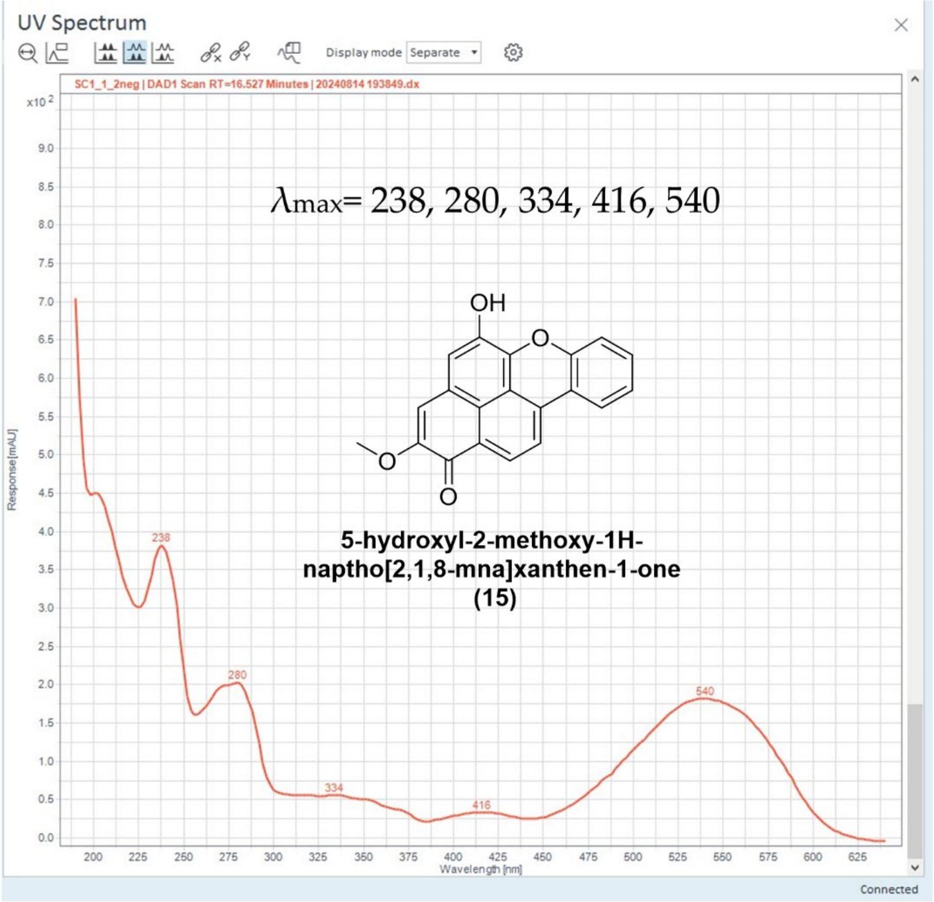

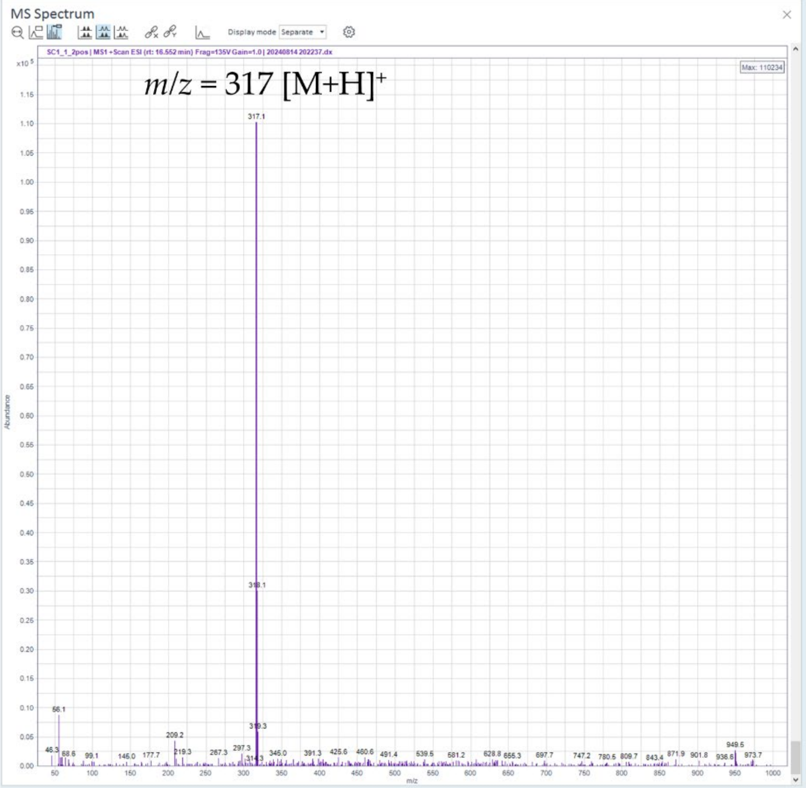

S43. Profiling data (UV chromatogram and ESI-MS) of **(16)** 5-methoxy-1H-naphtho[2,1,8-mna]xanthen-1-one

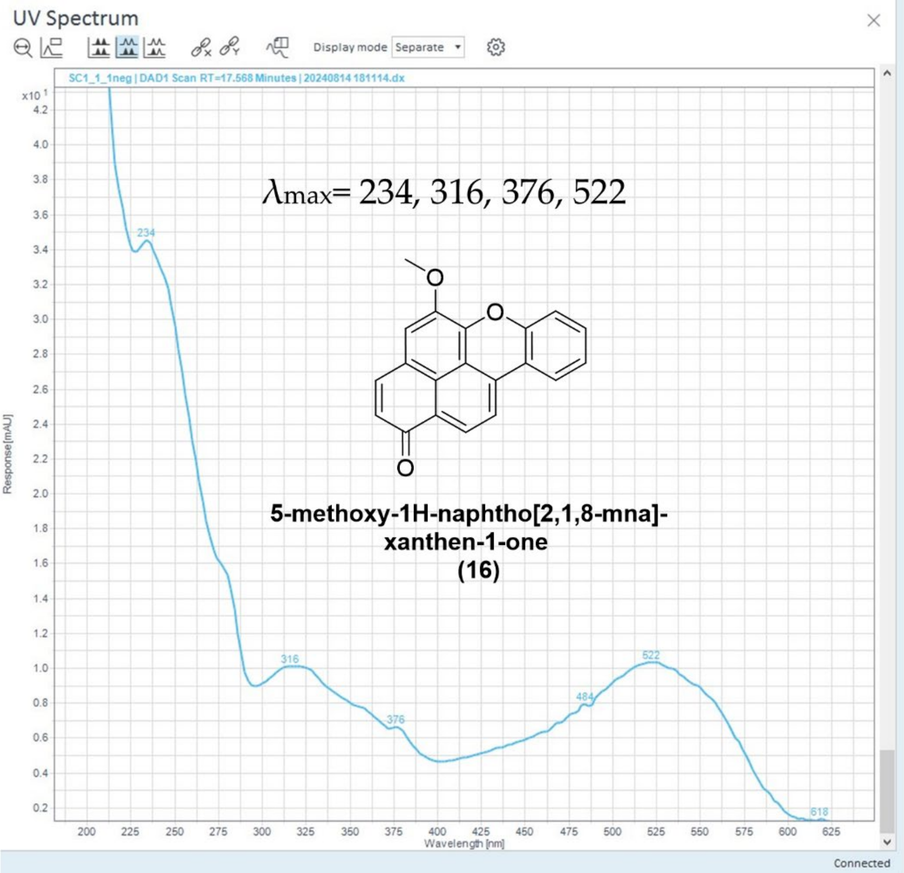

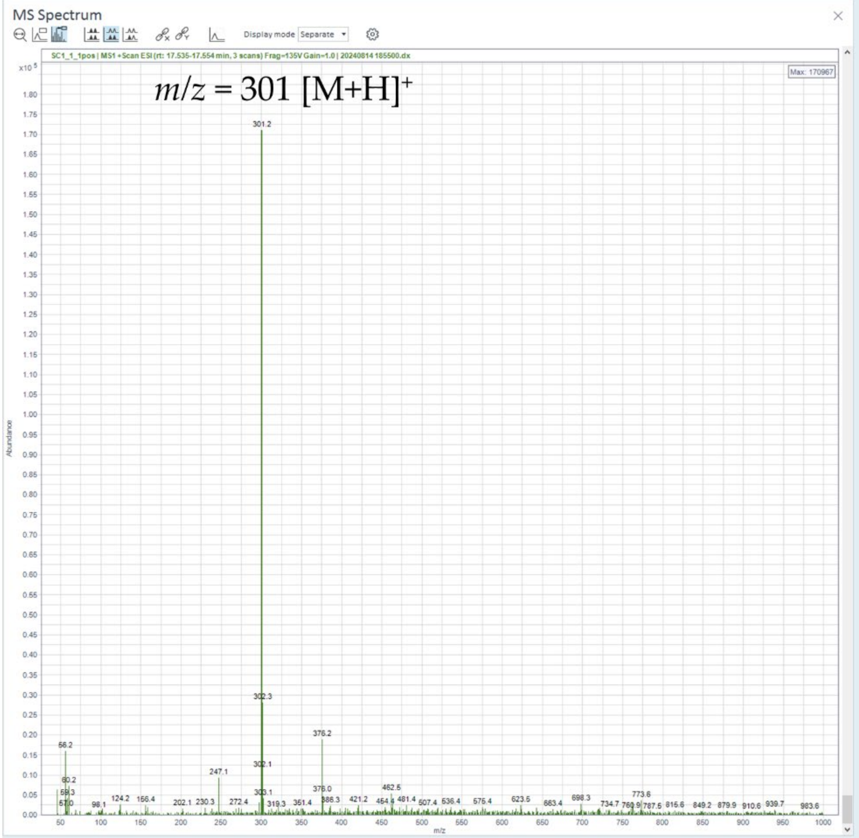

S44. Profiling data (UV chromatogram and ESI-MS) of **(17)** 2,5-dimethoxy-1H-naphtho[2,1,8-mna]xanthen-1-one

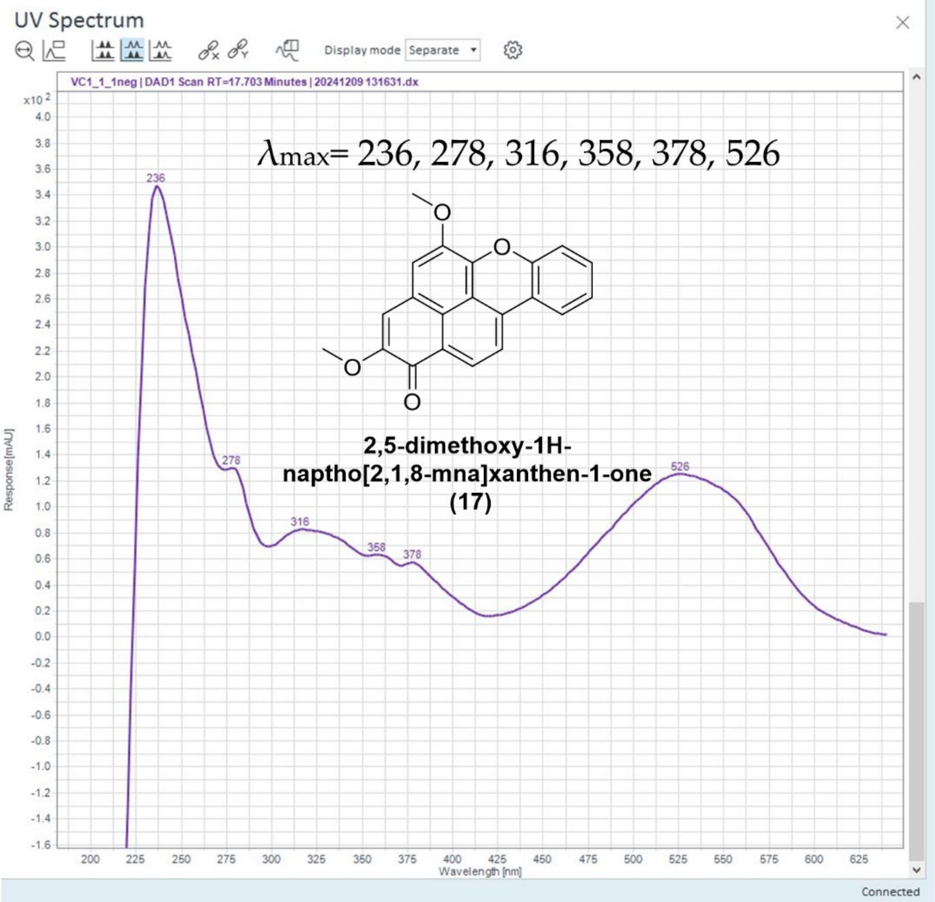

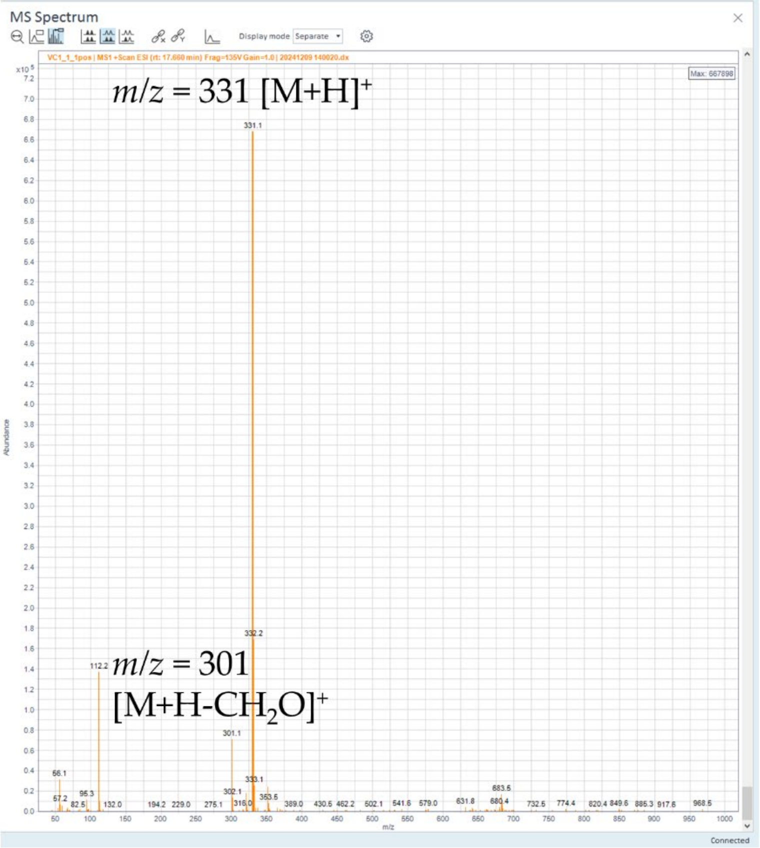

S45. Profiling data (UV chromatogram and ESI-MS) of **(18)** Hemoflurone B

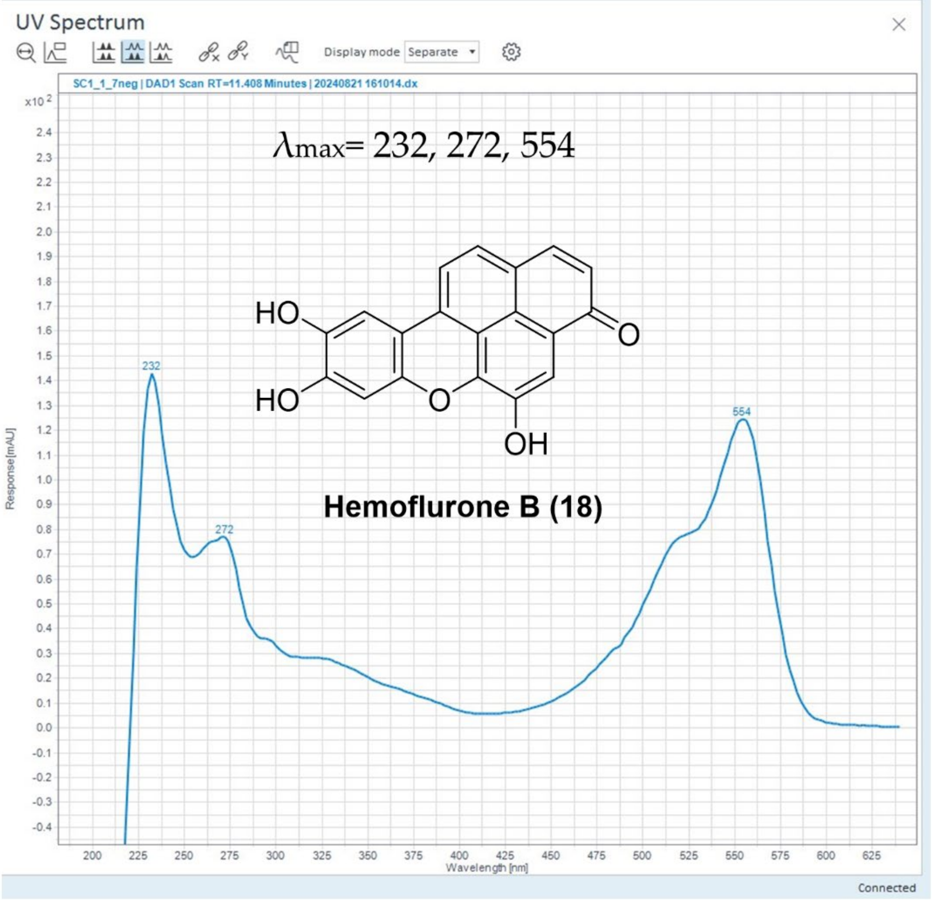

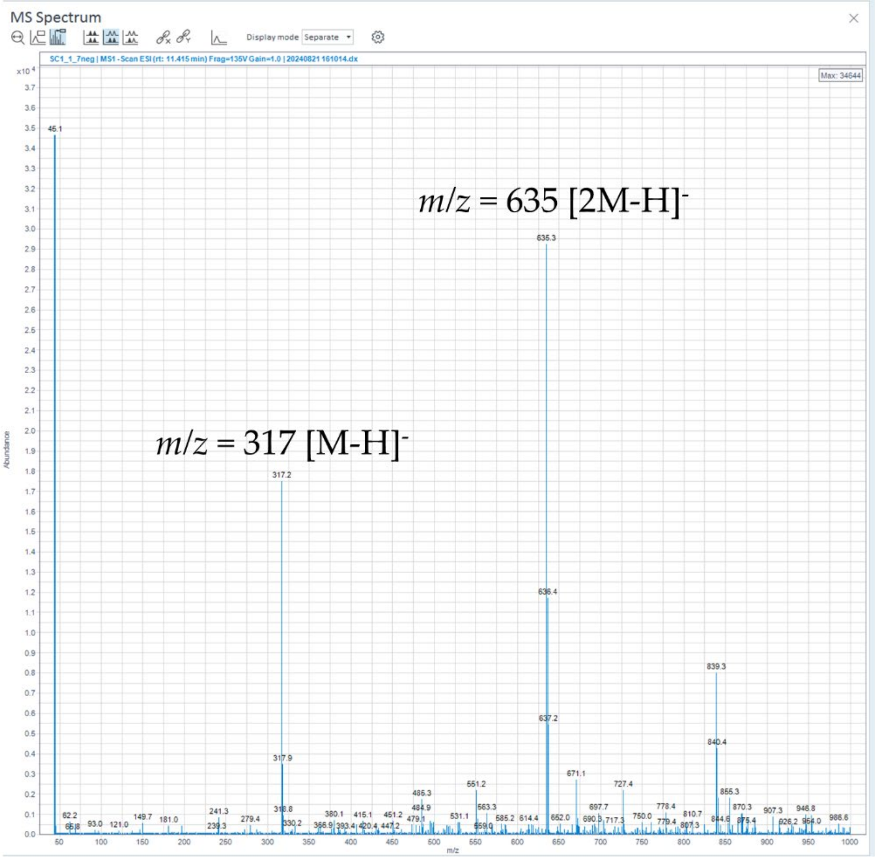

S46. Profiling data (UV chromatogram and ESI-MS) of **(19)** 6-(β-D-glycopyranosyl)-5-hydroxy-7-phenyl-1H-benzo[de]isochromen-1-one

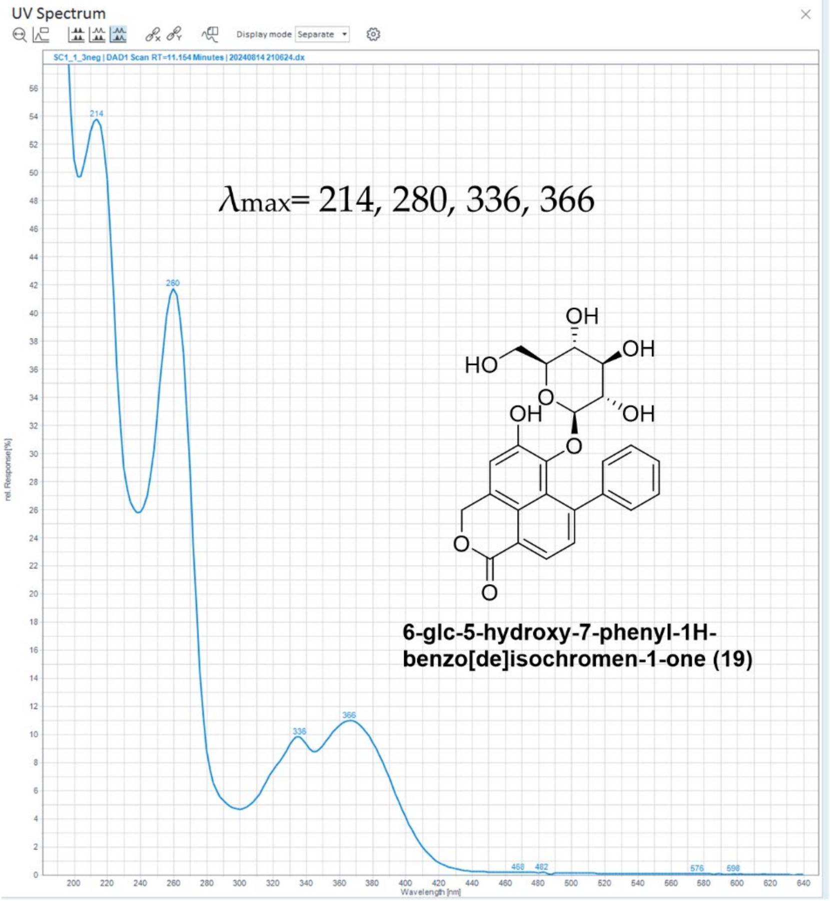

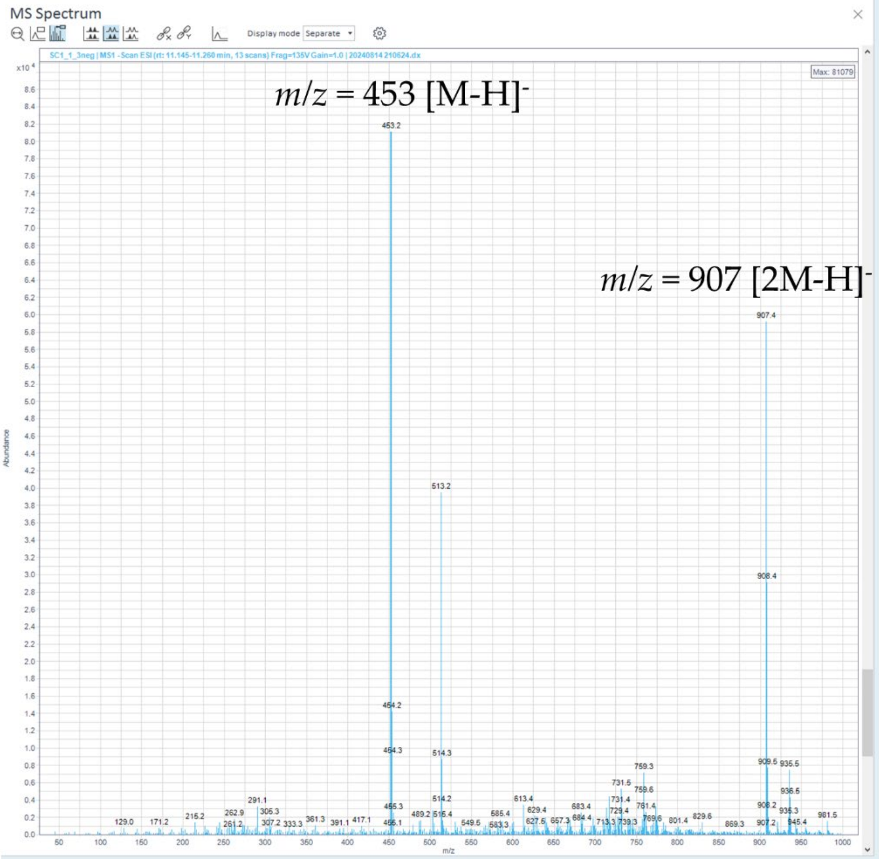

S47. Profiling data (UV chromatogram and ESI-MS) of **(20)** 6-O-[(6″-O-malonyl)-β-D-glucopyranosyl]-5-hydroxy-7-phenyl-3H-benzo[de]isochromen-1-one

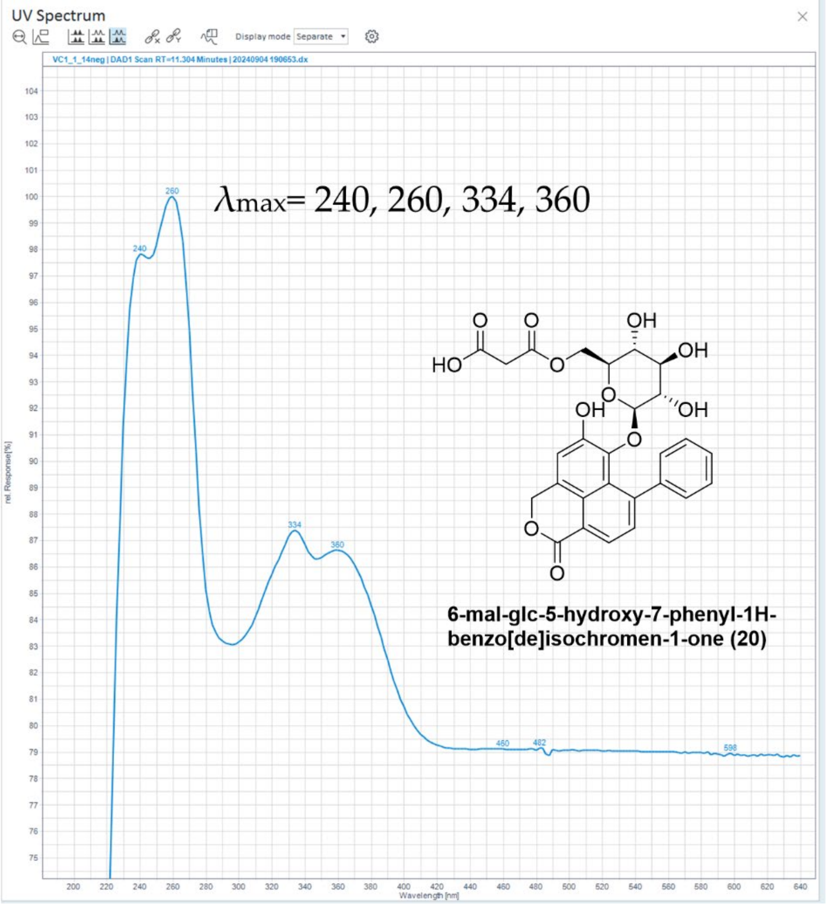

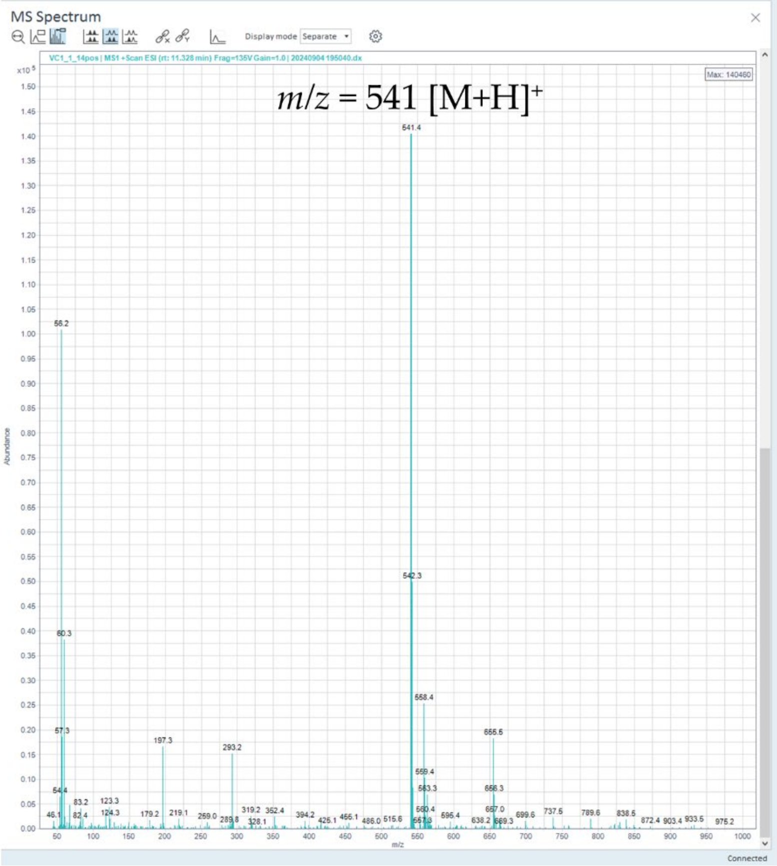

S48. Profiling data (UV chromatogram and ESI-MS) of **(21)** Haemodorose

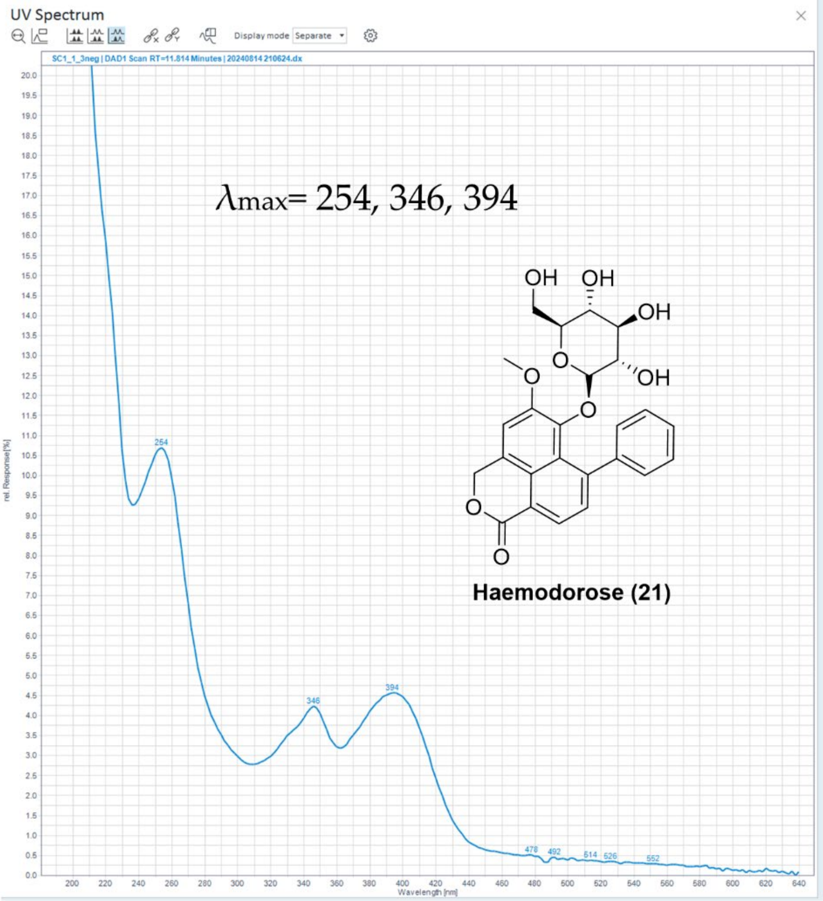

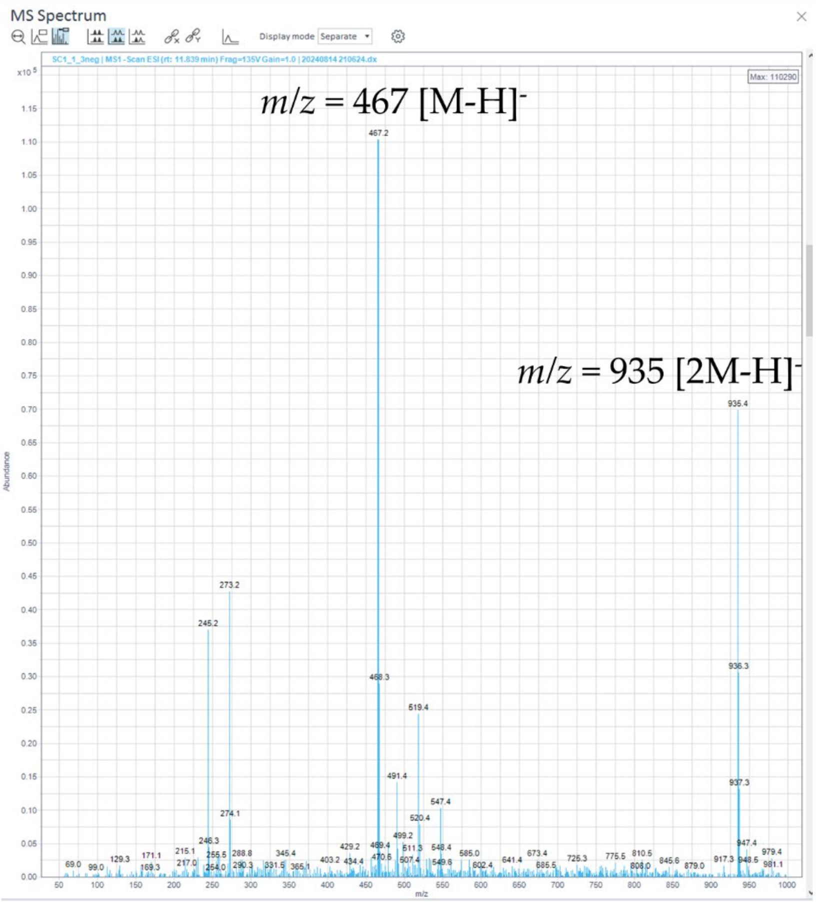

S49. Profiling data (UV chromatogram and ESI-MS) of **(22)** 6-(β-D-glycopyranosyl)-5-hydroxy-7(4′-hydroxyphenyl)-1H,3H-benzo[de]isochromen-1-one

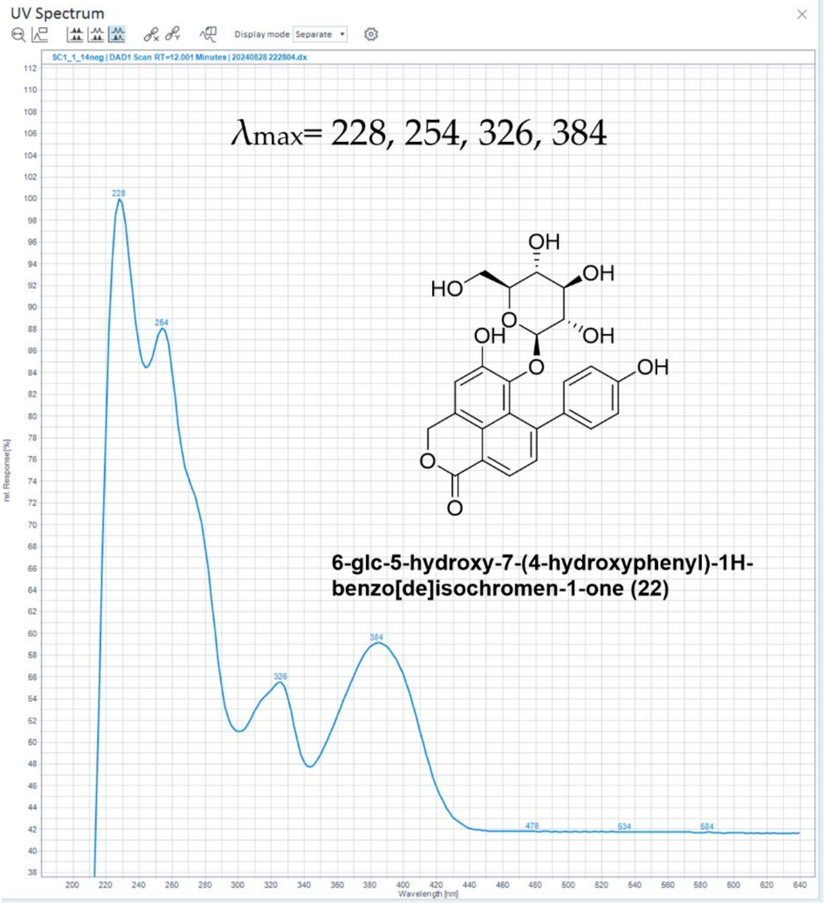

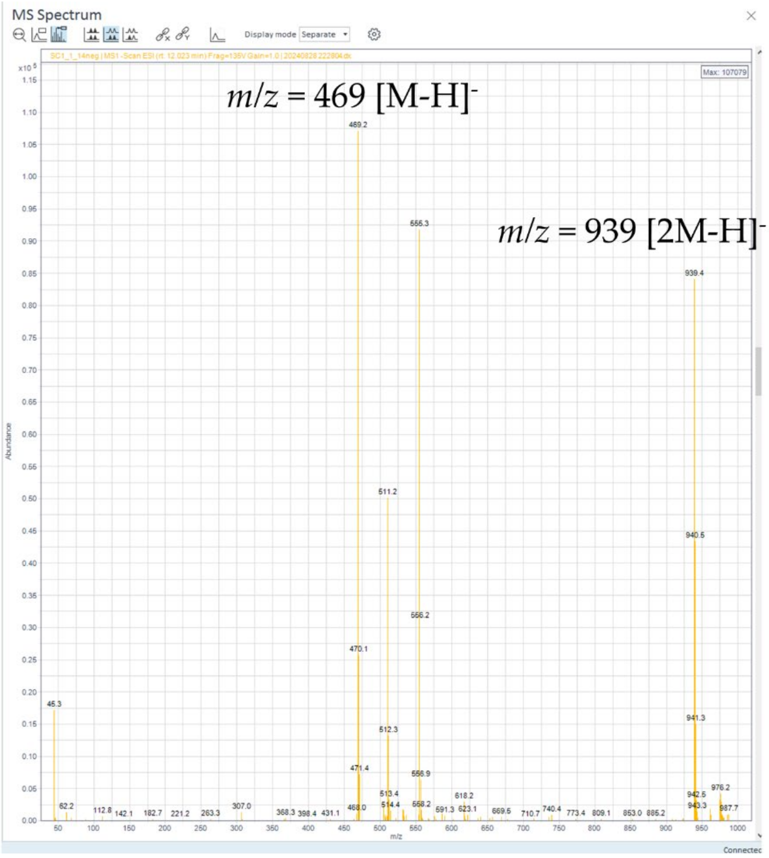

S50. Profiling data (UV chromatogram and ESI-MS) of **(23)** Haemodordioxolane

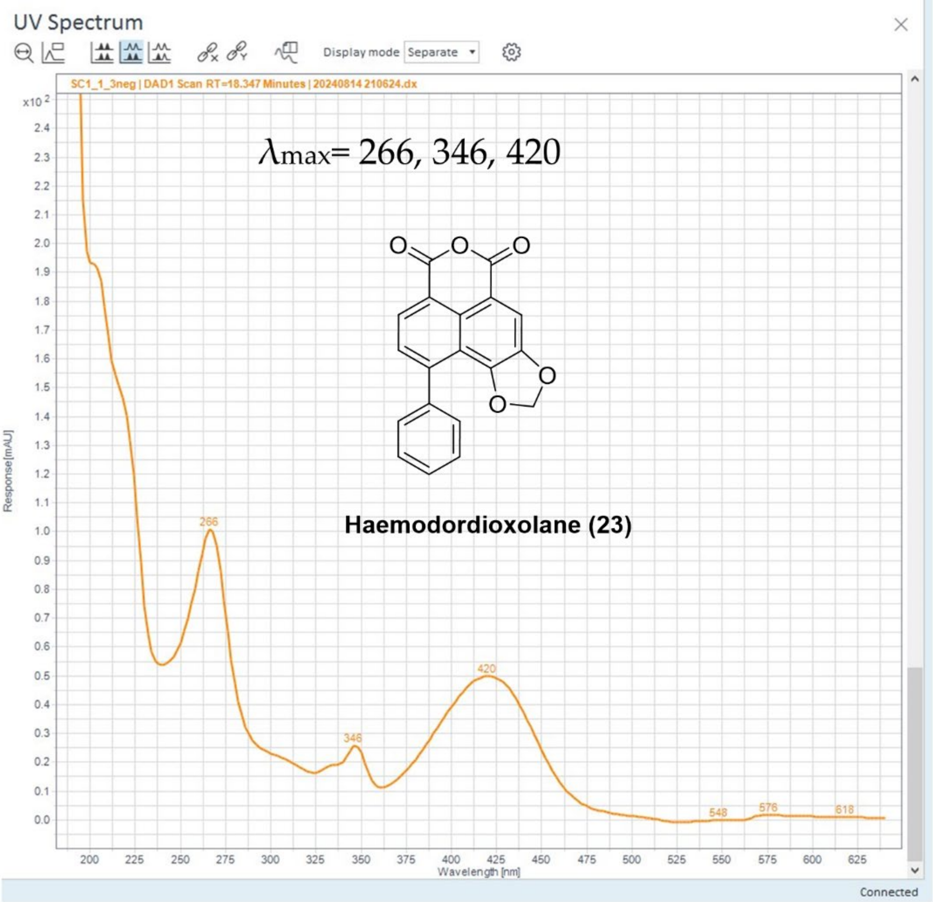

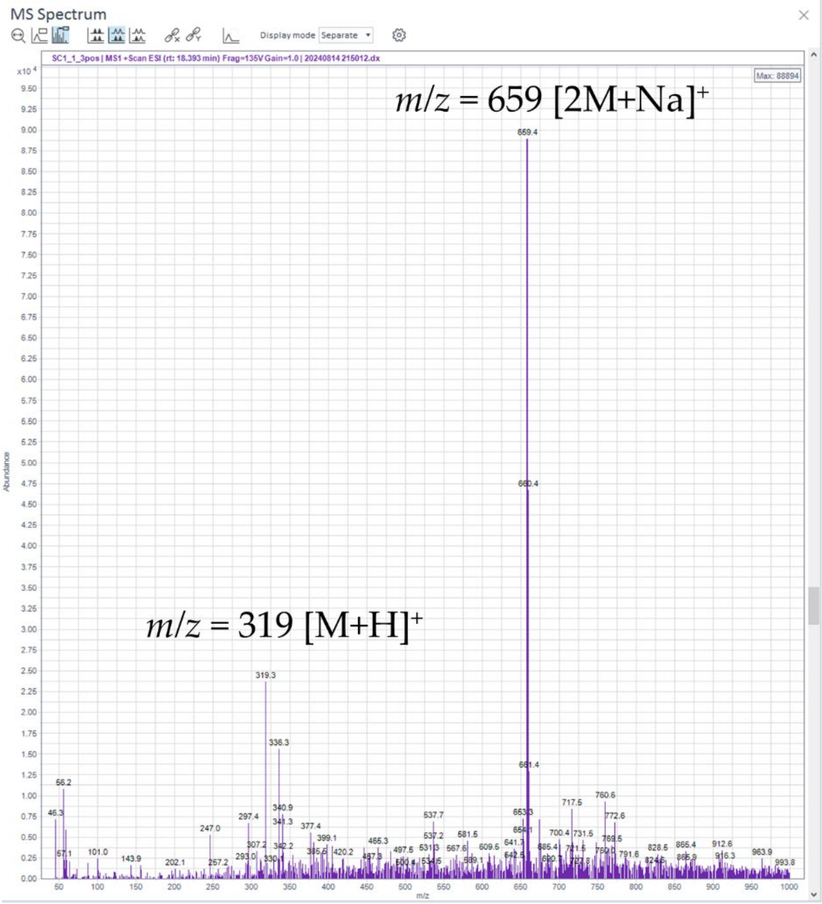

S51. Profiling data (UV chromatogram and ESI-MS) of **(24)** Haemodorol

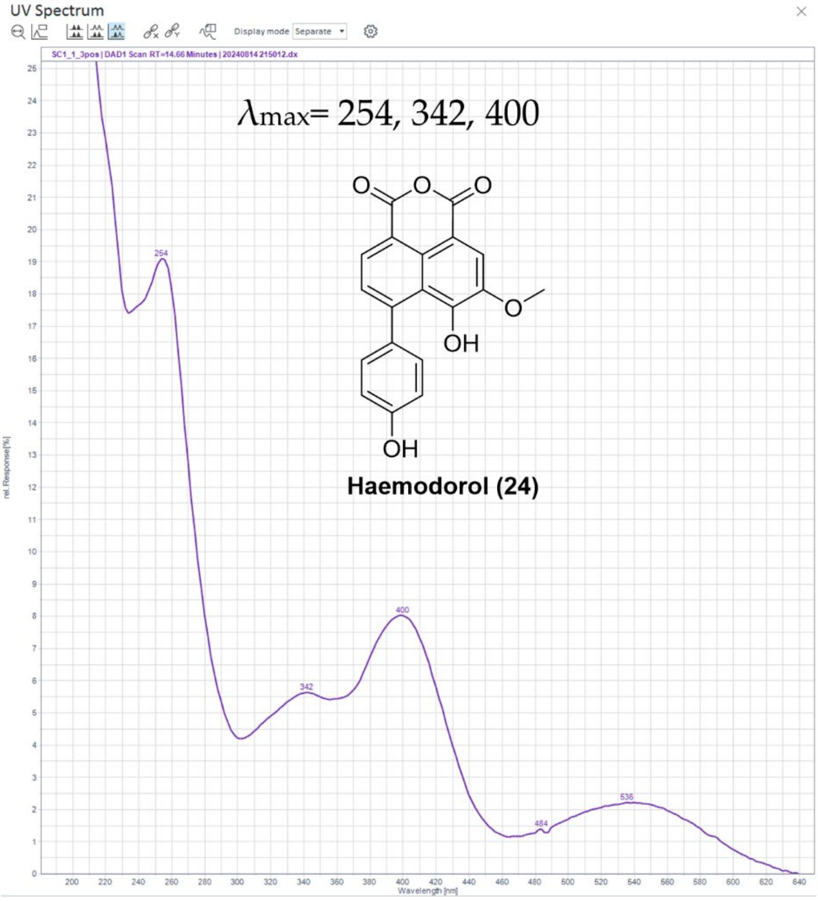

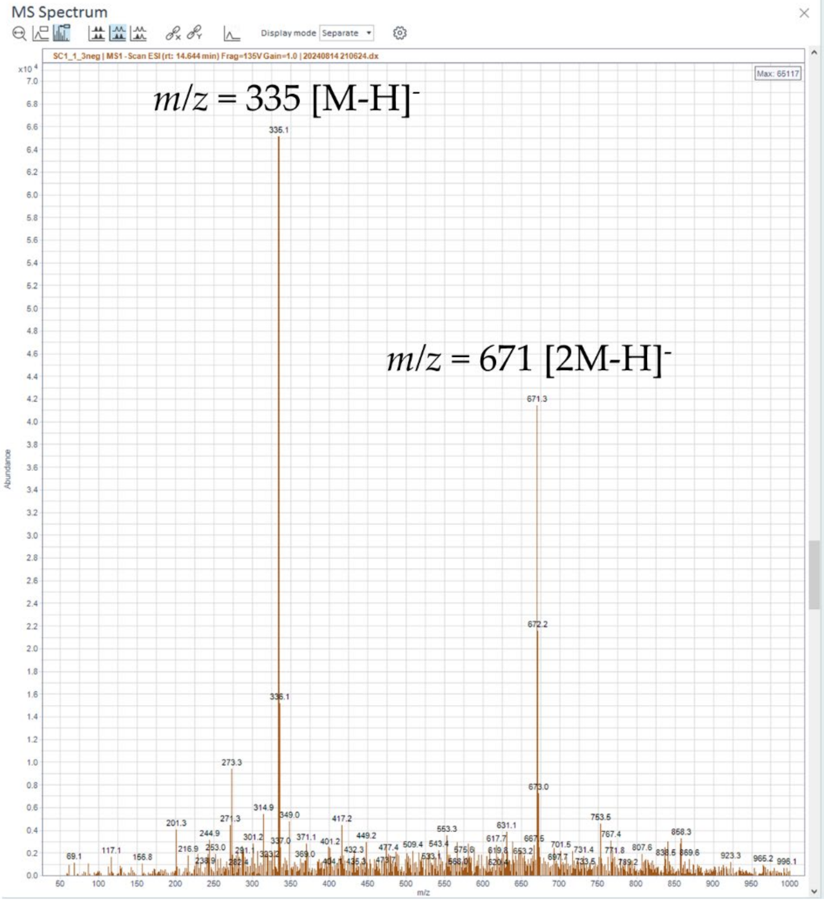

S52. Profiling data (UV chromatogram and ESI-MS) of **(25)** Haemodordione

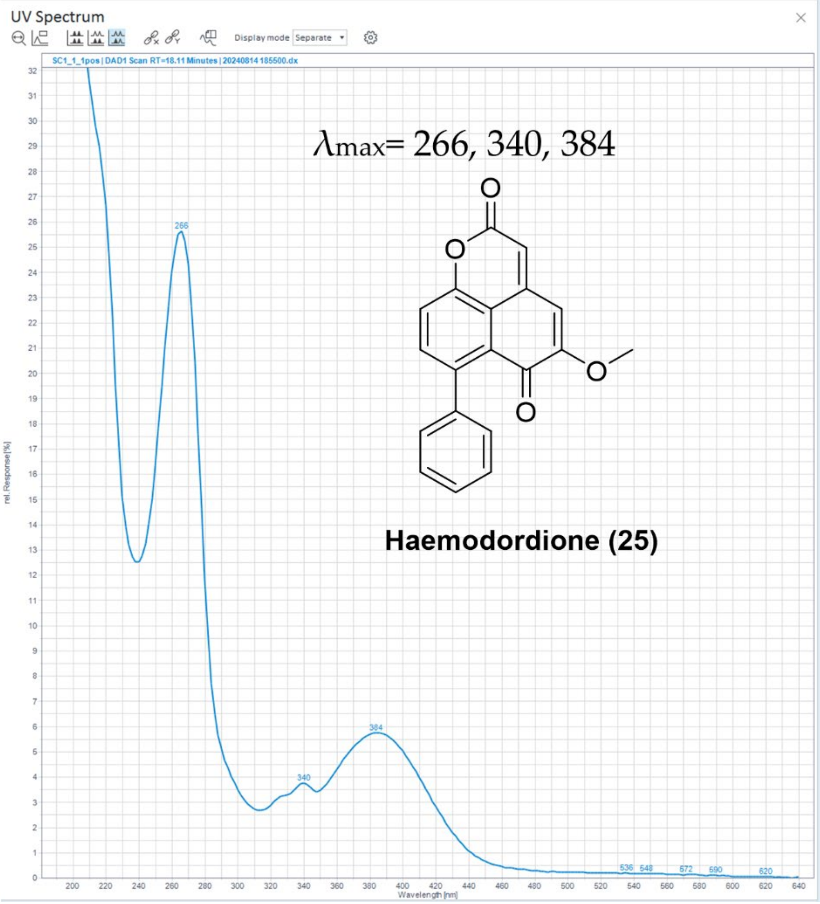

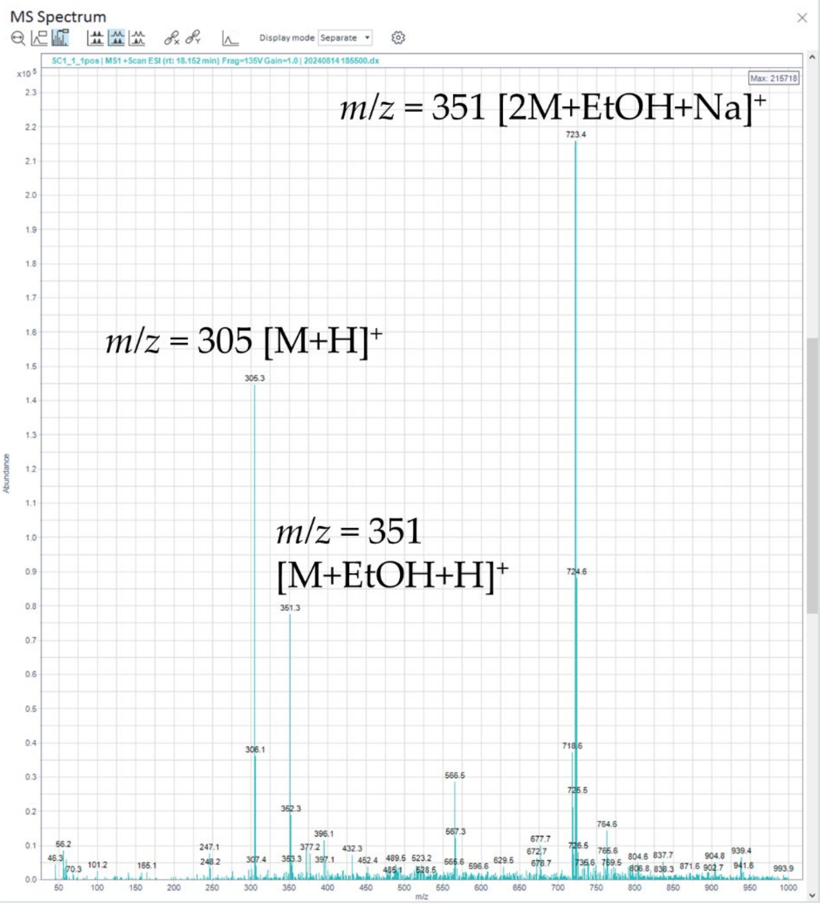

S53. Profiling data (UV chromatogram and ESI-MS) of **(26)** 5,6-dimethoxy-7-phenylbenzo[de]isochromene-1,3-dione

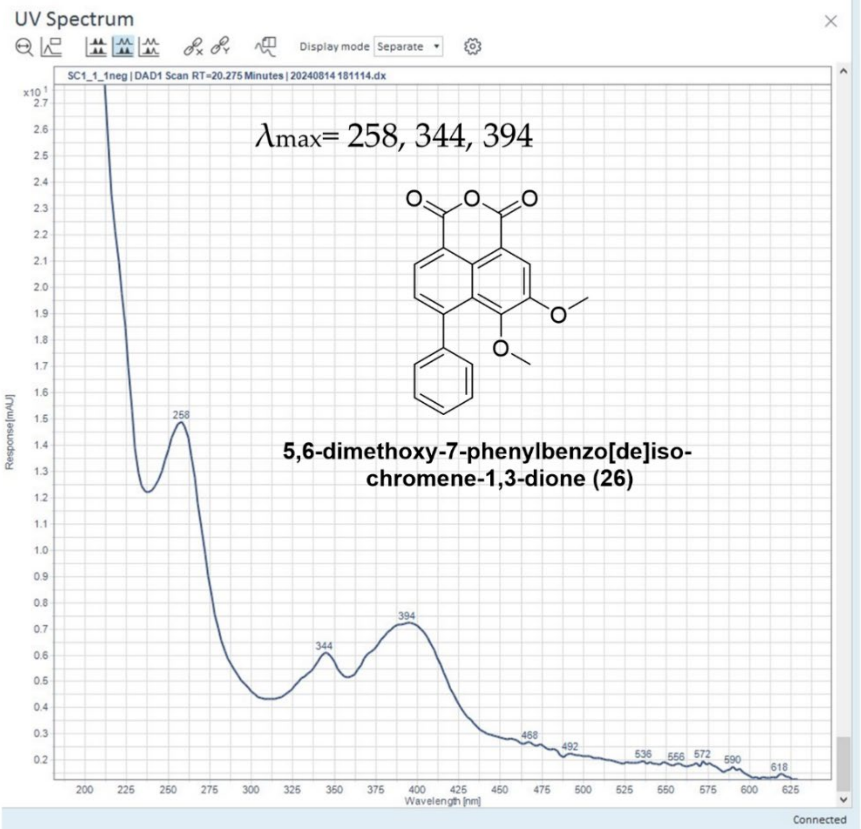

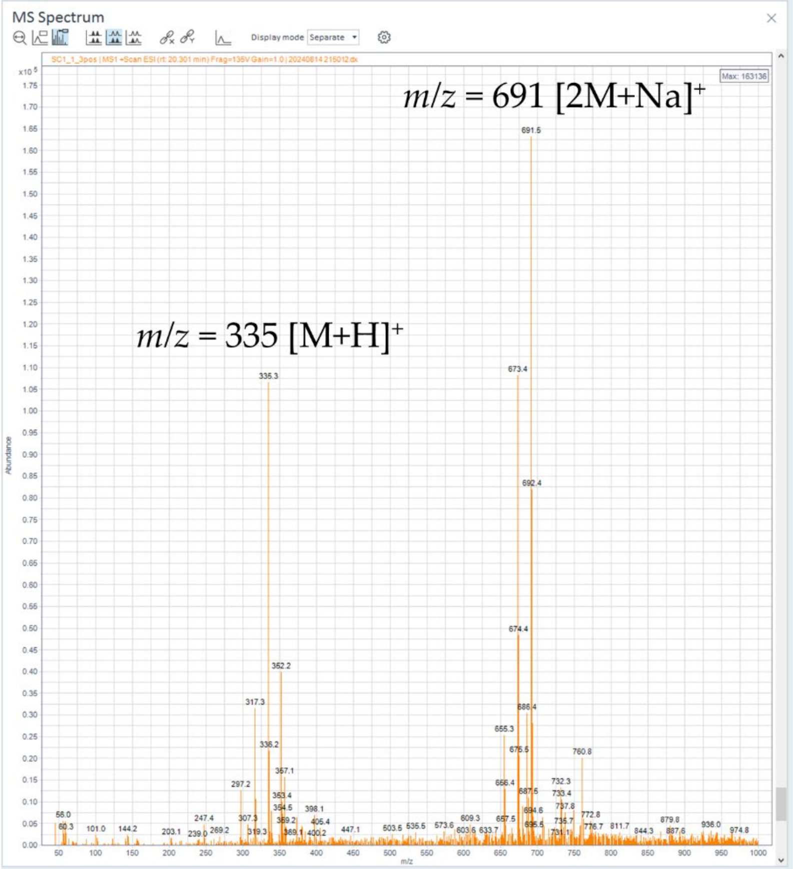

S54. Profiling data (UV chromatogram and ESI-MS) of **(27)** Haemodorone

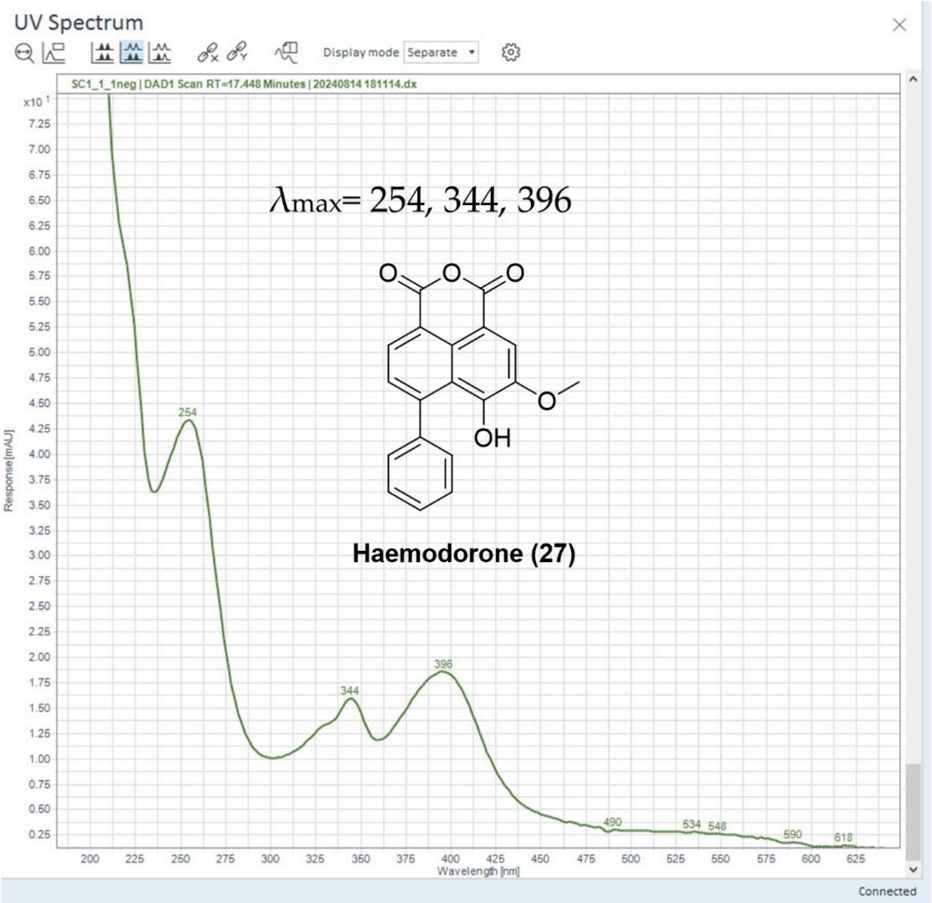

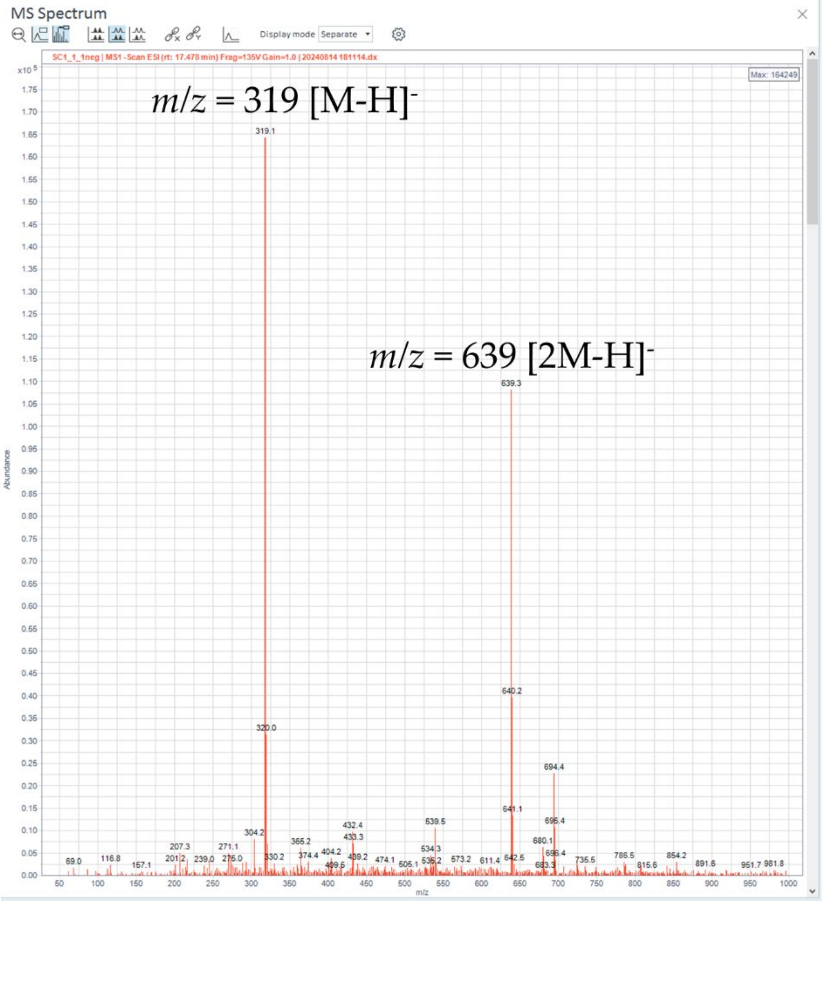

S55. Profiling data (UV chromatogram and ESI-MS) of **(28)** (6-((2-((2,5-dimethoxy-1-oxo-7-phenyl-phenalen-6-yl)oxy)-4,5-dihydroxy-6-(hydroxymethyl)tetrahydro-pyran-3-yl)oxy)-3,4,5-trihydroxytetrahydro-pyran-2-yl)methyl (E)-3-(3,4-dihydroxyphenyl)acrylate

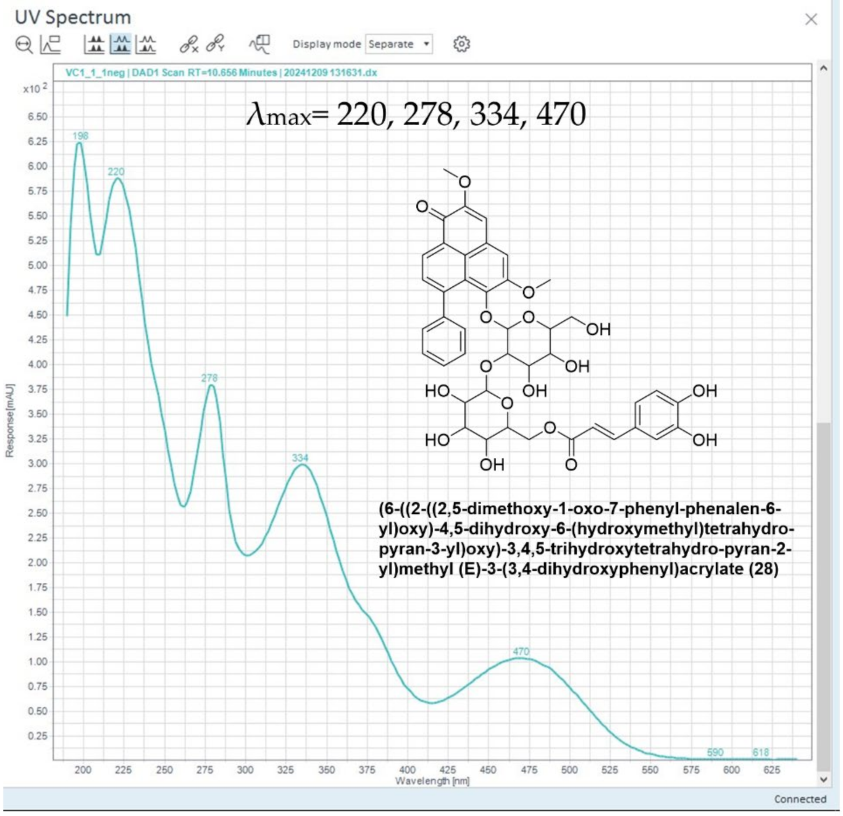

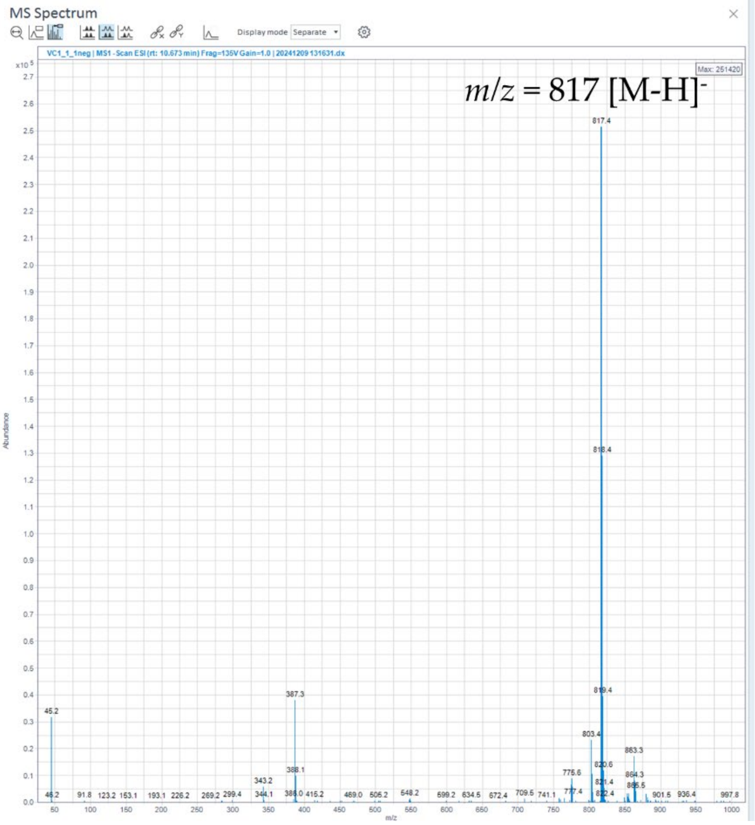

S56. Profiling data (UV chromatogram and ESI-MS) of **(29)** P-hydroxycinnamate of salipurposide

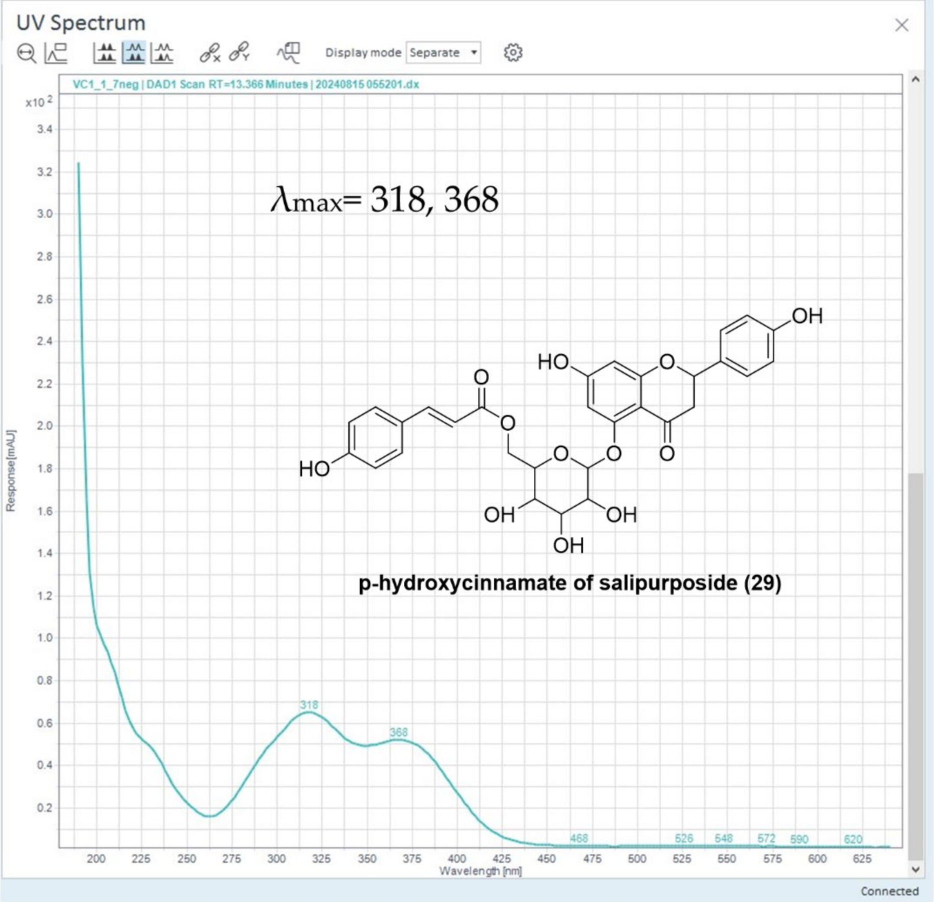

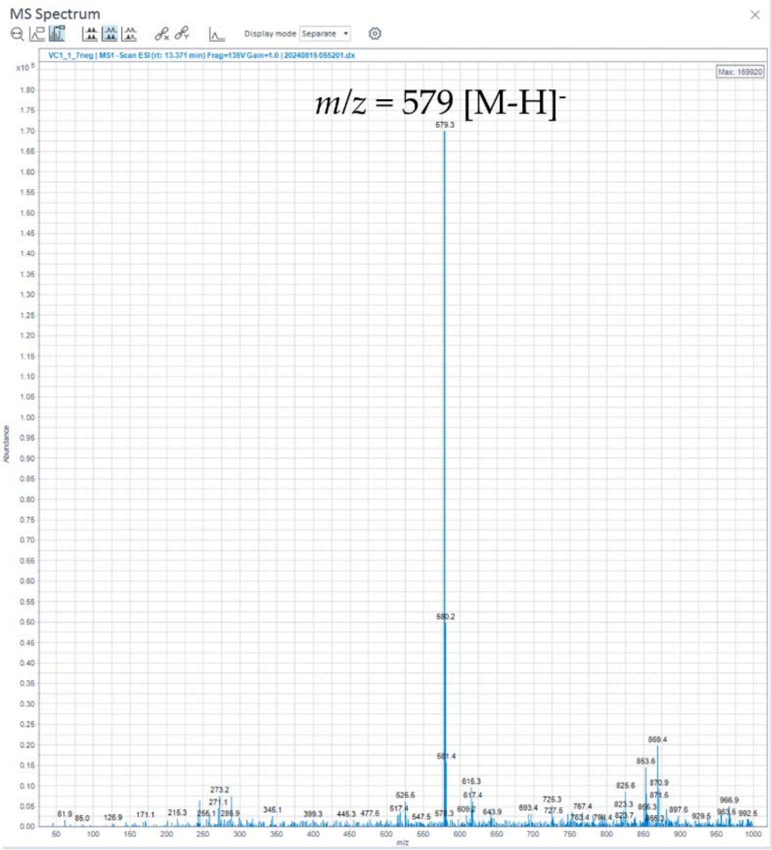

S57. Profiling data (UV chromatogram and ESI-MS) of **(30)** Fulginosin A

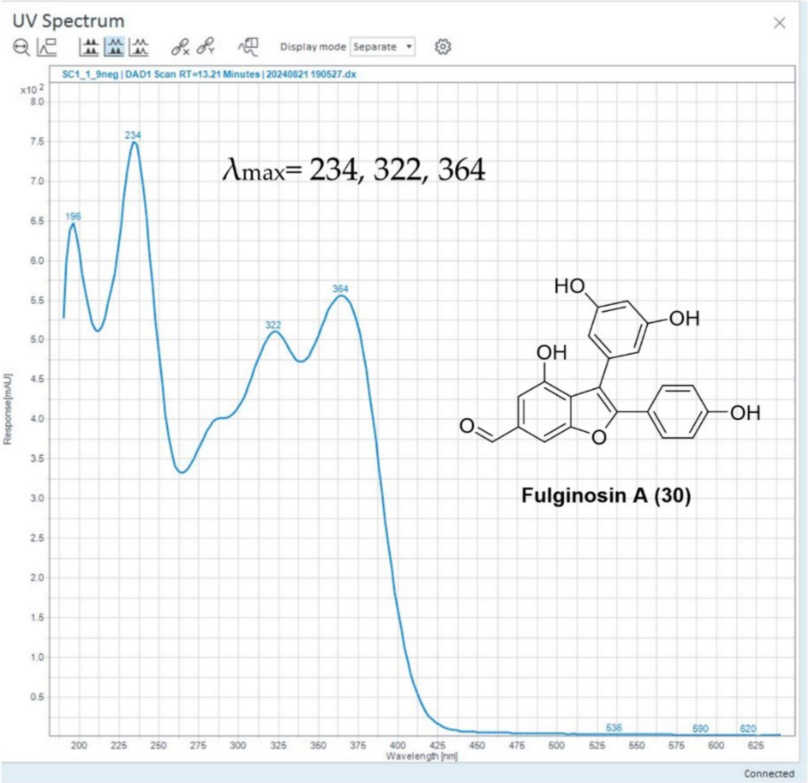

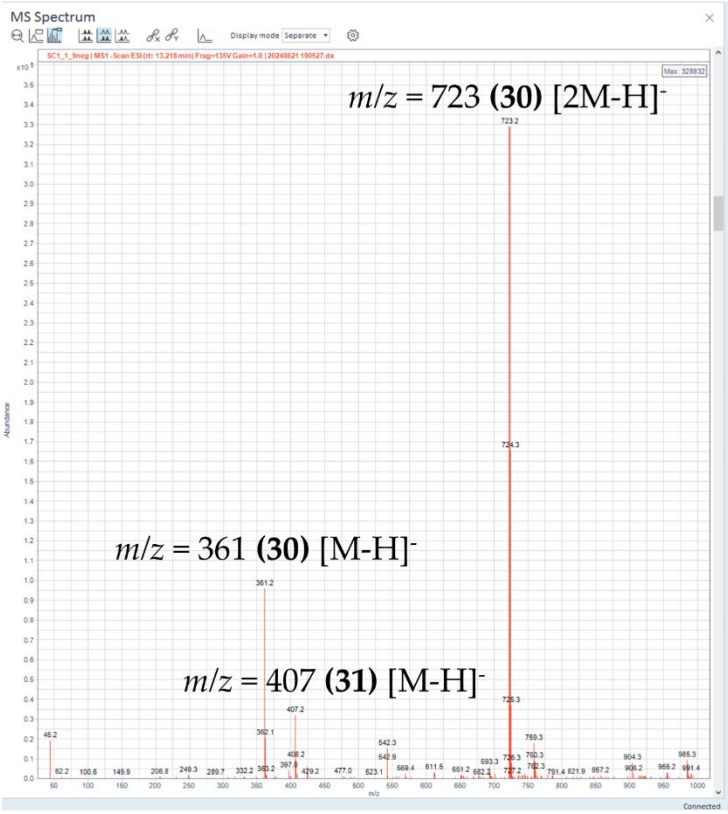

S58. Profiling data (UV chromatogram and ESI-MS) of **(31)** Fulginosin B

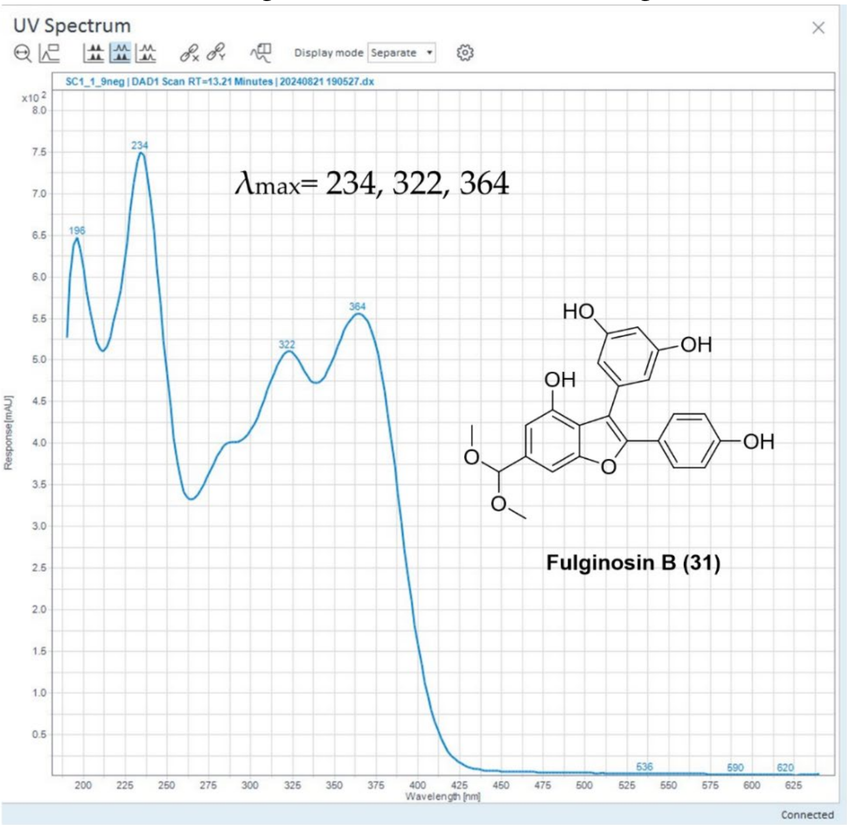

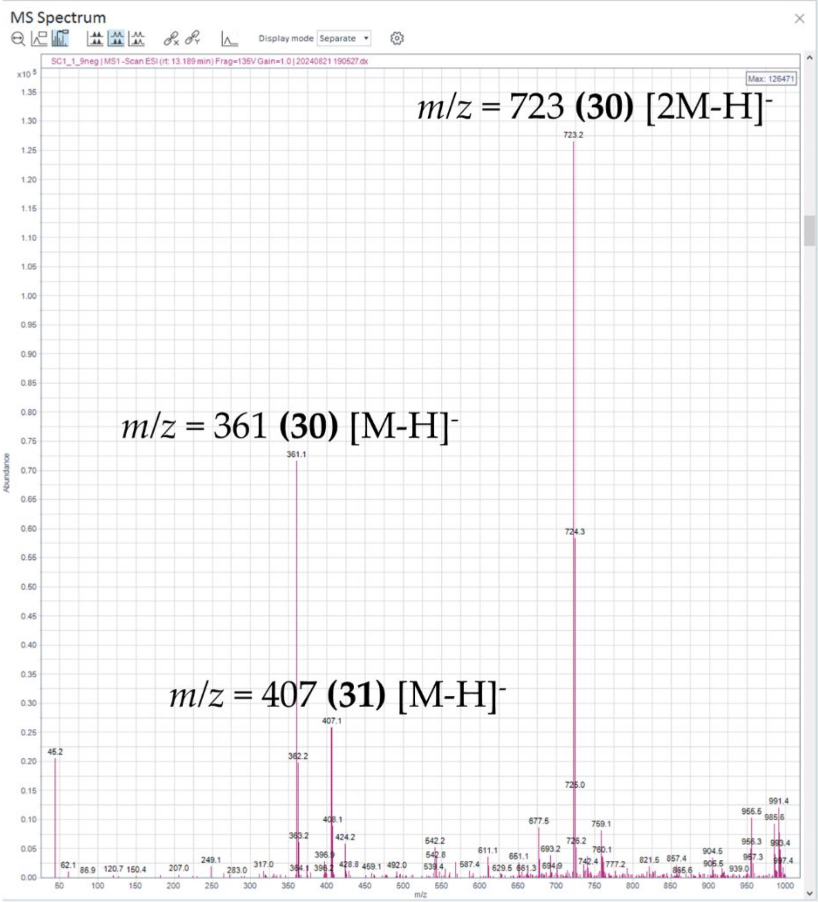

S59. Profiling data (UV chromatogram and ESI-MS) of **(32)** Angiopressin A

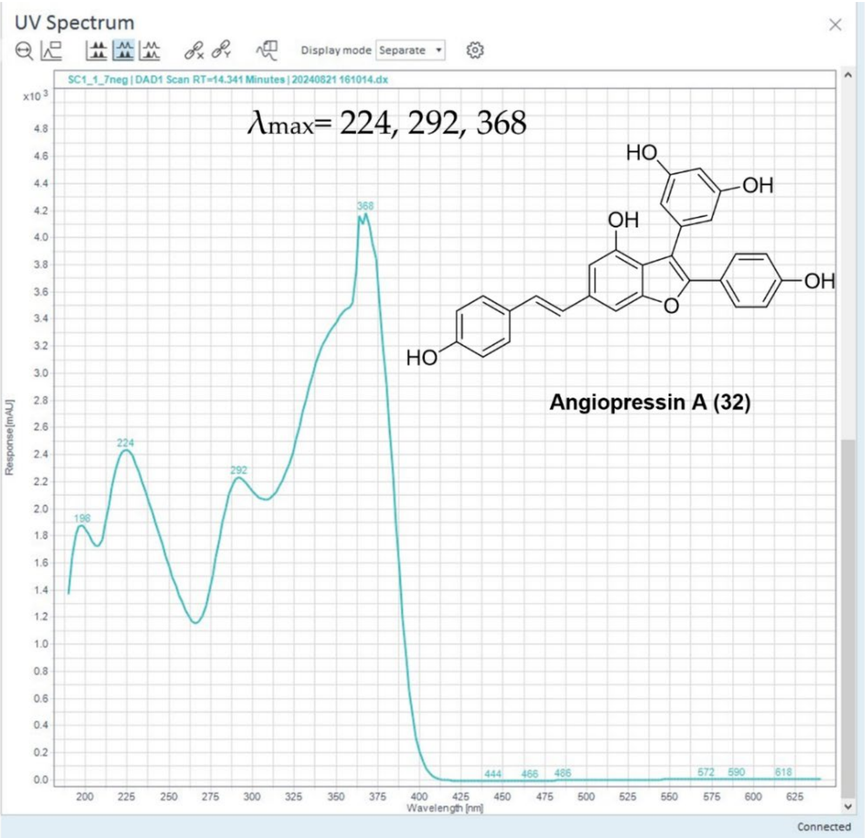

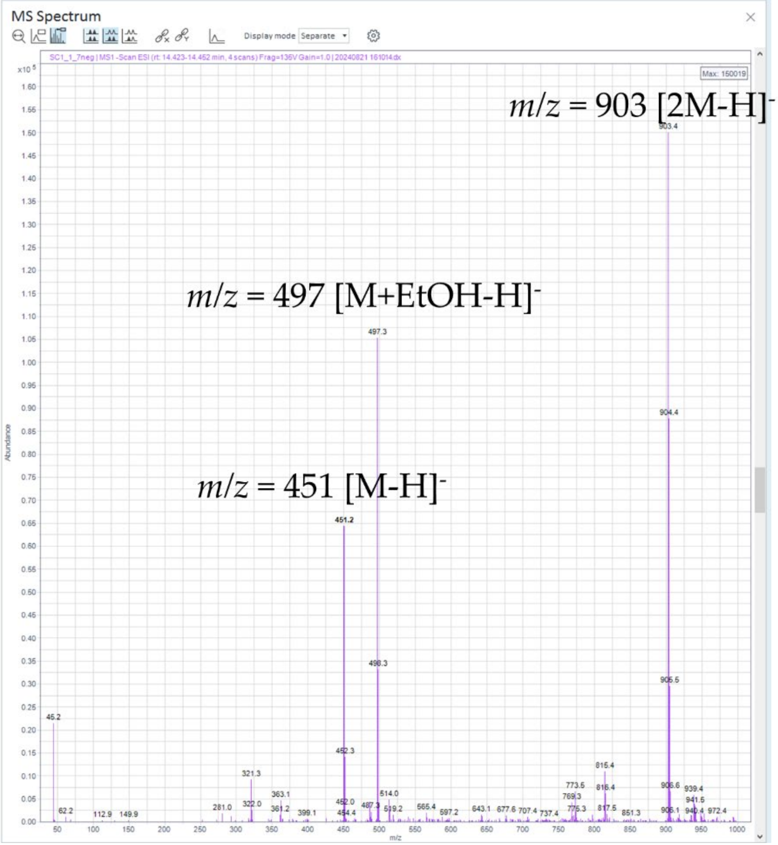

S60. Profiling data (UV chromatogram and ESI-MS) of **(33)** 2-phenylnaphthalic anhydride

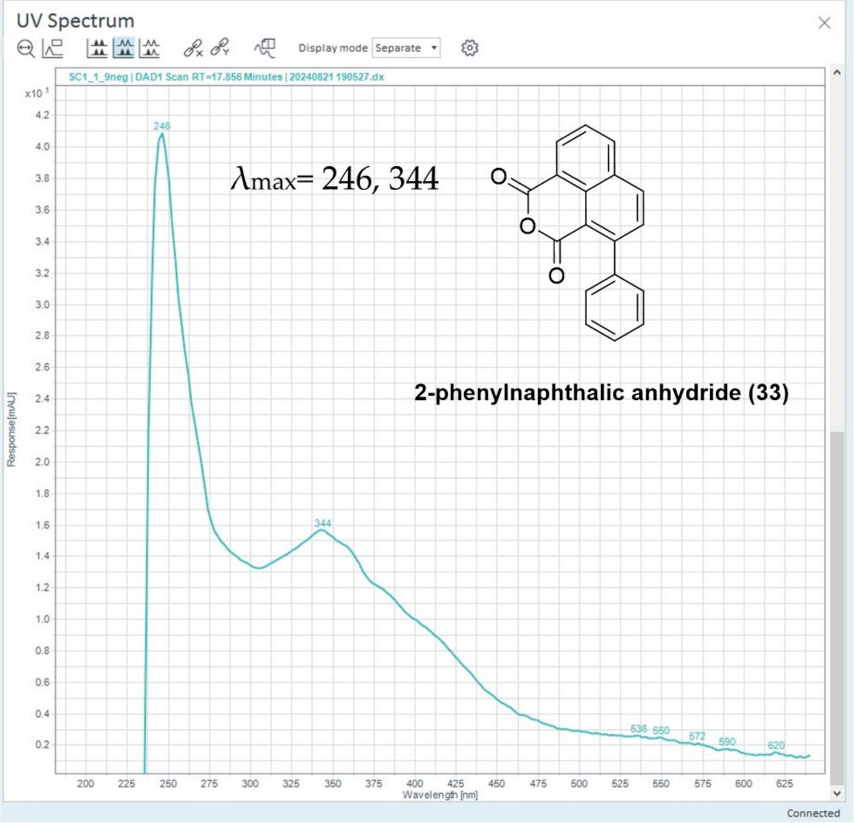

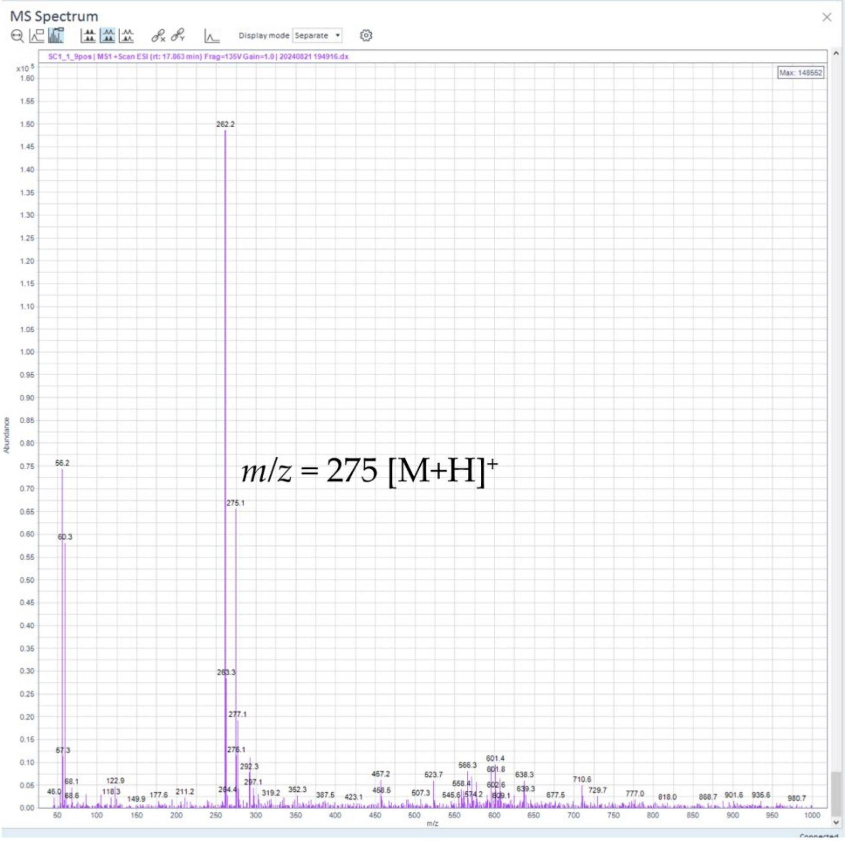

S61. Profiling data (UV chromatogram and ESI-MS) of **(34)** Rutin

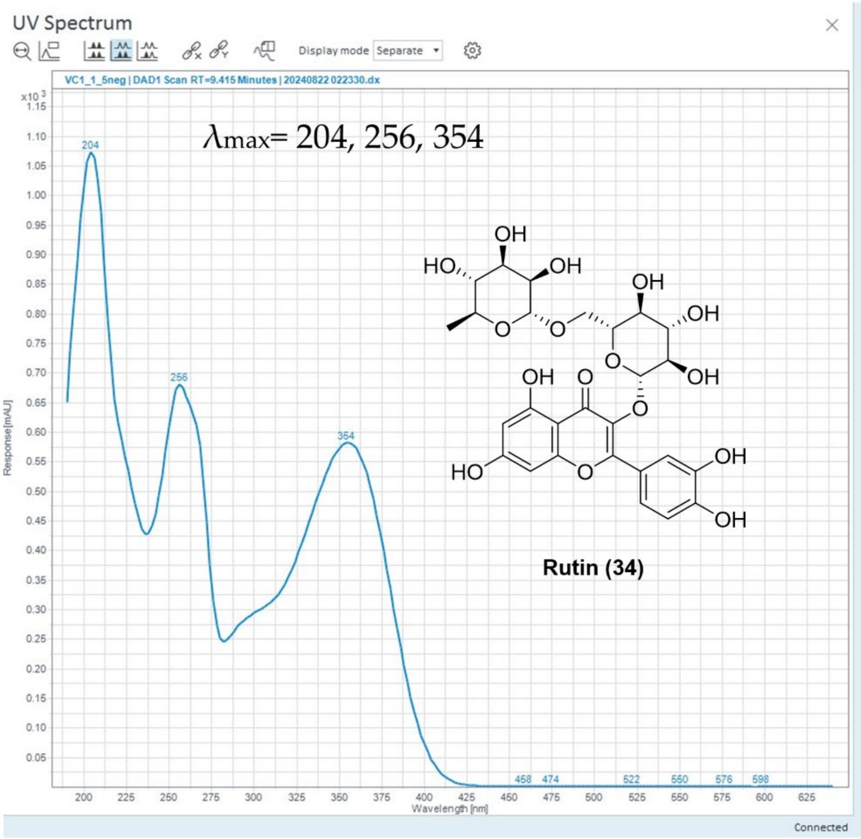

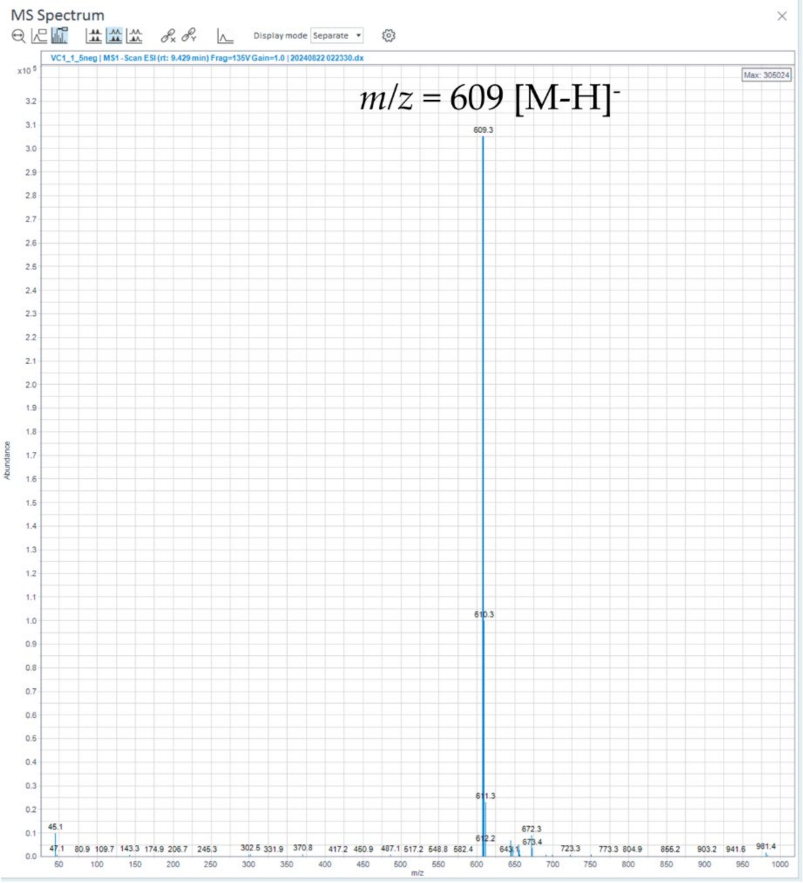

S62. +HRLC(ESI)MS spectrum of 2005_01a Peak 1

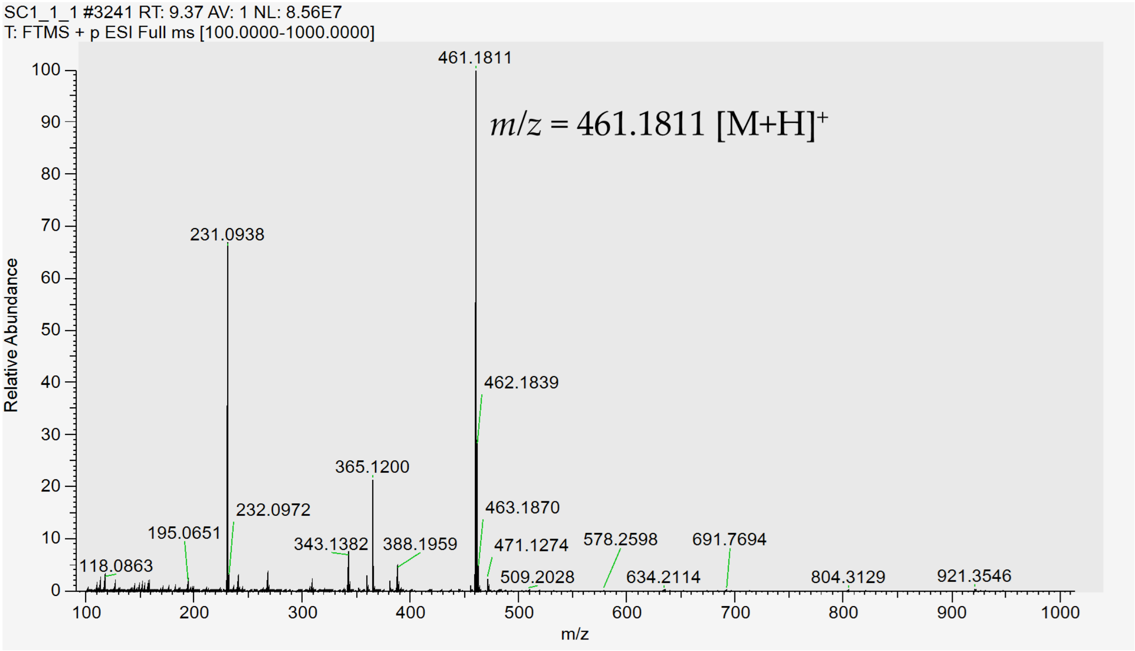

S63. +HRLC(ESI)MS spectrum of 2005_01a Peak 2

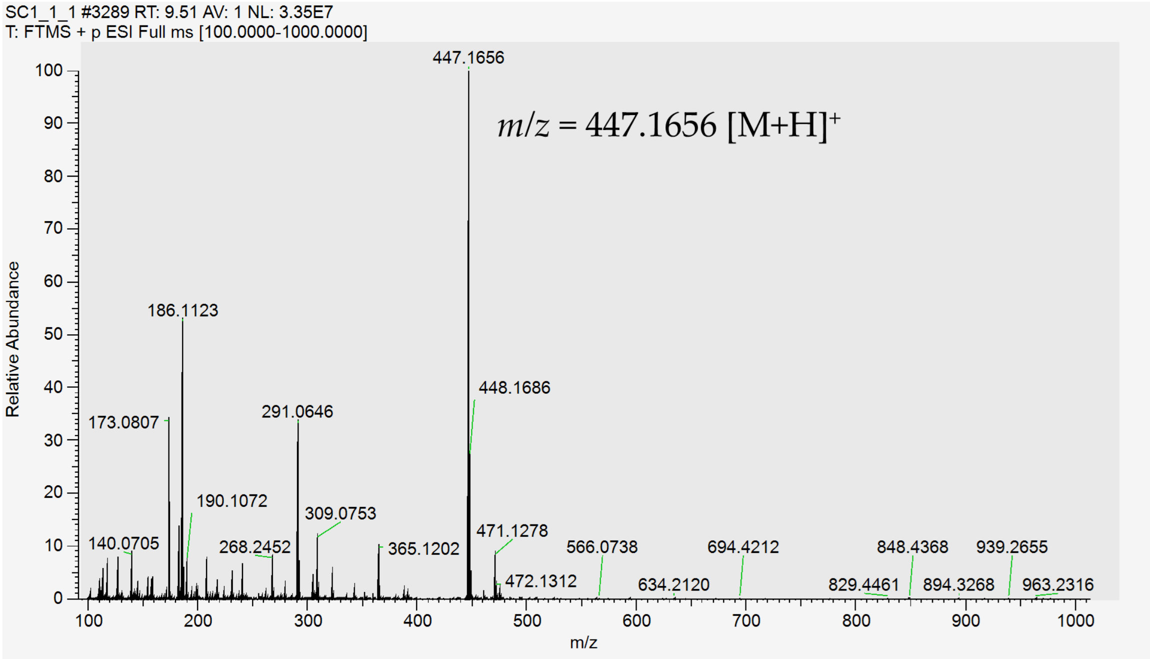

S64. +HRLC(ESI)MS spectrum of 2005_01a Peak 3

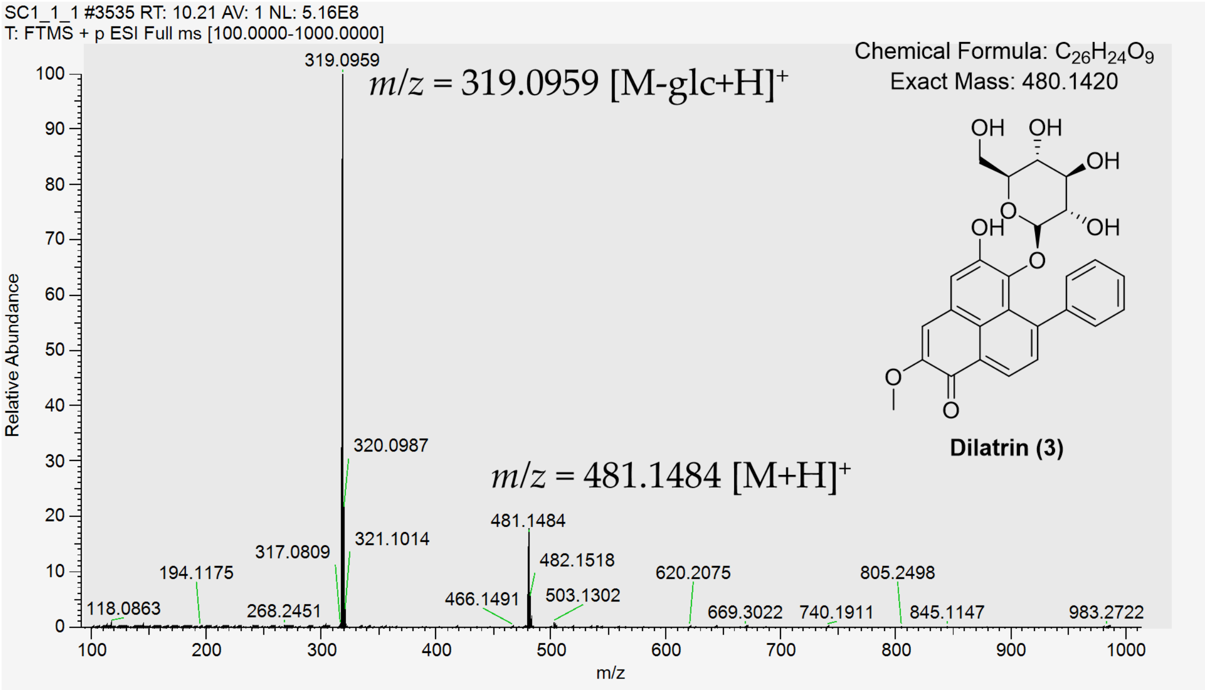

S65. +HRLC(ESI)MS spectrum of 2005_01a Peak 4

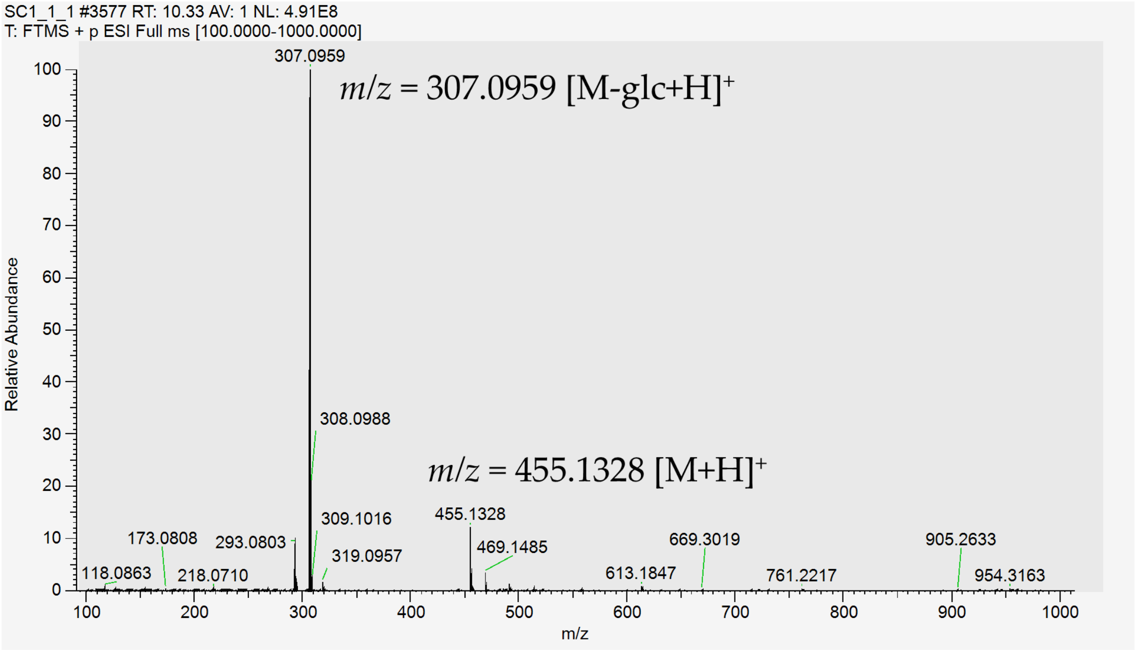

S66. +HRLC(ESI)MS spectrum of 2005_01a Peak 5

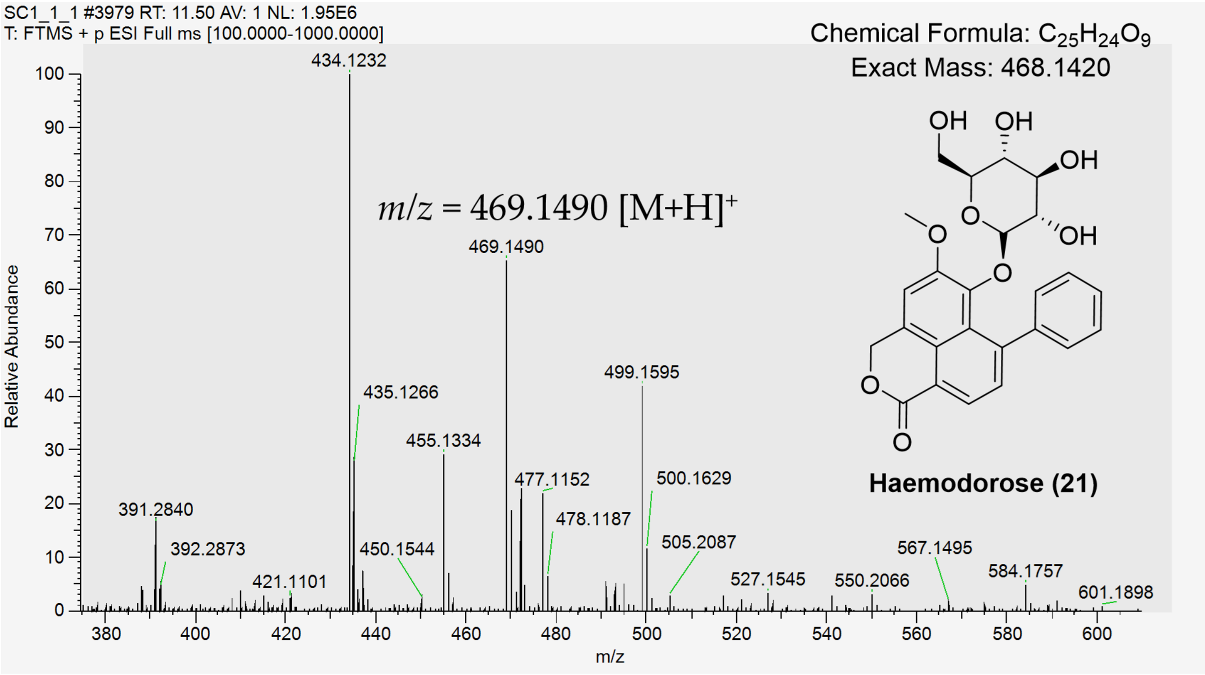

S67. +HRLC(ESI)MS spectrum of 2005_01a Peak 6

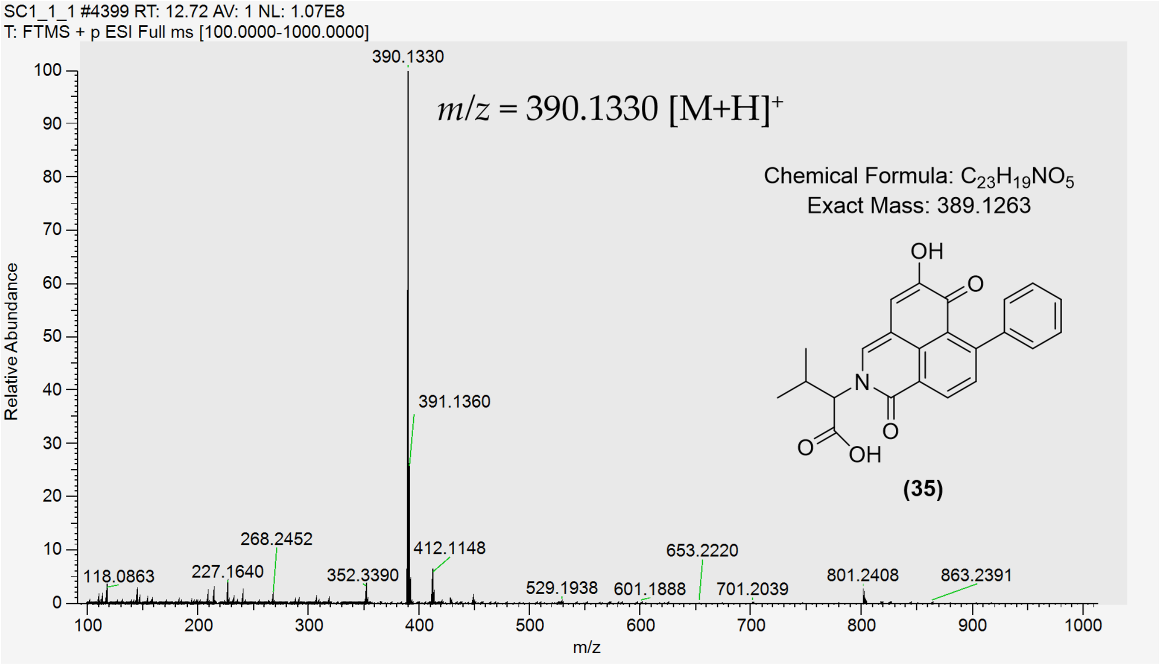

S68. +HRLC(ESI)MS spectrum of 2005_01a Peak 7

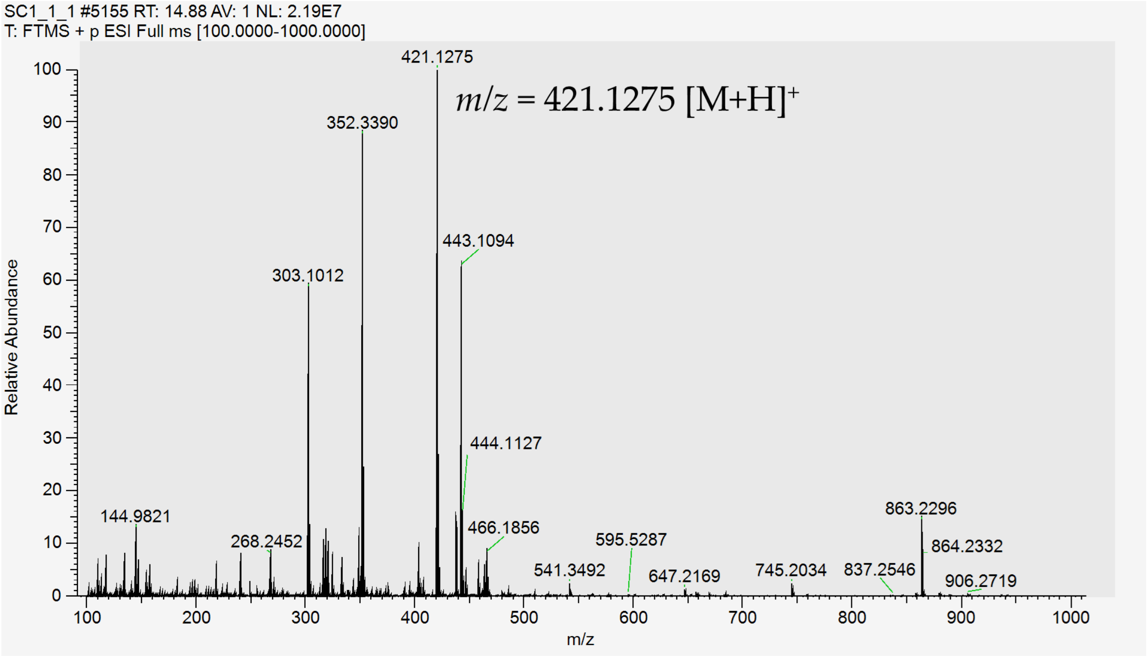

S69. +HRLC(ESI)MS spectrum of 2005_01a Peak 8

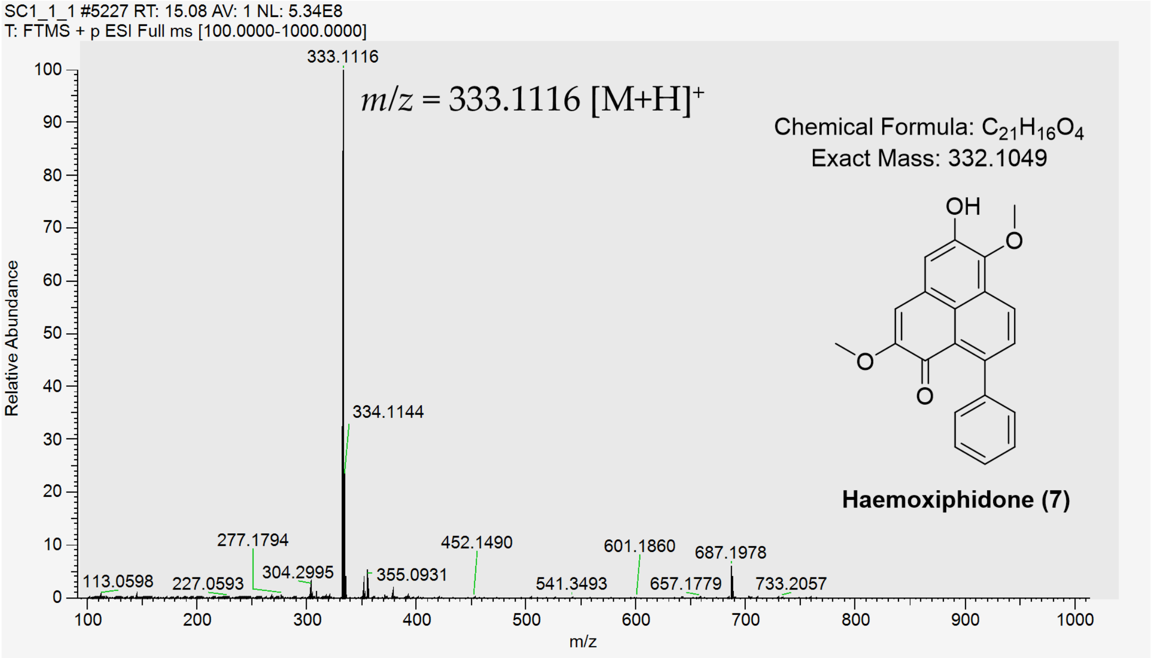

S70. +HRLC(ESI)MS spectrum of 2005_01a Peak 9

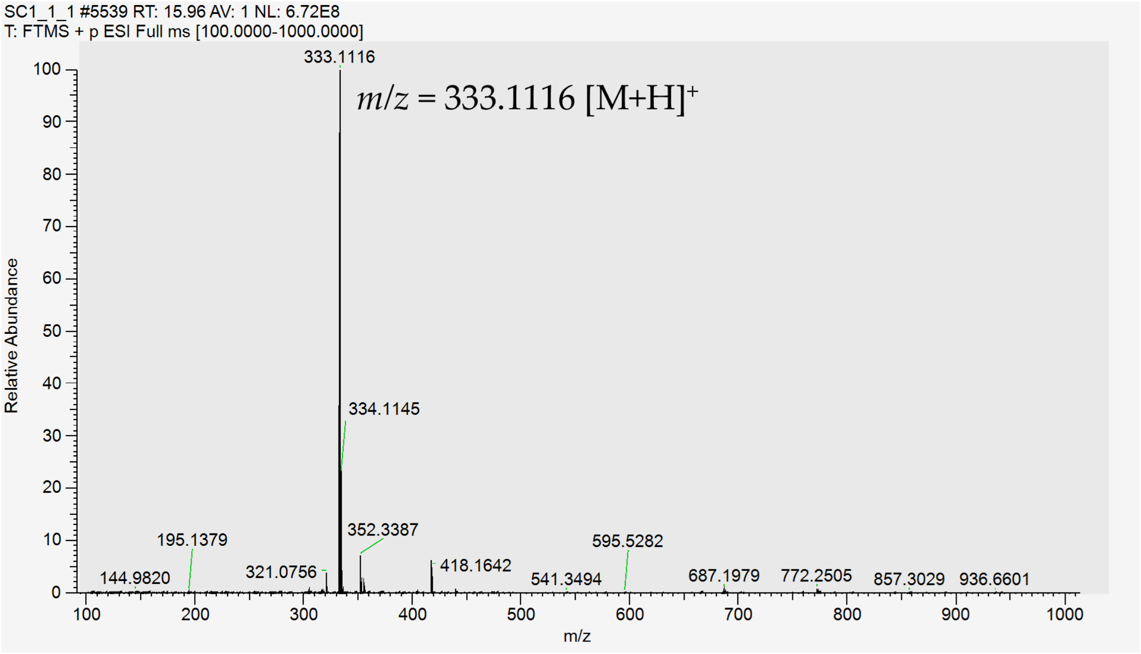

S71. +HRLC(ESI)MS spectrum of 2005_01a Peak 10

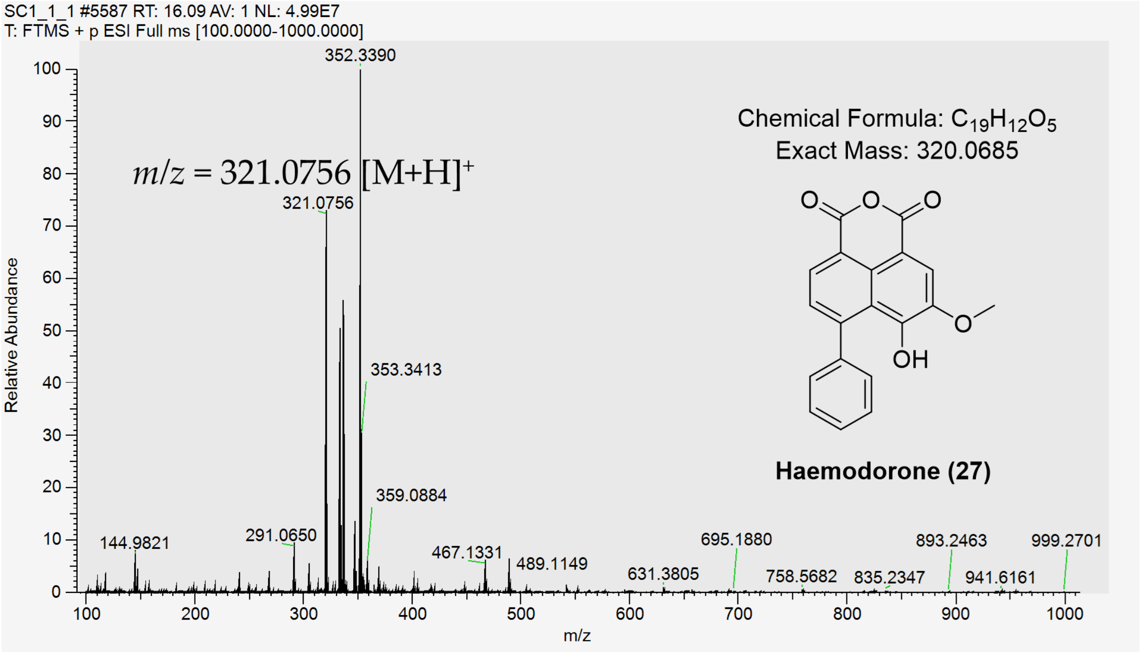

S72. +HRLC(ESI)MS spectrum of 2005_01a Peak 11

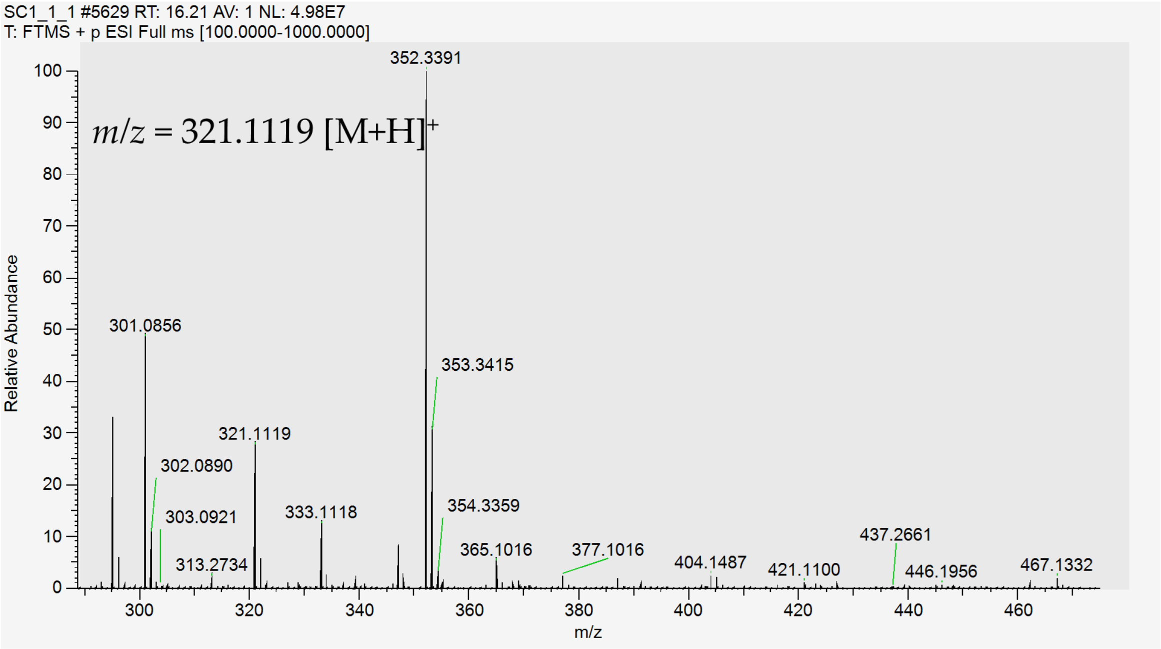

S73. +HRLC(ESI)MS spectrum of 2005_01a Peak 11a

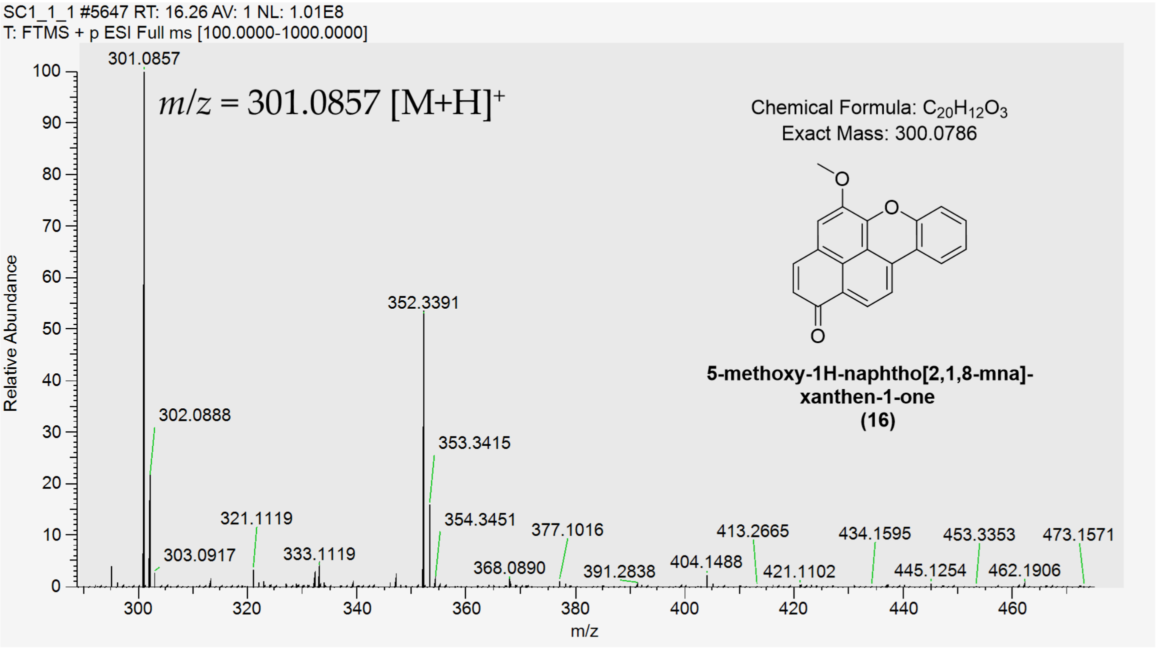

S74. +HRLC(ESI)MS spectrum of 2005_01a Peak 12

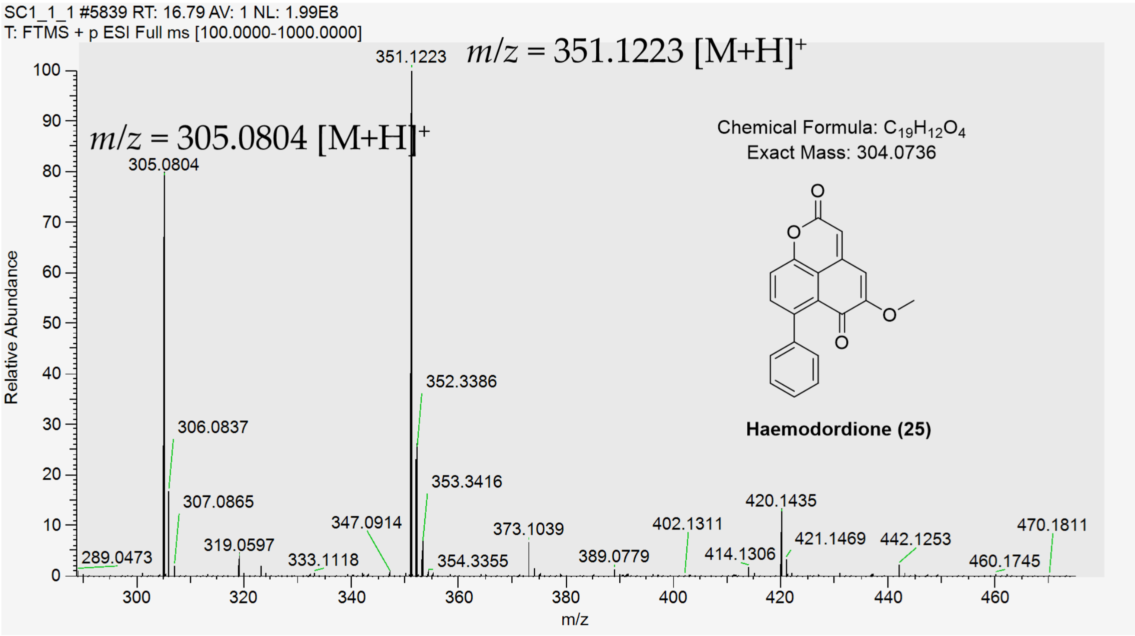

S75. +HRLC(ESI)MS spectrum of 2005_01a Peak 13

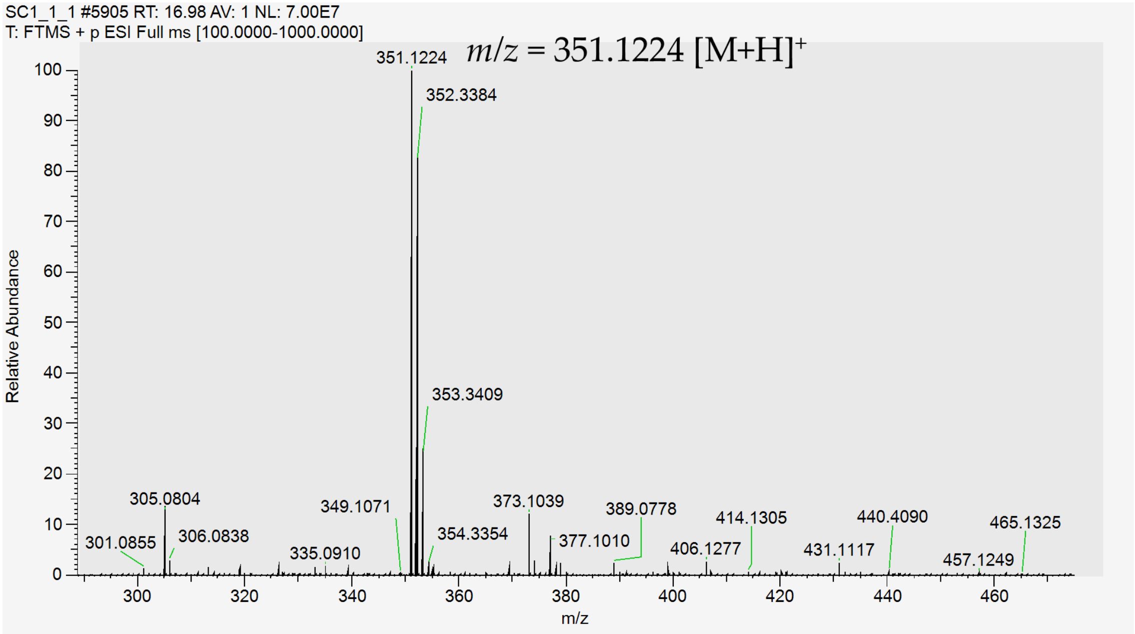

S76. +HRLC(ESI)MS spectrum of 2005_01a Peak 14

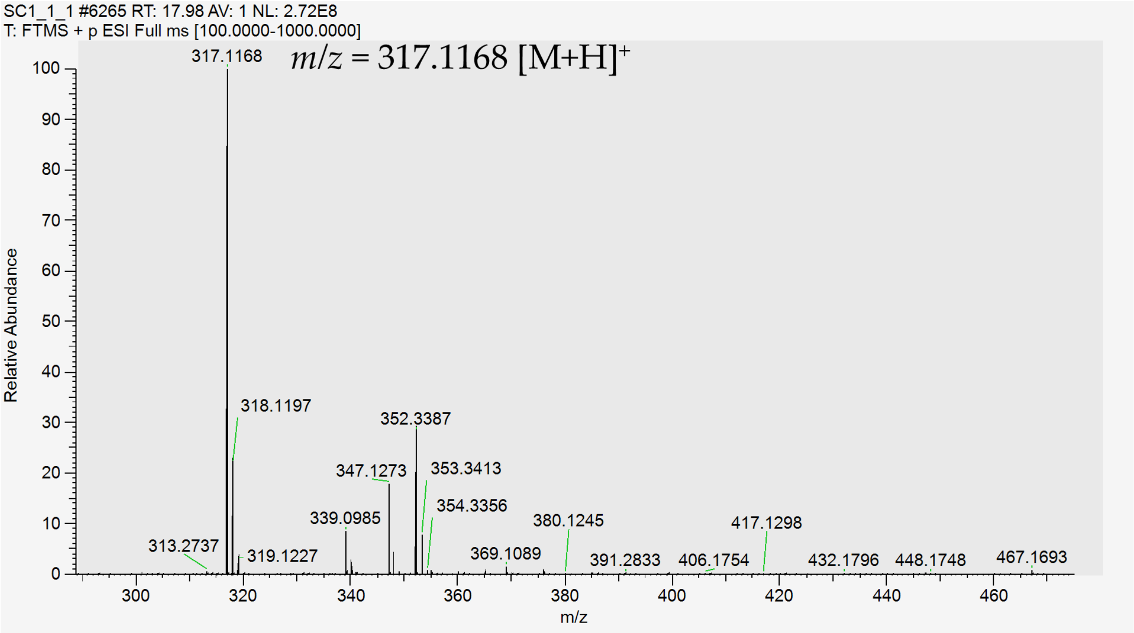

S77. +HRLC(ESI)MS spectrum of 2005_01a Peak 15

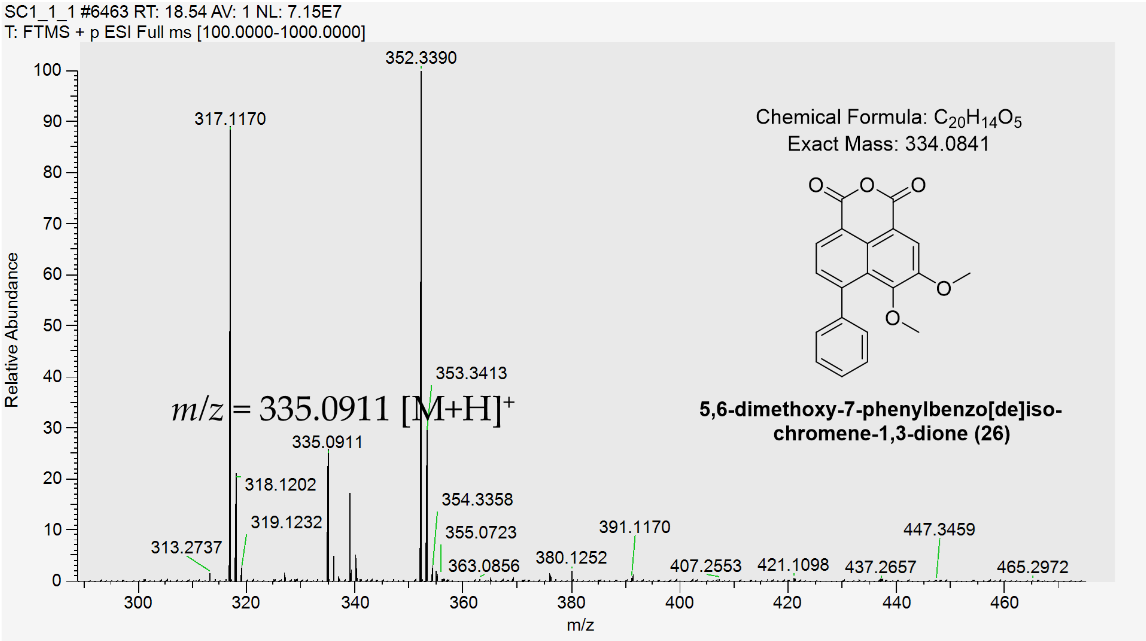

S78. +HRLC(ESI)MS spectrum of 2010_17b Peak 1

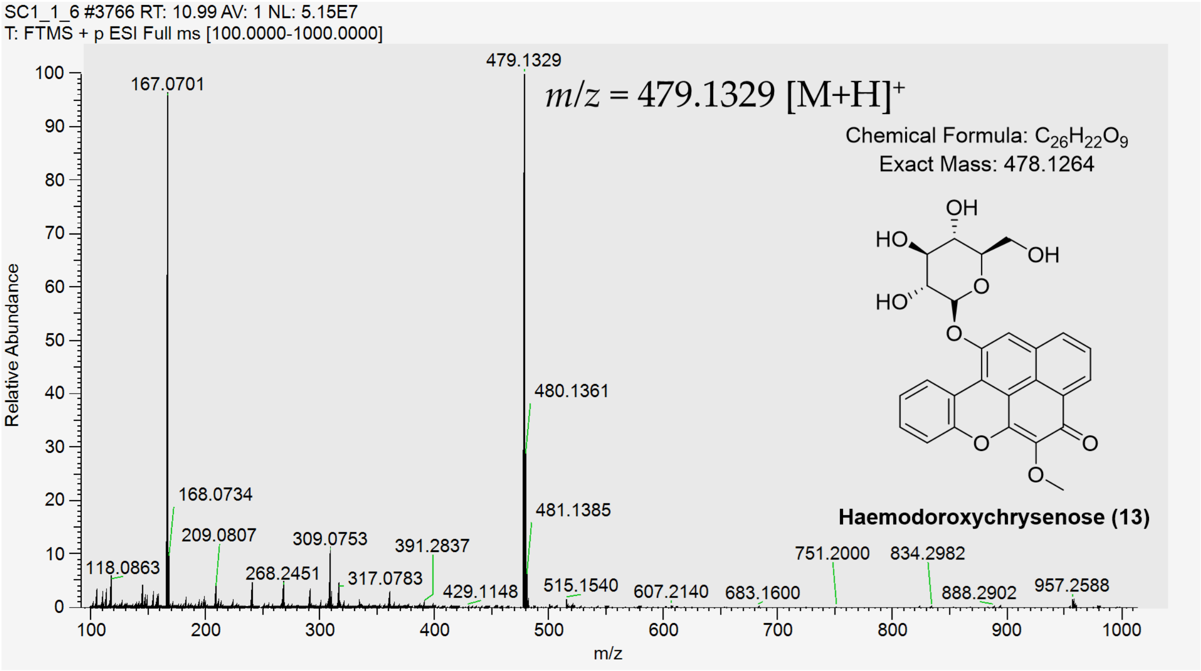

S79. +HRLC(ESI)MS spectrum of 2010_17b Peak 2

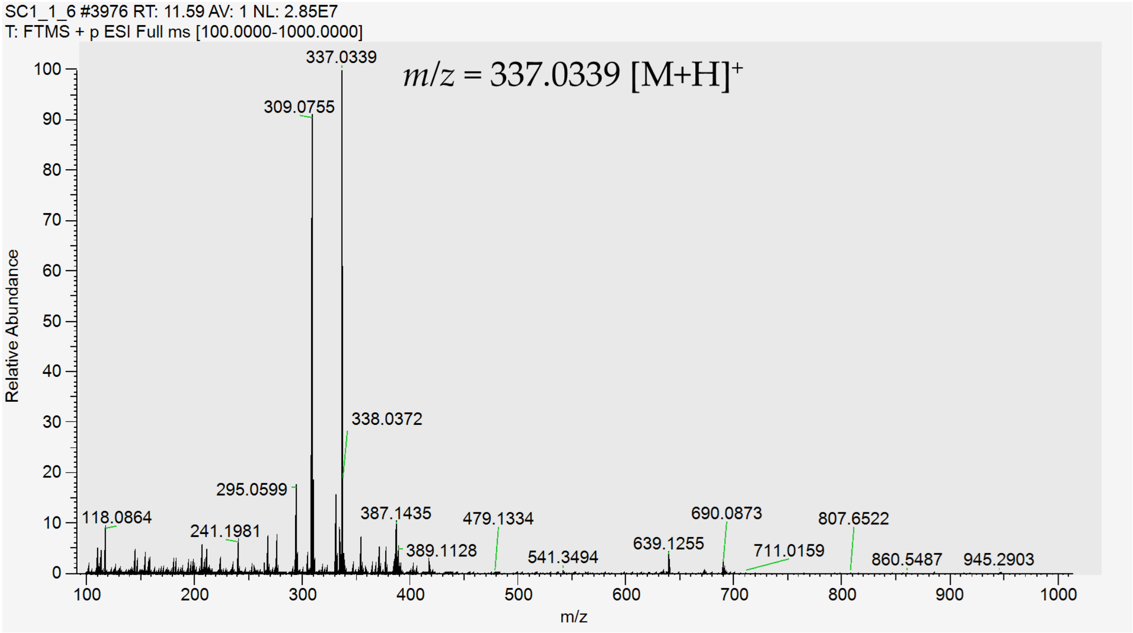

S80. +HRLC(ESI)MS spectrum of 2010_17b Peak 3

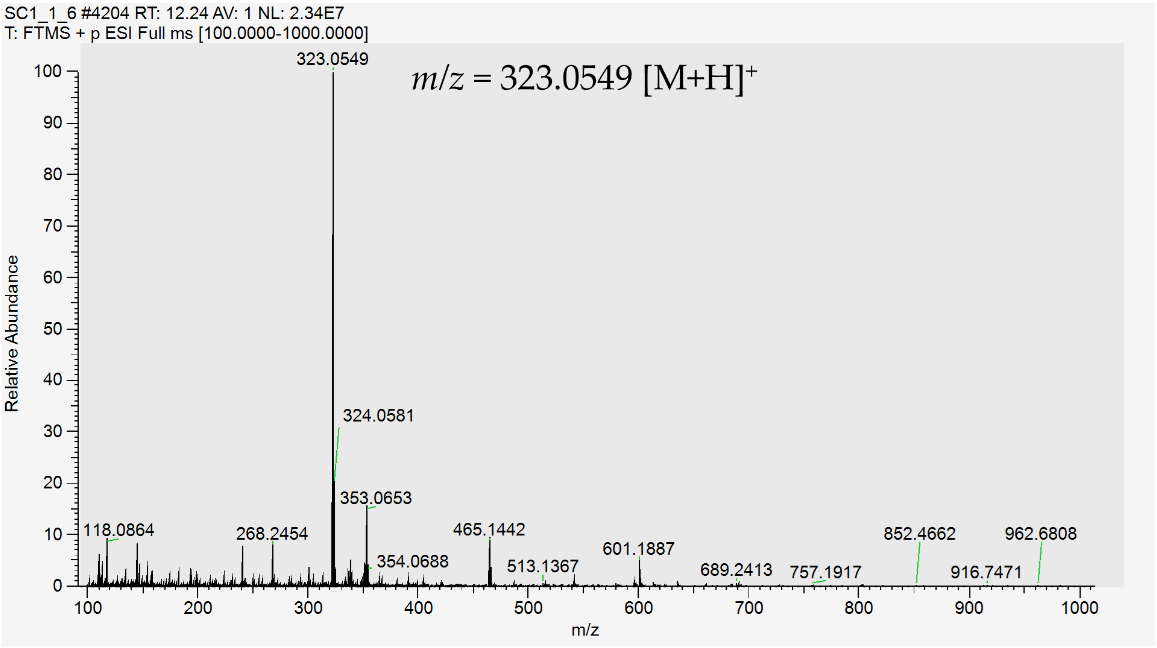

S81. +HRLC(ESI)MS spectrum of 2010_17b Peak 4

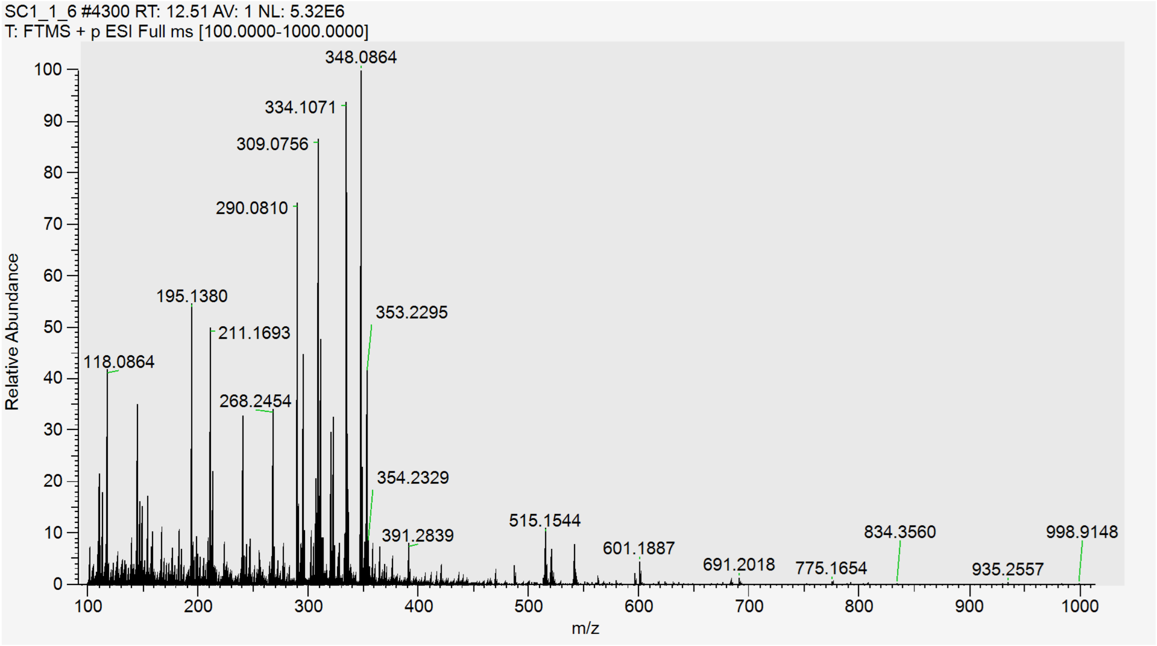

S82. +HRLC(ESI)MS spectrum of 2010_17b Peak 5

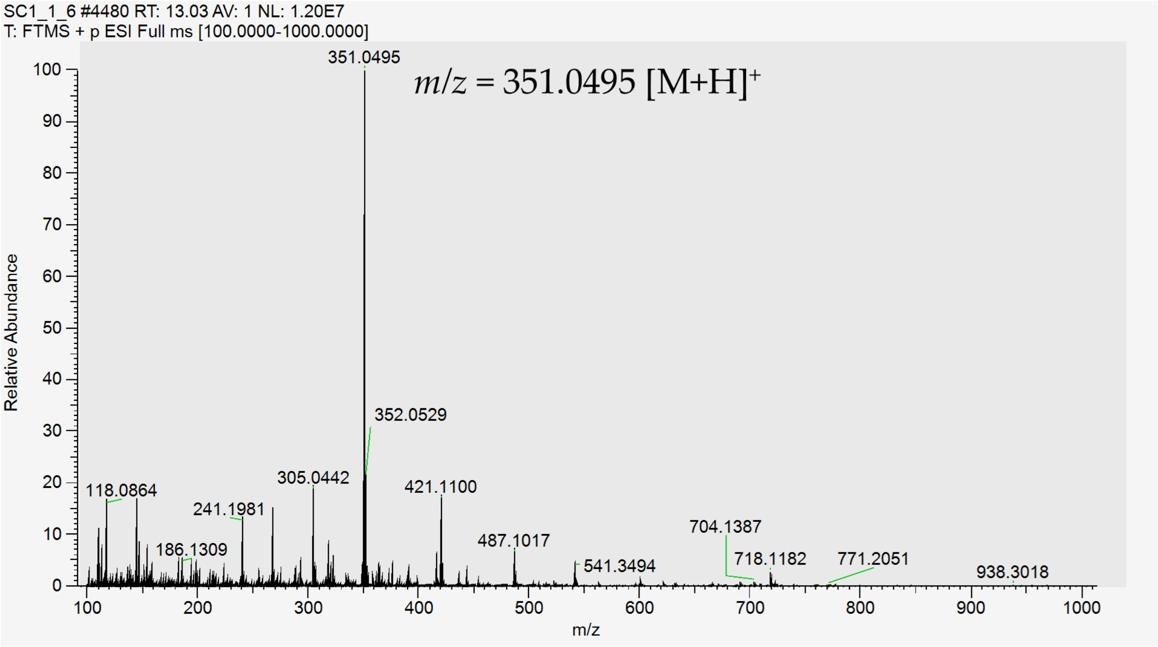

S83. +HRLC(ESI)MS spectrum of 2010_17b Peak 6

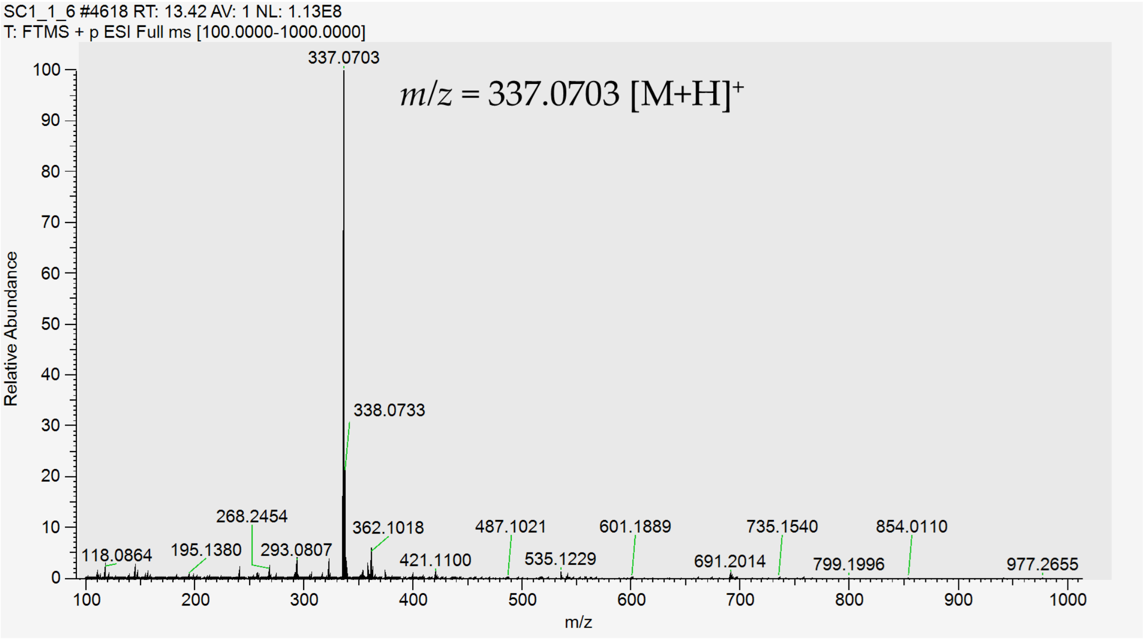

S84. +HRLC(ESI)MS spectrum of 2010_17b Peak 7

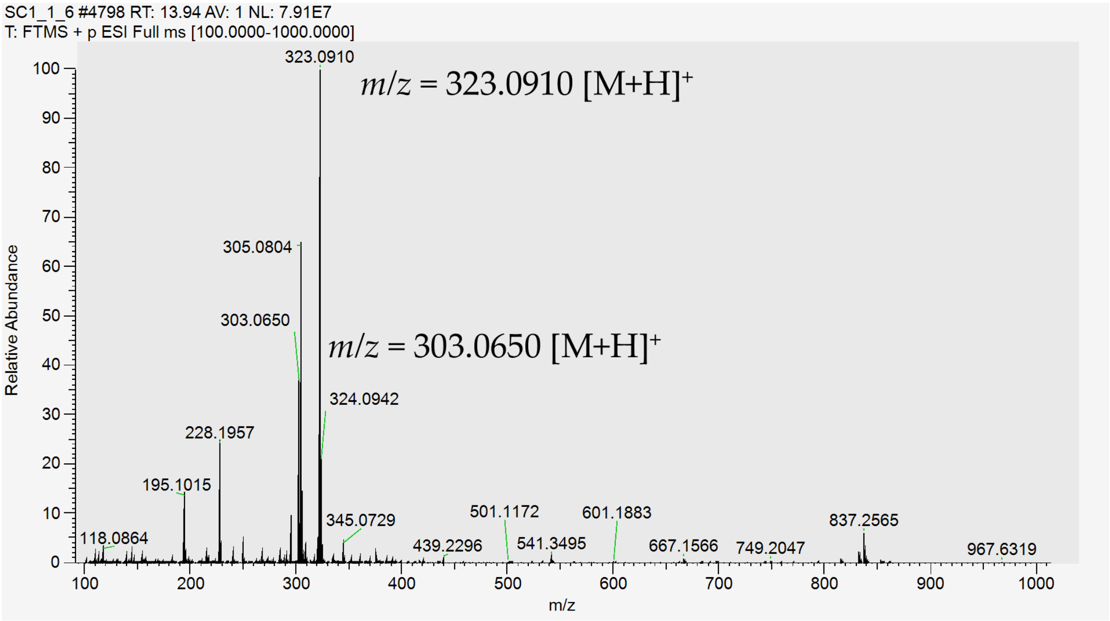

S85. +HRLC(ESI)MS spectrum of 2010_17b Peak 8

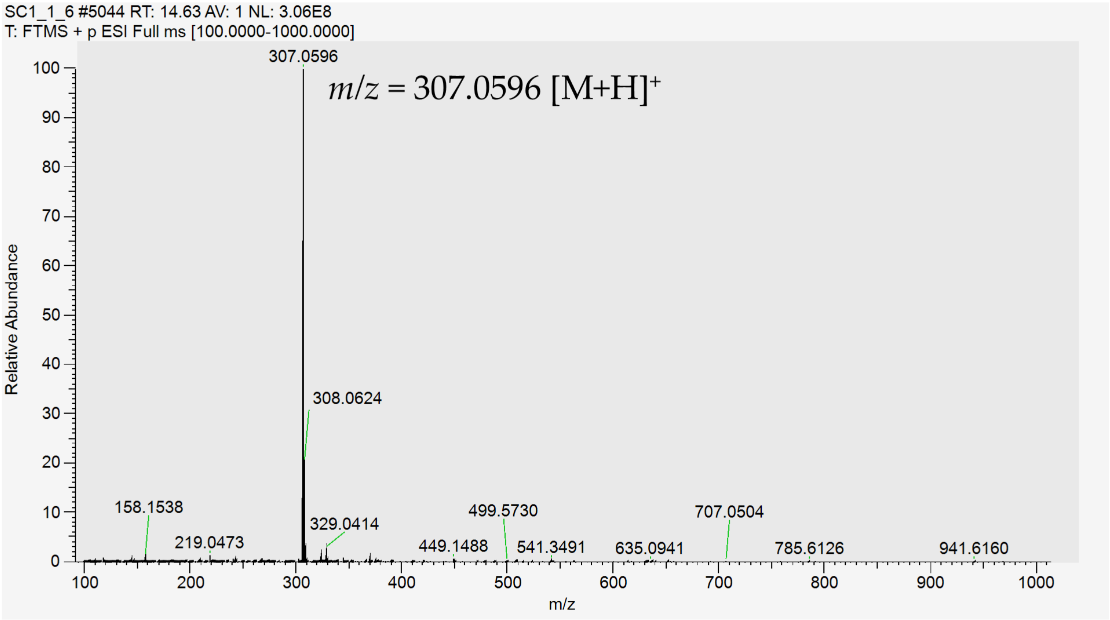

S86. +HRLC(ESI)MS spectrum of 2010_17b Peak 9

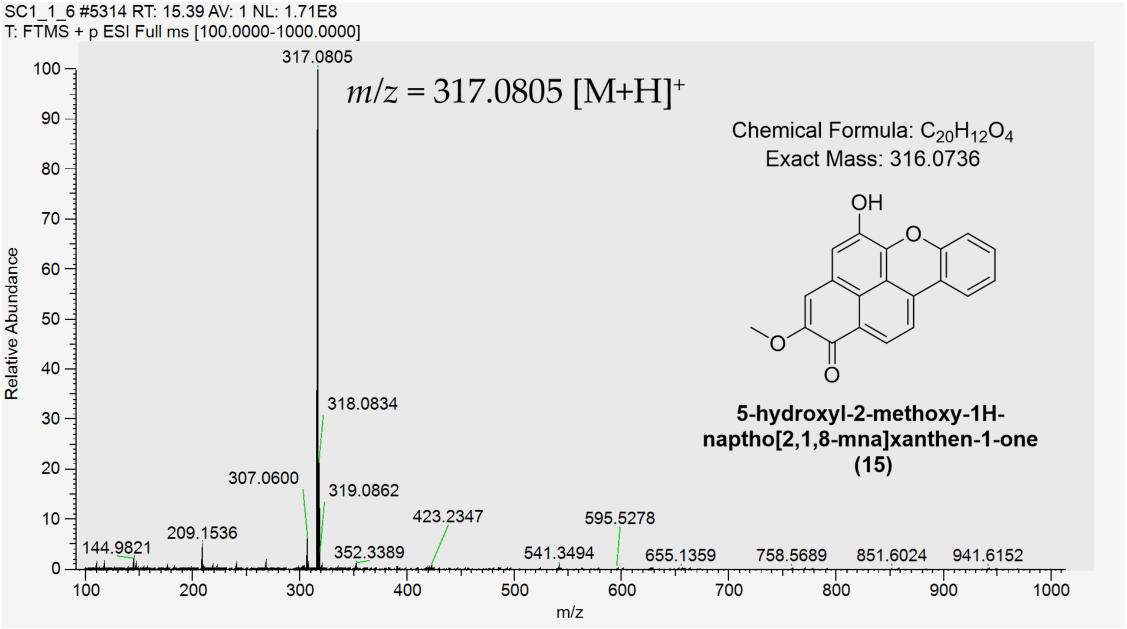

S87. +HRLC(ESI)MS spectrum of 2010_17b Peak 10

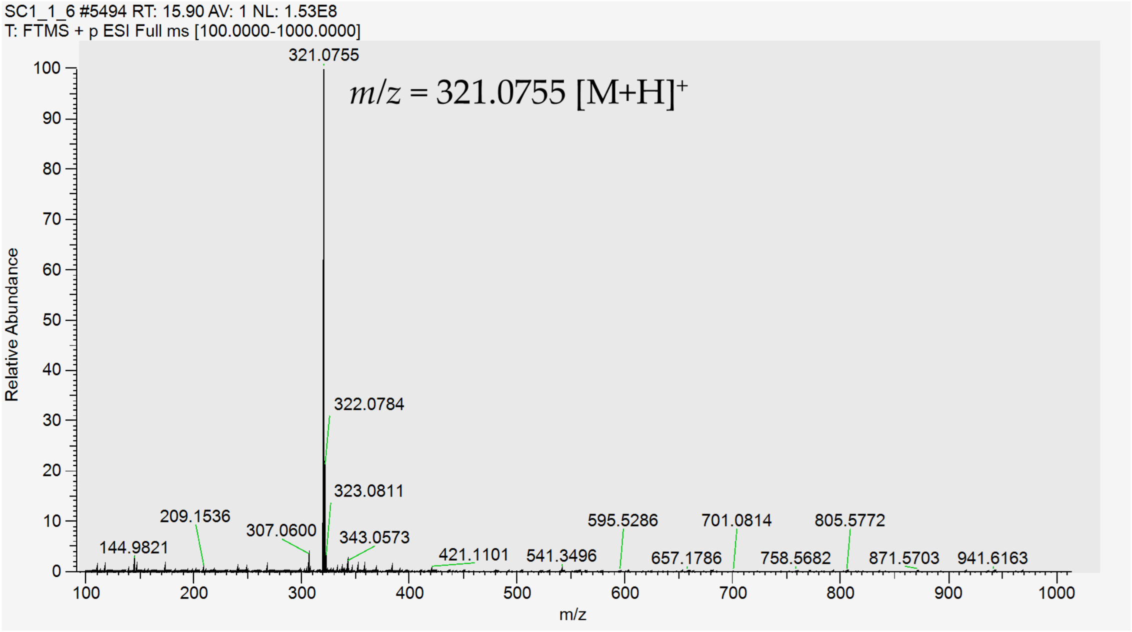

S88. +HRLC(ESI)MS spectrum of 2023_01a Peak 2

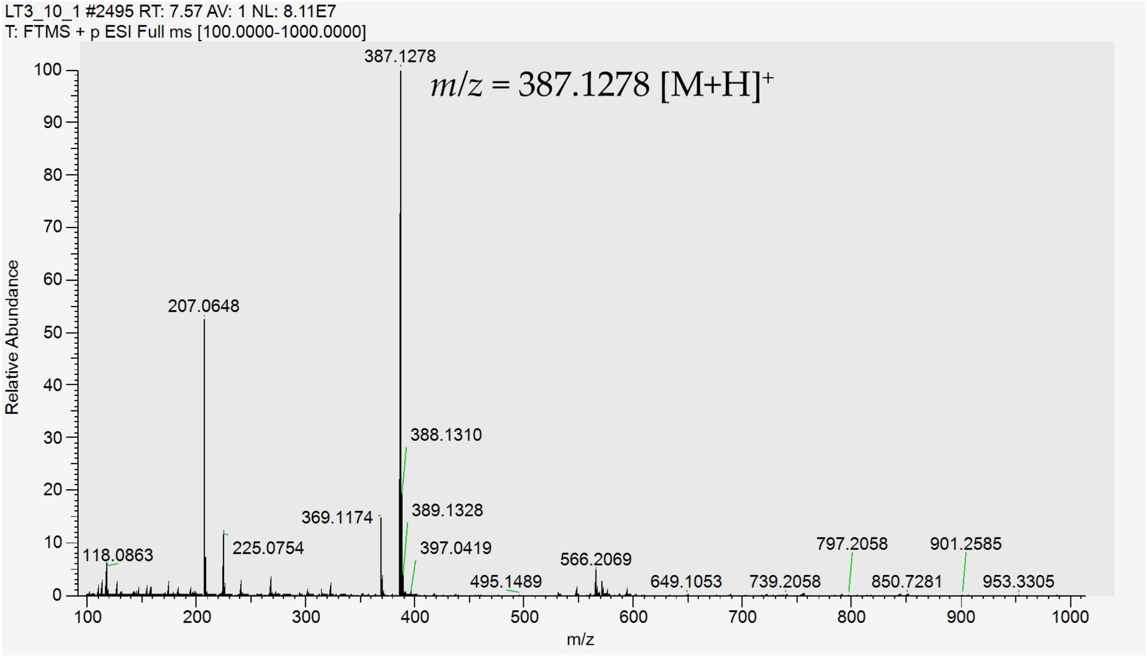

S89. +HRLC(ESI)MS spectrum of 2023_01a Peak 3

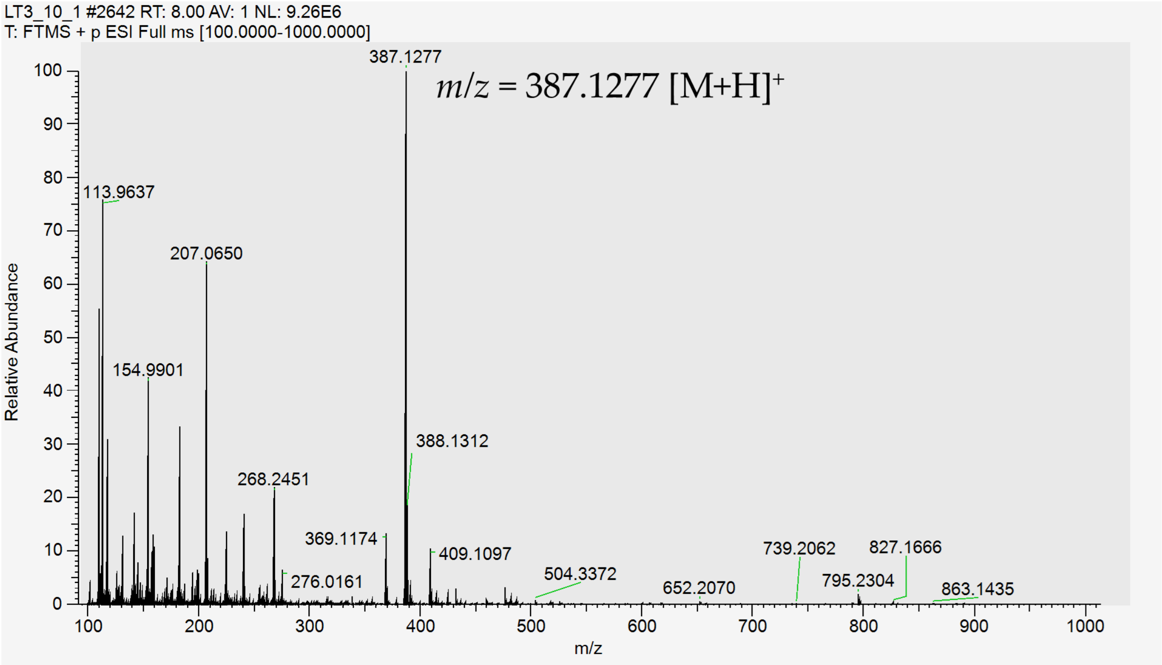

S90. +HRLC(ESI)MS spectrum of 2023_01a Peak 6

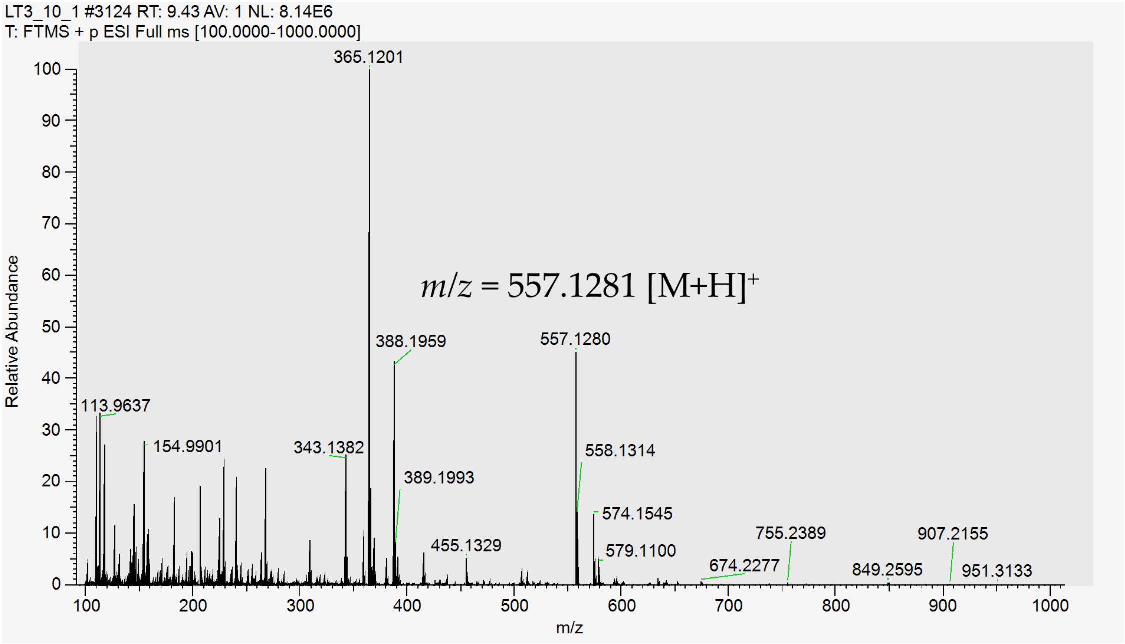

S91. +HRLC(ESI)MS spectrum of 2023_01a Peak 7

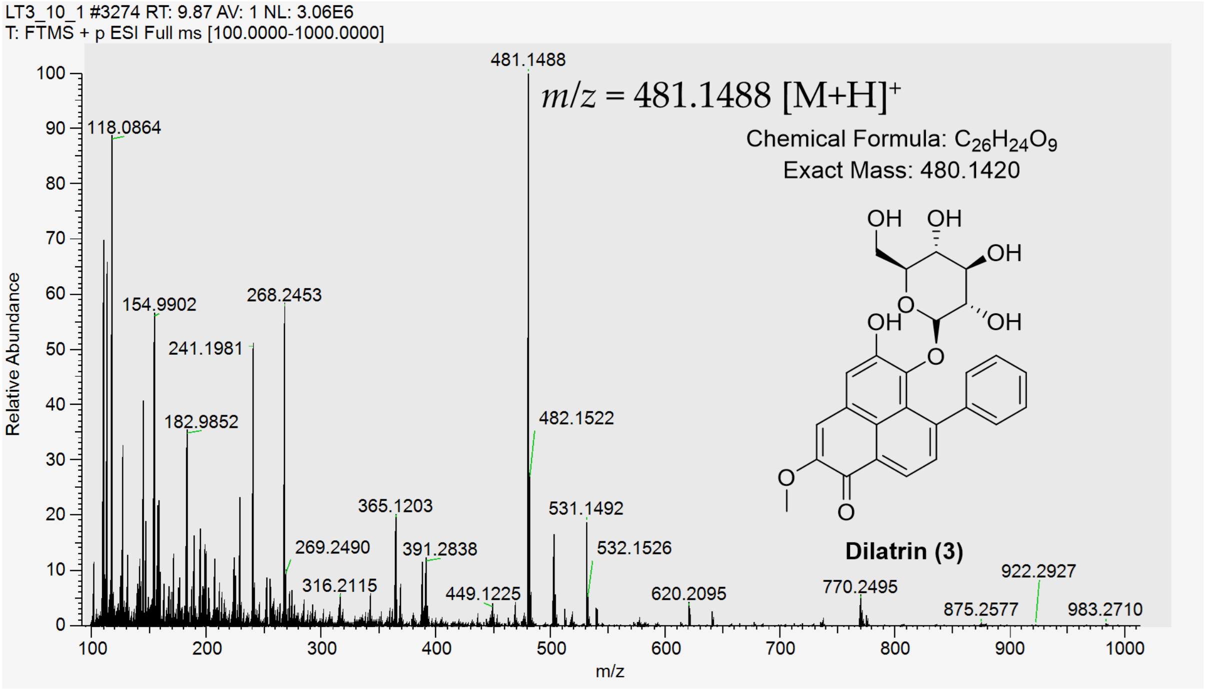

S92. +HRLC(ESI)MS spectrum of 2023_01a Peak 8

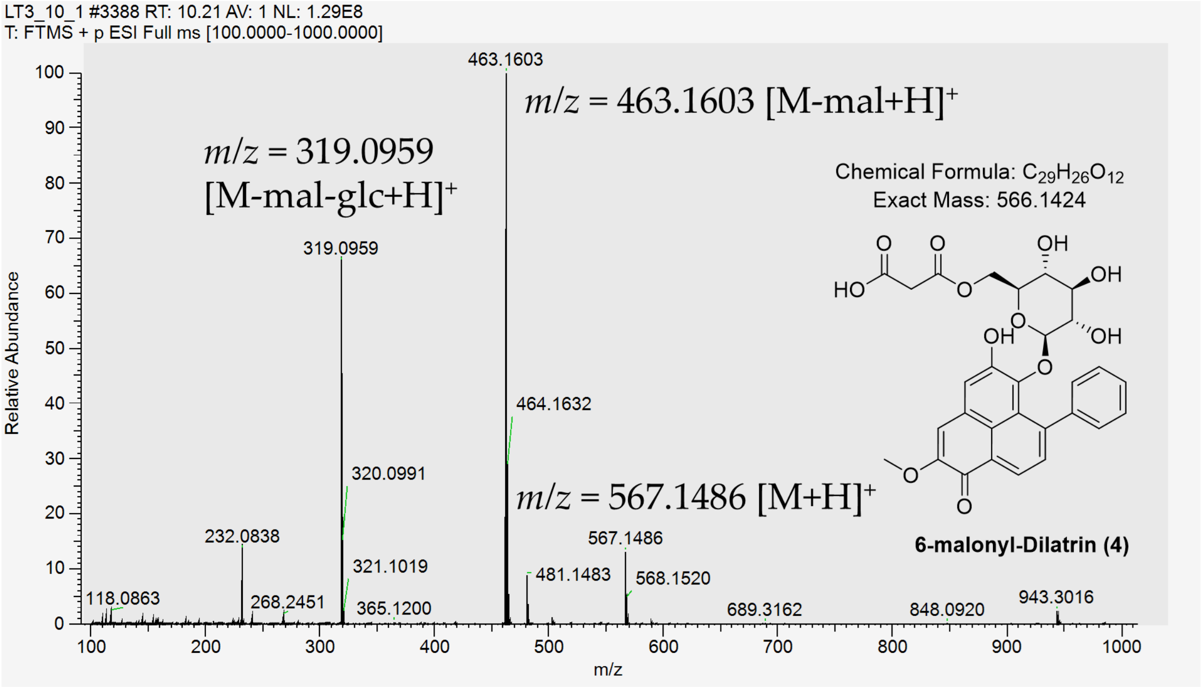

S93. +HRLC(ESI)MS spectrum of 2023_01a Peak 9

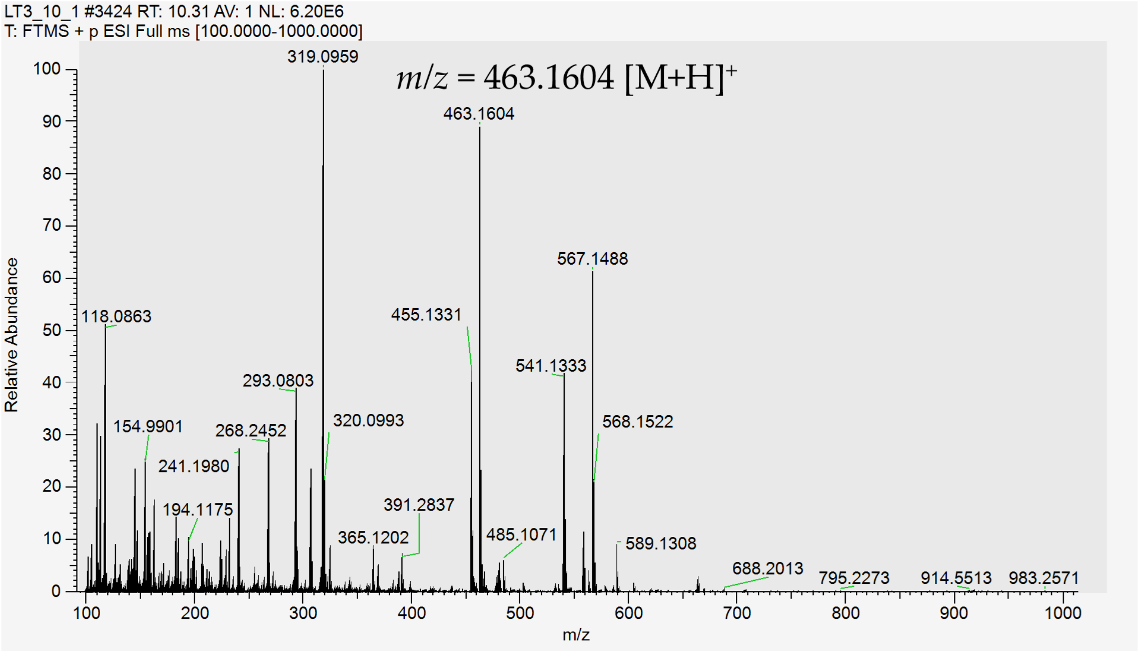

S94. +HRLC(ESI)MS spectrum of 2023_01a Peak 10

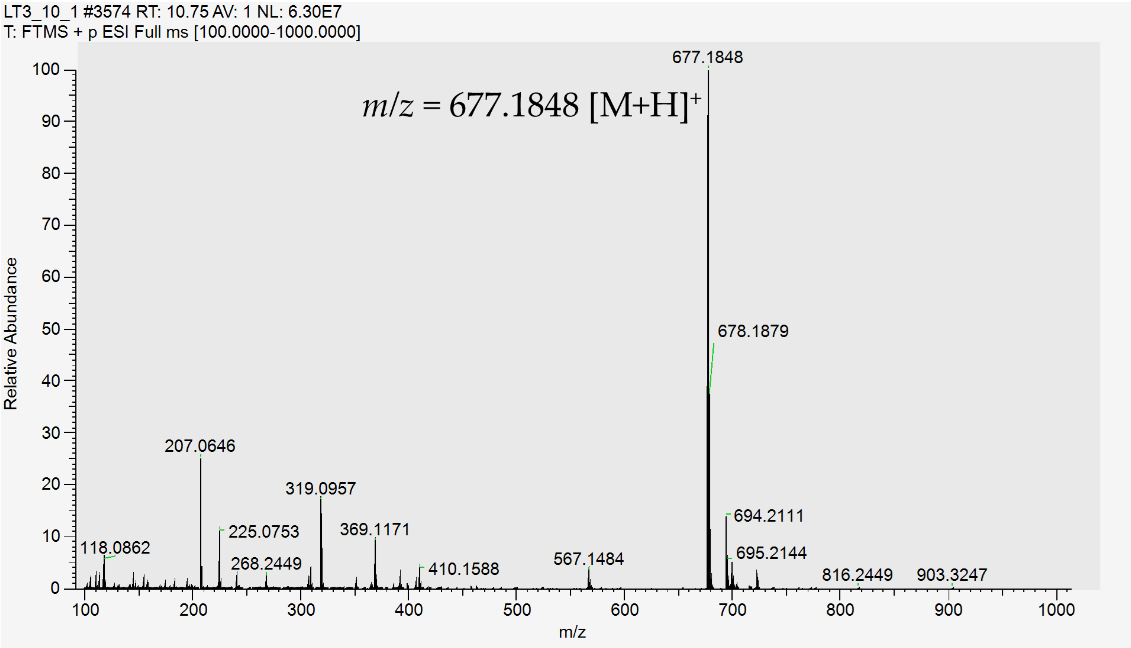

S95. +HRLC(ESI)MS spectrum of 2023_01a Peak 11

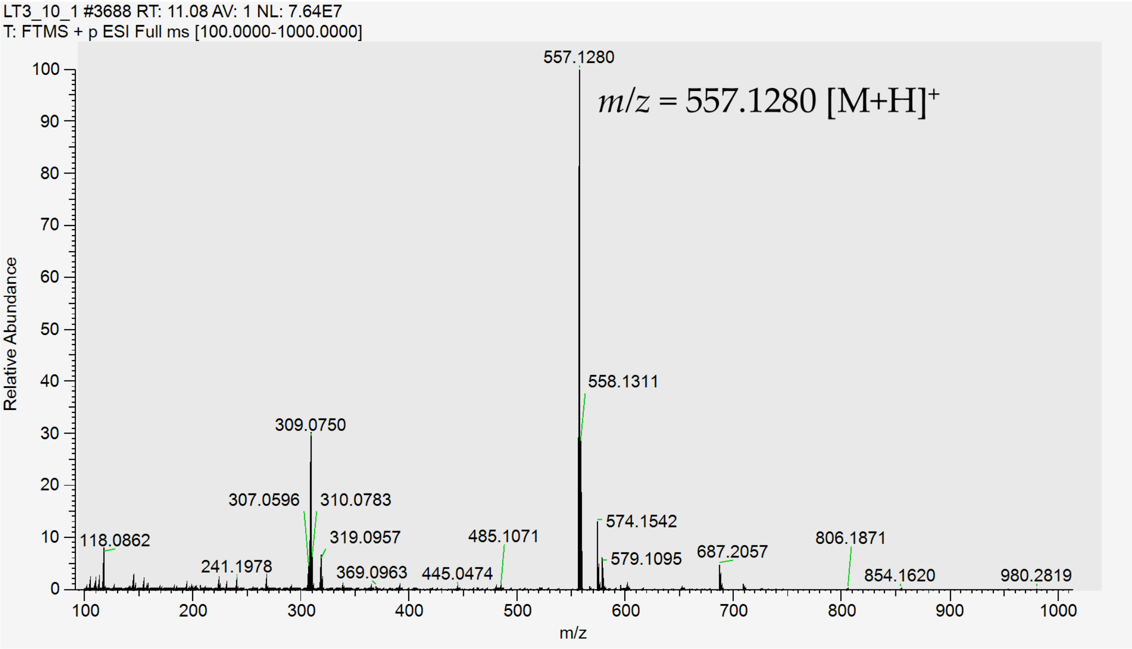

S96. +HRLC(ESI)MS spectrum of 2023_01a Peak 12

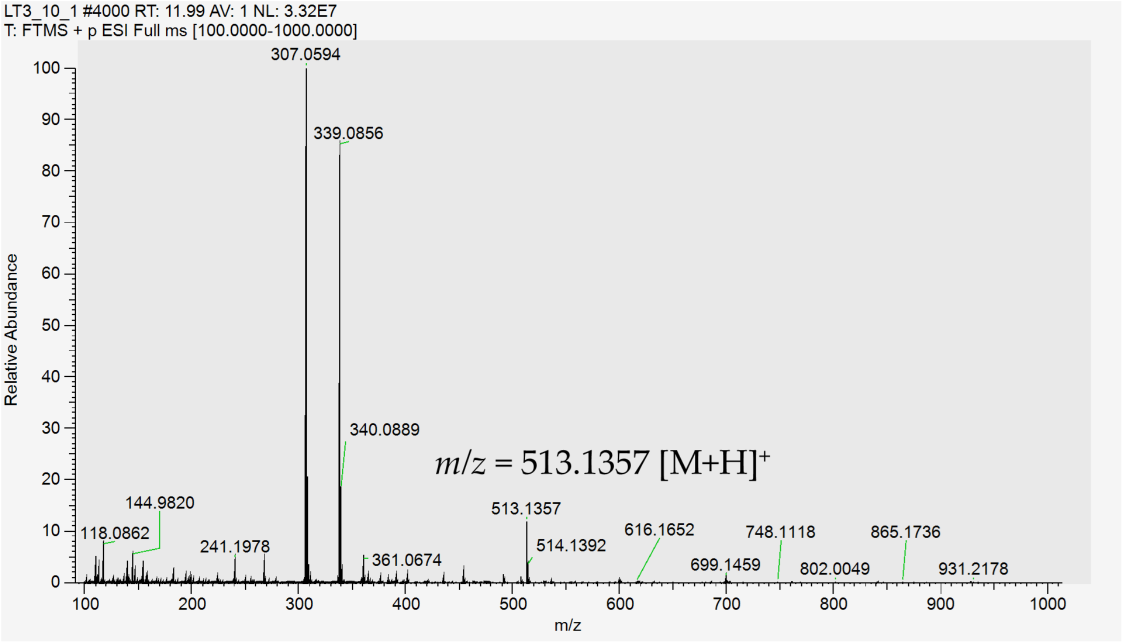

S97. +HRLC(ESI)MS spectrum of 2023_01a Peak 13

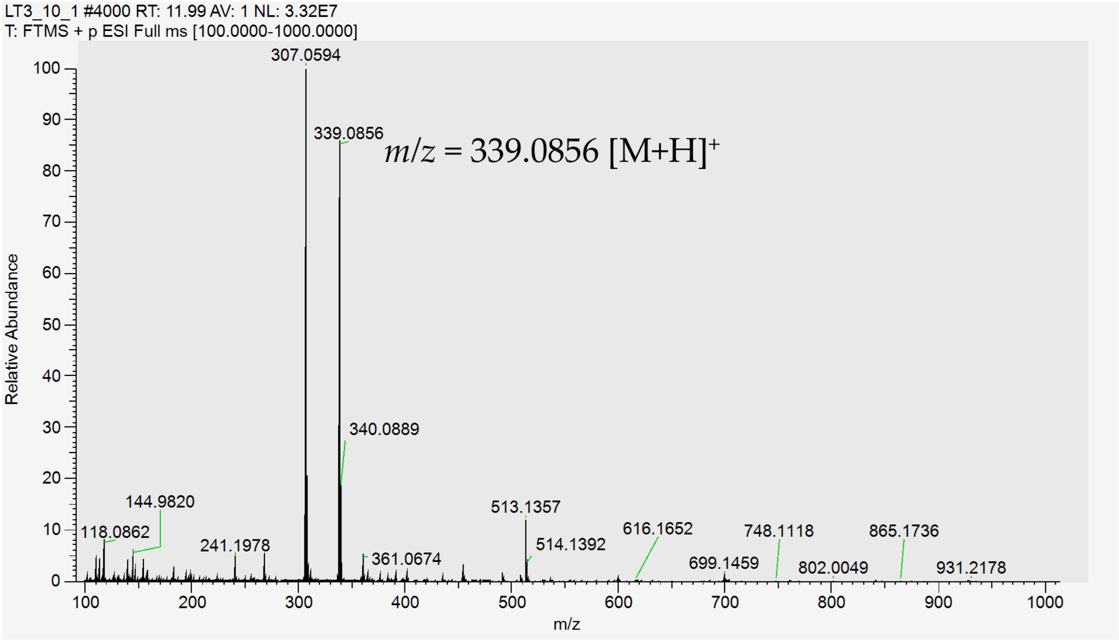

S98. +HRLC(ESI)MS spectrum of 2023_01a Peak 14

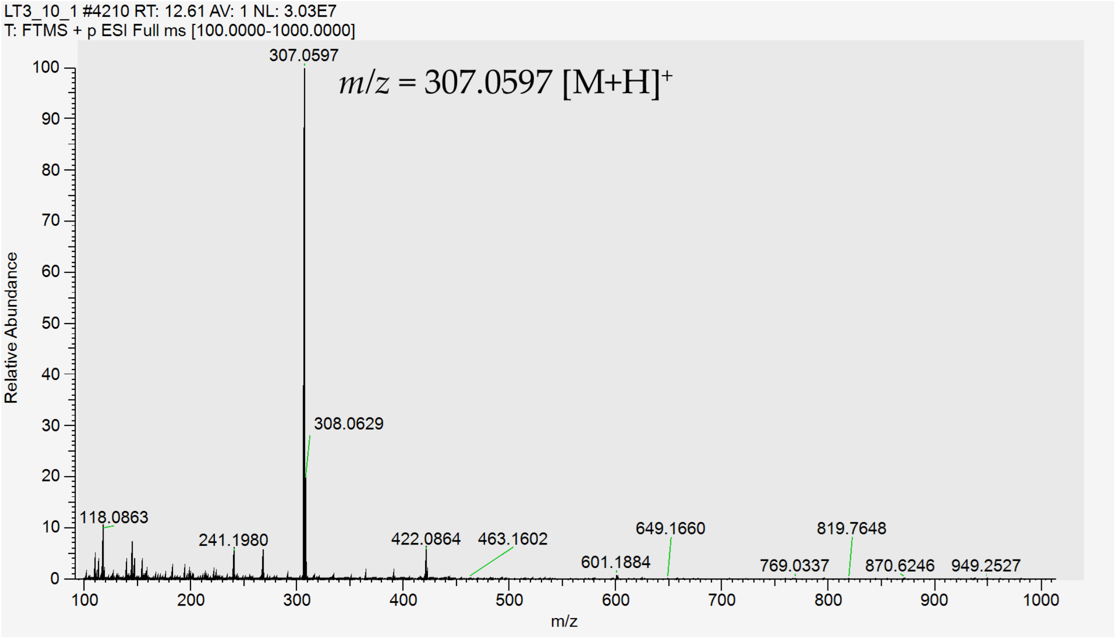

S99. +HRLC(ESI)MS spectrum of 2023_01a Peak 15

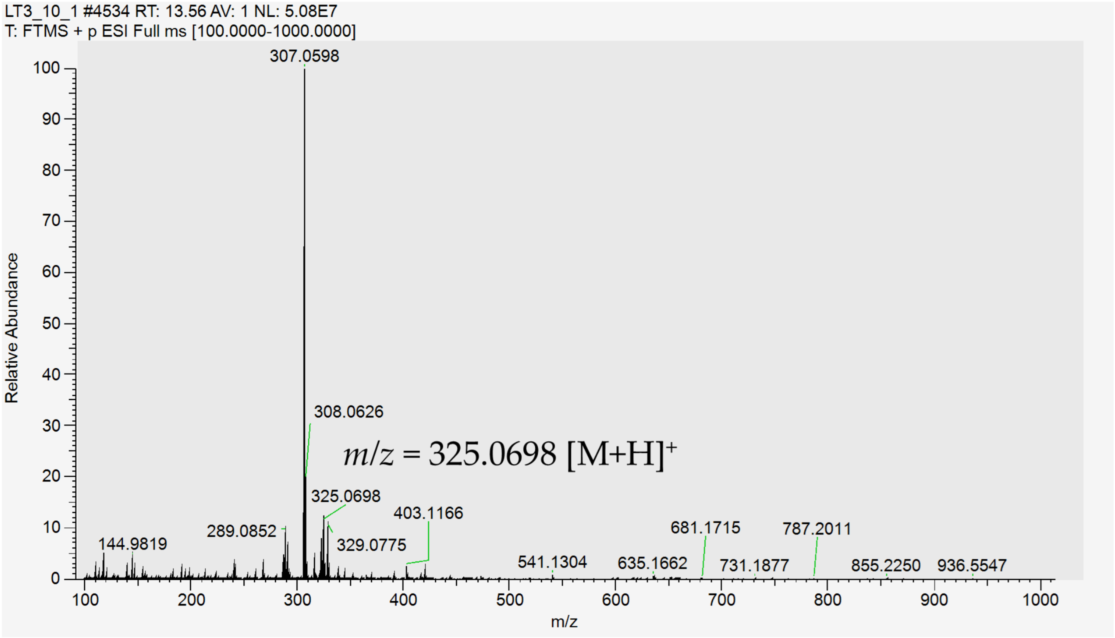

S100. +HRLC(ESI)MS spectrum of 2023_01a Peak 16

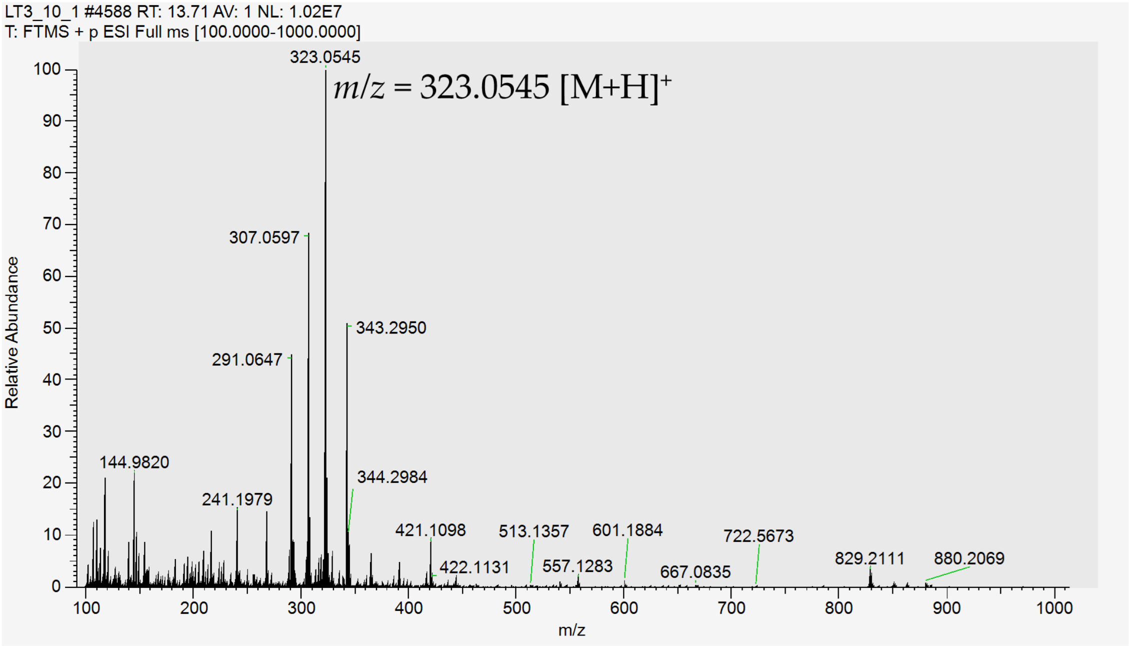

S101. +HRLC(ESI)MS spectrum of 2023_01a Peak 18

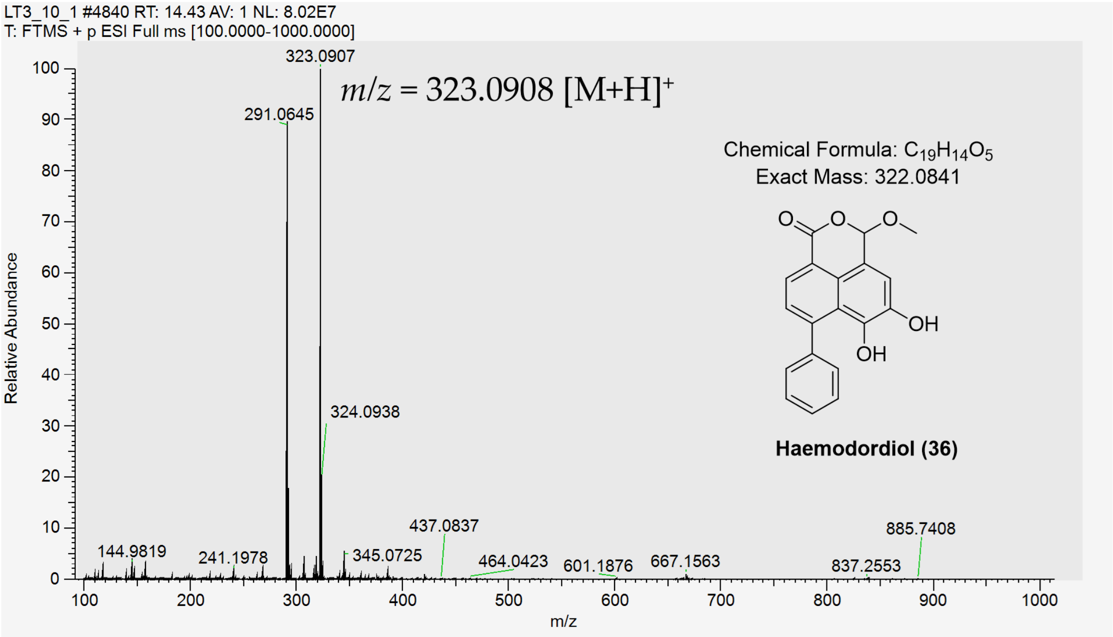

S102. +HRLC(ESI)MS spectrum of 2023_01a Peak 19

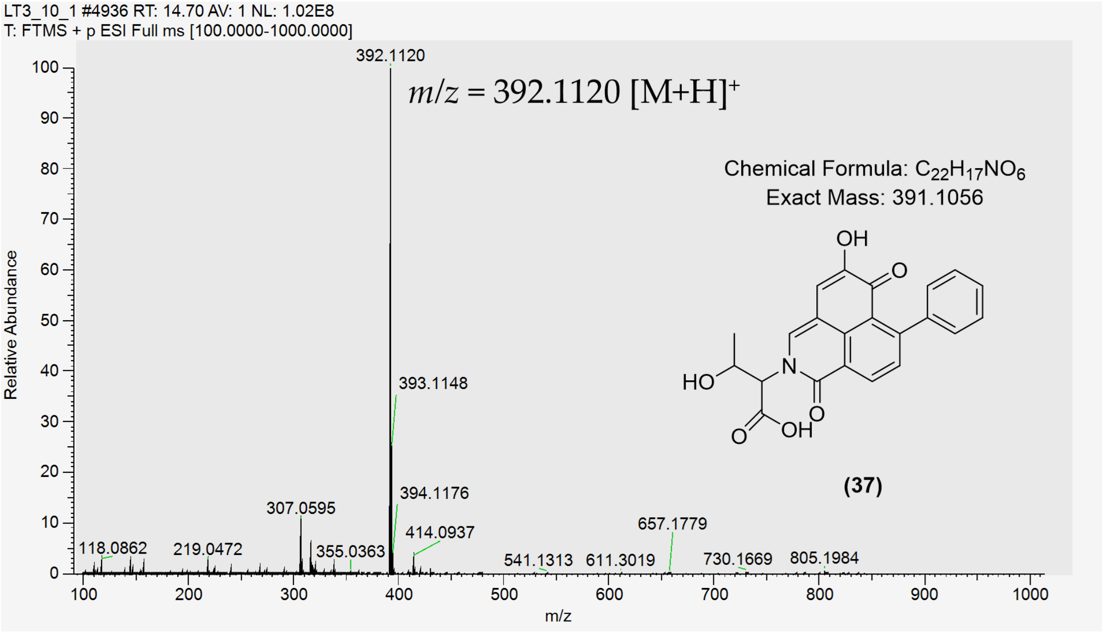

S103. +HRLC(ESI)MS spectrum of 2023_01a Peak 20

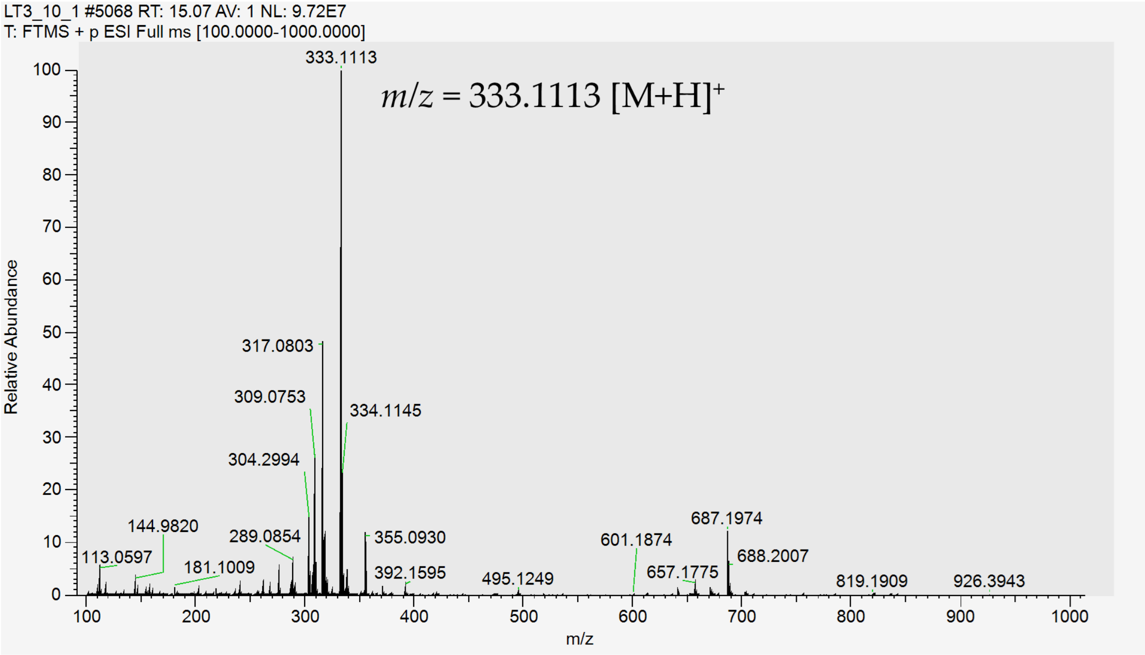

S104. +HRLC(ESI)MS spectrum of 2023_01a Peak 21

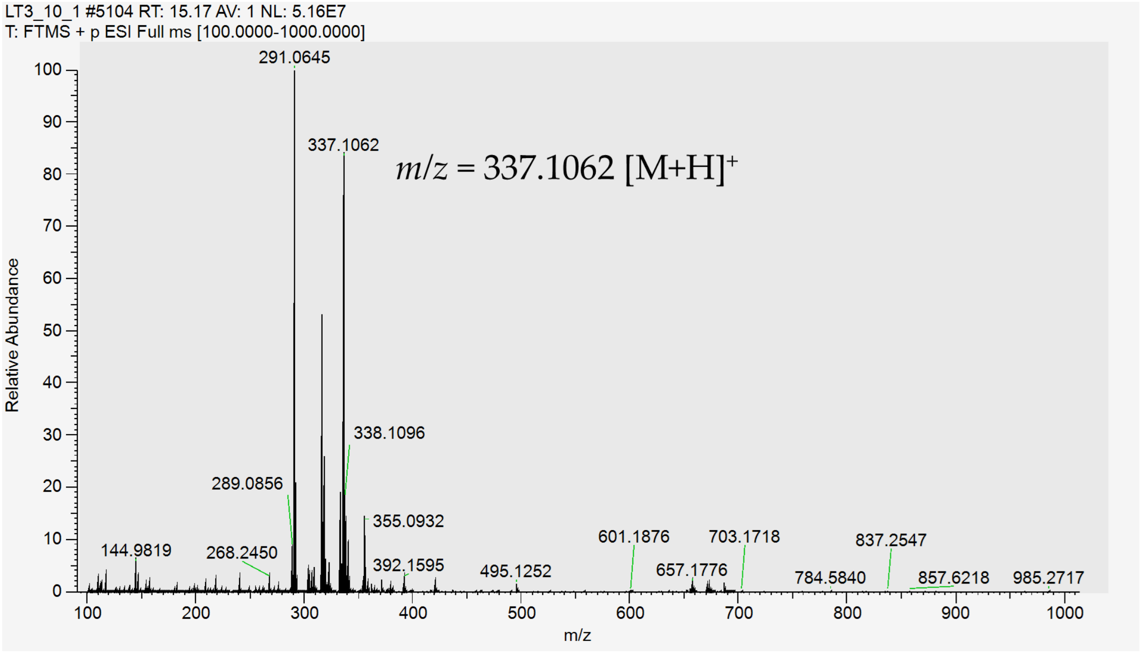

S105. +HRLC(ESI)MS spectrum of 2023_01a Peak 22

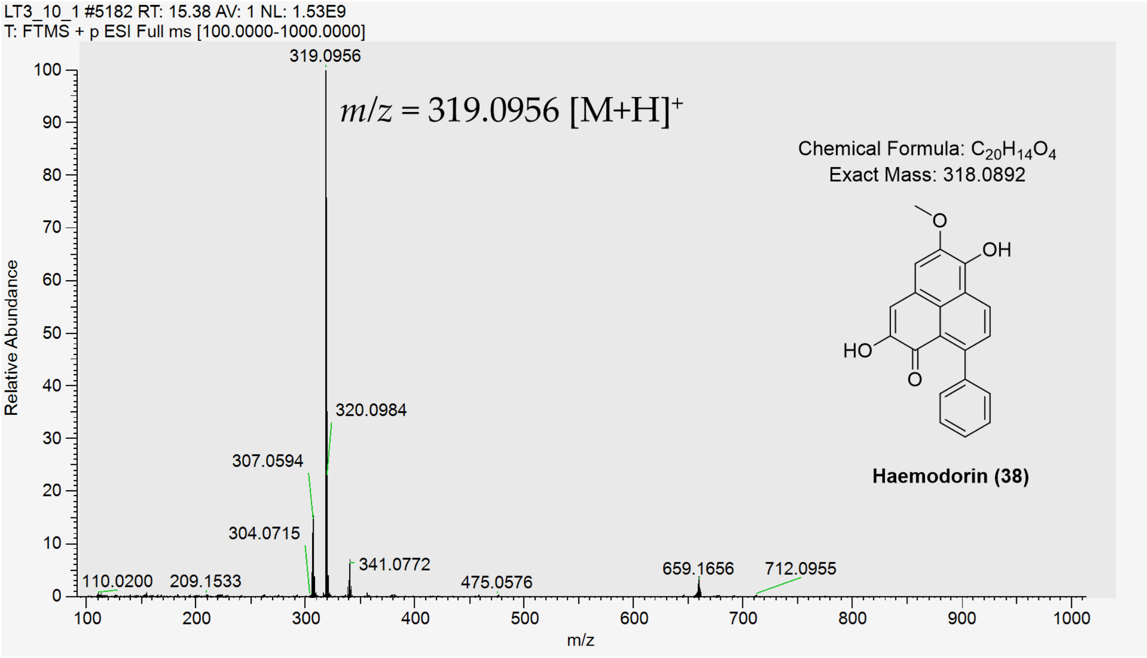

S106. +HRLC(ESI)MS spectrum of 2023_01a Peak 23

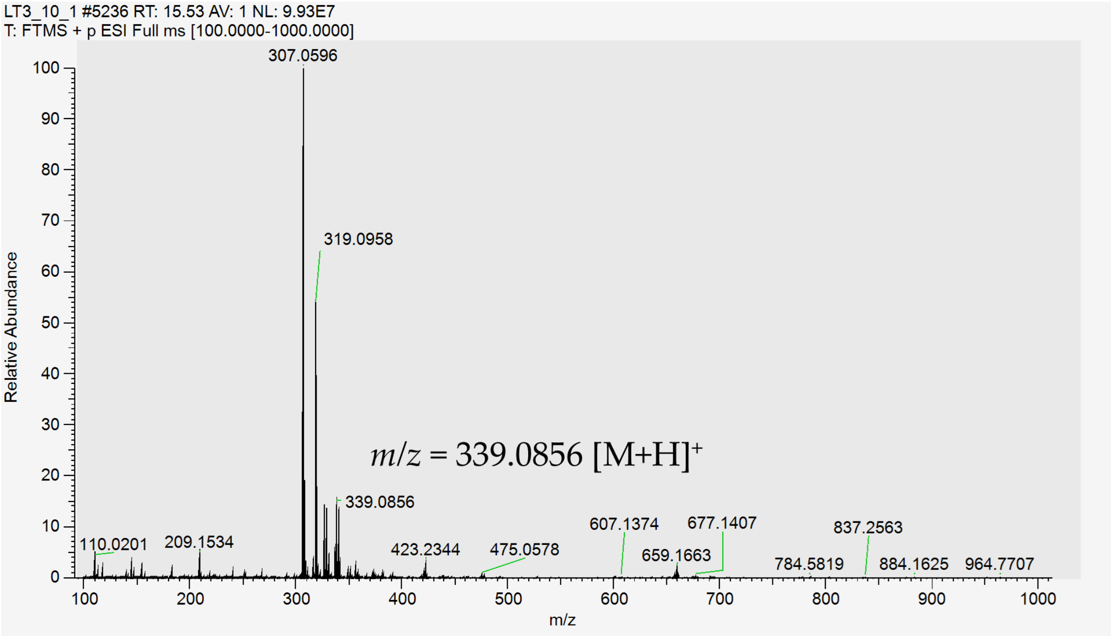

S107. +HRLC(ESI)MS spectrum of 2023_01a Peak 24

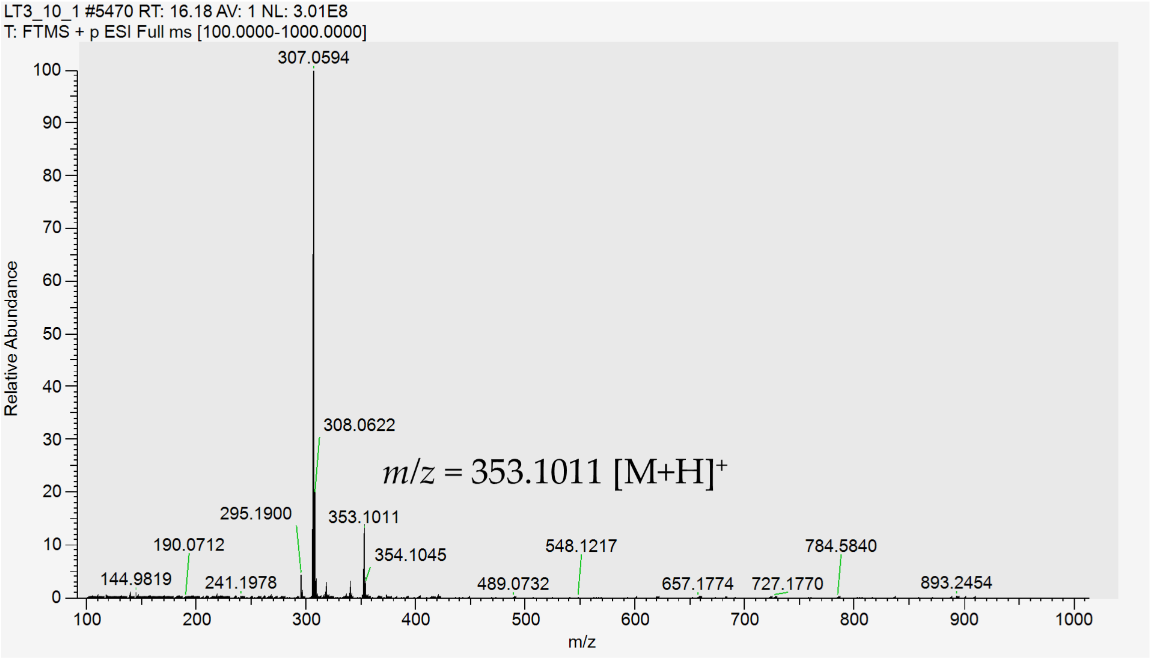

S108. +HRLC(ESI)MS spectrum of 2023_01a Peak 25

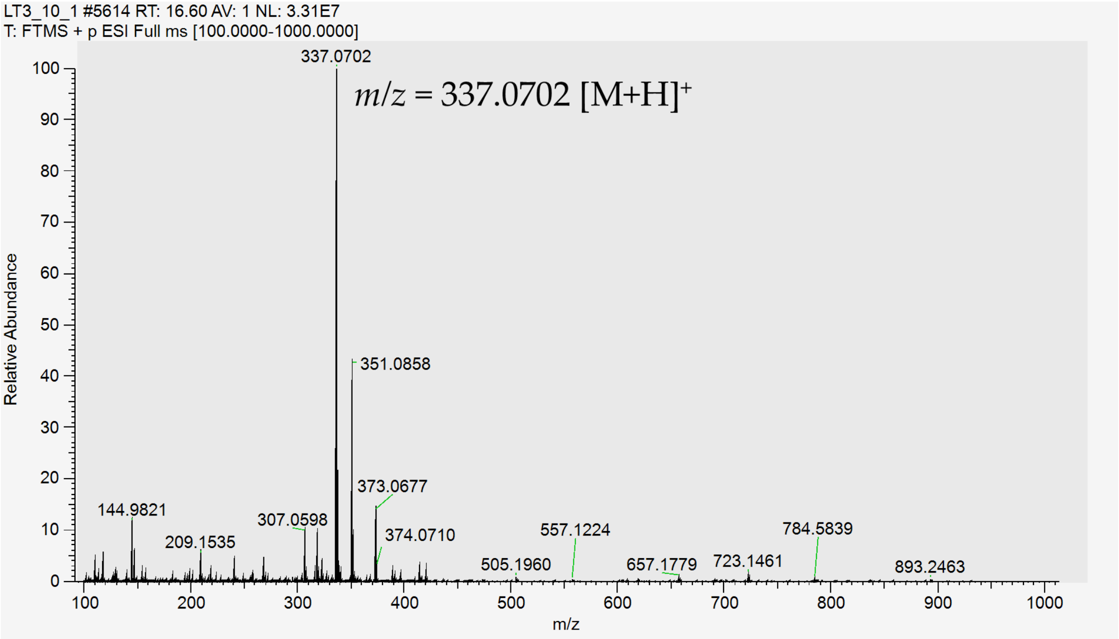

S109. +HRLC(ESI)MS spectrum of 2021_18c Peak 1

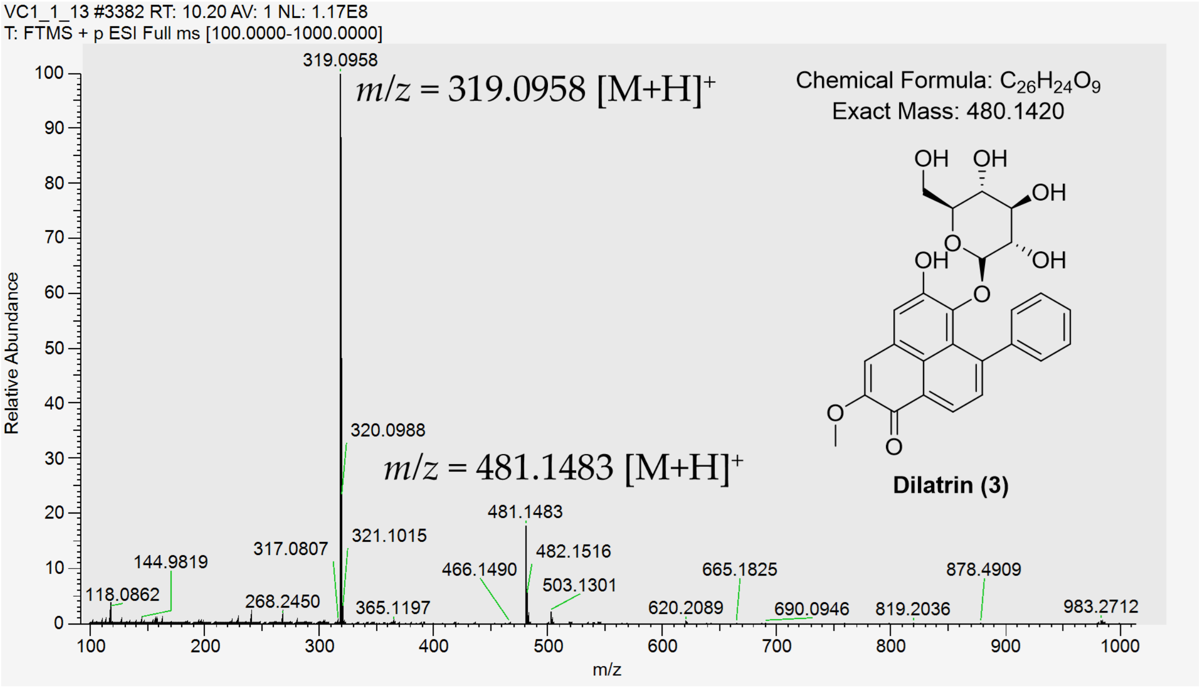

S110. +HRLC(ESI)MS spectrum of 2021_18c Peak 2

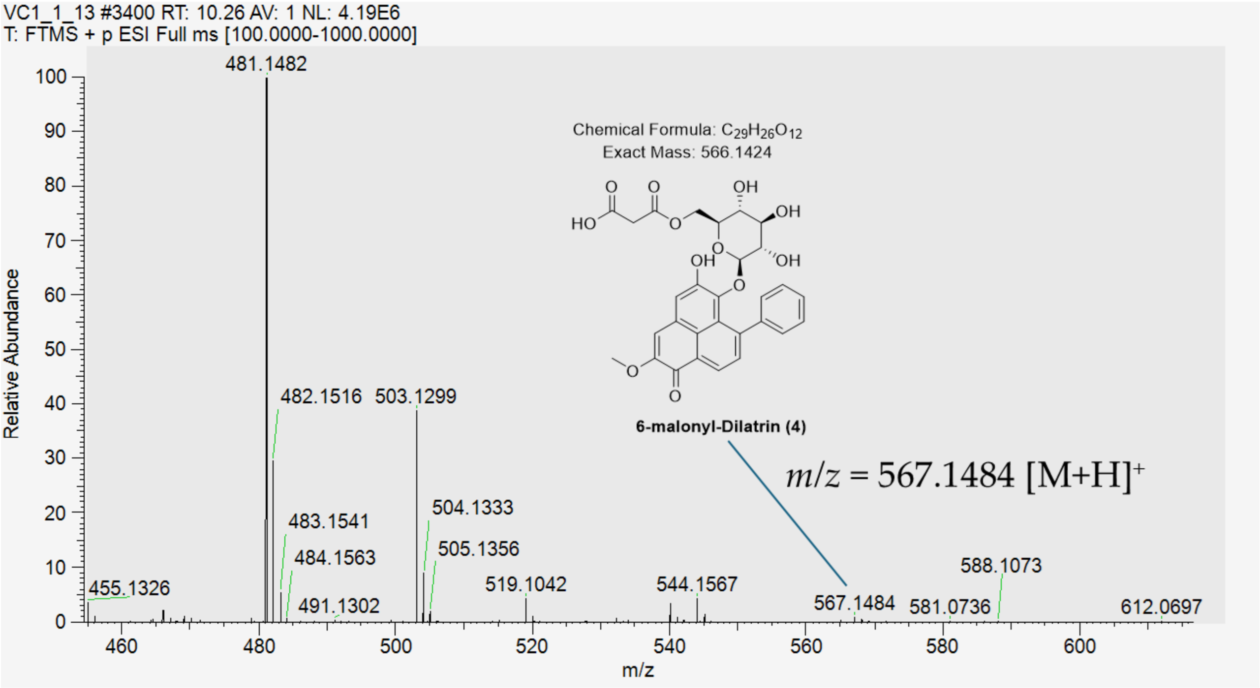

S111. +HRLC(ESI)MS spectrum of 2021_18c Peak 3

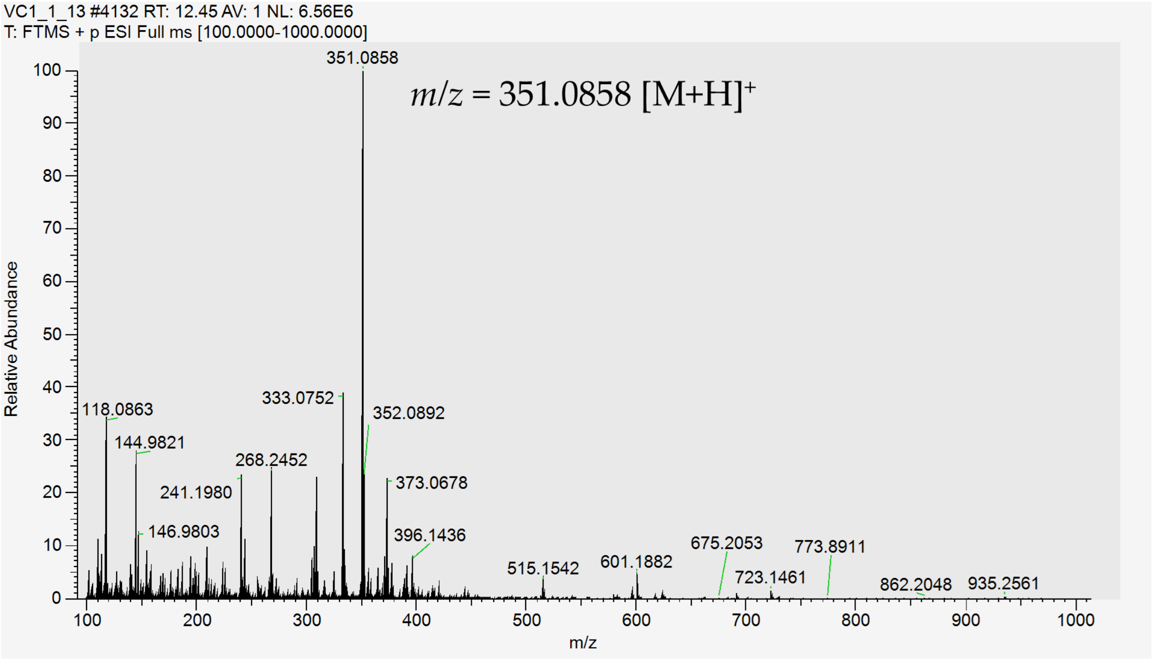

S112. +HRLC(ESI)MS spectrum of 2021_18c Peak 5

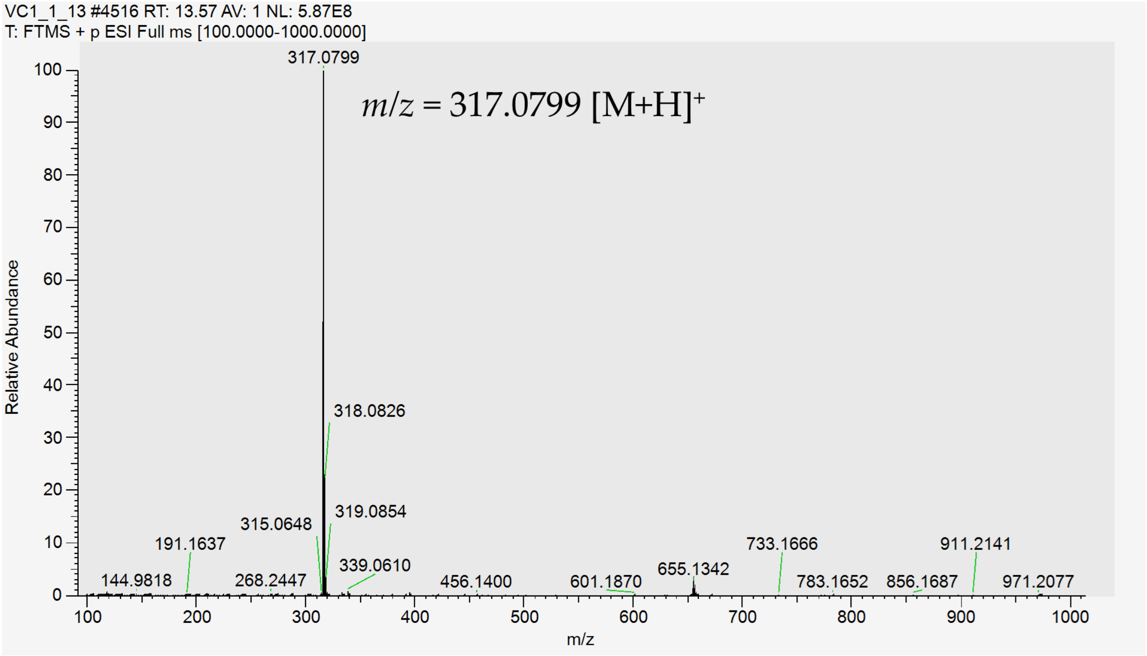

S113. +HRLC(ESI)MS spectrum of 2021_18c Peak 6

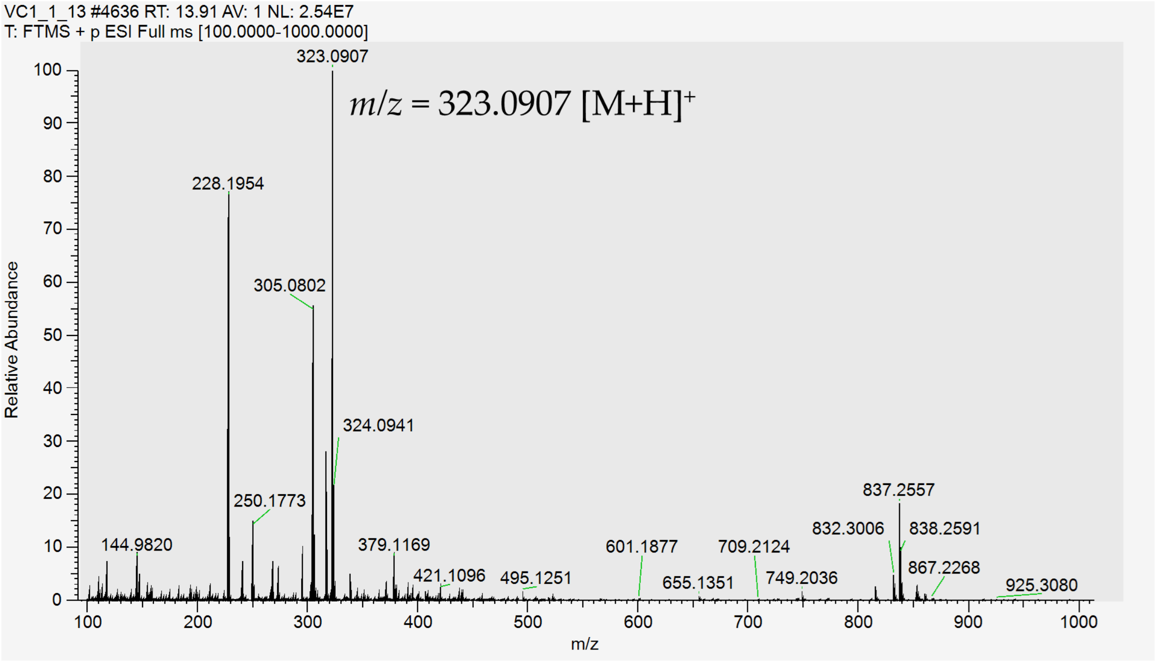

S114. +HRLC(ESI)MS spectrum of 2021_18c Peak 7

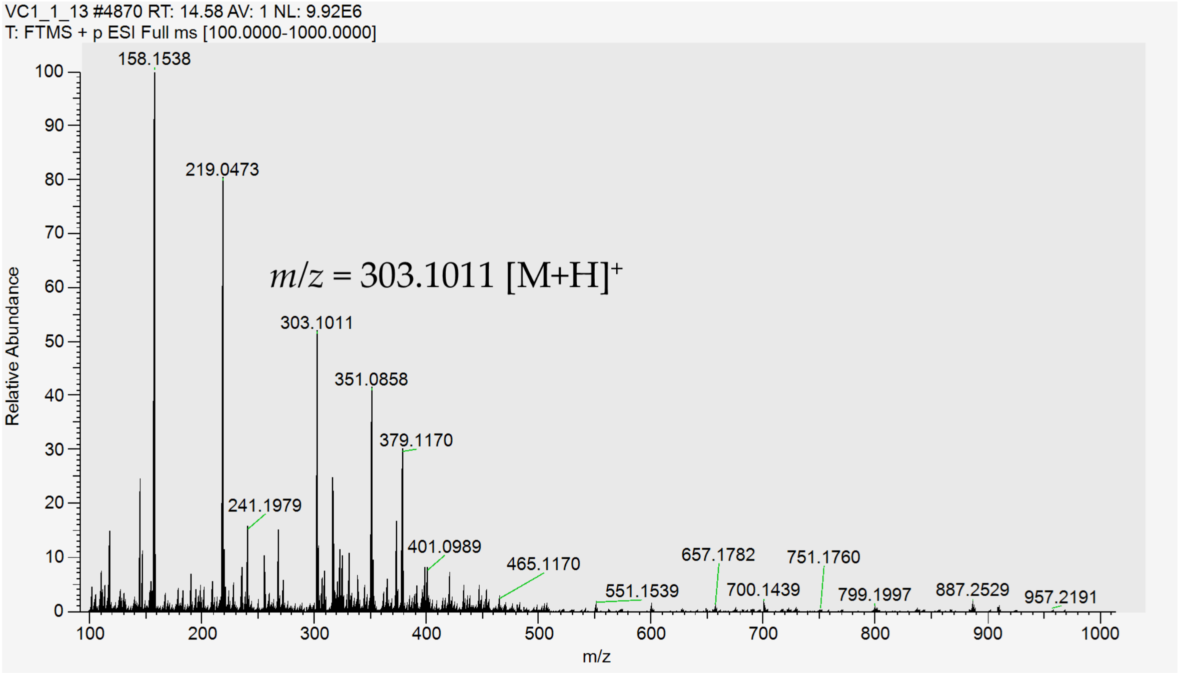

S115. +HRLC(ESI)MS spectrum of 2021_18c Peak 8

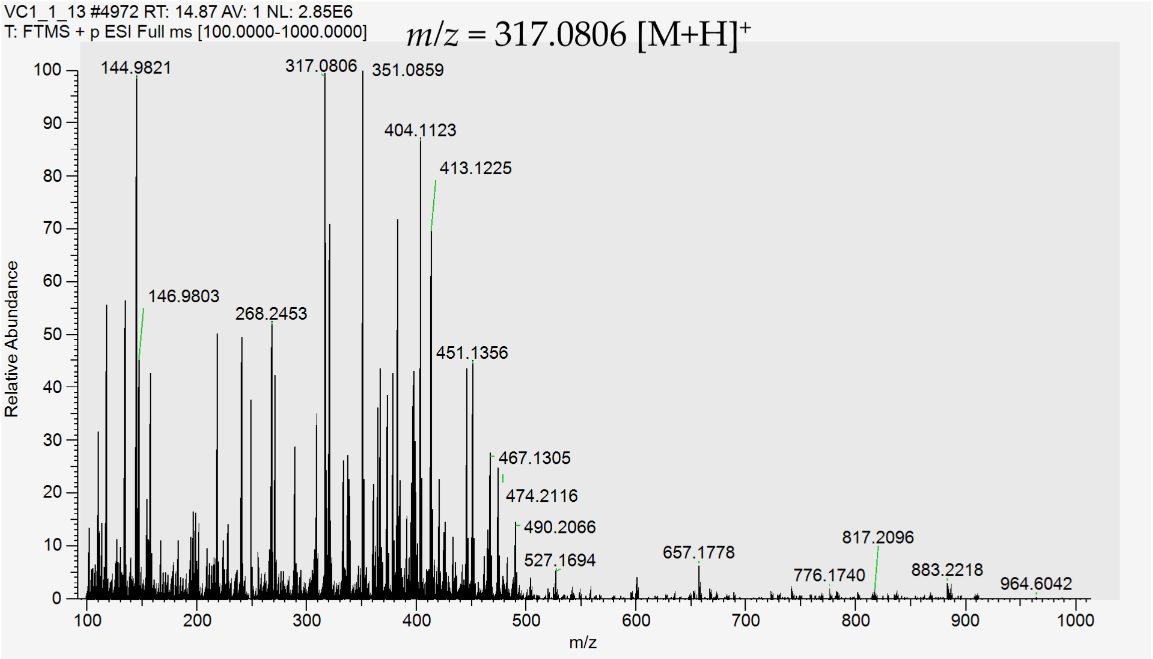

S116. +HRLC(ESI)MS spectrum of 2021_18c Peak 9

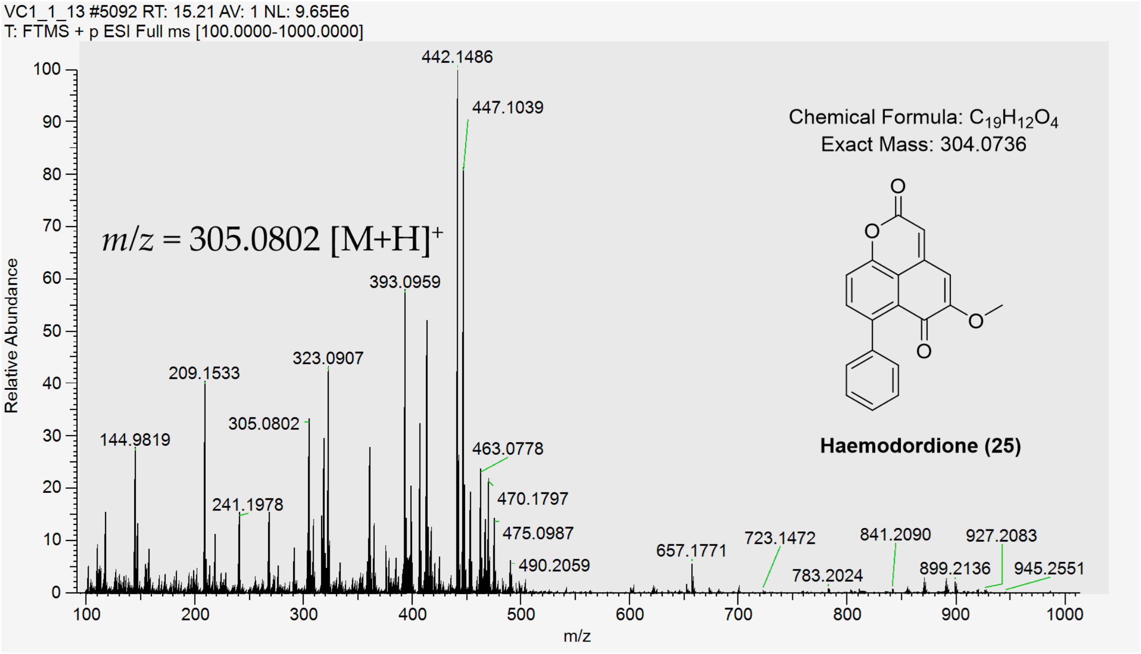

S117. +HRLC(ESI)MS spectrum of 2021_18c Peak 10

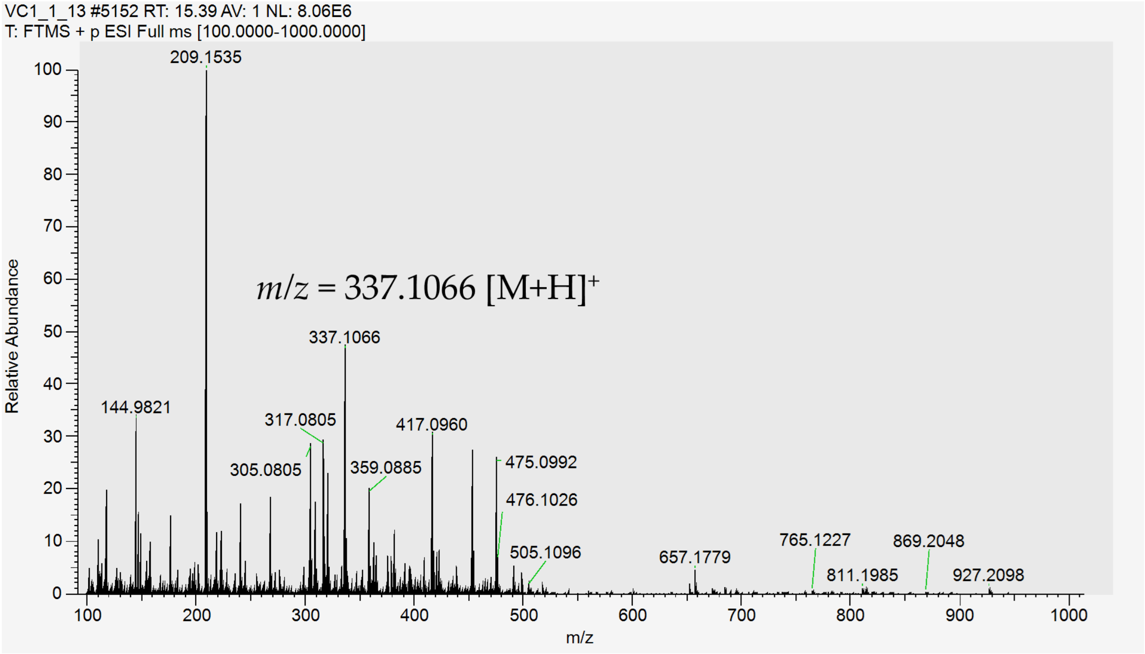

S118. +HRLC(ESI)MS spectrum of 2021_18c Peak 11

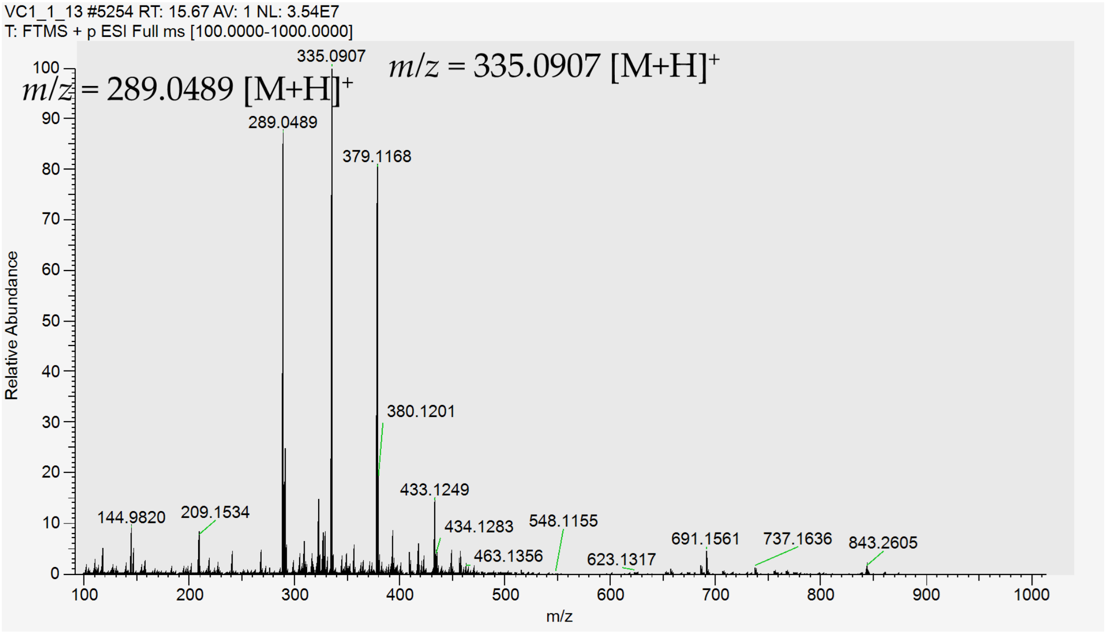

S119. +HRLC(ESI)MS spectrum of 2021_18c Peak 12

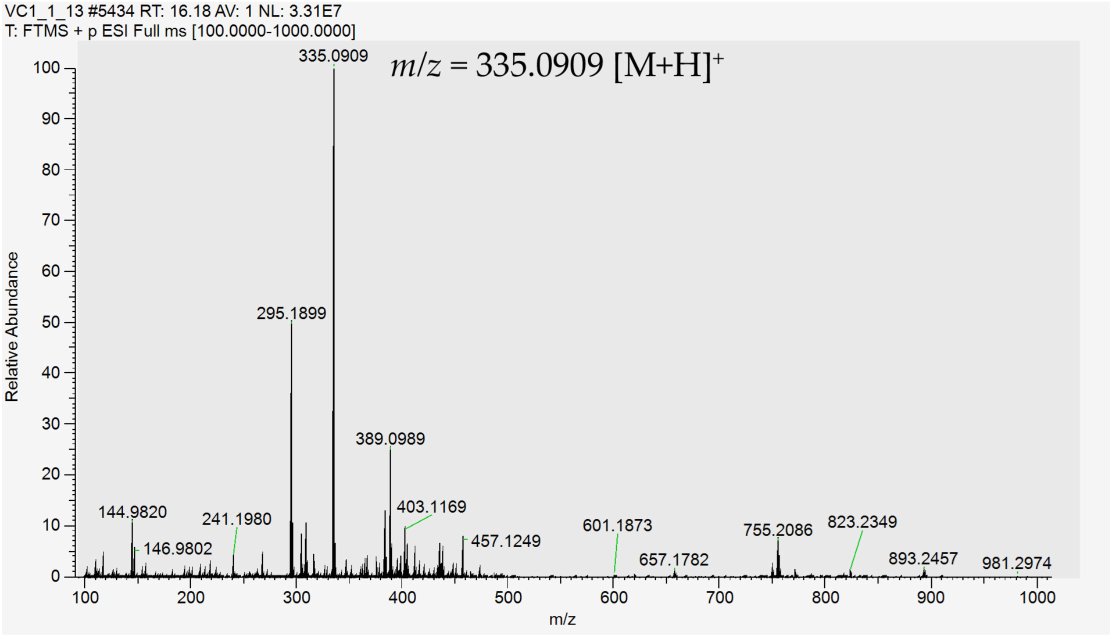

S120. +HRLC(ESI)MS spectrum of 2021_18c Peak 13

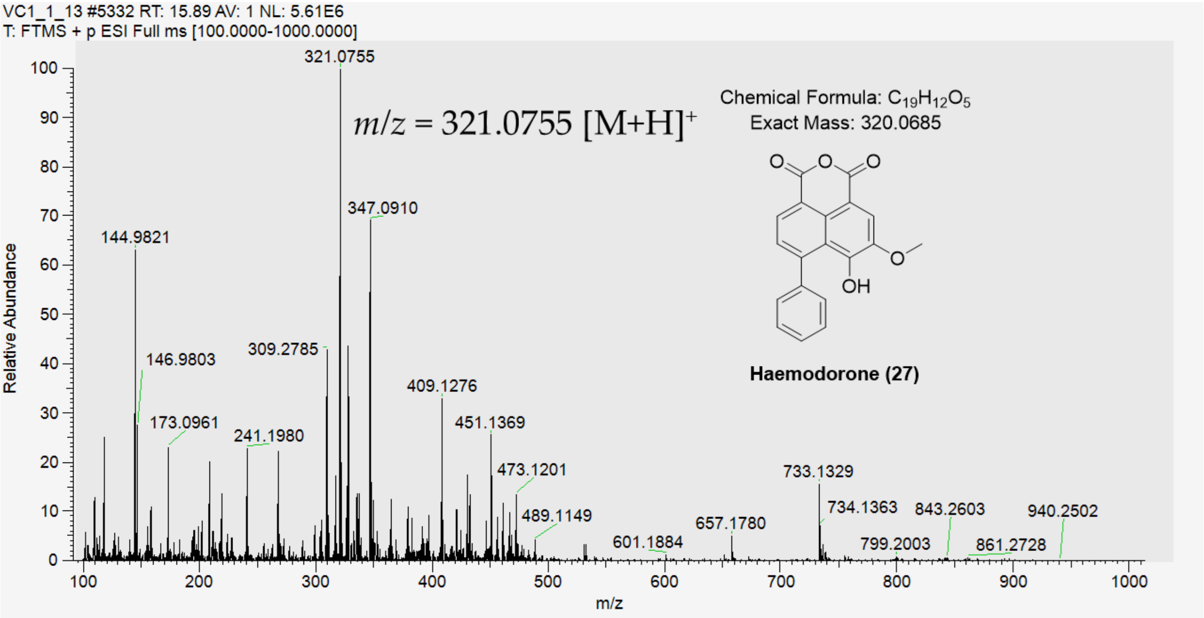

S121. +HRLC(ESI)MS spectrum of 2021_18c Peak 14

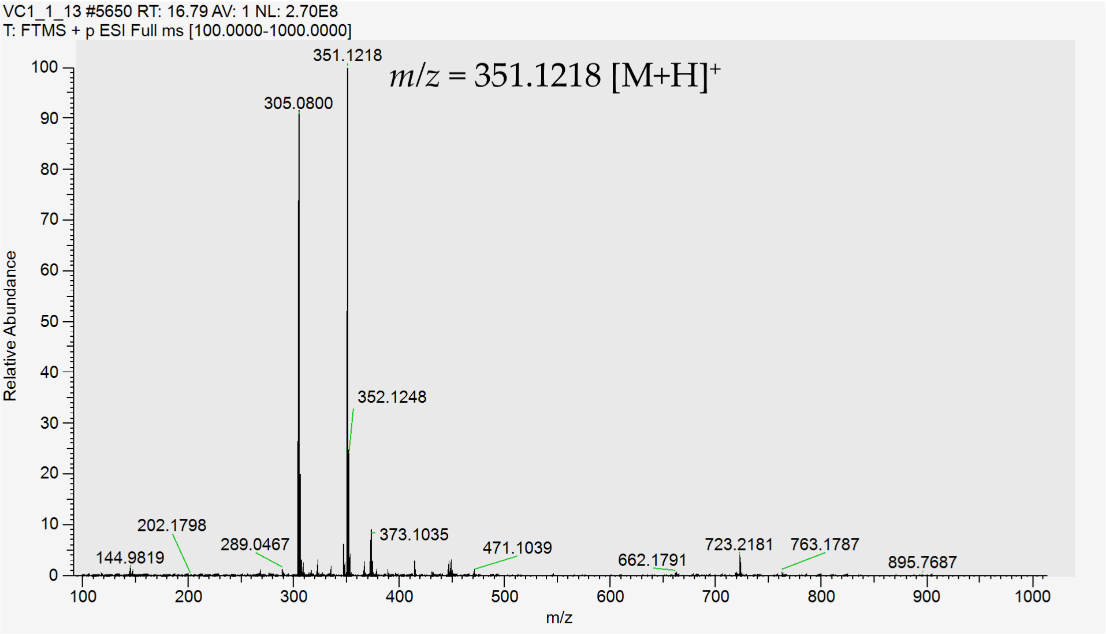

S122. Extract masses and yields from voucher samples

Species	Material Type (Source)	Extract mass (mg)	Extract yield (%) (2 g extraction)
H. simulans	Bulbs (2005_01a)	25.3	1.27
	Stems (2005_01b)	14.1	0.71
	Bulbs (2007_01a)	53.8	2.69
	Stems (2007_01b)	2.8	0.14
	Bulbs (2010_17a)	87.2	4.36
	Stems (2010_17b)	3.7	0.19
H. brevisepalum	Bulbs (2010_19a)	47.9	2.40
	Stems (2010_19b)	70.4	3.52
H. spicatum	Bulbs (2010_20a)	297.7	14.89
	Stems (2010_20b)	162.0	8.10
M. fuliginosa	Bulbs (2011_01a)	11.6	0.58
	Stems (2011_01b)	72.4	3.62
	Bulbs (2011_02a)	32.2	1.61
	Stems (2011_02b)	59.5	2.98
	Bulbs (2012_01a)	110.3	5.52
	Stems (2012_01b)	74.8	3.74
	Flowers (2012_05a)	16.8	0.84
	Stems/leaves (2012_05b)	19.9	1.00
	Stems (2012_05c)	56.3	2.82
	Flowers (2013_02)	11.5	0.58
H. coccineum	Leaves/stems (2021_17a)	4.2	0.21
	Roots (2021_17b)	2.0	0.10
	Leaves/bulbs (2022_08)	12.1	0.61
	Bulbs (2023_01a)	114.1	5.71
	Stems (2023_01b)	8.7	0.44
H. distichophyllum	Leaves (2021_18a)	3.2	0.16
	Flowers/seeds (2021_18b)	3.4	0.17
	Roots (2021_18c)	1.7	0.09
	Leaves (2022_07a)	4.4	0.22
	Roots/bulbs (2022_07b)	0.9	0.05
